# Supplementary material for: Age-period-cohort analysis and projection of cancer mortality in Hong Kong, 1998–2030
Source: BMJ Open. 2023 Oct 11;13(10):e072751. doi: 10.1136/bmjopen-2023-072751 (PMC10583025; doi:10.1136/bmjopen-2023-072751)
Supplement: Supplementary data [file bmjopen-2023-072751supp001.pdf]

Supplementary Material for

"An age-period-cohort analysis and projection of cancer mortality in Hong Kong, 1998–2030"

APC Figures

eFigure 1(a) Colon cancer 3
eFigure 1(b) Liver cancer 4
eFigure 1(c) Pancreatic cancer 5
eFigure 1(d) Stomach cancer 6
eFigure 1(e) Prostate cancer 7

Projection Figures

Colon cancer
eFigure 2(a)Projection (all ages) 8
eFigure 2(b)Projection (<60 years) 9
eFigure 2(c)Projection (⩾60 years) 10
Liver cancer
eFigure 3(a)Projection (all ages) 11
eFigure 3(b)Projection (<60 years) 12
eFigure 3(c)Projection (⩾60 years) 13
Pancreatic cancer
eFigure 4(a)Projection (all ages) 14
eFigure 4(b)Projection (<60 years) 15
eFigure 4(c)Projection (⩾60 years) 16
Stomach cancer
eFigure 5(a)Projection (all ages) 17
eFigure 5(b)Projection (<60 years) 18

Zhao Y, *et al. BMJ Open* 2023; 13:e072751. doi: 10.1136/bmjopen-2023-072751

eFigure 5(c)Projection (⩾60 years) ... 19
Prostate cancer
eFigure 6 Projection (all ages, <60 years and ⩾60 years) ... 20

Tables

eTable 1 Lung cancer ... 21
eTable 2 Colon cancer ... 22
eTable 3 Liver cancer ... 23
eTable 4 Pancreatic cancer ... 24
eTable 5 Stomach cancer ... 25
eTable 6 Prostate cancer ... 26

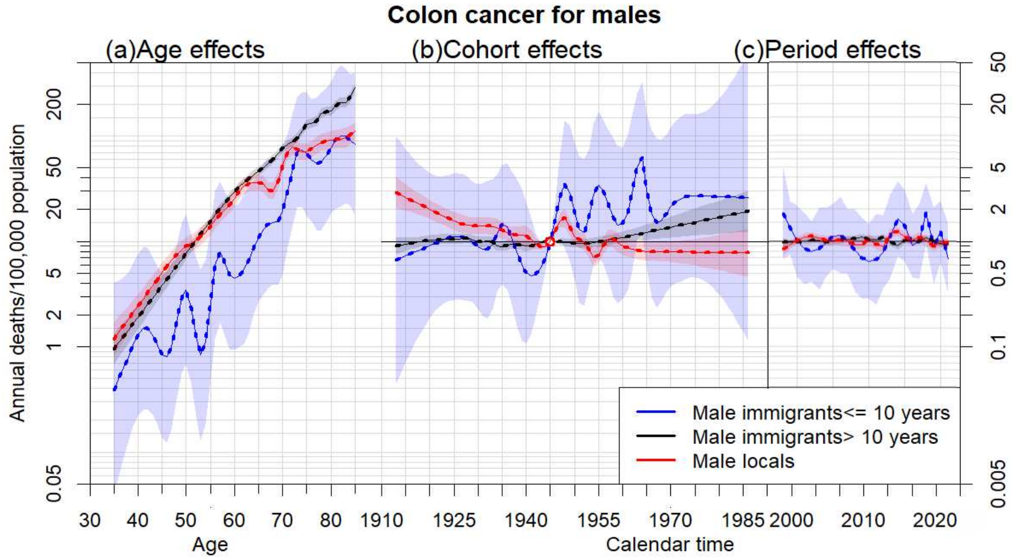

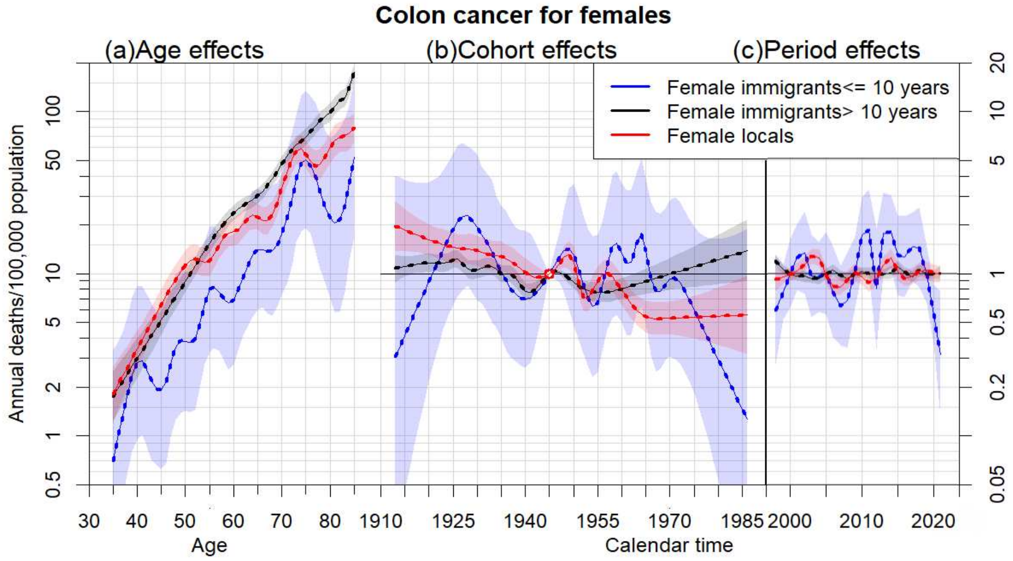

eFigure 1(a). Parameter estimates of age (a), cohort (b) and period (c) effects based on an age-period-cohort model of male and female colon cancer mortality rates by immigrant groups: locals, immigrants stay in Hong Kong for more than 10 years and immigrants stay in Hong Kong for less than or equal to 10 years. Age effect was assessed by mortality (left axis). Cohort and period effects were assessed by relative risk (right axis), 95% confidence intervals are shown as shaded bands.

Zhao Y, *et al. BMJ Open* 2023; 13:e072751. doi: 10.1136/bmjopen-2023-072751

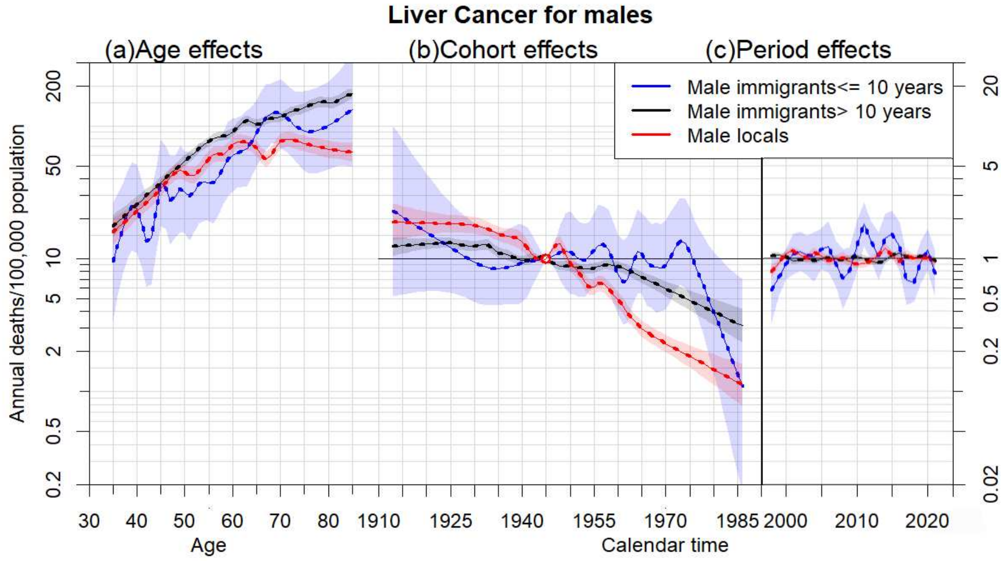

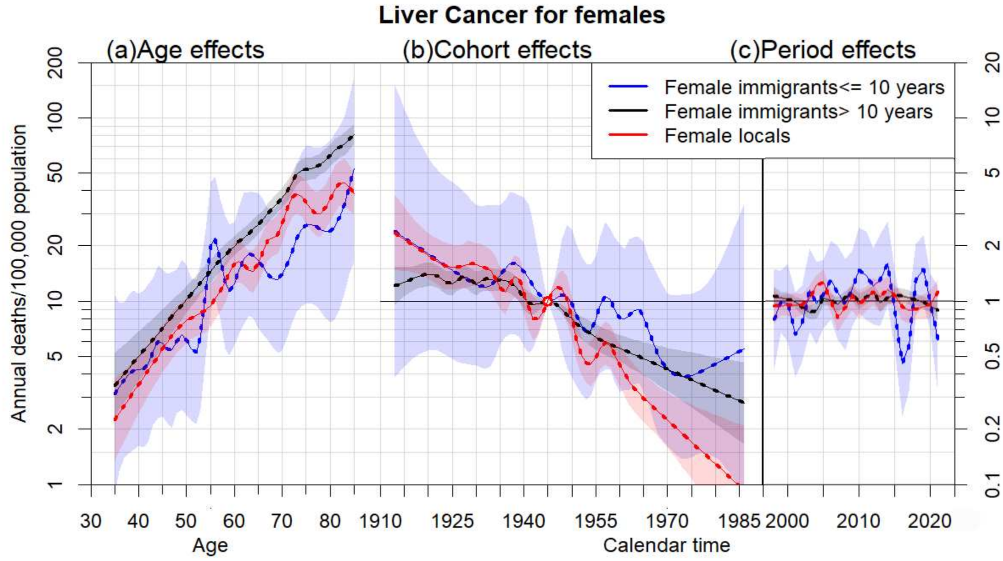

eFigure 1(b). Parameter estimates of age (a), cohort (b) and period (c) effects based on an age-period-cohort model of male and female liver cancer mortality rates by immigrant groups: locals, immigrants stay in Hong Kong for more than 10 years and immigrants stay in Hong Kong for less than or equal to 10 years. Age effect was assessed by mortality (left axis). Cohort and period effects were assessed by relative risk (right axis), 95% confidence intervals are shown as shaded bands.

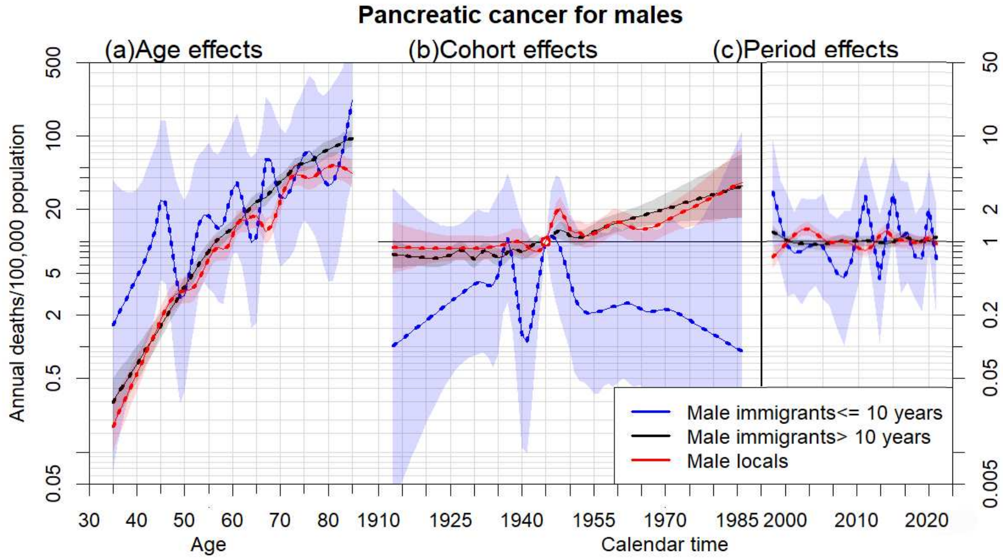

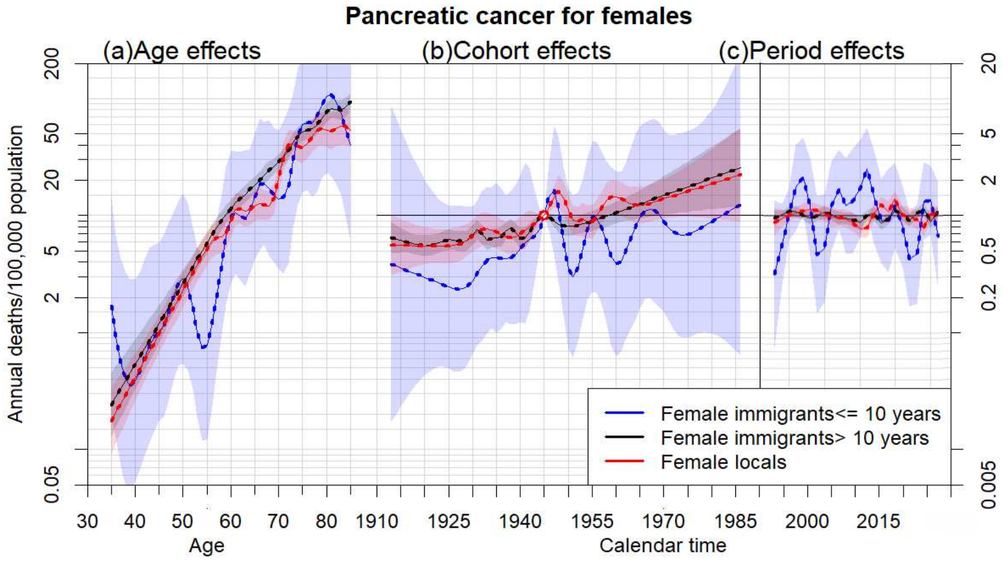

eFigure 1(c). Parameter estimates of age (a), cohort (b) and period (c) effects based on an age-period-cohort model of male and female pancreatic cancer mortality rates by immigrant groups: locals, immigrants stay in Hong Kong for more than 10 years and immigrants stay in Hong Kong for less than or equal to 10 years. Age effect was assessed by mortality (left axis). Cohort and period effects were assessed by relative risk (right axis), 95% confidence intervals are shown as shaded bands.

Zhao Y, *et al. BMJ Open* 2023; 13:e072751. doi: 10.1136/bmjopen-2023-072751

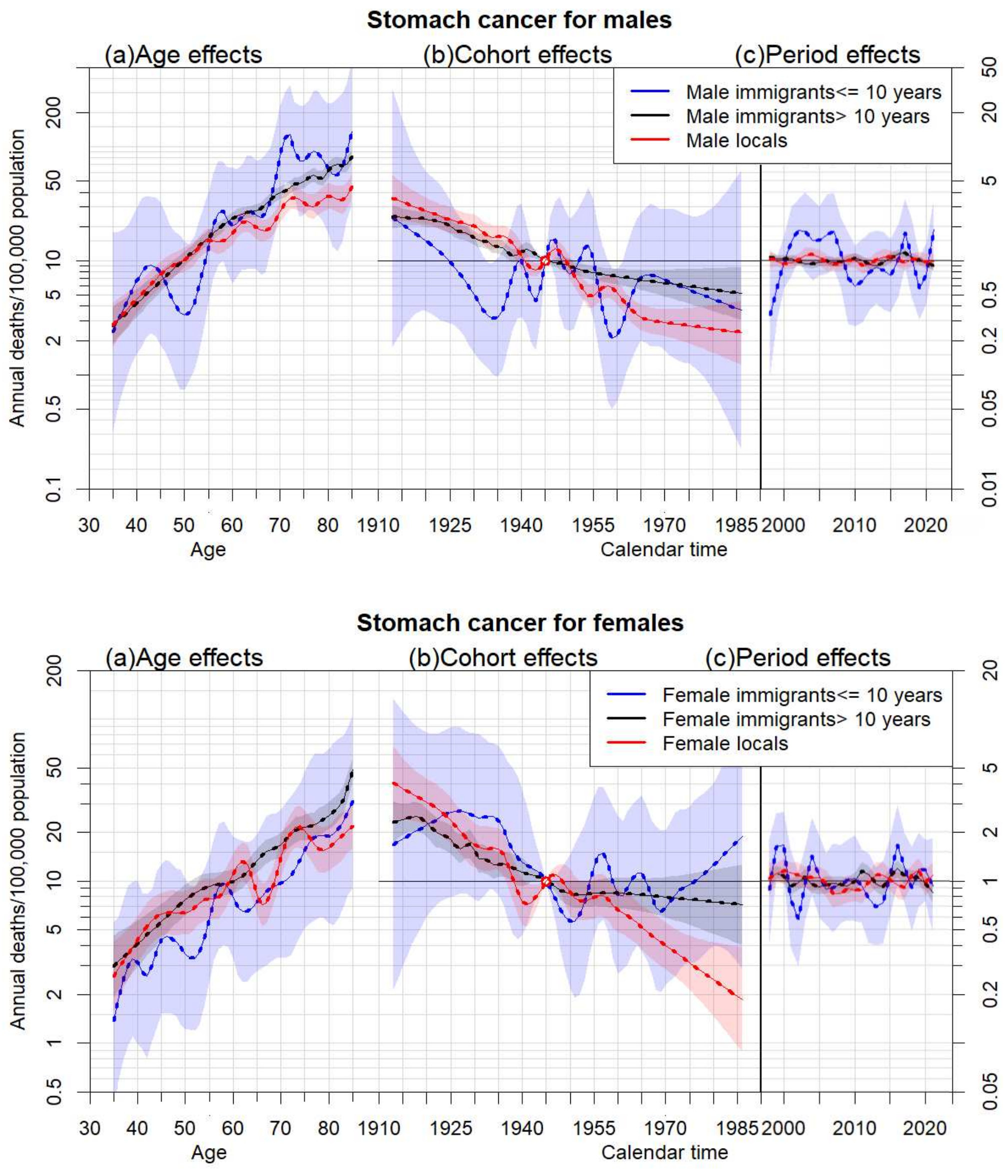

eFigure 1(d). Parameter estimates of age (a), cohort (b) and period (c) effects based on an age-period-cohort model of male and female stomach cancer mortality rates by immigrant groups: locals, immigrants stay in Hong Kong for more than 10 years and immigrants stay in Hong Kong for less than or equal to 10 years. Age effect was assessed by mortality (left axis). Cohort and period effects were assessed by relative risk (right axis), 95% confidence intervals are shown as shaded bands.

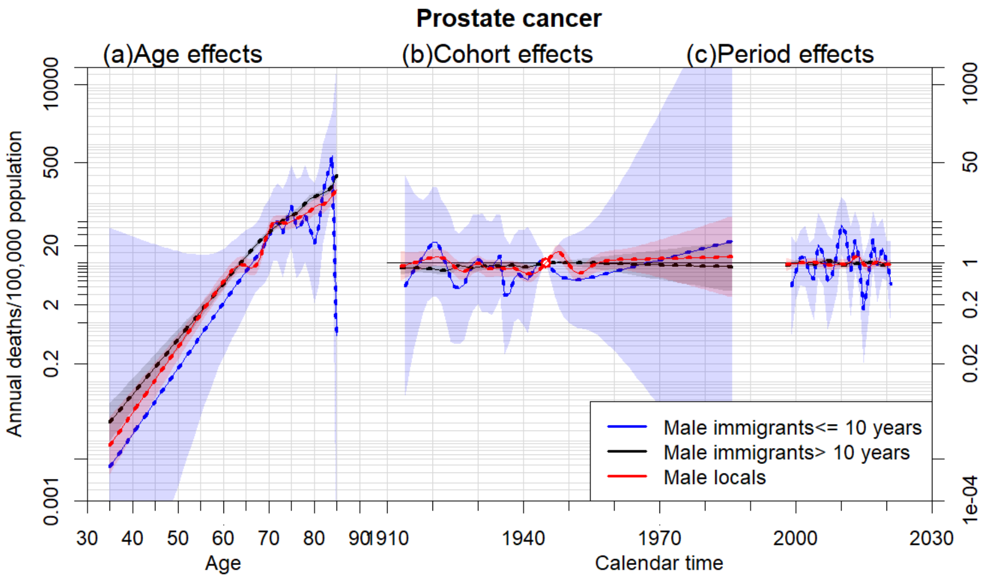

eFigure 1(e). Parameter estimates of age (a), cohort (b) and period (c) effects based on an age-period-cohort model of male prostate cancer mortality rates by immigrant groups: locals, immigrants stay in Hong Kong for more than 10 years and immigrants stay in Hong Kong for less than or equal to 10 years. Age effect was assessed by mortality (left axis). Cohort and period effects were assessed by relative risk (right axis), 95% confidence intervals are shown as shaded bands.

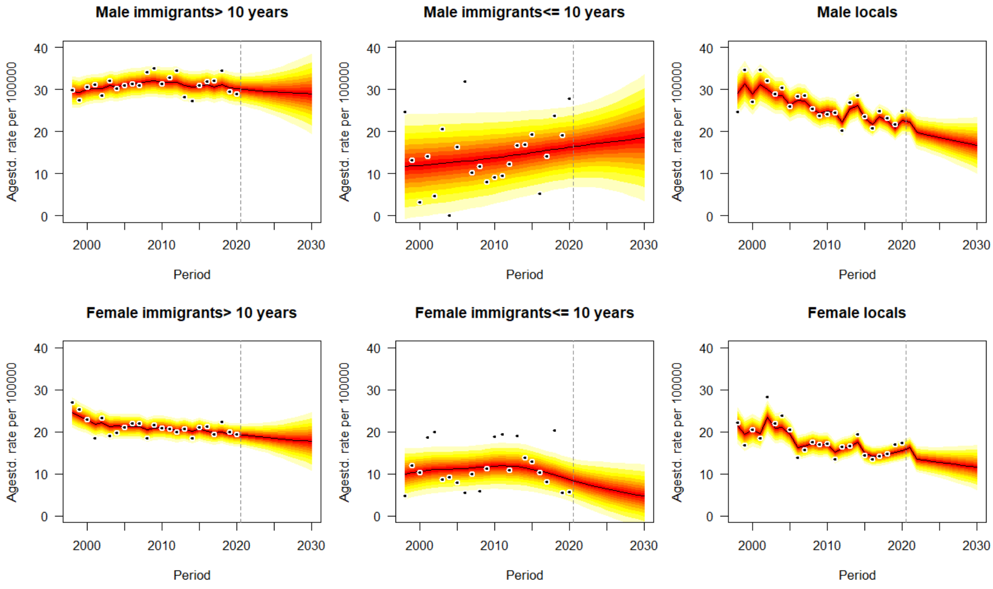

eFigure 2(a). Projections of colon cancer mortality rates by gender and immigrant status from 2022 to 2030. Observations are shown as dots with the predictive distribution between the 5% and 95% quantile, whereby each lighter shade of red represents an additional 10% predictive CI. The predictive mean is shown as black solid line and the vertical dashed line indicates where prediction started.

Zhao Y*, et al. BMJ Open* 2023; 13:e072751. doi: 10.1136/bmjopen-2023-072751

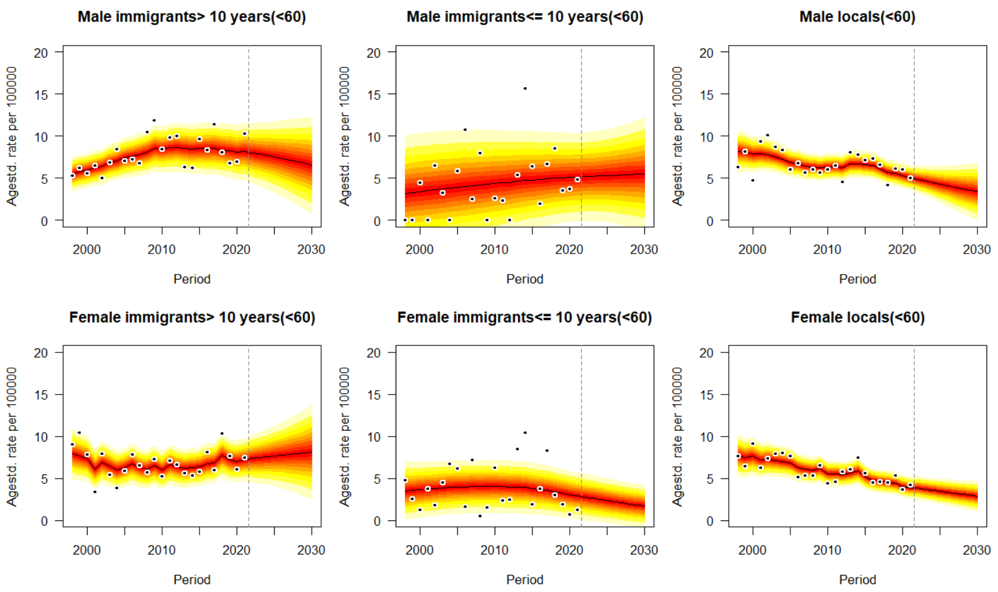

eFigure 2(b). Projections of colon cancer mortality rates for the population younger than 60 by gender and immigrant status from 2022 to 2030. Observations are shown as dots with the predictive distribution between the 5% and 95% quantile, whereby each lighter shade of red represents an additional 10% predictive CI. The predictive mean is shown as black solid line and the vertical dashed line indicates where prediction started.

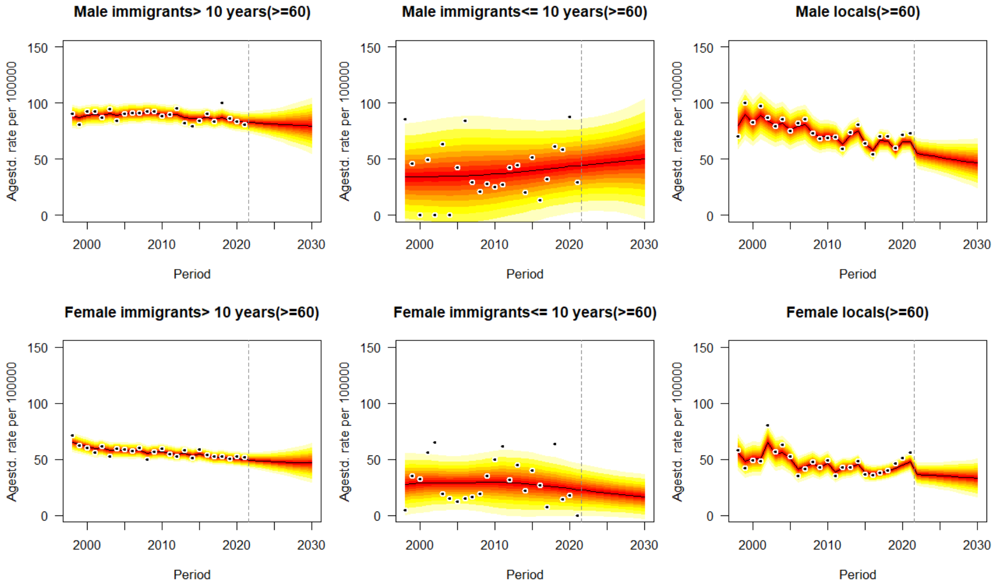

eFigure 2(c). Projections of colon cancer mortality rates for the population older than or equal to 60 by gender and immigrant status from 2022 to 2030. Observations are shown as dots with the predictive distribution between the 5% and 95% quantile, whereby each lighter shade of red represents an additional 10% predictive CI. The predictive mean is shown as black solid line and the vertical dashed line indicates where prediction started.

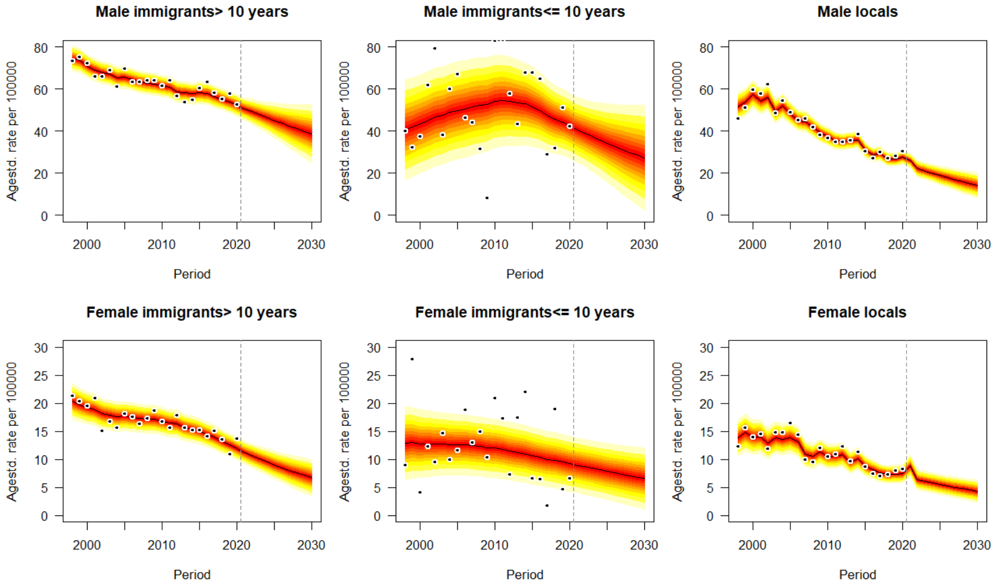

eFigure 3(a). Projections of liver cancer mortality rates by gender and immigrant status from 2022 to 2030. Observations are shown as dots with the predictive distribution between the 5% and 95% quantile, whereby each lighter shade of red represents an additional 10% predictive CI. The predictive mean is shown as black solid line and the vertical dashed line indicates where prediction started.

Zhao Y, *et al. BMJ Open* 2023; 13:e072751. doi: 10.1136/bmjopen-2023-072751

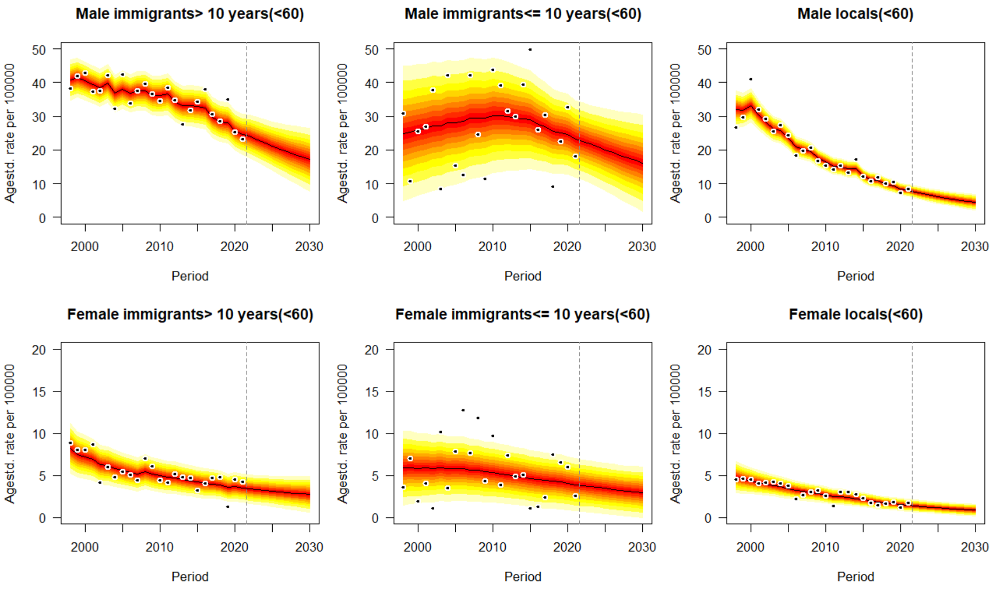

eFigure 3(b). Projections of liver cancer mortality rates for the population younger than 60 by gender and immigrant status from 2022 to 2030. Observations are shown as dots with the predictive distribution between the 5% and 95% quantile, whereby each lighter shade of red represents an additional 10% predictive CI. The predictive mean is shown as black solid line and the vertical dashed line indicates where prediction started.

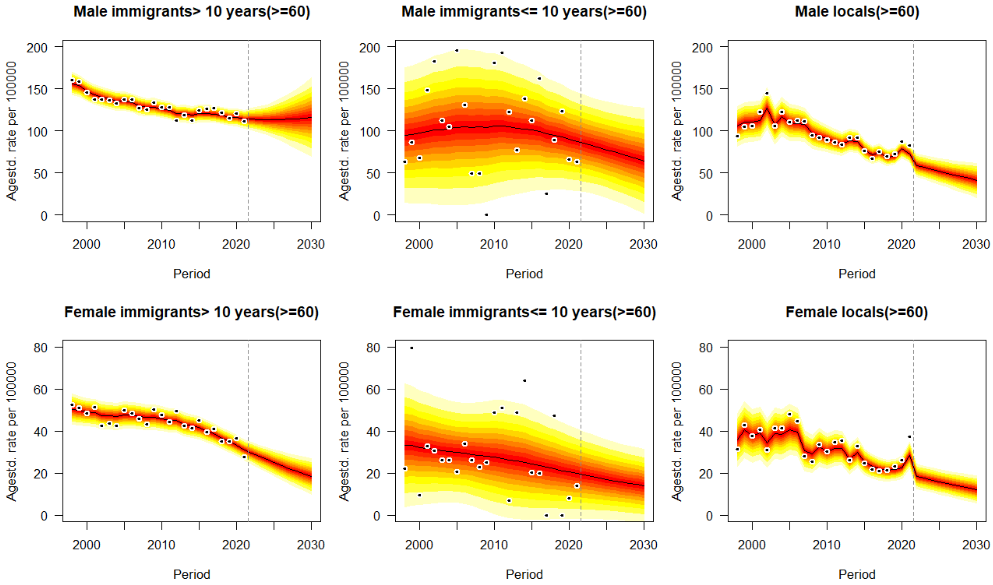

eFigure 3(c). Projections of liver cancer mortality rates for the population older than or equal to 60 by gender and immigrant status from 2022 to 2030. Observations are shown as dots with the predictive distribution between the 5% and 95% quantile, whereby each lighter shade of red represents an additional 10% predictive CI. The predictive mean is shown as black solid line and the vertical dashed line indicates where prediction started.

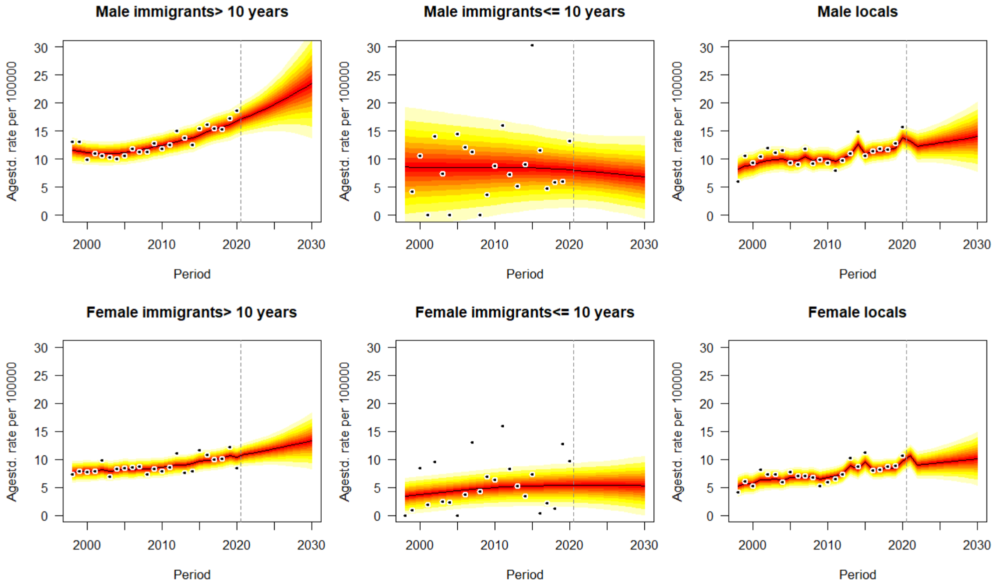

eFigure 4(a). Projections of pancreatic cancer mortality rates by gender and immigrant status from 2022 to 2030. Observations are shown as dots with the predictive distribution between the 5% and 95% quantile, whereby each lighter shade of red represents an additional 10% predictive CI. The predictive mean is shown as black solid line and the vertical dashed line indicates where prediction started.

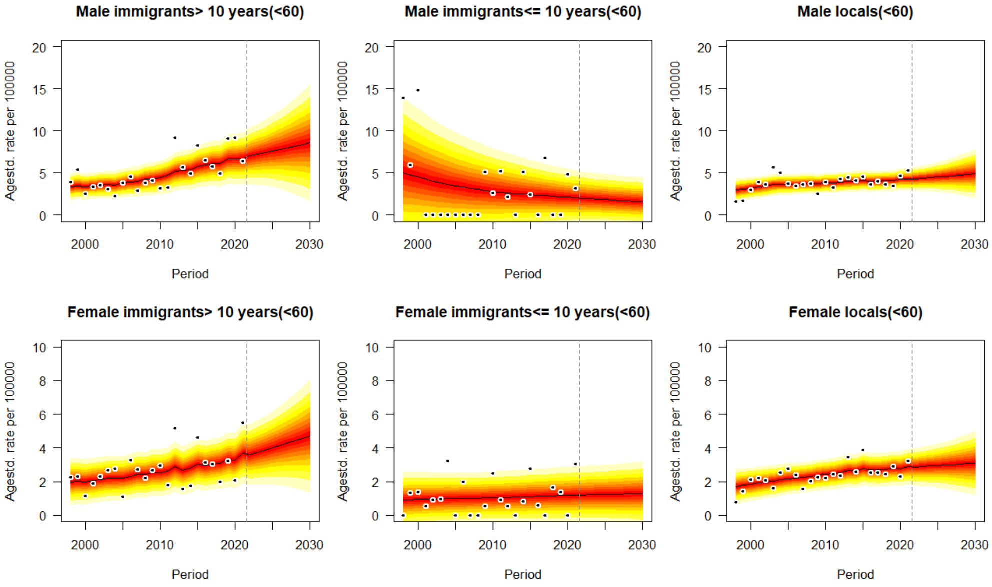

eFigure 4(b). Projections of pancreatic cancer mortality rates for the population younger than 60 by gender and immigrant status from 2022 to 2030. Observations are shown as dots with the predictive distribution between the 5% and 95% quantile, whereby each lighter shade of red represents an additional 10% predictive CI. The predictive mean is shown as black solid line and the vertical dashed line indicates where prediction started.

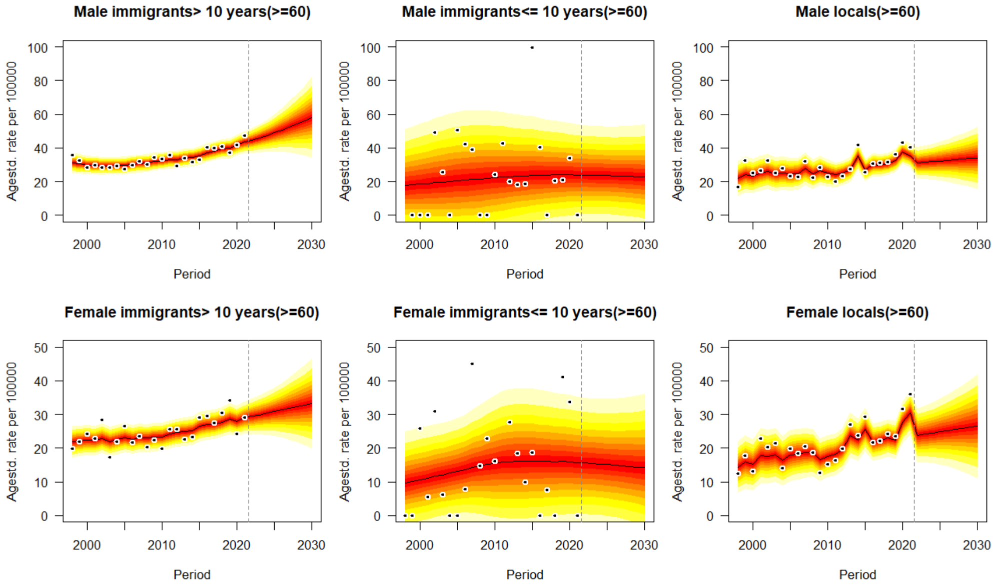

eFigure 4(c). Projections of pancreatic cancer mortality rates for the population older than or equal to 60 by gender and immigrant status from 2022 to 2030. Observations are shown as dots with the predictive distribution between the 5% and 95% quantile, whereby each lighter shade of red represents an additional 10% predictive CI. The predictive mean is shown as black solid line and the vertical dashed line indicates where prediction started.

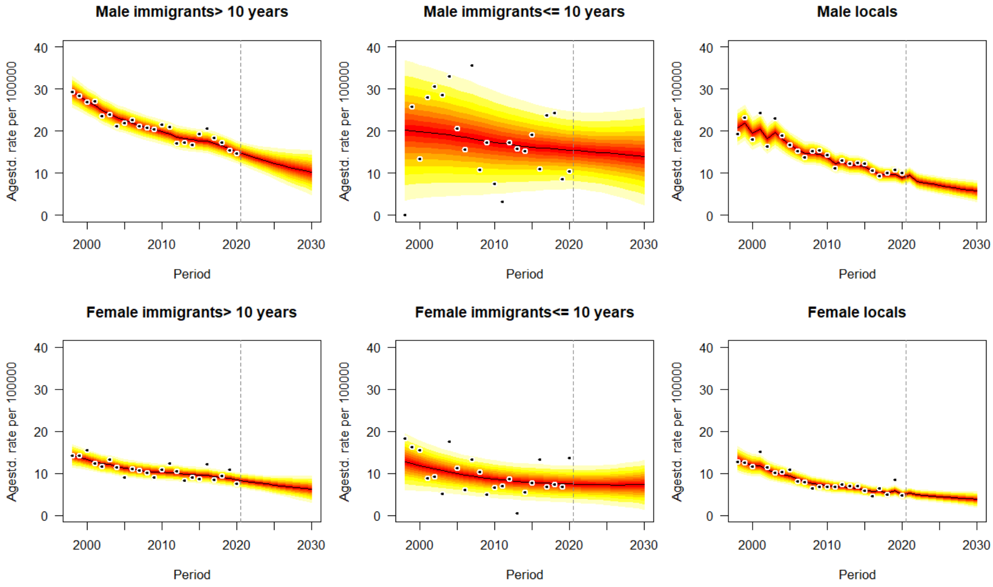

eFigure 5(a). Projections of stomach cancer mortality rates by gender and immigrant status from 2022 to 2030. Observations are shown as dots with the predictive distribution between the 5% and 95% quantile, whereby each lighter shade of red represents an additional 10% predictive CI. The predictive mean is shown as black solid line and the vertical dashed line indicates where prediction started.

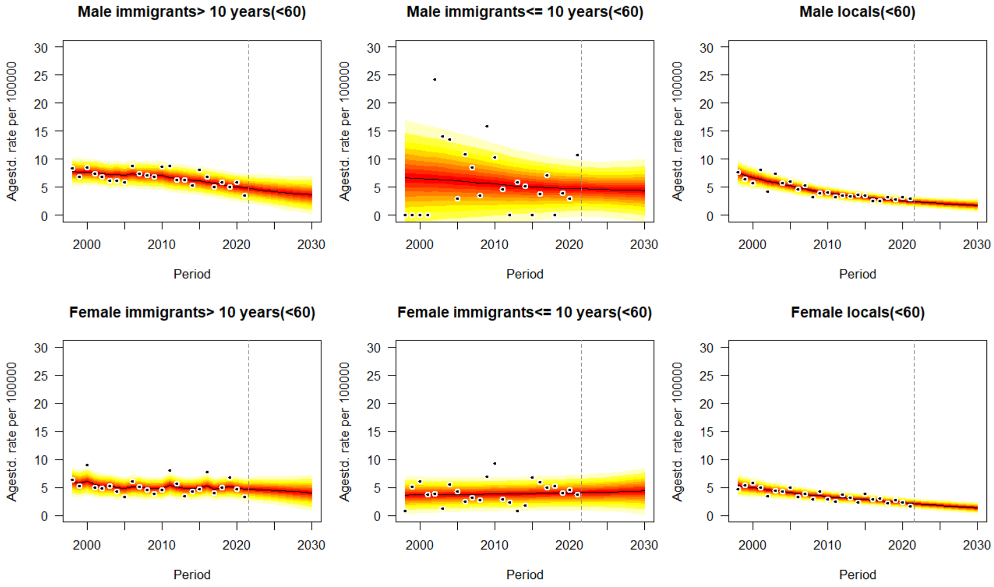

eFigure 5(b). Projections of stomach cancer mortality rates for the population younger than 60 by gender and immigrant status from 2022 to 2030. Observations are shown as dots with the predictive distribution between the 5% and 95% quantile, whereby each lighter shade of red represents an additional 10% predictive CI. The predictive mean is shown as black solid line and the vertical dashed line indicates where prediction started.

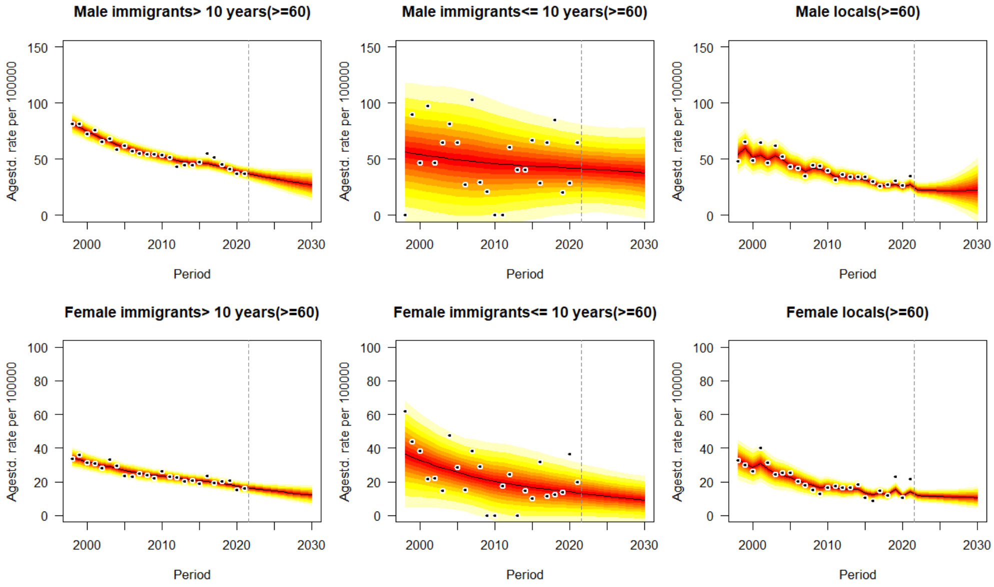

eFigure 5(c). Projections of stomach cancer mortality rates for the population older than or equal to 60 by gender and immigrant status from 2022 to 2030. Observations are shown as dots with the predictive distribution between the 5% and 95% quantile, whereby each lighter shade of red represents an additional 10% predictive CI. The predictive mean is shown as black solid line and the vertical dashed line indicates where prediction started.

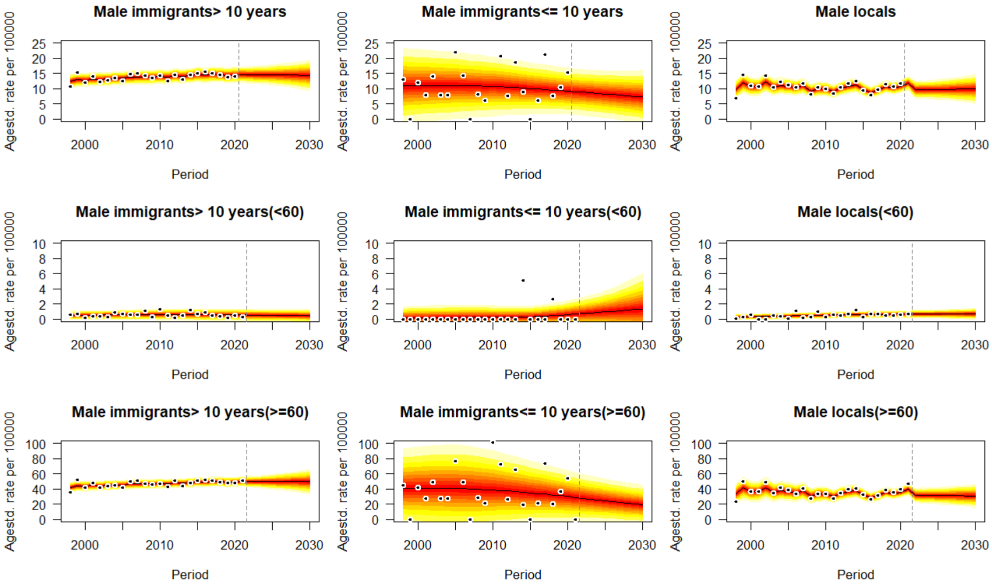

eFigure 6. Projections of prostate cancer mortality rates for males by immigrant status and age groups (less than, greater than or equal to 60 years old) from 2022 to 2030. Observations are shown as dots with the predictive distribution between the 5% and 95% quantile, whereby each lighter shade of red represents an additional 10% predictive CI. The predictive mean is shown as black solid line and the vertical dashed line indicates where prediction started.

Zhao Y, *et al. BMJ Open* 2023; 13:e072751. doi: 10.1136/bmjopen-2023-072751

Predictive mean of age-standardized mortality rates of lung cancer per 100,000 population										
Year	2021	2022	2023	2024	2025	2026	2027	2028	2029	2030
Female immigrants >10	41.80 (1.27)	41.34 (1.86)	40.58 (2.27)	39.87 (2.75)	39.19 (3.28)	38.53 (3.86)	37.89 (4.46)	37.26 (5.09)	36.65 (5.74)	36.04 (6.4)
Female immigrants ≤ 10	23.92 (4.00)	22.22 (4.67)	20.56 (5.38)	19.01 (6.10)	17.57 (6.80)	16.24 (7.45)	15.00 (8.04)	13.85 (8.56)	12.79 (9.01)	11.81 (9.39)
Female locals	34.67 (1.76)	30.22 (3.54)	30.63 (4.77)	31.05 (6.38)	31.48 (8.29)	31.9 (10.47)	32.32 (12.87)	32.73 (15.48)	33.15 (18.31)	33.55 (21.33)
Male immigrants >10	102.90 (2.43)	100.18 (4.18)	97.18 (5.33)	94.34 (6.72)	91.71 (8.24)	89.15 (9.84)	86.66 (11.47)	84.19 (13.11)	81.81 (14.74)	79.55 (16.37)
Male immigrants ≤10	81.26 (9.21)	79.90 (10.41)	79.81 (11.82)	79.72 (13.42)	79.62 (15.19)	79.50 (17.09)	79.32 (19.09)	79.08 (21.18)	78.78 (23.32)	78.41 (25.53)
Male locals	60.96 (2.82)	52.27 (4.86)	50.83 (5.39)	49.56 (6.13)	48.18 (6.97)	46.64 (7.84)	45.13 (8.76)	43.83 (9.76)	42.67 (10.8)	41.43 (11.8)
Female immigrants>10(<60y)	15.51 (1.12)	14.51 (1.50)	13.90 (1.76)	13.29 (2.04)	12.71 (2.33)	12.13 (2.62)	11.57 (2.91)	11.02 (3.18)	10.49 (3.43)	9.98 (3.68)
Female immigrants ≤ 10(<60y)	8.14 (1.91)	7.79 (1.95)	7.18(2.23)	6.62(2.53)	6.10(2.81)	5.63(3.08)	5.19(3.32)	4.79 (3.53)	4.42 (3.72)	4.09 (3.88)
Female locals(<60y)	10.25 (0.77)	9.48 (0.89)	9.17(1.02)	8.87(1.16)	8.57(1.32)	8.27(1.49)	7.97(1.65)	7.68 (1.82)	7.38 (1.98)	7.09 (2.13)
Male immigrants>10(<60y)	27.81 (2.10)	26.36 (3.58)	24.96 (3.94)	23.64 (4.35)	22.38 (4.79)	21.17 (5.23)	20.03 (5.67)	18.96 (6.10)	17.96 (6.51)	17.03 (6.90)
Male immigrants ≤ 10(<60y)	15.01 (2.98)	13.38 (3.71)	12.02 (4.17)	10.79 (4.59)	9.68 (4.95)	8.69 (5.24)	7.79 (5.46)	6.98 (5.61)	6.25 (5.69)	5.59 (5.72)
Male locals(<60y)	15.19 (0.78)	14.45 (1.15)	14.03 (1.29)	13.61 (1.46)	13.14 (1.64)	12.65 (1.82)	12.13 (2.01)	11.55 (2.17)	10.93 (2.31)	10.26 (2.43)
Female immigrants >10(≥ 60y)	108.85 (4.80)	107.21 (5.17)	106.26 (6.24)	105.52 (7.54)	104.94 (9.04)	104.51 (10.72)	104.21 (12.57)	104.07 (14.61)	104.06 (16.78)	104.16 (19.14)
Female immigrants ≤ 10(≥60y)	66.16 (13.25)	63.84 (15.72)	59.88 (17.50)	56.14 (19.31)	52.60 (21.03)	49.27 (22.66)	46.14 (24.16)	43.20 (25.52)	40.44 (26.74)	37.85 (27.81)
Female locals(≥60y)	77.33 (9.40)	76.53 (10.11)	76.22 (10.85)	75.94 (11.79)	75.69 (12.94)	75.49 (14.28)	75.32 (15.80)	75.19 (17.48)	75.10 (19.33)	75.03 (21.32)
Male immigrants>10(≥60y)	293.56 (9.13)	289.8 (11.7)	286.6 (15.19)	284.28 (19.51)	282.78 (24.49)	281.99 (30.07)	281.88 (36.31)	282.31 (43.15)	283.37 (50.66)	285.03 (58.86)
Male immigrants ≤ 10(≥60y)	244.88 (30.29)	247.01 (36.85)	251.24 (42.94)	255.62 (50.06)	260.14 (58.14)	264.82 (67.14)	269.61 (77.01)	274.52 (87.75)	279.55 (99.34)	284.69 (111.81)
Male locals(≥60y)	150.75 (16.22)	146.29 (18.46)	143.54 (20.58)	141.84 (23.97)	140.07 (28.24)	138.14 (33.39)	136.65 (39.82)	136.49 (47.87)	137.24 (57.47)	138.26 (68.52)

eTable 1. Predictive means and standard deviations (in brackets) of age-standardized mortality rates of lung cancer per 100,000 population for each gender, age group (less than, greater or equal to 60 years old) and immigrant status from 2022 to 2030. Reported means and standard deviations (in brackets) of age-standardized mortality rates in 2021 are also listed.

Predictive mean of age-standardized mortality rates of colon cancer per 100,000 population										
Year	2021	2022	2023	2024	2025	2026	2027	2028	2029	2030
Female immigrants >10	20.03 (0.95)	18.95 (1.13)	18.77 (1.37)	18.59 (1.66)	18.42 (1.98)	18.27 (2.33)	18.12 (2.71)	17.98 (3.11)	17.85 (3.53)	17.73 (3.96)
Female immigrants ≤ 10	8.11 (2.19)	7.70 (2.51)	7.25 (2.81)	6.82 (3.11)	6.42 (3.37)	6.03 (3.61)	5.67 (3.83)	5.33 (4.01)	5.01 (4.17)	4.71 (4.31)
Female locals	13.77 (1.30)	13.47 (1.61)	13.24 (1.72)	13.01 (1.87)	12.77 (2.04)	12.53 (2.24)	12.29 (2.46)	12.06 (2.68)	11.82 (2.92)	11.59 (3.16)
Male immigrants >10	31.22 (1.28)	29.82 (1.46)	29.66 (1.79)	29.52 (2.19)	29.41 (2.63)	29.30 (3.11)	29.21 (3.64)	29.14 (4.19)	29.06 (4.78)	28.98 (5.39)
Male immigrants ≤10	15.47 (2.14)	16.77 (3.77)	17.02 (4.18)	17.23 (4.64)	17.45 (5.14)	17.67 (5.69)	17.88 (6.27)	18.09 (6.91)	18.31 (7.56)	18.50 (8.26)
Male locals	21.28 (1.38)	19.81 (2.07)	19.39 (2.22)	18.97 (2.42)	18.57 (2.61)	18.18 (2.85)	17.81 (3.12)	17.43 (3.40)	17.06 (3.71)	16.71 (4.03)
Female immigrants >10(<60y)	7.09 (0.99)	7.36 (1.12)	7.46 (1.28)	7.56 (1.46)	7.65 (1.68)	7.74 (1.92)	7.83 (2.19)	7.92 (2.48)	8.01 (2.79)	8.09 (3.13)
Female immigrants ≤ 10(<60y)	3.11 (0.67)	2.82 (0.86)	2.65 (0.91)	2.51 (0.97)	2.36 (1.02)	2.22 (1.07)	2.08 (1.11)	1.95 (1.14)	1.83 (1.18)	1.72 (1.22)
Female locals(<60y)	4.10 (0.41)	3.87 (0.50)	3.73 (0.54)	3.61 (0.59)	3.47 (0.65)	3.34 (0.70)	3.22 (0.76)	3.11 (0.82)	2.99 (0.88)	2.88 (0.94)
Male immigrants >10(<60y)	8.29 (0.91)	7.98 (1.17)	7.85 (1.38)	7.71 (1.60)	7.54 (1.83)	7.36 (2.08)	7.17(2.32)	6.97(2.57)	6.76(2.81)	6.55(3.05)
Male immigrants ≤ 10(<60y)	5.03 (1.44)	5.18 (1.58)	5.22 (1.75)	5.26 (1.93)	5.30 (2.14)	5.34 (2.36)	5.38(2.59)	5.43(2.84)	5.47(3.11)	5.51(3.38)
Male locals(<60y)	5.14 (0.43)	4.88 (0.63)	4.66 (0.79)	4.46 (0.96)	4.26 (1.13)	4.08 (1.31)	3.91(1.48)	3.73(1.65)	3.57(1.82)	3.42(1.97)
Female immigrants >10(≥60y)	52.16 (2.59)	49.21 (2.99)	48.70 (3.56)	48.26 (4.26)	47.87 (5.05)	47.54 (5.94)	47.26 (6.90)	47.05 (7.94)	46.91 (9.06)	46.81 (10.26)
Female immigrants ≤ 10(≥60y)	24.01 (5.83)	22.44 (6.56)	21.69 (6.96)	20.95 (7.38)	20.23 (7.80)	19.52 (8.23)	18.84 (8.66)	18.17 (9.08)	17.51 (9.49)	16.86 (9.90)
Female locals(≥60y)	37.42 (5.31)	36.69 (5.74)	36.29 (6.06)	35.87 (6.46)	35.46 (6.95)	35.04 (7.5)	34.61 (8.12)	34.19 (8.79)	33.77 (9.51)	33.34 (10.27)
Male immigrants >10(≥60y)	84.17 (3.55)	82.72 (4.09)	82.16 (4.95)	81.64 (5.97)	81.19 (7.12)	80.81 (8.39)	80.47 (9.77)	80.15 (11.24)	79.85 (12.81)	79.56 (14.45)
Male immigrants ≤ 10(≥60y)	43.25 (11.07)	44.93 (13.09)	45.62 (14.52)	46.30 (16.09)	46.96 (17.80)	47.61 (19.64)	48.25 (21.62)	48.88 (23.73)	49.51 (25.97)	50.13 (28.34)
Male locals(≥60y)	55.79 (6.86)	54.89 (7.65)	53.75 (8.03)	52.63 (8.52)	51.54 (9.12)	50.47 (9.8)	49.43 (10.55)	48.42 (11.37)	47.42 (12.25)	46.44 (13.16)

eTable 2. Predictive means and standard deviations (in brackets) of age-standardized mortality rates of colon cancer per 100,000 population for each gender, age group (less than, greater or equal to 60 years old) and immigrant status from 2022 to 2030. Reported means and standard deviations (in brackets) of age-standardized mortality rates in 2021 are also listed.

Predictive mean of age-standardized mortality rates of liver cancer per 100,000 population										
Year	2021	2022	2023	2024	2025	2026	2027	2028	2029	2030
Female immigrants >10	11.34 (0.66)	10.68 (0.71)	10.09 (0.85)	9.54 (1.01)	9.01 (1.16)	8.50 (1.31)	8.02(1.45)	7.57(1.59)	7.14(1.72)	6.74(1.83)
Female immigrants ≤10	9.15 (1.55)	8.66 (1.82)	8.38 (1.95)	8.11 (2.08)	7.84 (2.22)	7.58 (2.36)	7.32(2.49)	7.07(2.63)	6.82(2.76)	6.58(2.88)
Female locals	6.72 (0.69)	6.36 (0.88)	6.08 (0.90)	5.81 (0.93)	5.53 (0.97)	5.26 (1.01)	5.01(1.06)	4.77(1.11)	4.53(1.15)	4.3(1.21)
Male immigrants >10	52.17 (1.78)	49.22 (2.36)	47.76 (2.93)	46.35 (3.59)	45.01 (4.31)	43.67 (5.05)	42.37 (5.81)	41.1(6.56)	39.89 (7.33)	38.71 (8.08)
Male immigrants ≤10	42.33 (5.87)	39.03 (6.49)	37.39 (7.47)	35.81 (8.51)	34.26 (9.58)	32.76 (10.63)	31.31 (11.65)	29.91 (12.62)	28.56 (13.54)	27.25 (14.40)
Male locals	24.22 (1.77)	22.16 (2.09)	21.02 (2.22)	19.91 (2.39)	18.85 (2.58)	17.83 (2.79)	16.85 (3.03)	15.92 (3.21)	15.03 (3.40)	14.18 (3.59)
Female immigrants >10(<60y)	3.62 (0.45)	3.39 (0.52)	3.29 (0.57)	3.20 (0.63)	3.12 (0.69)	3.04 (0.75)	2.96(0.82)	2.89(0.89)	2.82(0.96)	2.75(1.03)
Female immigrants ≤ 10(<60y)	4.10 (0.79)	3.81 (0.91)	3.69 (0.96)	3.57 (1.02)	3.46 (1.08)	3.36 (1.15)	3.25(1.22)	3.15(1.29)	3.06(1.36)	2.97(1.43)
Female locals(<60y)	1.50 (0.13)	1.37 (0.2)	1.29 (0.21)	1.22 (0.23)	1.16 (0.24)	1.10 (0.26)	1.04(0.27)	0.99(0.29)	0.94(0.30)	0.89(0.31)
Male immigrants >10(<60y)	26.32 (2.11)	24.04 (2.35)	23.02 (2.63)	22.05 (2.94)	21.13 (3.27)	20.25 (3.61)	19.41 (3.95)	18.62 (4.30)	17.86 (4.64)	17.14 (4.98)
Male immigrants ≤ 10(<60y)	25.52 (2.99)	22.56 (3.96)	21.71 (4.44)	20.87 (4.94)	20.04 (5.45)	19.22 (5.95)	18.42 (6.45)	17.63 (6.91)	16.86 (7.36)	16.11 (7.78)
Male locals(<60y)	8.25 (0.69)	7.47 (0.74)	6.97 (0.79)	6.52 (0.86)	6.11 (0.93)	5.73 (1.01)	5.38(1.08)	5.04(1.15)	4.73(1.21)	4.44(1.27)
Female immigrants >10(≥ 60y)	33.67 (1.88)	29.63 (2.01)	27.99 (2.36)	26.42 (2.75)	24.92 (3.14)	23.49 (3.52)	22.13 (3.88)	20.85 (4.23)	19.64 (4.55)	18.50 (4.85)
Female immigrants ≤10(≥ 60y)	21.72 (5.11)	19.08 (5.81)	18.38 (6.14)	17.71 (6.48)	17.03 (6.83)	16.39 (7.16)	15.76 (7.49)	15.16 (7.80)	14.57 (8.11)	14.01 (8.39)
Female locals(≥60y)	20.63 (3.03)	18.41 (3.23)	17.55 (3.26)	16.72 (3.32)	15.91 (3.40)	15.11 (3.49)	14.34 (3.59)	13.59 (3.69)	12.87 (3.81)	12.17 (3.93)
Male immigrants >10(≥ 60y)	115.39 (4.54)	113.96 (5.95)	113.43 (7.65)	113.17 (9.70)	113.16 (12.04)	113.37 (14.66)	113.79 (17.56)	114.39 (20.73)	115.19 (24.18)	116.17 (27.91)
Male immigrants ≤10(≥ 60y)	88.61 (15.58)	85.14 (18.85)	82.59 (20.6)	80.02 (22.44)	77.42 (24.34)	74.83 (26.24)	72.23 (28.12)	69.64 (29.94)	67.07 (31.70)	64.52 (33.38)
Male locals(≥60y)	62.88 (5.97)	58.95 (7.91)	56.51 (8.20)	54.14 (8.61)	51.84 (9.12)	49.61 (9.70)	47.46 (10.33)	45.38 (11.01)	43.38 (11.68)	41.45 (12.36)

eTable 3. Predictive means and standard deviations (in brackets) of age-standardized mortality rates of liver cancer per 100,000 population for each gender, age group (less than, greater or equal to 60 years old) and immigrant status from 2022 to 2030. Reported means and standard deviations (in brackets) of age-standardized mortality rates in 2021 are also listed.

Predictive mean of age-standardized mortality rates of pancreatic cancer per 100,000 population										
**Year**	**2021**	**2022**	**2023**	**2024**	**2025**	**2026**	**2027**	**2028**	**2029**	**2030**
**Female immigrants >10**	10.89(0.62)	11.11(0.75)	11.36(0.91)	11.61(1.09)	11.87(1.31)	12.14(1.56)	12.42(1.84)	12.71(2.15)	13.01(2.48)	13.3(2.85)
**Female immigrants ≤ 10**	5.51(1.44)	5.44(1.56)	5.44(1.69)	5.43(1.84)	5.42(1.99)	5.41(2.15)	5.39(2.32)	5.36(2.49)	5.34(2.66)	5.31(2.84)
**Female locals**	8.79(1.10)	9.01(1.22)	9.15(1.34)	9.29(1.48)	9.43(1.64)	9.57(1.83)	9.71(2.05)	9.85(2.28)	9.99(2.54)	10.14(2.83)
**Male immigrants >10**	16.30(0.98)	17.87(1.19)	18.48(1.49)	19.11(1.87)	19.78(2.32)	20.47(2.83)	21.18(3.42)	21.92(4.07)	22.69(4.81)	23.49(5.61)
**Male immigrants ≤10**	8.10(2.02)	7.87(2.37)	7.76(2.53)	7.64(2.70)	7.51(2.87)	7.38(3.05)	7.24(3.23)	7.09(3.41)	6.95(3.58)	6.81(3.75)
**Male locals**	11.97(1.26)	12.29(1.49)	12.49(1.64)	12.69(1.83)	12.91(2.06)	13.11(2.33)	13.33(2.63)	13.55(2.97)	13.78(3.34)	14.02(3.74)
**Female immigrants >10(<60y)**	3.47(0.33)	3.62(0.57)	3.74(0.66)	3.87(0.77)	4.01(0.89)	4.14(1.02)	4.28(1.18)	4.42(1.34)	4.57(1.53)	4.72(1.73)
**Female immigrants ≤ 10(<60y)**	1.12(0.33)	1.21(0.48)	1.22(0.52)	1.23(0.56)	1.24(0.61)	1.25(0.66)	1.26(0.71)	1.26(0.77)	1.27(0.83)	1.28(0.89)
**Female locals(<60y)**	2.76(0.27)	2.88(0.36)	2.91(0.41)	2.93(0.48)	2.96(0.55)	2.99(0.63)	3.02(0.71)	3.04(0.81)	3.07(0.90)	3.10(1.01)
**Male immigrants >10(<60y)**	6.88(0.98)	7.05(1.11)	7.24(1.32)	7.43(1.56)	7.62(1.84)	7.82(2.16)	8.01(2.50)	8.21(2.88)	8.40(3.30)	8.61(3.75)
**Male immigrants ≤ 10(<60y)**	2.20(0.71)	2.01(0.85)	1.95(0.91)	1.9(0.94)	1.84(0.99)	1.79(1.04)	1.74(1.09)	1.69(1.14)	1.64(1.19)	1.60(1.24)
**Male locals(<60y)**	4.16(0.35)	4.33(0.48)	4.41(0.57)	4.46(0.68)	4.53(0.81)	4.61(0.94)	4.69(1.09)	4.77(1.26)	4.85(1.44)	4.93(1.63)
**Female immigrants >10(≥60y)**	28.58(1.83)	29.45(2.11)	29.91(2.54)	30.38(3.06)	30.85(3.66)	31.33(4.33)	31.81(5.08)	32.29(5.91)	32.78(6.79)	33.27(7.74)
**Female immigrants ≤ 10(≥60y)**	16.79(5.29)	15.65(6.08)	15.49(6.71)	15.33(7.36)	15.16(8.03)	14.97(8.73)	14.79(9.43)	14.59(10.14)	14.39(10.86)	14.19(11.58)
**Female locals(≥60y)**	22.80(4.23)	23.85(4.46)	24.21(4.81)	24.56(5.23)	24.91(5.73)	25.25(6.30)	25.58(6.95)	25.90(7.67)	26.22(8.47)	26.54(9.34)
**Male immigrants >10(≥60y)**	42.70(2.55)	44.36(3.02)	45.85(3.76)	47.41(4.69)	49.04(5.78)	50.73(7.05)	52.48(8.50)	54.28(10.13)	56.16(11.95)	58.11(13.98)
**Male immigrants ≤ 10(≥60y)**	24.68(8.21)	23.96(9.01)	23.87(9.74)	23.75(10.52)	23.61(11.33)	23.45(12.17)	23.28(13.04)	23.09(13.93)	22.89(14.83)	22.68(15.75)
**Male locals(≥60y)**	30.10(4.68)	31.17(5.22)	31.55(5.63)	31.93(6.14)	32.30(6.75)	32.66(7.45)	33.01(8.23)	33.35(9.11)	33.69(10.08)	34.03(11.12)

eTable 4. Predictive means and standard deviations (in brackets) of age-standardized mortality rates of pancreatic cancer per 100,000 population for each gender, age group (less than, greater or equal to 60 years old) and immigrant status from 2022 to 2030. Reported means and standard deviations (in brackets) of age-standardized mortality rates in 2021 are also listed.

Predictive mean of age-standardized mortality rates of stomach cancer per 100,000 population										
Year	2021	2022	2023	2024	2025	2026	2027	2028	2029	2030
Female immigrants >10	8.20 (0.55)	7.95 (0.62)	7.71 (0.74)	7.47 (0.87)	7.25 (1.01)	7.03 (1.15)	6.83 (1.29)	6.62 (1.43)	6.43 (1.57)	6.24 (1.71)
Female immigrants ≤ 10	7.51 (1.44)	7.36 (1.56)	7.33 (1.69)	7.30 (1.85)	7.28 (2.01)	7.27 (2.20)	7.27 (2.40)	7.28 (2.61)	7.31 (2.84)	7.33 (3.09)
Female locals	5.26 (0.40)	4.91 (0.52)	4.75 (0.57)	4.61 (0.63)	4.47 (0.71)	4.34 (0.77)	4.21 (0.84)	4.08 (0.91)	3.95 (0.99)	3.83 (1.06)
Male immigrants >10	15.22 (0.64)	13.89 (0.97)	13.34(1.21)	12.81 (1.46)	12.31 (1.73)	11.83 (1.99)	11.38 (2.26)	10.95 (2.51)	10.54 (2.76)	10.15 (3.01)
Male immigrants ≤10	15.83 (3.04)	15.21 (3.38)	15.07 (3.67)	14.93 (3.98)	14.79 (4.31)	14.64 (4.65)	14.51 (5.02)	14.35 (5.39)	14.19 (5.78)	14.03 (6.17)
Male locals	8.14 (0.89)	8.07 (0.99)	7.73 (1.03)	7.41(1.07)	7.10 (1.13)	6.81 (1.19)	6.51 (1.26)	6.23 (1.33)	5.97 (1.39)	5.71 (1.46)
Female immigrants >10(<60y)	4.81 (0.56)	4.69 (0.79)	4.62 (0.87)	4.55 (0.96)	4.47 (1.07)	4.39 (1.17)	4.31 (1.29)	4.22 (1.41)	4.13 (1.52)	4.03 (1.64)
Female immigrants ≤ 10(<60y)	3.89 (0.80)	4.08 (0.93)	4.10 (1.03)	4.13 (1.14)	4.17 (1.27)	4.21 (1.41)	4.24 (1.55)	4.28 (1.70)	4.32 (1.87)	4.36 (2.05)
Female locals(<60y)	2.28 (0.21)	2.08 (0.27)	1.98 (0.29)	1.88 (0.32)	1.79 (0.35)	1.71 (0.37)	1.61 (0.41)	1.53 (0.43)	1.44 (0.45)	1.37 (0.47)
Male immigrants >10(<60y)	4.94 (0.57)	4.71 (0.79)	4.55 (0.89)	4.41 (0.99)	4.25 (1.10)	4.12 (1.21)	3.98 (1.32)	3.86 (1.43)	3.74 (1.54)	3.63 (1.65)
Male immigrants ≤ 10(<60y)	4.81 (1.31)	4.70 (1.42)	4.66 (1.55)	4.63 (1.69)	4.59 (1.83)	4.55 (1.99)	4.52 (2.15)	4.48 (2.32)	4.44 (2.50)	4.41 (2.68)
Male locals(<60y)	2.48 (0.21)	2.37 (0.29)	2.28 (0.32)	2.21 (0.35)	2.12 (0.38)	2.04 (0.42)	1.97 (0.45)	1.91 (0.49)	1.83 (0.52)	1.77(0. 55)
Female immigrants >10(≥60y)	17.80 (1.04)	16.23 (1.26)	15.65 (1.47)	15.08 (1.70)	14.55 (1.94)	14.03 (2.18)	13.54 (2.43)	13.07 (2.68)	12.62 (2.92)	12.19 (3.16)
Female immigrants ≤ 10(≥60y)	14.72 (4.29)	13.01 (4.83)	12.52 (5.11)	12.03 (5.37)	11.55 (5.63)	11.08 (5.88)	10.63 (6.12)	10.19 (6.35)	9.76(6.56)	9.34 (6.75)
Female locals(≥60y)	12.20 (1.66)	11.86 (1.84)	11.67 (1.98)	11.49 (2.15)	11.33 (2.35)	11.18 (2.58)	11.04 (2.84)	10.91 (3.11)	10.79(3.4)	10.68 (3.71)
Male immigrants >10(≥ 60y)	37.23 (2.29)	36.59 (2.56)	35.17(3.18)	33.82 (3.86)	32.55 (4.57)	31.34 (5.28)	30.19 (6.01)	29.08 (6.70)	28.02 (7.40)	27.01 (8.07)
Male immigrants ≤ 10(≥60y)	42.30 (10.88)	41.43 (11.78)	41.03 (12.71)	40.61 (13.70)	40.17 (14.75)	39.71 (15.85)	39.24 (16.99)	38.75 (18.16)	38.23 (19.35)	37.71 (20.57)
Male locals(≥60y)	23.04 (3.29)	22.69 (3.56)	22.37(4.07)	22.16(4.8 4)	21.89 (5.86)	21.61 (7.22)	21.52 (9.02)	21.74 (11.29)	22.17 (14.03)	22.73 (17.28)

eTable 5. Predictive means and standard deviations (in brackets) of age-standardized mortality rates of stomach cancer per 100,000 population for each gender, age group (less than, greater or equal to 60 years old) and immigrant status from 2022 to 2030. Reported means and standard deviations (in brackets) of age-standardized mortality rates in 2021 are also listed.

Predictive mean of age-standardized mortality rates of prostate cancer per 100,000 population										
Year	2021	2022	2023	2024	2025	2026	2027	2028	2029	2030
Male immigrants >10	14.81 (0.61)	14.59 (0.79)	14.57 (0.96)	14.56 (1.15)	14.54 (1.37)	14.51 (1.61)	14.48 (1.86)	14.45 (2.13)	14.42 (2.42)	14.38 (2.72)
Male immigrants ≤10	9.03 (2.95)	8.78 (3.11)	8.58 (3.29)	8.39 (3.49)	8.19(3.69)	8.10(3.89)	7.82(4.11)	7.63(4.31)	7.45(4.51)	7.27(4.72)
Male locals	9.54 (1.40)	9.66 (1.57)	9.67 (1.66)	9.69 (1.77)	9.72(1.91)	9.75(2.06)	9.78(2.23)	9.82(2.43)	9.86(2.64)	9.9(2.88)
Male immigrants >10(<60y)	0.57 (0.12)	0.52 (0.17)	0.51 (0.19)	0.50 (0.21)	0.49(0.22)	0.48(0.24)	0.47(0.25)	0.46(0.27)	0.45(0.29)	0.44(0.31)
Male immigrants ≤ 10(<60y)	0.65 (0.59)	0.73 (0.77)	0.81 (0.93)	0.87 (1.10)	0.94(1.31)	1.01(1.51)	1.09(1.75)	1.16(2.02)	1.24(2.32)	1.33(2.64)
Male locals(<60y)	0.63 (0.12)	0.66 (0.14)	0.66 (0.16)	0.66 (0.19)	0.67(0.21)	0.67(0.24)	0.67(0.27)	0.68(0.31)	0.68(0.33)	0.69(0.37)
Male immigrants >10(≥ 60y)	49.43 (2.59)	49.61 (2.73)	49.63 (3.29)	49.64 (3.94)	49.64 (4.68)	49.64 (5.51)	49.63 (6.38)	49.62 (7.32)	49.61 (8.32)	49.58(9.3 7)
Male immigrants ≤ 10(≥60y)	28.29 (9.15)	27.66 (9.78)	26.53 (10.21)	25.4 (10.63)	24.28 (11.03)	23.16 (11.41)	22.07 (11.76)	21.01 (12.09)	19.96 (12.38)	18.95(12. 63)
Male locals(≥60y)	31.57 (5.17)	31.48 (5.49)	31.40 (5.76)	31.32 (6.09)	31.24 (6.48)	31.15 (6.94)	31.06 (7.44)	30.96 (8.01)	30.86 (8.61)	30.74(9.2 6)

eTable 6. Predictive means and standard deviations (in brackets) of age-standardized mortality rates of prostate cancer per 100,000 population for each age group (less than, greater or equal to 60 years old) and immigrant status from 2022 to 2030. Reported means and standard deviations (in brackets) of age-standardized mortality rates in 2021 are also listed.

Zhao Y, *et al. BMJ Open* 2023; 13:e072751. doi: 10.1136/bmjopen-2023-072751
