# Supplementary material for: Age-period-cohort analysis and projection of cancer mortality in Hong Kong, 1998–2030
Source: BMJ Open. 2023 Oct 11;13(10):e072751. doi: 10.1136/bmjopen-2023-072751 (PMC10583025; doi:10.1136/bmjopen-2023-072751)
Supplement: Author's manuscript [file bmjopen-2023-072751.draft_revisions.pdf]

BMJ Open

BMJ Open is committed to open peer review. As part of this commitment we make the peer review history of every article we publish publicly available.

When an article is published we post the peer reviewers' comments and the authors' responses online. We also post the versions of the paper that were used during peer review. These are the versions that the peer review comments apply to.

The versions of the paper that follow are the versions that were submitted during the peer review process. They are not the versions of record or the final published versions. They should not be cited or distributed as the published version of this manuscript.

BMJ Open is an open access journal and the full, final, typeset and author-corrected version of record of the manuscript is available on our site with no access controls, subscription charges or pay-per-view fees (http://bmjopen.bmj.com).

If you have any questions on BMJ Open's open peer review process please email info.bmjopen@bmj.com

BMJ Open

# An age-period-cohort analysis and projection of cancer mortality in Hong Kong, 1998–2030



| | |
|---|---|
| Journal: | *BMJ Open* |
| Manuscript ID | bmjopen-2023-072751 |
| Article Type: | Original research |
| Date Submitted by the Author: | 13-Feb-2023 |





| | |
|---|---|
| Complete List of Authors: | Zhao, Yanji; The Hong Kong Polytechnic University, Department of Applied Mathematics<br>Zhuang, Zian; The Hong Kong Polytechnic University, Department of Applied Mathematics; University of California Los Angeles, Department of Biostatistics<br>Yang, Lin; The Hong Kong Polytechnic University, School of Nursing<br>He, Daihai; The Hong Kong Polytechnic University, Department of Applied Mathematics; The Hong Kong Polytechnic University, Research Institute for Future Food |





| | |
|---|---|
| Keywords: | STATISTICS & RESEARCH METHODS, ONCOLOGY, Risk Factors |



SCHOLARONE™
Manuscripts

I, the Submitting Author has the right to grant and does grant on behalf of all authors of the Work (as defined in the below author licence), an exclusive licence and/or a non-exclusive licence for contributions from authors who are: i) UK Crown employees; ii) where BMJ has agreed a CC-BY licence shall apply, and/or iii) in accordance with the terms applicable for US Federal Government officers or employees acting as part of their official duties; on a worldwide, perpetual, irrevocable, royalty-free basis to BMJ Publishing Group Ltd ("BMJ") its licensees and where the relevant Journal is co-owned by BMJ to the co-owners of the Journal, to publish the Work in this journal and any other BMJ products and to exploit all rights, as set out in our licence.

The Submitting Author accepts and understands that any supply made under these terms is made by BMJ to the Submitting Author unless you are acting as an employee on behalf of your employer or a postgraduate student of an affiliated institution which is paying any applicable article publishing charge ("APC") for Open Access articles. Where the Submitting Author wishes to make the Work available on an Open Access basis (and intends to pay the relevant APC), the terms of reuse of such Open Access shall be governed by a Creative Commons licence – details of these licences and which Creative Commons licence will apply to this Work are set out in our licence referred to above.

Other than as permitted in any relevant BMJ Author's Self Archiving Policies, I confirm this Work has not been accepted for publication elsewhere, is not being considered for publication elsewhere and does not duplicate material already published. I confirm all authors consent to publication of this Work and authorise the granting of this licence.

For peer review only

# An age-period-cohort analysis and projection of cancer mortality in Hong Kong, 1998–2030

Yanji Zhao[1, #], Zian Zhuang[1,2, #], Lin Yang[3] and Daihai He [1,4*]

1 Department of Applied Mathematics, Hong Kong Polytechnic University, Hong Kong, China

2 Department of Biostatistics, University of California, Los Angeles, CA, United States

3 School of Nursing, Hong Kong Polytechnic University, Hong Kong, China

4 Research Institute for Future Food, Hong Kong Polytechnic University, Hong Kong, China

# These authors contributed equally to this work.

* To whom correspondence should be addressed:

Department of Applied Mathematics, The Hong Kong Polytechnic University, Hong Kong, China

Tel: 852-27667864; daihai.he@polyu.edu.hk (DH).

**Email of all authors**

Yanji Zhao: yan-ji.zhao@connect.polyu.hk; Zian Zhuang: zianzhuang@ucla.edu;

Yang Lin: l.yang@polyu.edu.hk; Daihai He: daihai.he@polyu.edu.hk.

*Word count:2954*



For peer review only

For peer review only - http://bmjopen.bmj.com/site/about/guidelines.xhtml

## Abstract

**Objectives:** Explore age, period, birth cohort effects and effects across different genders and immigration groups on mortality rates of lung, pancreatic, colon, liver, prostate and stomach cancers and their projections.

**Design, Setting, and Participants:** Death registry data in Hong Kong between 1998 and 2021. The population data were stratified by age, sex, immigration status, and length of stay in Hong Kong. Immigration status was classified into three groups: locals born in Hong Kong, long-stay immigrants and short-stay immigrants.

**Main Outcomes and Measures:** Age-period-cohort analysis was used to examine age, period, and birth cohort effects for genders and immigration groups from 1998 to 2021. Bayesian age-period-cohort models were applied to predict the mortality rates from 2022 to 2030.

**Results:** Short-stay immigrants indicated relatively pronounced fluctuations in age, cohort and period effects for six types of cancers, as increasing trends of age effect occurred for both genders and all immigration groups. Monotone decreasing trends or plateau of forecasting occur for both genders and all immigration groups in cancers except for increasing trends for short-stay male immigrants with colon cancer and long-stay male immigrants with pancreatic cancer. Long-stay male immigrants with lung cancer would perform the most significant decline in predictive mean from 100.18 to 79.55 deaths/100,000 population, while the same immigration group with pancreatic cancer would indicate the most significant uptrend in each year of 17.87 deaths and 23.49 deaths/100,000 population in 2022 and 2030, respectively.

**Conclusions** Immigrants had more pronounced fluctuations and sharper slopes of age, cohort, and period effects than locals. Men will be at a higher risk of mortality from cancers than women in the future, except for prostate cancer. Long-stay immigrants for each type of cancer and gender will be at a higher mortality risk than locals.


***Keywords***: Age-period-cohort analysis, immigration, mortality, lung cancer, pancreatic cancer, colon cancer, liver cancer, prostate cancer, stomach cancer



For peer review only - http://bmjopen.bmj.com/site/about/guidelines.xhtml

## Strengths and limitations of this study

- Complicated population distribution has resulted in intricate causes of cancers, while few studies have assessed the relationship between immigration status and cancer mortality. This study provides new evidence regarding the relationship between immigration status and cancer mortality.

- We could only depict trends and variations among different immigration and sex groups and insufficiently perform the estimates of the contributions of three effects or subgroups to mortality rates.

For peer review only



For peer review only - http://bmjopen.bmj.com/site/about/guidelines.xhtml

# Introduction

Several migration waves from mainland China to Hong Kong have occurred over the past century. These migration waves included a large-scale migration inflow from 1945 to 1950 (the Chinese Civil War) and a few small-scale inflows in the 1950s, 1970s, and 1990s [1-3]. In 2016, immigrants from mainland China formed approximately 38% of the population of Hong Kong. These inflows have led to a growing interest in research on the disparity of health conditions between the locals and immigrants.

Cancer has been one of most common causes of death, as an estimated 19.3 million new cancer cases and 9.9 million new cancer-associated deaths occurred worldwide in 2020 [4]. In Hong Kong, lung cancer is one of the most common causes of cancer deaths [5, 6]. Previous studies suggested that the primary cause of lung cancer is cigarette smoking [7-11]. Genetic factors, asbestos, radon gas, second-hand smoke, and other forms of air pollution have been proven to influence the risk of lung cancer [12-18]. The overall daily smoking rate in mainland China was approximately 23.2% in 2018 [21], whereas the daily smoking rate in Hong Kong was only 10.2% in 2019 [22]. The leading causes of liver cancer include viral infection, drinking of alcohol and polluted water and food supplies which are also culprits for colon, stomach and pancreatic cancer [19]. Alcohol consumption per capita in Hong Kong has reached to 2.37 liters in 2021 [23], compared to 7.0 liters of per capita consumption of alcohol in mainland China in 2018 [24]. As approximately 99% of prostate cancer cases occur after age 50, factors of prostate cancer have been regarded as old age, race, family history and the diet of red meat consumption [20]. In addition to these risk factors, studies have suggested that cancer mortality rates vary depending on migrant status [25-28]. According to data from the Census and Statistics Department of Hong Kong, approximately 81% of immigrants in Hong Kong immigrated from China mainland, Macau, and Taiwan. Immigrants from mainland China account for a bulk of this population. Previous studies have shown that child immigrants in Hong Kong tend to suffer from a higher risk of wheezing disorders and cardiovascular diseases, and immigrant women have higher age-specific mortality rates of breast cancer than locally-born women in Hong Kong [29, 30]. However, to date, few studies have investigated the effect of length of stay in Hong Kong and birthplace on the risk of other types of cancer.

In this study, we compared the mortality rates of lung, pancreatic, colon, liver, prostate and stomach cancers between locally born residents in Hong Kong and immigrants from mainland China. Both populations are widely considered as ethnically homogeneous with similar cultures. Nevertheless, due to different early life experiences, immigrants are exposed to more various social economy and lifestyle than locals. Therefore, it's constructive to ascertain whether immigrants from mainland China have a different mortality pattern of cancers with locals to



For peer review only - http://bmjopen.bmj.com/site/about/guidelines.xhtml

verify the significance of migration status for this health outcome. As Age-period-cohort (APC) analysis plays a vital role in studying time-specific phenomena in epidemiology, in this study, we developed APC models specified by sex and migrant status to assess the effects of age, period, birth cohort, and of the length of stay in Hong Kong on the mortality risks of cancers. Additionally, we predicted the mortality rates for the locally born population and immigrants in Hong Kong for the next 9 years using a predictive model, taking into account age, period, and birth cohort effects as well.

## Materials and methods

*Data*

We obtained the death registry data in Hong Kong between 1998 and 2021 from the Census and Statistics Department of Hong Kong, as the data in 2022 has not been available up to now. The population data were stratified by age, sex, immigration status, and length of stay in Hong Kong. We retrieved six types of cancer cases from the death registry data using ICD codes, such as ICD-9 code 162 and ICD-10 codes C34.0–C34.3, C348, and C349 for lung cancer. To assure comparability among registries, deaths from the age group of 35–85 years were selected, since cases younger than 35 and older than 85 were relatively trivial for lack of statistical interpretability [31]. Immigration status was classified into three groups: locals born in Hong Kong, immigrants who have lived in Hong Kong for >10 years before death defined as long-stay immigrants, and immigrants who have lived in Hong Kong for ≤10 years before death defined as short-stay immigrants. Notably, much focus was placed on immigrants from mainland China, because approximately 81% of immigrants in Hong Kong came from mainland China, Macau, and Taiwan based on the data from the Census and Statistics Department of Hong Kong. Moreover, few cases recorded from Macau and Taiwan are statistically insignificant in the analysis. Demographics and population projections from 2022 to 2030 were retrieved from the Census and Statistics Department of Hong Kong and estimated with cubic smoothing spline as the prerequisite of the predictive model. Codes for APC and BAPC analysis are available in the GitHub repository (https://github.com/kshz2164313/APC-population-projections-for-immigration-HK).

*Patient and Public Involvement*

No patient involved. Death registry data was obtained from Census and Statistics Department of Hong Kong.

*Statistical analysis*

We modeled cancer mortality rates in Hong Kong using APC analysis based on log-linear Poisson regression models. The model aimed to disentangle age, period, and cohort effects of



For peer review only - http://bmjopen.bmj.com/site/about/guidelines.xhtml

time-varying phenomena simultaneously [32, 33], given that

$$\log(E_{ij}) = \alpha_i + \beta_j + \gamma_k + \mu + \log(\theta_{ij}) \quad (1)$$

where $E_{ij}$ denotes expected mortality; $\alpha_i$, $\beta_j$, and $\gamma_k$ denote age, period, and cohort effect, respectively, for $i = 1,...,I$, $j = 1,...,J$, $k = 1,...,K$ with $k = I - i + j$. $\log(\theta_{ij})$ is the offset. We mainly focused on the contributions of sex and immigration status due to the non-identifiability problem that the effects of these three components are collinear with each other (denoted as period – age = cohort) [34]. The median dates of birth among cases were regarded as the reference cohort, while the second and penultimate period effects were constrained to the reference groups, as birth cohort effect and period effect were assessed with relative risks. For sex and immigration status, maximum likelihood framework was applied to estimate the relative risks and 95% confidence intervals (CIs) by age groups, calendar period, and birth cohort.

Several projection approaches for future cancer mortality have been developed, but a Bayesian age-period-cohort (BAPC) model built upon integrated nested Laplace approximations (INLA) [35] yields relatively higher coverage and better performance for all evaluated parameter combinations [36]. To prevent some sampling problems caused by Markov chain Monte Carlo (MCMC), this MCMC-free BAPC approach was applied to predict future cancer mortality within a fully Bayesian inference setting and provide outputs of interest simply, such as projected age-standardized and age-specific rates. Convergence checks are not necessary for this technique [35]. The projections of age-standardized cancer mortality rates for each sex and migrant status, taking into account age, period, and birth cohort effects, were performed based on the weights of population age groups from the WHO World Standard population [37], with 95% prediction intervals.

All analyses were performed via R version 4.2.1 (R Core Team, R Foundation for Statistical Computing, Vienna, Austria, 2013, http://www.R-project.org/). The APC models were established using the Epi package, and the projections based on Bayesian APC models were performed with the BAPC package.

## Results

Figure 1ab, eFigure 1-4ab and eFigure 5a in Supplement 1 illustrate the estimates of age (assessed by cancer mortality), period and cohort effects (assessed by relative risk) based on APC models among three migrant groups for men and women with six types of cancers, respectively. All the mortality rates for each gender and immigration status exhibit notable increasing trends with age. Compared to other immigration groups, age, cohort and period



For peer review only

For peer review only - http://bmjopen.bmj.com/site/about/guidelines.xhtml

effects of six types of cancer for immigrants who stayed in Hong Kong for ≤10 years revealed relatively more pronounced fluctuations and deviations from those effects in other immigration groups. Significant increasing trends of age effect occurred in all types of cancer, regardless of gender and immigration status. For example, compared to no significant effects of immigration status for women on mortality rates of lung cancer by age (Figure 1b), the higher age effect for men who have stayed in Hong Kong for >10 years occurred after the age of 50 years and the lower age effect of men who had short stays occurred before the age of 62 years (Figure 1a). In addition to compatible dynamics of period effect for locals and long-stay immigrants, similar birth cohort effects for locals and long-stay immigrants in lung, colon, liver and stomach cancers occurred before 1945, whereas significant differences of birth cohort effects between these two immigration groups occurred after 1960. Locals and long-stay immigrants in pancreatic and prostate cancer perform almost similar cohort effects all the time. Local men and women had sharper slopes of cohort effect of short stay after 1950 than other immigration groups. Short-stay immigrants who have stayed in Hong Kong for ≤10 years had more fluctuating period effects before 2020 than those for locals and long-stay immigrants. Lack of young cases, especially young short-stay immigrants, of prostate cancer leads to significant deviations and variances in age and cohort effects.

Figure 1c, eFigure 1-4c and eFigure 5b in Supplement 1 illustrates the age-standardized mortality rates of six types of cancer from 1998 to 2021 and their projections by sex and immigrant status from 2022 to 2030, taking into account age, period, and birth cohort effects. Means and standard deviations of predictive mortality rates are shown in Table 1, Table 2 and eTable 1-4 in Supplement 1. Monotone decreasing trends or plateau of forecasting occur for both genders and all immigration groups in cancers except for increasing trends for male immigrants who have stayed in Hong Kong for ≤10 years with colon cancer and male immigrants who have stayed in Hong Kong for > 10 years with pancreatic cancer. Except for prostate cancer, men will be at higher risk of mortality rates of cancers than women in the future. Given the future developing trends of other groups, the group of immigrant men who have stayed in Hong Kong for >10 years with lung, liver, pancreatic, prostate and colon cancer will have relatively higher mortality rates in each year compared with other immigration groups and pronounced decline in predictive mean. Compared with other cancers and immigration groups, male immigrants who have stayed in Hong Kong for >10 years with lung cancer would perform the most significant decline in predictive mean from 100.18 to 79.55 deaths per 100,000 population (average 2.92 deaths/100,000 per annum) (Table 1), while the same immigration group with pancreatic cancer would indicate the most significant uptrend in each year of 17.87 deaths and 23.49 deaths per 100,000 population in 2022 and 2030, respectively (average 0.62 deaths/100,000 per annum) (Table 2).



For peer review only - http://bmjopen.bmj.com/site/about/guidelines.xhtml

## Discussion

Early detection of cancer is positive and instructive for increasing chances of cure. Nevertheless, the high mortality rate of cancer results from late diagnosis among most patients after progression to more advanced or severe stages. Individuals at high risk of cancer, such as smokers, alcoholics or those who are frequently exposed to susceptible circumstances, should be screened for early detections to increase opportunities for cure [38]. Therefore, the differences in mortality rates among immigration groups are synonymous with detection means, therapies, and social history in disparate periods and areas.

Age effects for both genders and all immigrant groups revealed pronounced increasing trends on all cancer mortality rates. The group of long-stay immigrants had a higher risk of death from lung, colon and liver cancers than the other two immigration groups after the age of 60 years. Short-stay male immigrants were less likely to die from lung cancer before the age of 65 years. The contrast in age effects among the immigration groups was partially consistent with studies [25, 39] that highlighted the age effects for locals and immigrants on breast cancer mortality in Hong Kong and lung cancer incidence in Sweden, as they both showed similar trends and magnitudes between locals and immigrants before the age of 60 years. They are also compatible with the results in [40] that diagnosis of liver cancer is the most frequent among populations at 55 to 65 years old. According to these trends, young individuals, especially new young immigrant men, who have benefited from all-rounded development in mainland China and Hong Kong, are more likely to seek early detection and be treated for cancers using more advanced treatments [41]. Differences in birth cohort effects among immigrant groups partially comply with the interpretation above.

We observed significant trends of cohort effects among locals and immigrants. These findings are partially consistent but subtly different from previous findings, regarding the effect of immigration status on cancers. Zhao et al. [25] described multiple peaks of cohort effects on breast cancer mortality between locals and immigrants in Hong Kong, as well as a significant decline of cohort effects after 1950. In contrast, Sung et al. [42] investigated the difference in breast cancer incidence between Chinese Americans and non-Hispanic whites in the U.S. and emphasized that Chinese Americans were at lower risk of breast cancer than non-Hispanic whites born in the same year. Here, we interpret the cohort-driven trends resulting from the intricacy of social history and lifestyle. Compared to a relatively stable social development in Hong Kong, representing downward trends of relative risks for locals, wars and social instability in mainland China resulted in several immigration waves from mainland China to Hong Kong before 1950. Additionally, remarkable increasing trends were recorded for new



For peer review only - http://bmjopen.bmj.com/site/about/guidelines.xhtml

immigrants after 1950, which corresponded to the economic downturn after wars and famine between 1959 and 1961 during their youth [43]. The increasing trends for new immigrants and similar trends for locals and long-stay immigrants were consistent with the finding that nutrient deficiency contributes to higher risk of severe mortality rates of cancers [44]. Furthermore, we speculate that these trends, especially those for locals and long-stay immigrants, are most likely attributed to social development and personal behaviors, such as daily habits, occupational history, different diagnoses and treatments, and domestic environmental exposures. It's notable that short-stay immigrants suffered from lower risk of death from colon cancer for all ages (eFigure 2ab in Supplement 1). As locals and immigrants in Hong Kong transitioned to a more westernized lifestyles, higher consumption of meat was associated with a higher risk of these types of cancer, whereas consumptions of vegetables had a strong protective effect against pancreatic cancer, and moderate consumption of coffee appeared to be beneficial against lung cancer [45,46]. Further studies on potential risk factors are required.

Short-stay immigrants had more fluctuating and non-stationary but inconspicuous period effects before 2021 than locals and long-stay immigrants. Cumulatively, an arch pattern and fluctuating curve depicting period effects externally resulted in an arch pattern of age-standardized mortality rates for short-stay immigrant women and irregular rates for short-stay immigrant men before 2021. The external performance of different period effects on mortality rates could be most likely attributed to the higher effect of different lifestyles and social development on new immigrants than on long-stay immigrants and locals in Hong Kong. With respect to the age-standardized mortality rates and projections, consistent with previous findings [47,48], we predict that the mortality rates of cancer in Hong Kong after 2021 will continue to decline or remain relatively stable, consistent with the trends before 2020, except for male immigrants who have stayed in Hong Kong for ≤10 years with colon cancer and male immigrants who have stayed in Hong Kong for >10 years with pancreatic cancer. Men will be at higher risk of mortality rates of cancer than women, regardless of immigration status. They are also compatible with the results in [4] that males suffer from higher risk of these types of cancer except for prostate cancer than females. Furthermore, new immigrant women will be at lower risk than local women, even though long-stay immigrants will suffer from higher mortality rates than locals in the future. Potential interpretations could be consistent with those for birth cohort effects, as age and period effect are considered as confounders of cohort effect.

In the past few decades, spurred by an increasing burden of high incidence and mortality rates of cancer, several studies focused on the inherent identification dilemma of three effects in the APC model. Further, complicated population distribution and immigration status in Hong



For peer review only - http://bmjopen.bmj.com/site/about/guidelines.xhtml

Kong, one of the areas with the highest population density and migration frequency in the world, have intricate causes and inherent dynamics of cancer and other diseases. To our knowledge, few studies have assessed the relationship between immigration status and cancer mortality. Therefore, this study is original to examine the effect of the length of stay in Hong Kong and origins of previous residence on cancer deaths, which is instructive for further immigration policy making and targeted strategies of disease detection and intervention. However, this study had several limitations. Given the non-identifiability problem in age-period-cohort models, we could only depict trends and variations among different immigration and sex groups, as illustrated in figures, and insufficiently perform the estimates of the contributions of three effects or subgroups to mortality rates. Furthermore, we adopted cubic smoothing spline to estimate populations of immigrants and locals due to the large proportion of unspecified immigration status from official demographic projections.

## Conclusion

We conclude that immigrants, especially short-stay immigrants, had more pronounced fluctuations and sharper slopes of age, cohort, and period effects than locals. Men will be at a higher risk of mortality rates of six types of cancer than women in the future. Male immigrants who have stayed in Hong Kong for ≤10 years with colon cancer and male immigrants who have stayed in Hong Kong for >10 years with pancreatic cancer would perform significant uptrend in the future, while other immigration groups for each type of cancer would continue to decline or remain relatively stable. The predictive means of long-stay immigrants for each type of cancer would be greater than locals.



For peer review only - http://bmjopen.bmj.com/site/about/guidelines.xhtml

## Declaration

### Ethical approval and consent to participate

Ethical approval and consent to participate are not applicable. This study does not involve human participants. Data was obtained from the Census and Statistics Department of Hong Kong.

### Consent for publication

Not applicable.

### Availability of data and materials

Data of population in Hong Kong was retrieved from Census and Statistics Department. Codes for APC and BAPC analysis are available in the GitHub repository https://github.com/kshz2164313/APC-population-projections-for-immigration-HK

### Author contributions

**Yanji Zhao:** Methodology, Formal analysis, Data Curation, Writing - Original Draft, Visualization
**Zian Zhuang:** Methodology, Formal analysis, Data Curation, Writing - Review & Editing
**Lin Yang:** Validation, Writing - Review & Editing
**Daihai He:** Conceptualization, Writing - Review & Editing, Supervision

### Funding

The work described in this paper was partially supported by a grant from the Research Grants Council of the Hong Kong Special Administrative Region, China (HKU C7123-20G).

### Conflict of interest

None declared.

### Acknowledgements

None.

For peer review only



For peer review only - http://bmjopen.bmj.com/site/about/guidelines.xhtml

# References


1.Fan S-C. The population projection of Hong Kong. Southeast Asian Journal of Social Science. 1974;2(1/2):105-17.

2.Department CaS. Hong Kong Statistics 1947-1967 (Report). https://www.statistics.gov.hk/pub/hist/1961_1970/B10100031967AN67E0100.pdf, Accessed 4th May 2019.

3. Department CaS. Demographic Trends in Hong Kong 1981-2011 (Report). http://www.statistics.gov.hk/pub/B1120017032012XXXXB0100.pdf, Accessed 4th May 2019.

4. Sung H, Ferlay J, Siegel RL, Laversanne M, Soerjomataram I, Jemal A, et al. Global Cancer Statistics 2020: GLOBOCAN Estimates of Incidence and Mortality Worldwide for 36 Cancers in 185 Countries. CA: A Cancer Journal for Clinicians. 2021 2021/05/01;71(3):209-49. doi: https://doi.org/10.3322/caac.21660.

5. Wang XR, Chiu YL, Qiu H, Au JSK, Yu ITS. The roles of smoking and cooking emissions in lung cancer risk among Chinese women in Hong Kong. Annals of Oncology. 2009 2009/04/01/;20(4):746-51. doi: https://doi.org/10.1093/annonc/mdn699.

6. Chiu Y-L, Wang X-R, Qiu H, Yu IT-S. Risk factors for lung cancer: a case–control study in Hong Kong women. Cancer Causes & Control. 2010 2010/05/01;21(5):777-85. doi: 10.1007/s10552-010-9506-9.

7. Office on S, Health. Publications and Reports of the Surgeon General. Women and Smoking: A Report of the Surgeon General. Atlanta (GA): Centers for Disease Control and Prevention (US); 2001.

8. Escobedo LG, Peddicord JP. Smoking prevalence in US birth cohorts: the influence of gender and education. American Journal of Public Health. 1996 1996/02/01;86(2):231-6. doi: 10.2105/AJPH.86.2.231.

9. Husten CG, Shelton DM, Chrismon JH, Lin YC, Mowery P, Powell FA. Cigarette smoking and smoking cessation among older adults: United States, 1965-94. Tobacco Control. 1997;6(3):175. doi: 10.1136/tc.6.3.175.

10. Bolego C, Poli A, Paoletti R. Smoking and gender. Cardiovascular Research. 2002;53(3):568-76. doi: 10.1016/S0008-6363(01)00520-X.

11. Doll R, Hill AB. The mortality of doctors in relation to their smoking habits; a preliminary report. Br Med J. 1954;1(4877):1451-5. PMID: 13160495. doi: 10.1136/bmj.1.4877.1451.

12. Ramada Rodilla JM, Calvo Cerrada B, Serra Pujadas C, Delclos GL, Benavides FG. Fiber burden and asbestos-related diseases: an umbrella review. Gaceta Sanitaria. 2021 2021/06/11/. doi: https://doi.org/10.1016/j.gaceta.2021.04.001.

13. Collishaw NE, Kirkbride J, Wigle DT. Tobacco smoke in the workplace: an occupational health hazard. Can Med Assoc J. 1984;131(10):1199-204. PMID: 6498670.

14. Dresler CM, Fratelli C, Babb J, Everley L, Evans AA, Clapper ML. Gender differences in genetic susceptibility for lung cancer. Lung Cancer. 2000 2000/12/01/;30(3):153-60. doi: https://doi.org/10.1016/S0169-5002(00)00163-X.

15. Alexandrov K, Cascorbi I, Rojas M, Bouvier G, Kriek E, Bartsch H. CYP1A1 and GSTM1 genotypes affect benzo[ a ]pyrene DNA adducts in smokers' lung: comparison with aromatic/hydrophobic adduct formation. Carcinogenesis. 2002;23(12):1969-77. doi:




For peer review only - http://bmjopen.bmj.com/site/about/guidelines.xhtml

10.1093/carcin/23.12.1969.

16. Samet JM. Radon and Lung Cancer. JNCI: Journal of the National Cancer Institute. 1989;81(10):745-58. doi: 10.1093/jnci/81.10.745.

17. Darby S, Hill D, Auvinen A, Barros-Dios JM, Baysson H, Bochicchio F, et al. Radon in homes and risk of lung cancer: collaborative analysis of individual data from 13 European case-control studies. BMJ. 2005;330(7485):223. doi: 10.1136/bmj.38308.477650.63.

18. Raaschou-Nielsen O, Andersen ZJ, Beelen R, Samoli E, Stafoggia M, Weinmayr G, et al. Air pollution and lung cancer incidence in 17 European cohorts: prospective analyses from the European Study of Cohorts for Air Pollution Effects (ESCAPE). The Lancet Oncology. 2013 2013/08/01/;14(9):813-22. doi: https://doi.org/10.1016/S1470-2045(13)70279-1.

19. Abubakar II, Tillmann T, Banerjee A. Global, regional, and national age-sex specific all-cause and cause-specific mortality for 240 causes of death, 1990-2013: a systematic analysis for the Global Burden of Disease Study 2013. Lancet. 2015 Jan 10;385(9963):117-71.

20. Wild C. World cancer report 2014. Wild CP, Stewart BW, editors. Geneva, Switzerland: World Health Organization; 2014.

21. *2018 Summary-20190719*. Retrieved August 26, 2022, from https://www.who.int/docs/default-source/wpro---documents/countries/china/2018-gats-china-factsheet-cn-en.pdf?sfvrsn=3f4e2da9_2

22. *Thematic household survey*.Retrieved August 26, 2022, from https://www.censtatd.gov.hk/en/data/stat_report/product/B1130201/att/B11302702020XXXXB0100.pdf

23. Estimated alcohol consumption per capita in Hong Kong. Change4Health. (n.d.). Retrieved December 1, 2022, from https://www.change4health.gov.hk/en/alcohol_aware/figures/alcohol_consumption/index.html

24. World Health Organization. Global status report on alcohol and health 2018. World Health Organization; 2019 Feb 14.

25. Zhao S, Dong H, Qin J, Liu H, Li Y, Chen Y, et al. Breast cancer mortality in Chinese women: does migrant status play a role? Annals of Epidemiology. 2019 2019/12/01/;40:28-34.e2. doi: https://doi.org/10.1016/j.annepidem.2019.10.006.

26. Gomez SL, Yang J, Lin S-W, McCusker M, Sandler A, Cheng I, et al. Incidence trends of lung cancer by immigration status among Chinese Americans. Cancer Epidemiol Biomarkers Prev. 2015;24(8):1157-64. PMID: 25990553. doi: 10.1158/1055-9965.EPI-15-0123.

27.Hemminki K, Li X, Czene K. Cancer risks in first-generation immigrants to Sweden. International Journal of Cancer. 2002 2002/05/10;99(2):218-28. doi: https://doi.org/10.1002/ijc.10322.

28. Vanthomme K, Rosskamp M, De Schutter H, Vandenheede H. Lung cancer incidence differences in migrant men in Belgium, 2004–2013: histology-specific analyses. BMC Cancer. 2021 2021/03/30;21(1):328. doi: 10.1186/s12885-021-08038-6.

29. Schooling M, Leung GM, Janus ED, Ho SY, Hedley AJ, Lam TH. Childhood migration and cardiovascular risk. International Journal of Epidemiology. 2004;33(6):1219-26. doi: 10.1093/ije/dyh221.

30. Leung JYY, Li AM, Leung GM, Schooling CM. Mode of delivery and childhood hospitalizations for asthma and other wheezing disorders. Clinical & Experimental Allergy.



For peer review only - http://bmjopen.bmj.com/site/about/guidelines.xhtml

2015 2015/06/01;45(6):1109-17. doi: https://doi.org/10.1111/cea.12548.

31. Baker A, Bray I. Bayesian projections: what are the effects of excluding data from younger age groups?. American Journal of Epidemiology. 2005 Oct 15;162(8):798-805.

32. Rosenberg PS, Anderson WF. Age-Period-Cohort Models in Cancer Surveillance Research: Ready for Prime Time? APC Models. Cancer Epidemiology, Biomarkers & Prevention. 2011 Jul 1;20(7):1263-8.

33. Holford T. Analyzing the effects of age, period and cohort on incidence and mortality rates. Stat Meth Med Res. 1992;1:317-37.

34. Brookmeyer R, Stroup DF, editors. Monitoring the health of populations: statistical principles and methods for public health surveillance. Oxford University Press; 2004.

35. Riebler A, Held L. Projecting the future burden of cancer: Bayesian age–period–cohort analysis with integrated nested Laplace approximations. Biometrical Journal. 2017 May;59(3):531-49.

36. Knoll M, Furkel J, Debus J, Abdollahi A, Karch A, Stock C. An R package for an integrated evaluation of statistical approaches to cancer incidence projection. BMC medical research methodology. 2020 Dec;20(1):1-1.

37. Ahmad OB, Boschi-Pinto C, Lopez AD, Murray CJ, Lozano R, Inoue M. Age standardization of rates: a new WHO standard. Geneva: World Health Organization. 2001 Jan;9(10):1-4.

38. Sung H, Ferlay J, Siegel RL, Laversanne M, Soerjomataram I, Jemal A, Bray F. Global cancer statistics 2020: GLOBOCAN estimates of incidence and mortality worldwide for 36 cancers in 185 countries. CA: a cancer journal for clinicians. 2021 May;71(3):209-49.

39. Mousavi SM, Fallah M, Sundquist K, Hemminki K. Age-and time-dependent changes in cancer incidence among immigrants to Sweden: colorectal, lung, breast and prostate cancers. International journal of cancer. 2012 Jul 15;131(2):E122-8.

40. National Cancer Institute. SEER stat fact sheets: liver and intrahepatic bile duct cancer.

41. Wu X, Chung VC, Hui EP, Ziea ET, Ng BF, Ho RS, Tsoi KK, Wong S, Wu JC. Effectiveness of acupuncture and related therapies for palliative care of cancer: overview of systematic reviews. Scientific reports. 2015 Nov 26;5(1):1-5.

42. Sung H, Rosenberg PS, Chen WQ, Hartman M, Lim WY, Chia KS, Wai-Kong Mang O, Tse L, Anderson WF, Yang XR. The impact of breast cancer-specific birth cohort effects among younger and older C hinese populations. International journal of cancer. 2016 Aug 1;139(3):527-34.

43. The world economy volume 1: a millennial perspective, 2, Historical statistics: Academic Foundation, Gurgaon, India (2007)

44. Elias SG, Peeters PH, Grobbee DE, van Noord PA. The 1944-1945 Dutch famine and subsequent overall cancer incidence. Cancer Epidemiology Biomarkers & Prevention. 2005 Aug;14(8):1981-5.

45. Chiu YL, Wang XR, Qiu H, Yu IT. Risk factors for lung cancer: a case–control study in Hong Kong women. Cancer Causes & Control. 2010 May;21(5):777-85.

46. Li J, Lam AS, Yau ST, Yiu KK, Tsoi KK. Antihypertensive treatments and risks of lung Cancer: A large population-based cohort study in Hong Kong. BMC cancer. 2021 Dec;21(1):1-9.

47. Du J, Sun H, Sun Y, Du J, Cao W, Sun S. Assessment of age, period, and cohort effects of



For peer review only - http://bmjopen.bmj.com/site/about/guidelines.xhtml

474 lung cancer incidence in Hong Kong and projection up to 2030 based on changing demographics. American Journal of Cancer Research. 2021;11(12):5902.
476 48. *Centre for Health Protection, Department of Health - Lung Cancer*. Centre for Health Protection. Retrieved August 10, 2022, from https://www.chp.gov.hk/en/healthtopics/content/25/49.html

For peer review only



For peer review only - http://bmjopen.bmj.com/site/about/guidelines.xhtml

**Figure 1**. Parameter estimates of age, period and cohort effects based on an age-period-cohort model of males' (a) and females' (b) lung cancer mortality rates and projections from 2022 to 2030 (c) by immigrant groups: locals, immigrants stay in Hong Kong for more than 10 years and immigrants stay in Hong Kong for less than or equal to 10 years. Age effect was assessed by mortality (left axis). Cohort and period effects were assessed by relative risk (right axis), 95% confidence intervals are shown as shaded bands. Observations in (c) are shown as dots with the predictive distribution between the 5% and 95% quantile, whereby each lighter shade of blue represents an additional 10% predictive CI. The predictive mean is shown as black solid line and the vertical dashed line indicates where prediction started.

For peer review only



For peer review only - http://bmjopen.bmj.com/site/about/guidelines.xhtml

| **Predictive mean of age-standardized morality rates of lung cancer per 100,000 population** | | | | | | |
|---|---|---|---|---|---|---|
| Year | Female immigrants >10 (SD) | Female immigrants ≤10 (SD) | Female locals (SD) | Male immigrants >10 (SD) | Male immigrants ≤10 (SD) | Male locals (SD) |
| 2022 | 41.33(1.86) | 22.21(4.67) | 30.21(3.54) | 100.18(4.18) | 79.89(10.40) | 52.26(4.85) |
| 2023 | 40.58(2.26) | 20.55(5.37) | 30.63(4.77) | 97.17(5.33) | 79.81(11.82) | 50.82(5.38) |
| 2024 | 39.86(2.74) | 19.01(6.10) | 31.05(6.38) | 94.33(6.71) | 79.72(13.42) | 49.56(6.12) |
| 2025 | 39.18(3.28) | 17.57(6.80) | 31.47(8.29) | 91.69(8.24) | 79.62(15.18) | 48.17(6.96) |
| 2026 | 38.52(3.85) | 16.23(7.45) | 31.89(10.46) | 89.14(9.83) | 79.49(17.08) | 46.64(7.83) |
| 2027 | 37.88(4.46) | 15.00(8.04) | 32.31(12.86) | 86.65(11.46) | 79.32(19.09) | 45.12(8.75) |
| 2028 | 37.26(5.09) | 13.85(8.56) | 32.73(15.48) | 84.18(13.10) | 79.08(21.17) | 43.82(9.75) |
| 2029 | 36.64(5.73) | 12.79(9.01) | 33.14(18.30) | 81.80(14.73) | 78.77(23.32) | 42.66(10.79) |
| 2030 | 36.04(6.39) | 11.80(9.39) | 33.55(21.32) | 79.55(16.37) | 78.40(25.52) | 41.43(11.79) |

**Table 1.** Predictive means and standard deviations of age-standardized morality rates of lung cancer per 100,000 population for each gender and immigrant status from 2022 to 2030.

For peer review only



For peer review only - http://bmjopen.bmj.com/site/about/guidelines.xhtml

| Predictive mean of age-standardized morality rates of pancreatic cancer per 100,000 population | | | | | | |
|---|---|---|---|---|---|---|
| Year | Female immigrants >10 (SD) | Female immigrants ≤10 (SD) | Female locals (SD) | Male immigrants >10 (SD) | Male immigrants ≤10 (SD) | Male locals (SD) |
| 2022 | 11.11(0.74) | 5.437(1.55) | 9.011(1.22) | 17.87(1.18) | 7.874(2.37) | 12.28(1.48) |
| 2023 | 11.35(0.90) | 5.436(1.69) | 9.150(1.33) | 18.48(1.49) | 7.760(2.53) | 12.48(1.64) |
| 2024 | 11.61(1.09) | 5.430(1.83) | 9.289(1.47) | 19.11(1.87) | 7.639(2.70) | 12.69(1.83) |
| 2025 | 11.87(1.31) | 5.420(1.99) | 9.429(1.64) | 19.77(2.31) | 7.511(2.87) | 12.89(2.06) |
| 2026 | 12.14(1.56) | 5.405(2.15) | 9.569(1.83) | 20.46(2.83) | 7.376(3.05) | 13.11(2.32) |
| 2027 | 12.42(1.83) | 5.386(2.31) | 9.710(2.04) | 21.18(3.41) | 7.237(3.22) | 13.32(2.63) |
| 2028 | 12.70(2.14) | 5.363(2.48) | 9.851(2.28) | 21.92(4.07) | 7.094(3.40) | 13.55(2.96) |
| 2029 | 12.99(2.48) | 5.337(2.66) | 9.994(2.54) | 22.69(4.79) | 6.946(3.57) | 13.78(3.33) |
| 2030 | 13.29(2.84) | 5.307(2.84) | 10.13(2.82) | 23.49(5.60) | 6.796(3.75) | 14.02(3.74) |

**Table 2.** Predictive means and standard deviations of age-standardized morality rates of pancreatic cancer per 100,000 population for each gender and immigrant status from 2022 to 2030.

For peer review only

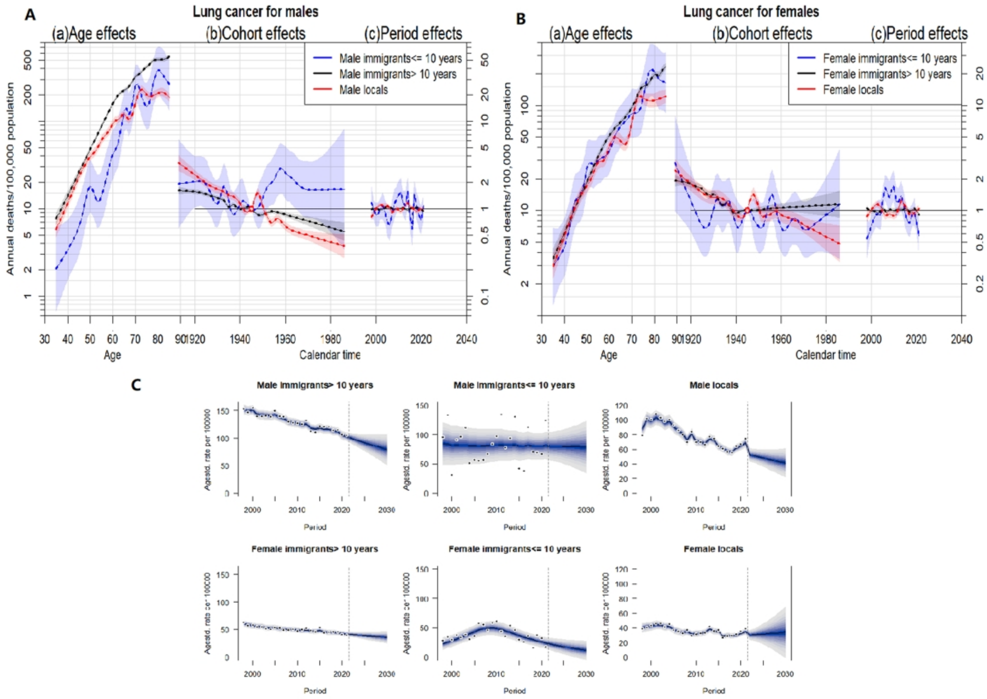


Figure 1. Parameter estimates of age, period and cohort effects based on an age-period-cohort model of males' (a) and females' (b) lung cancer mortality rates and projections from 2022 to 2030 (c) by immigrant groups: locals, immigrants stay in Hong Kong for more than 10 years and immigrants stay in Hong Kong for less than or equal to 10 years. Age effect was assessed by mortality (left axis). Cohort and period effects were assessed by relative risk (right axis), 95% confidence intervals are shown as shaded bands. Observations in (c) are shown as dots with the predictive distribution between the 5% and 95% quantile, whereby each lighter shade of blue represents an additional 10% predictive CI. The predictive mean is shown as black solid line and the vertical dashed line indicates where prediction started.

473x345mm (300 x 300 DPI)

| Predictive mean of age-standardized morality rates of colon cancer per 100,000 population | | | | | | |
|---|---|---|---|---|---|---|
| Year | Female immigrants >10 (SD) | Female immigrants ≤10 (SD) | Female locals (SD) | Male immigrants >10 (SD) | Male immigrants ≤10 (SD) | Male locals (SD) |
| 2022 | 18.95(1.12) | 7.704(2.50) | 13.46(1.59) | 29.81(1.45) | 16.77(3.76) | 19.80(2.07) |
| 2023 | 18.76(1.36) | 7.248(2.81) | 13.23(1.71) | 29.65(1.79) | 17.00(4.17) | 19.38(2.21) |
| 2024 | 18.58(1.65) | 6.819(3.10) | 13.00(1.86) | 29.51(2.18) | 17.22(4.64) | 18.97(2.39) |
| 2025 | 18.42(1.97) | 6.415(3.37) | 12.76(2.04) | 29.39(2.62) | 17.45(5.14) | 18.57(2.61) |
| 2026 | 18.26(2.33) | 6.034(3.61) | 12.53(2.24) | 29.30(3.11) | 17.66(5.68) | 18.17(2.85) |
| 2027 | 18.11(2.70) | 5.674(3.82) | 12.29(2.45) | 29.21(3.63) | 17.88(6.27) | 17.79(3.11) |
| 2028 | 17.98(3.10) | 5.334(4.01) | 12.05(2.68) | 29.13(4.19) | 18.09(6.89) | 17.42(3.40) |
| 2029 | 17.85(3.52) | 5.013(4.17) | 11.82(2.91) | 29.05(4.77) | 18.30(7.56) | 17.06(3.69) |
| 2030 | 17.72(3.96) | 4.710(4.30) | 11.58(3.16) | 28.98(5.38) | 18.50(8.25) | 16.70(4.00) |

**eTable 1.** Predictive means and standard deviations of age-standardized morality rates of colon cancer per 100,000 population for each gender and immigrant status from 2022 to 2030.

For peer review only

| Predictive mean of age-standardized morality rates of liver cancer per 100,000 population | | | | | | |
|---|---|---|---|---|---|---|
| Year | Female immigrants >10 (SD) | Female immigrants ≤10 (SD) | Female locals (SD) | Male immigrants >10 (SD) | Male immigrants ≤10 (SD) | Male locals (SD) |
| 2022 | 10.67(0.71) | 8.65(1.81) | 6.36(0.87) | 49.22(2.36) | 39.02(6.48) | 22.16(2.08) |
| 2023 | 10.09(0.85) | 8.38(1.94) | 6.07(0.90) | 47.76(2.93) | 37.38(7.46) | 21.01(2.21) |
| 2024 | 9.53(1.00) | 8.11(2.08) | 5.79(0.93) | 46.35(3.59) | 35.79(8.51) | 19.91(2.38) |
| 2025 | 9.01(1.15) | 7.84(2.21) | 5.52(0.97) | 44.99(4.30) | 34.25(9.58) | 18.85(2.58) |
| 2026 | 8.49(1.30) | 7.58(2.35) | 5.26(1.01) | 43.66(5.04) | 32.76(10.6) | 17.83(2.78) |
| 2027 | 8.02(1.45) | 7.32(2.49) | 5.01(1.06) | 42.36(5.80) | 31.31(11.6) | 16.85(2.99) |
| 2028 | 7.56(1.58) | 7.07(2.62) | 4.76(1.10) | 41.10(6.56) | 29.91(12.6) | 15.91(3.20) |
| 2029 | 7.13(1.71) | 6.82(2.75) | 4.53(1.15) | 39.88(7.32) | 28.55(13.5) | 15.02(3.40) |
| 2030 | 6.73(1.83) | 6.58(2.88) | 4.30(1.19) | 38.70(8.08) | 27.25(14.3) | 14.17(3.58) |

**eTable 2.** Predictive means and standard deviations of age-standardized morality rates of liver cancer per 100,000 population for each gender and immigrant status from 2022 to 2030.

For peer review only

| Predictive mean of age-standardized morality rates of stomach cancer per 100,000 population | | | | | | |
|---|---|---|---|---|---|---|
| Year | Female immigrants >10 (SD) | Female immigrants ≤10 (SD) | Female locals (SD) | Male immigrants >10 (SD) | Male immigrants ≤10 (SD) | Male locals (SD) |
| 2022 | 7.94(0.62) | 7.36(1.55) | 4.89(0.51) | 13.89(0.96) | 15.19(3.38) | 8.06(0.99) |
| 2023 | 7.70(0.73) | 7.32(1.69) | 4.75(0.56) | 13.33(1.20) | 15.06(3.66) | 7.73(1.02) |
| 2024 | 7.47(0.86) | 7.29(1.84) | 4.61(0.62) | 12.80(1.45) | 14.92(3.97) | 7.41(1.07) |
| 2025 | 7.24(1.00) | 7.28(2.01) | 4.47(0.69) | 12.30(1.72) | 14.78(4.30) | 7.09(1.12) |
| 2026 | 7.03(1.14) | 7.27(2.19) | 4.33(0.76) | 11.83(1.99) | 14.64(4.65) | 6.79(1.19) |
| 2027 | 6.82(1.29) | 7.27(2.39) | 4.20(0.83) | 11.38(2.25) | 14.49(5.01) | 6.50(1.25) |
| 2028 | 6.62(1.43) | 7.28(2.61) | 4.07(0.91) | 10.95(2.51) | 14.34(5.39) | 6.23(1.32) |
| 2029 | 6.42(1.57) | 7.30(2.84) | 3.95(0.98) | 10.53(2.76) | 14.18(5.77) | 5.96(1.39) |
| 2030 | 6.24(1.71) | 7.33(3.08) | 3.82(1.06) | 10.14(3.00) | 14.03(6.16) | 5.71(1.46) |

**eTable 3.** Predictive means and standard deviations of age-standardized morality rates of stomach cancer per 100,000 population for each gender and immigrant status from 2022 to 2030.

For peer review only

| Predictive mean of age-standardized morality rates of prostate cancer per 100,000 population | | | |
|---|---|---|---|
| Year | Male immigrants >10 (SD) | Male immigrants ≤10 (SD) | Male locals (SD) |
| 2022 | 14.58(0.79) | 8.77(3.10) | 9.65(1.56) |
| 2023 | 14.57(0.95) | 8.58(3.29) | 9.67(1.65) |
| 2024 | 14.55(1.14) | 8.38(3.48) | 9.69(1.76) |
| 2025 | 14.53(1.36) | 8.19(3.68) | 9.71(1.90) |
| 2026 | 14.51(1.60) | 8.00(3.89) | 9.74(2.05) |
| 2027 | 14.48(1.86) | 7.81(4.09) | 9.78(2.23) |
| 2028 | 14.45(2.13) | 7.63(4.30) | 9.81(2.43) |
| 2029 | 14.41(2.42) | 7.44(4.51) | 9.85(2.64) |
| 2030 | 14.37(2.71) | 7.27(4.72) | 9.89(2.87) |

**eTable 4.** Predictive means and standard deviations of age-standardized morality rates of prostate cancer per 100,000 population for each gender and immigrant status from 2022 to 2030.

For peer review only

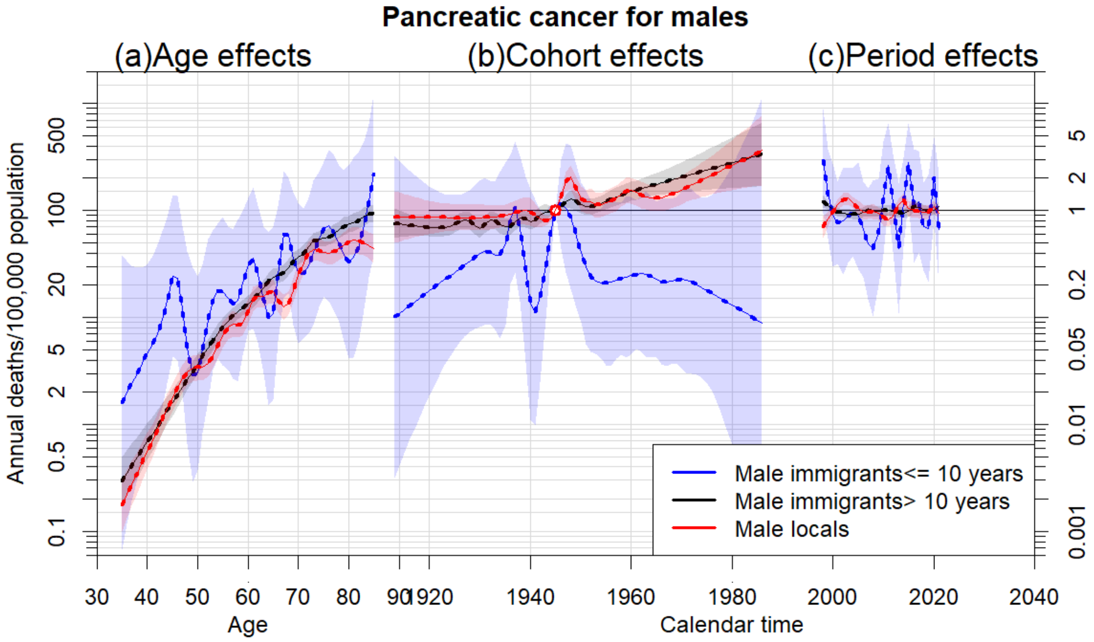


**eFigure 1(a).** Parameter estimates of age (a), period (b) and cohort (c) effects based on an age-period-cohort model of male pancreatic cancer mortality rates by immigrant groups: locals, immigrants stay in Hong Kong for more than 10 years and immigrants stay in Hong Kong for less than or equal to 10 years. Age effect was assessed by mortality (left axis). Cohort and period effects were assessed by relative risk (right axis), 95% confidence intervals are shown as shaded bands.

For peer review only

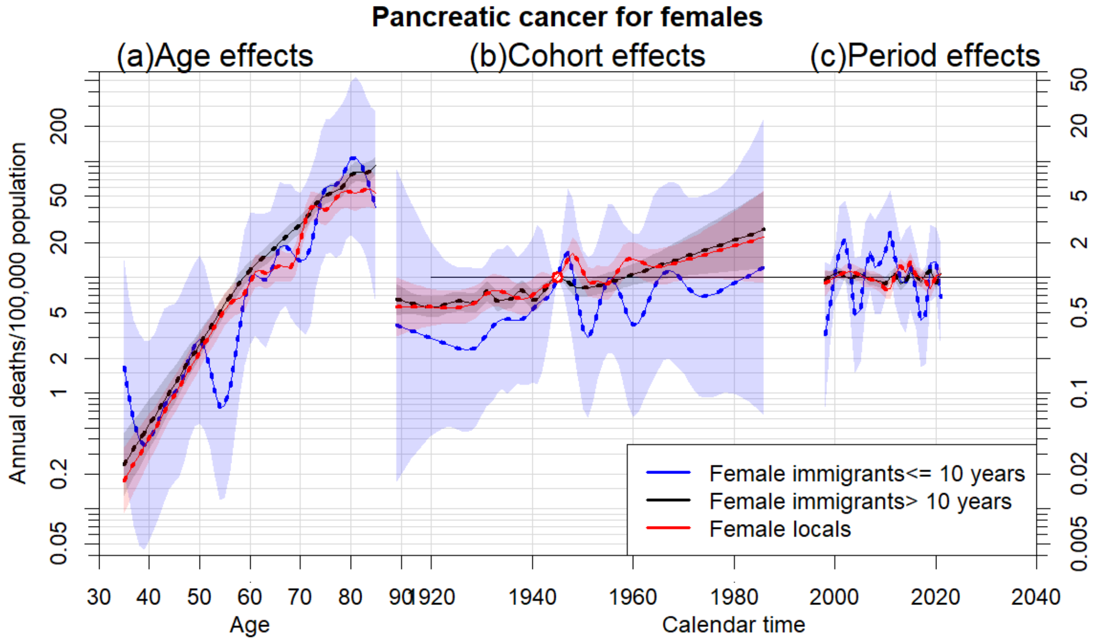


**eFigure 1(b)**. Parameter estimates of age (a), period (b) and cohort (c) effects based on an age-period-cohort model of female pancreatic cancer mortality rates by immigrant groups: locals, immigrants stay in Hong Kong for more than 10 years and immigrants stay in Hong Kong for less than or equal to 10 years. Age effect was assessed by mortality (left axis). Cohort and period effects were assessed by relative risk (right axis), 95% confidence intervals are shown as shaded bands.

For peer review only

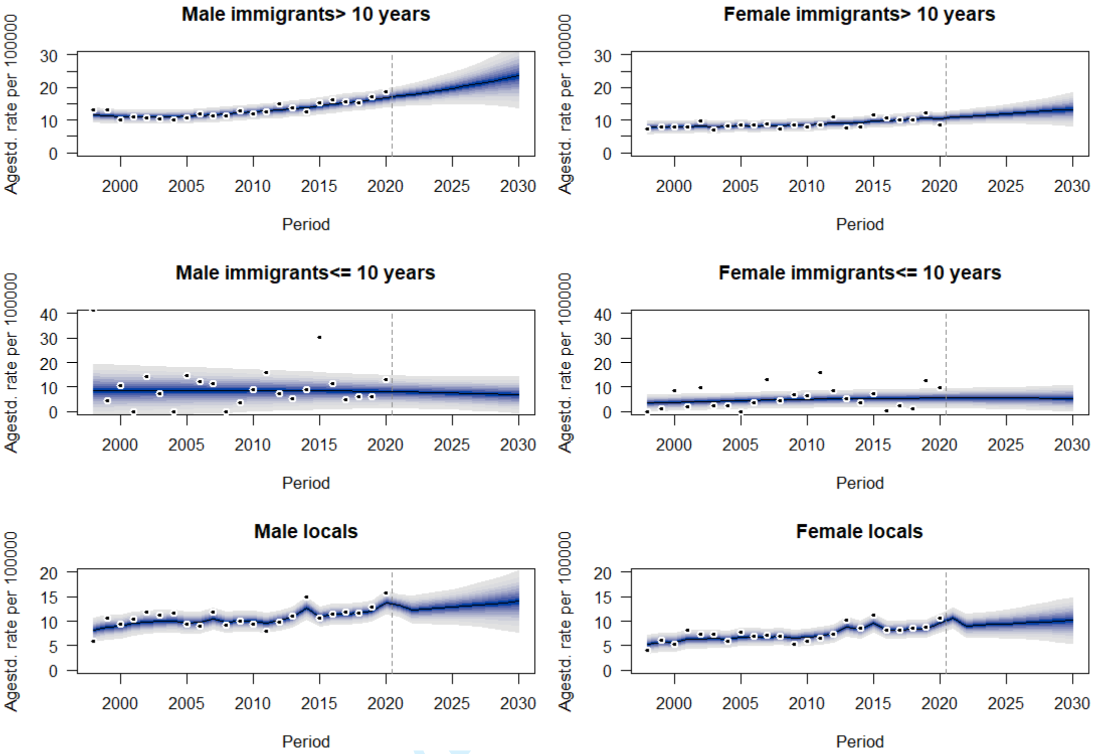


**eFigure 1(c).** Projections of pancreatic cancer mortality rates by gender and immigrant status from 2022 to 2030. Observations are shown as dots with the predictive distribution between the 5% and 95% quantile, whereby each lighter shade of blue represents an additional 10% predictive CI. The predictive mean is shown as black solid line and the vertical dashed line indicates where prediction started.

For peer review only - http://bmjopen.bmj.com/site/about/guidelines.xhtml

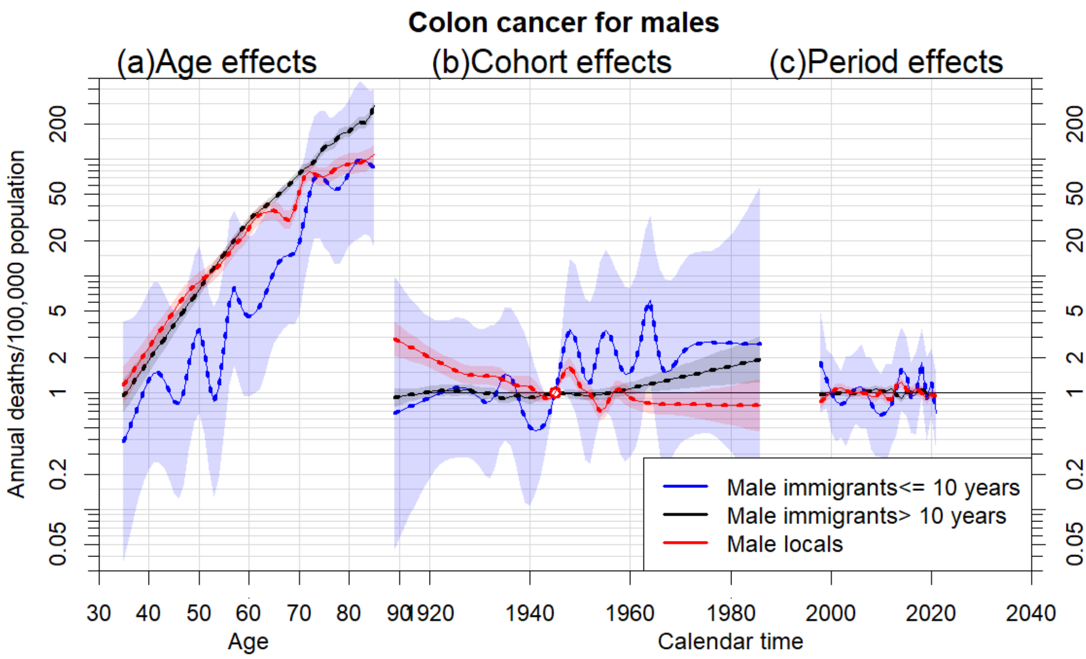


**eFigure 2(a)**. Parameter estimates of age (a), period (b) and cohort (c) effects based on an age-period-cohort model of male colon cancer mortality rates by immigrant groups: locals, immigrants stay in Hong Kong for more than 10 years and immigrants stay in Hong Kong for less than or equal to 10 years. Age effect was assessed by mortality (left axis). Cohort and period effects were assessed by relative risk (right axis), 95% confidence intervals are shown as shaded bands.

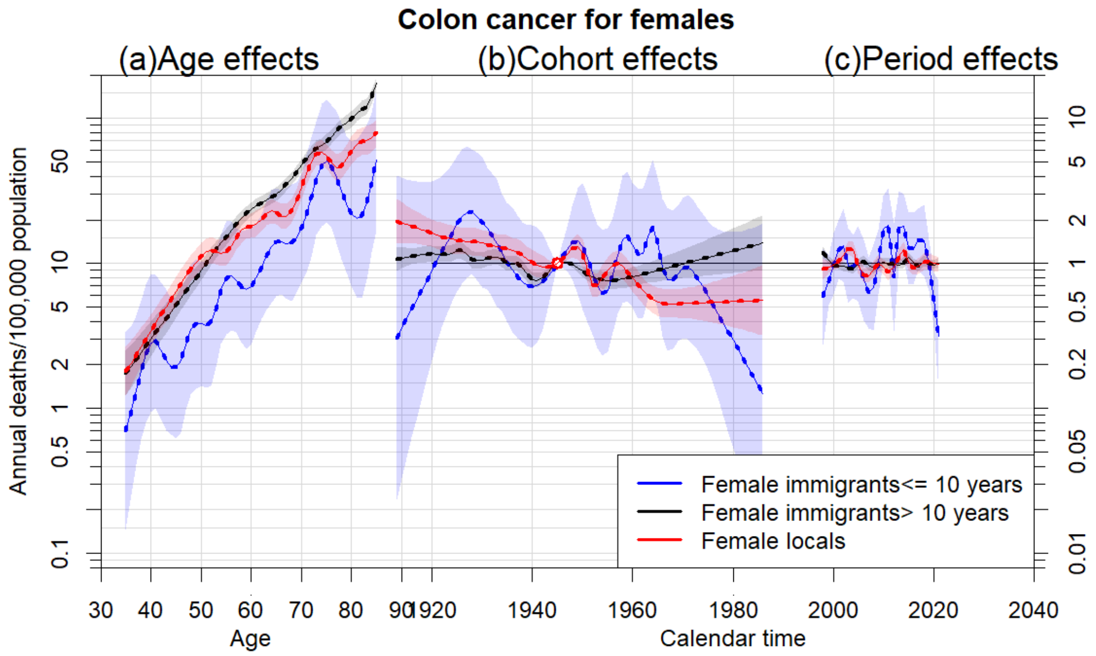


**Figure 2(b)**. Parameter estimates of age (a), period (b) and cohort (c) effects based on an age-period-cohort model of female colon cancer mortality rates by immigrant groups: locals, immigrants stay in Hong Kong for more than 10 years and immigrants stay in Hong Kong for less than or equal to 10 years. Age effect was assessed by mortality (left axis). Cohort and period effects were assessed by relative risk (right axis), 95% confidence intervals are shown as shaded bands.

For peer review only

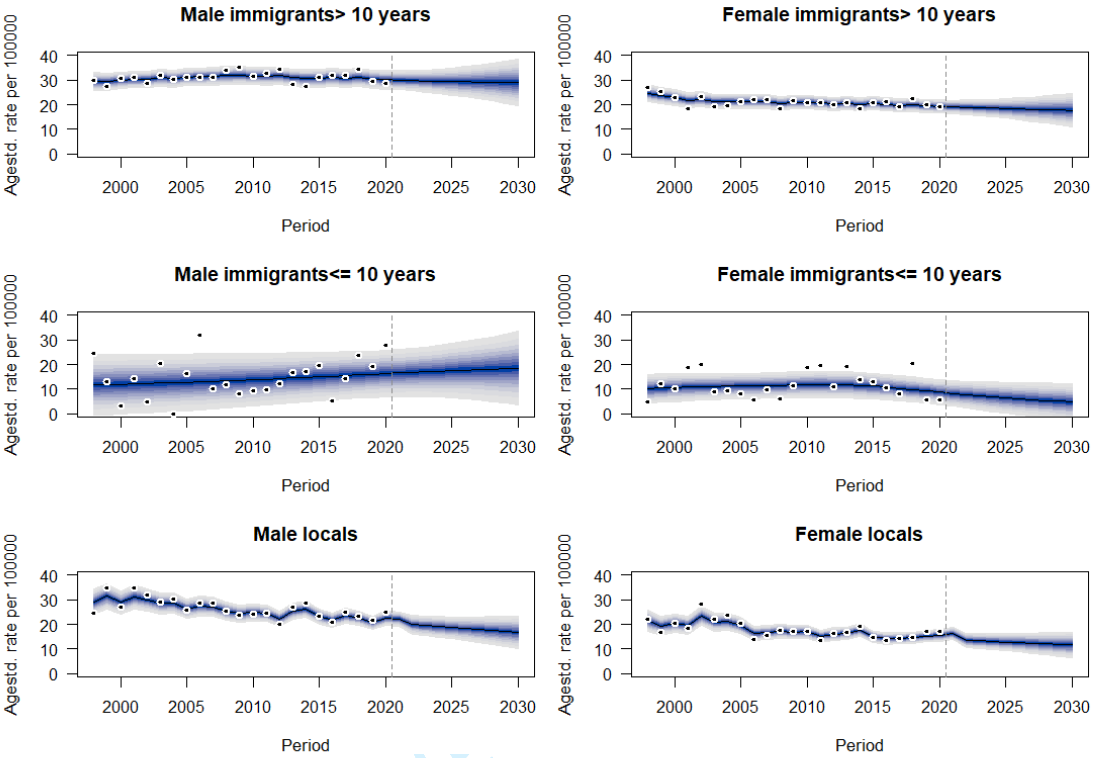


**eFigure 2(c).** Projections of colon cancer mortality rates by gender and immigrant status from 2022 to 2030. Observations are shown as dots with the predictive distribution between the 5% and 95% quantile, whereby each lighter shade of blue represents an additional 10% predictive CI. The predictive mean is shown as black solid line and the vertical dashed line indicates where prediction started.

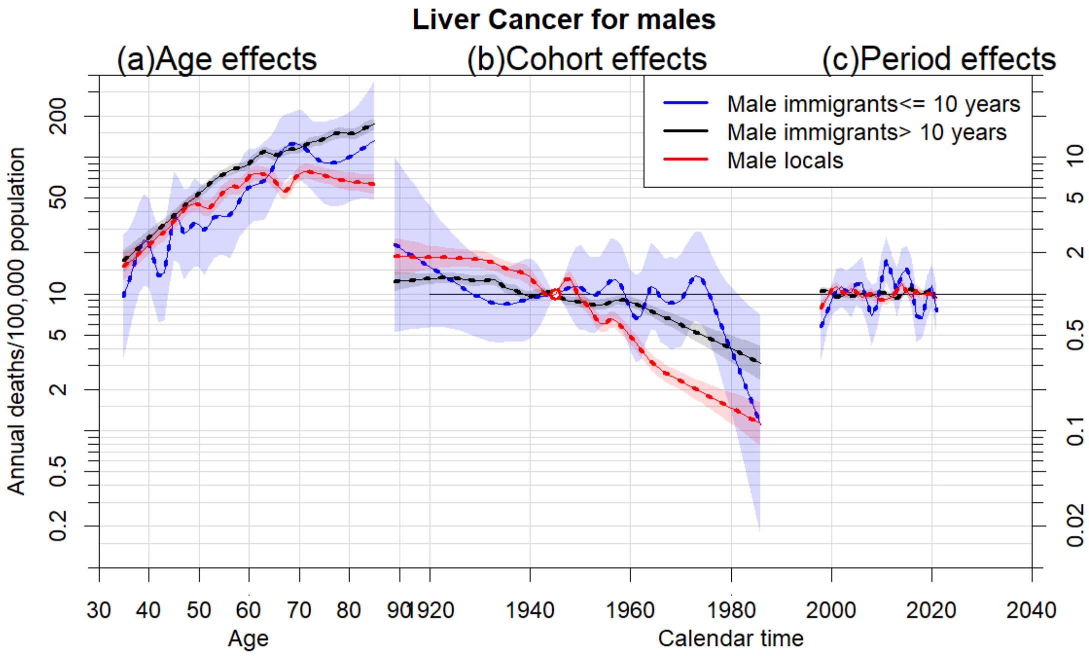


**eFigure 3(a).** Parameter estimates of age (a), period (b) and cohort (c) effects based on an age-period-cohort model of male liver cancer mortality rates by immigrant groups: locals, immigrants stay in Hong Kong for more than 10 years and immigrants stay in Hong Kong for less than or equal to 10 years. Age effect was assessed by mortality (left axis). Cohort and period effects were assessed by relative risk (right axis), 95% confidence intervals are shown as shaded bands.

For peer review only

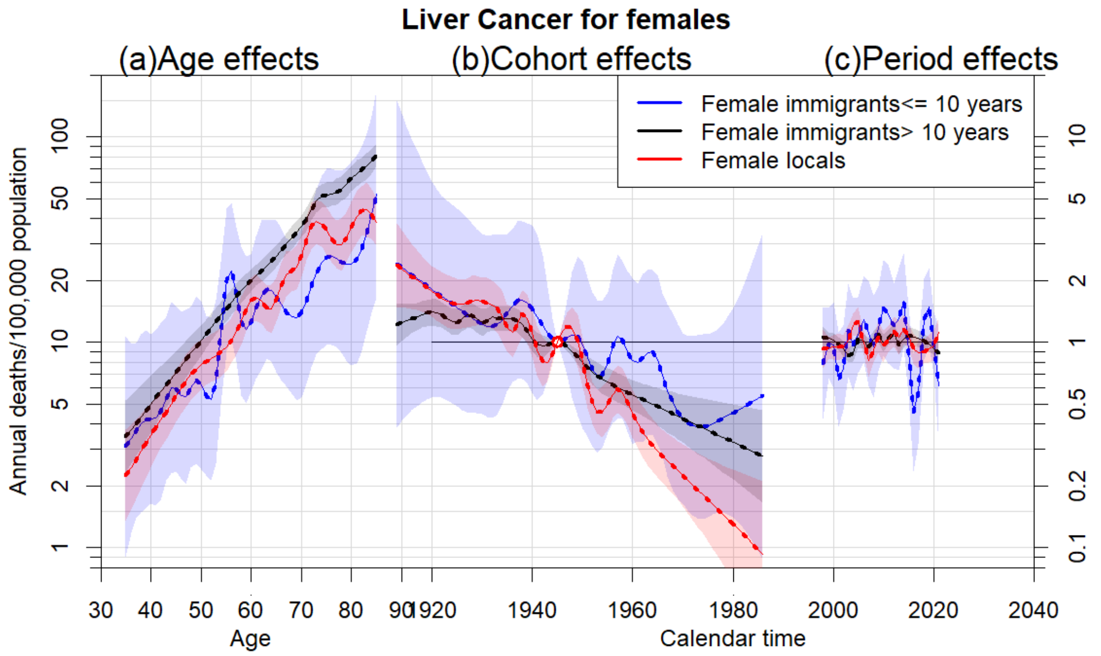


**eFigure 3(b)**. Parameter estimates of age (a), period (b) and cohort (c) effects based on an age-period-cohort model of female liver cancer mortality rates by immigrant groups: locals, immigrants stay in Hong Kong for more than 10 years and immigrants stay in Hong Kong for less than or equal to 10 years. Age effect was assessed by mortality (left axis). Cohort and period effects were assessed by relative risk (right axis), 95% confidence intervals are shown as shaded bands.

For peer review only - http://bmjopen.bmj.com/site/about/guidelines.xhtml

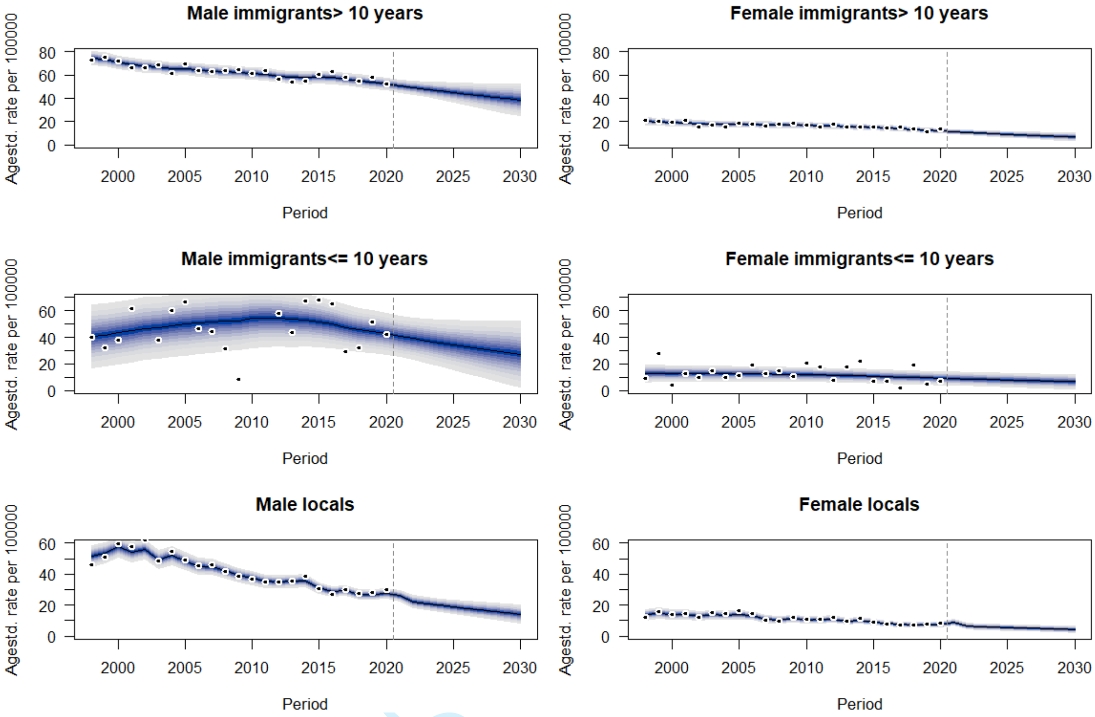


**eFigure 3(c).** Projections of liver cancer mortality rates by gender and immigrant status from 2022 to 2030. Observations are shown as dots with the predictive distribution between the 5% and 95% quantile, whereby each lighter shade of blue represents an additional 10% predictive CI. The predictive mean is shown as black solid line and the vertical dashed line indicates where prediction started.

For peer review only

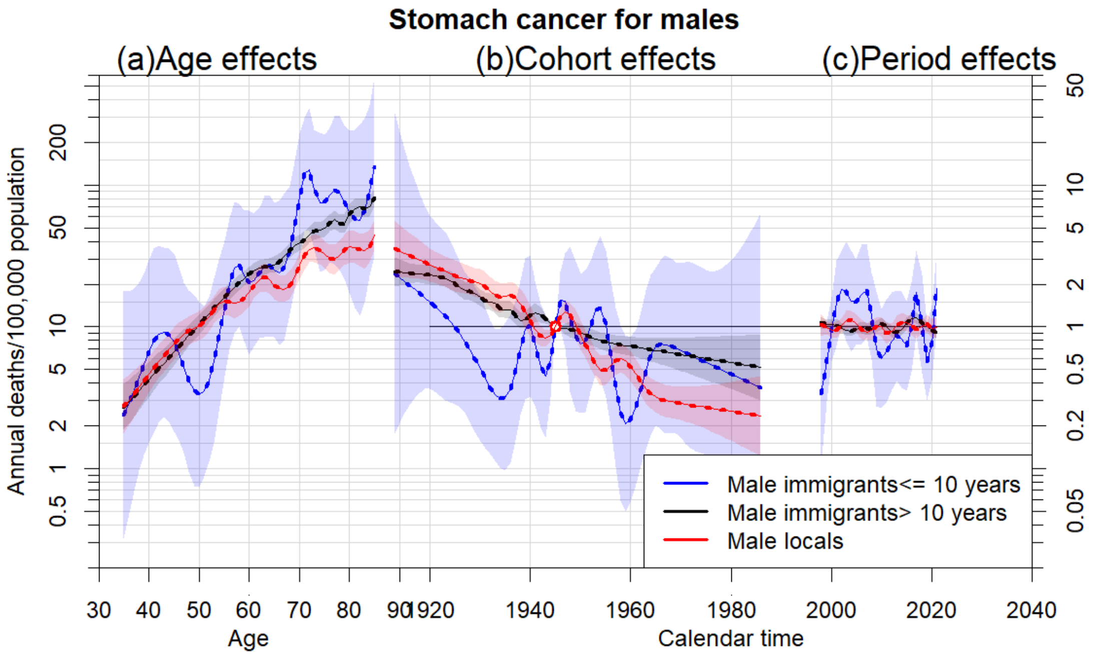


**eFigure 4(a)**. Parameter estimates of age (a), period (b) and cohort (c) effects based on an age-period-cohort model of male stomach cancer mortality rates by immigrant groups: locals, immigrants stay in Hong Kong for more than 10 years and immigrants stay in Hong Kong for less than or equal to 10 years. Age effect was assessed by mortality (left axis). Cohort and period effects were assessed by relative risk (right axis), 95% confidence intervals are shown as shaded bands.

For peer review only

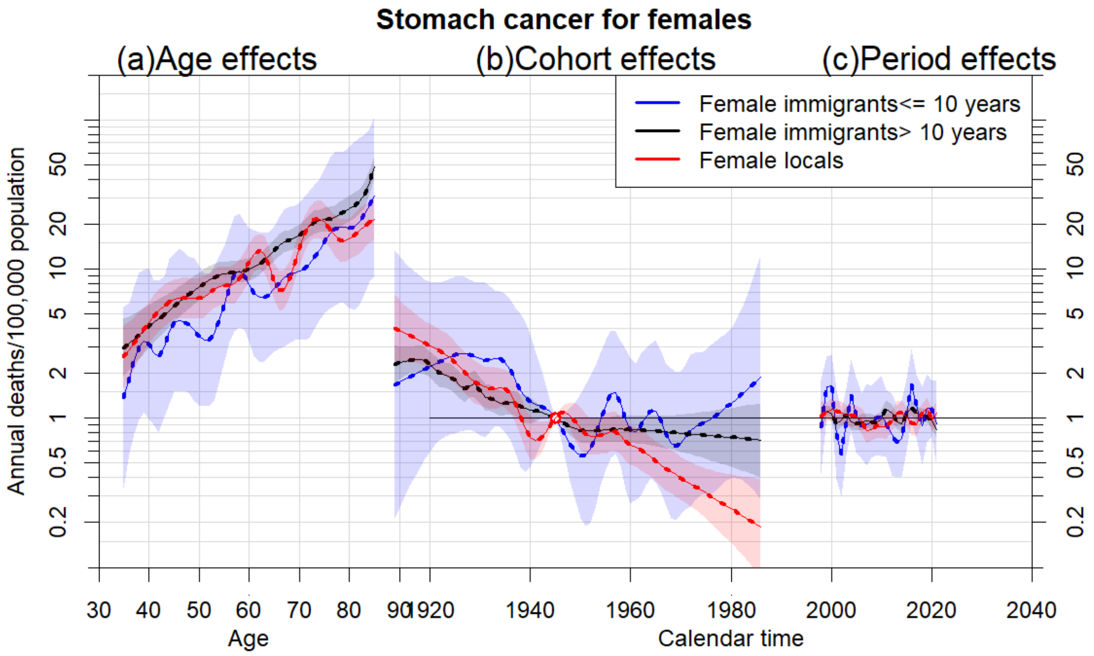


**eFigure 4(b)**. Parameter estimates of age (a), period (b) and cohort (c) effects based on an age-period-cohort model of female stomach cancer mortality rates by immigrant groups: locals, immigrants stay in Hong Kong for more than 10 years and immigrants stay in Hong Kong for less than or equal to 10 years. Age effect was assessed by mortality (left axis). Cohort and period effects were assessed by relative risk (right axis), 95% confidence intervals are shown as shaded bands.

For peer review only

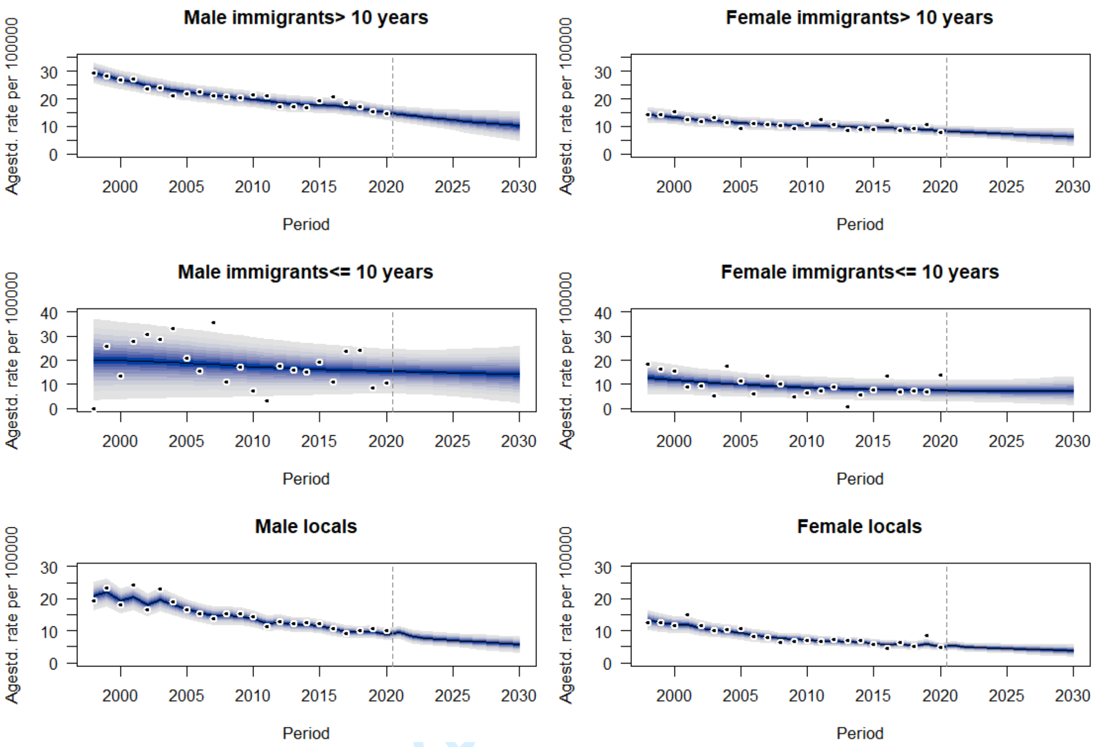


**eFigure 4(c).** Projections of stomach cancer mortality rates by gender and immigrant status from 2022 to 2030. Observations are shown as dots with the predictive distribution between the 5% and 95% quantile, whereby each lighter shade of blue represents an additional 10% predictive CI. The predictive mean is shown as black solid line and the vertical dashed line indicates where prediction started.

For peer review only

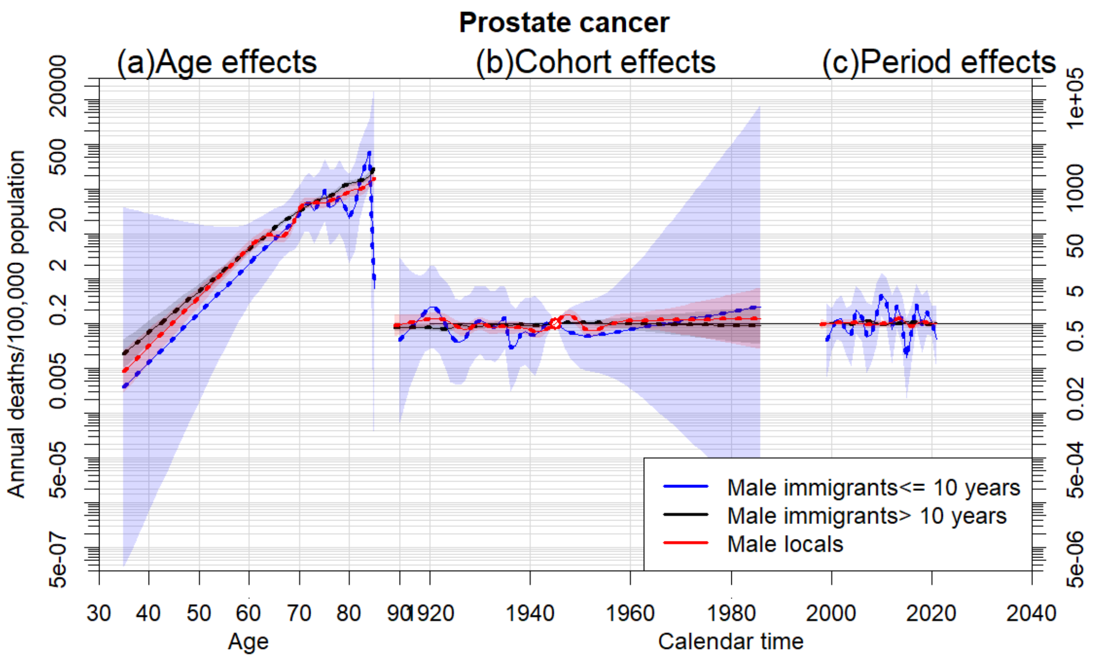


**eFigure 5(a)**. Parameter estimates of age (a), period (b) and cohort (c) effects based on an age-period-cohort model of male prostate cancer mortality rates by immigrant groups: locals, immigrants stay in Hong Kong for more than 10 years and immigrants stay in Hong Kong for less than or equal to 10 years. Age effect was assessed by mortality (left axis). Cohort and period effects were assessed by relative risk (right axis), 95% confidence intervals are shown as shaded bands.

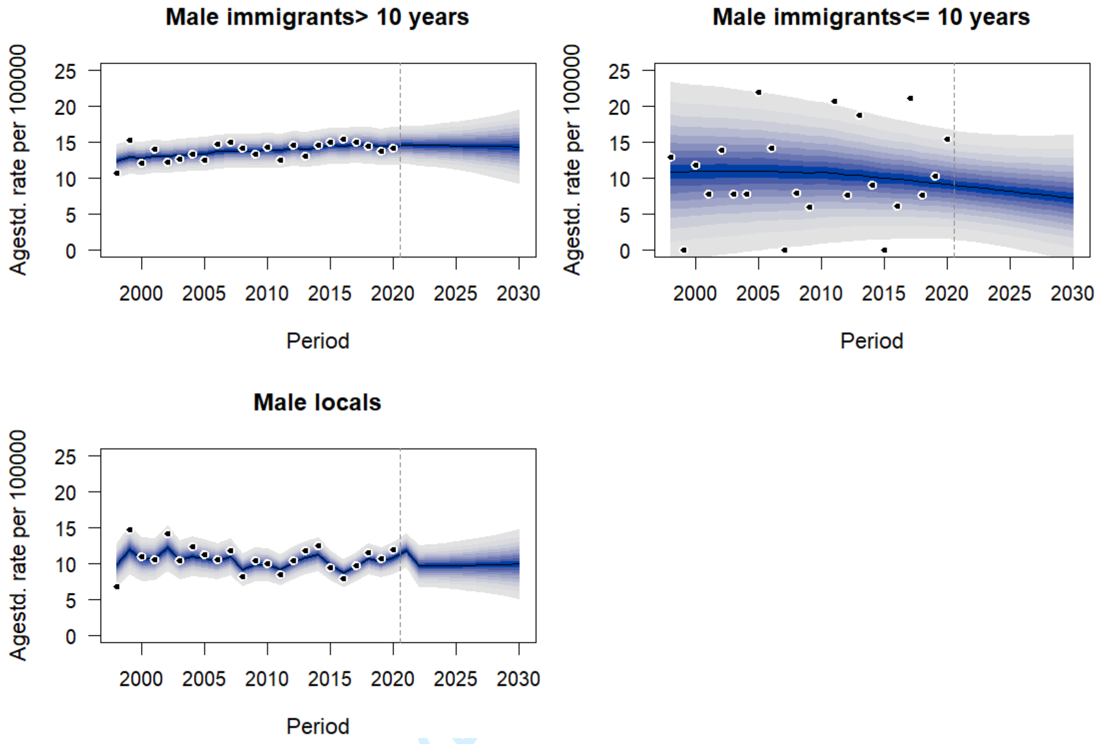


**eFigure 5(b).** Projections of prostate cancer mortality rates by immigrant status from 2022 to 2030. Observations are shown as dots with the predictive distribution between the 5% and 95% quantile, whereby each lighter shade of blue represents an additional 10% predictive CI. The predictive mean is shown as black solid line and the vertical dashed line indicates where prediction started.

For peer review only - http://bmjopen.bmj.com/site/about/guidelines.xhtml

# Reporting checklist for cohort study.

Based on the STROBE cohort guidelines.

## Instructions to authors

Complete this checklist by entering the page numbers from your manuscript where readers will find each of the items listed below.

Your article may not currently address all the items on the checklist. Please modify your text to include the missing information. If you are certain that an item does not apply, please write "n/a" and provide a short explanation.

Upload your completed checklist as an extra file when you submit to a journal.

In your methods section, say that you used the STROBE cohortreporting guidelines, and cite them as:

von Elm E, Altman DG, Egger M, Pocock SJ, Gotzsche PC, Vandenbroucke JP. The Strengthening the Reporting of Observational Studies in Epidemiology (STROBE) Statement: guidelines for reporting observational studies.

| | | Reporting Item | Page Number |
|---|---|---|---|
| **Title and abstract** | | | |
| Title | #1a | Indicate the study's design with a commonly used term in the title or the abstract | 1 |
| Abstract | #1b | Provide in the abstract an informative and balanced summary of what was done and what was found | 2 |
| **Introduction** | | | |
| Background / rationale | #2 | Explain the scientific background and rationale for the investigation being reported | 4 |
| Objectives | #3 | State specific objectives, including any prespecified hypotheses | 4 |
| **Methods** | | | |
| Study design | #4 | Present key elements of study design early in the paper | 5 |
| Setting | #5 | Describe the setting, locations, and relevant dates, including periods | 5 |

For peer review only

| | | | |
|---|---|---|---|
| | | of recruitment, exposure, follow-up, and data collection | |
| Eligibility criteria | #6a | Give the eligibility criteria, and the sources and methods of selection of participants. Describe methods of follow-up. | 5 |
| Eligibility criteria | #6b | For matched studies, give matching criteria and number of exposed and unexposed | n/a |
| Variables | #7 | Clearly define all outcomes, exposures, predictors, potential confounders, and effect modifiers. Give diagnostic criteria, if applicable | 5 |
| Data sources / measurement | #8 | For each variable of interest give sources of data and details of methods of assessment (measurement). Describe comparability of assessment methods if there is more than one group. Give information separately for for exposed and unexposed groups if applicable. | 5 |
| Bias | #9 | Describe any efforts to address potential sources of bias | 5 |
| Study size | #10 | Explain how the study size was arrived at | 5 |
| Quantitative variables | #11 | Explain how quantitative variables were handled in the analyses. If applicable, describe which groupings were chosen, and why | 5 |
| Statistical methods | #12a | Describe all statistical methods, including those used to control for confounding | |
| 5 | | | |
| Statistical methods | #12b | Describe any methods used to examine subgroups and interactions | 5 |
| Statistical methods | #12c | Explain how missing data were addressed | 5 |
| Statistical methods | #12d | If applicable, explain how loss to follow-up was addressed | n/a |
| Statistical methods | #12e | Describe any sensitivity analyses | |
| n/a | | | |
| **Results** | | | |
| Participants | #13a | Report numbers of individuals at each stage of study—eg numbers potentially eligible, examined for eligibility, confirmed eligible, | n/a |

For peer review only

| | | | |
|---|---|---|---|
| | | included in the study, completing follow-up, and analysed. Give information separately for for exposed and unexposed groups if applicable. | |
| Participants | #13b | Give reasons for non-participation at each stage | 5 |
| Participants | #13c | Consider use of a flow diagram | |
| n/a | | | |
| Descriptive data | #14a | Give characteristics of study participants (eg demographic, clinical, social) and information on exposures and potential confounders. Give information separately for exposed and unexposed groups if applicable. | 5 |
| Descriptive data | #14b | Indicate number of participants with missing data for each variable of interest | |
| n/a | | | |
| Descriptive data | #14c | Summarise follow-up time (eg, average and total amount) | |
| n/a | | | |
| Outcome data | #15 | Report numbers of outcome events or summary measures over time. Give information separately for exposed and unexposed groups if applicable. | |
| n/a | | | |
| Main results | #16a | Give unadjusted estimates and, if applicable, confounder-adjusted estimates and their precision (eg, 95% confidence interval). Make clear which confounders were adjusted for and why they were included | 6 |
| Main results | #16b | Report category boundaries when continuous variables were categorized | n/a |
| Main results | #16c | If relevant, consider translating estimates of relative risk into absolute risk for a meaningful time period | |
| n/a | | | |
| Other analyses | #17 | Report other analyses done—eg analyses of subgroups and interactions, and sensitivity analyses | 7 |

**Discussion**

For peer review only

| | | | |
|---|---|---|---|
| Key results | #18 | Summarise key results with reference to study objectives | 8 |
| Limitations | #19 | Discuss limitations of the study, taking into account sources of potential bias or imprecision. Discuss both direction and magnitude of any potential bias. | 10 |
| Interpretation | #20 | Give a cautious overall interpretation considering objectives, limitations, multiplicity of analyses, results from similar studies, and other relevant evidence. | 8 |
| Generalisability | #21 | Discuss the generalisability (external validity) of the study results | 9 |
| **Other Information** | | | |
| Funding | #22 | Give the source of funding and the role of the funders for the present study and, if applicable, for the original study on which the present article is based | 11 |

The STROBE checklist is distributed under the terms of the Creative Commons Attribution License CC-BY. This checklist was completed on 11. February 2023 using https://www.goodreports.org/, a tool made by the EQUATOR Network in collaboration with Penelope.ai

For peer review only

BMJ Open

# An age-period-cohort analysis and projection of cancer mortality in Hong Kong, 1998–2030



| | |
|---|---|
| Journal: | *BMJ Open* |
| Manuscript ID | bmjopen-2023-072751.R1 |
| Article Type: | Original research |
| Date Submitted by the Author: | 21-Jun-2023 |



| | |
|---|---|
| Complete List of Authors: | Zhao, Yanji; The Hong Kong Polytechnic University, Department of Applied Mathematics<br>Zhuang, Zian; The Hong Kong Polytechnic University, Department of Applied Mathematics; University of California Los Angeles, Department of Biostatistics<br>Yang, Lin; The Hong Kong Polytechnic University, School of Nursing<br>He, Daihai; The Hong Kong Polytechnic University, Department of Applied Mathematics; The Hong Kong Polytechnic University, Research Institute for Future Food |



| | |
|---|---|
| <b>Primary Subject Heading</b>: | Research methods |
| Secondary Subject Heading: | Public health, Oncology |
| Keywords: | STATISTICS & RESEARCH METHODS, ONCOLOGY, Risk Factors |
| | |


SCHOLARONE™
Manuscripts

*I, the Submitting Author has the right to grant and does grant on behalf of all authors of the Work (as defined in the below author licence), an exclusive licence and/or a non-exclusive licence for contributions from authors who are: i) UK Crown employees; ii) where BMJ has agreed a CC-BY licence shall apply, and/or iii) in accordance with the terms applicable for US Federal Government officers or employees acting as part of their official duties; on a worldwide, perpetual, irrevocable, royalty-free basis to BMJ Publishing Group Ltd ("BMJ") its licensees and where the relevant Journal is co-owned by BMJ to the co-owners of the Journal, to publish the Work in this journal and any other BMJ products and to exploit all rights, as set out in our licence.*

*The Submitting Author accepts and understands that any supply made under these terms is made by BMJ to the Submitting Author unless you are acting as an employee on behalf of your employer or a postgraduate student of an affiliated institution which is paying any applicable article publishing charge ("APC") for Open Access articles. Where the Submitting Author wishes to make the Work available on an Open Access basis (and intends to pay the relevant APC), the terms of reuse of such Open Access shall be governed by a Creative Commons licence – details of these licences and which Creative Commons licence will apply to this Work are set out in our licence referred to above.*

*Other than as permitted in any relevant BMJ Author's Self Archiving Policies, I confirm this Work has not been accepted for publication elsewhere, is not being considered for publication elsewhere and does not duplicate material already published. I confirm all authors consent to publication of this Work and authorise the granting of this licence.*

For peer review only

# An age-period-cohort analysis and projection of cancer mortality in Hong Kong, 1998–2030

Yanji Zhao[1,#], Zian Zhuang[1,2,#], Lin Yang[3] and Daihai He[1,4*]

1 Department of Applied Mathematics, Hong Kong Polytechnic University, Hong Kong, China

2 Department of Biostatistics, University of California, Los Angeles, CA, United States

3 School of Nursing, Hong Kong Polytechnic University, Hong Kong, China

4 Research Institute for Future Food, Hong Kong Polytechnic University, Hong Kong, China

# These authors contributed equally to this work.

* To whom correspondence should be addressed:

Department of Applied Mathematics, The Hong Kong Polytechnic University, Hong Kong, China
Tel: 852-27667864; daihai.he@polyu.edu.hk (DH).

**Email of all authors**

Yanji Zhao: yan-ji.zhao@connect.polyu.hk; Zian Zhuang: zianzhuang@ucla.edu;

Lin Yang: l.yang@polyu.edu.hk; Daihai He: daihai.he@polyu.edu.hk.

*Word count:3263*



For peer review only - http://bmjopen.bmj.com/site/about/guidelines.xhtml

# Abstract

**Objectives:** Complicated population distribution and immigration status in Hong Kong have brought out intricate causes of diseases. This study was aimed to explore age, period, birth cohort effects and effects across genders and immigration groups on mortality rates of lung, pancreatic, colon, liver, prostate and stomach cancers and their projections.

**Design, Setting, and Participants:** Death registry data in Hong Kong between 1998 and 2021, which were stratified by age, sex and immigration status. Immigration status was classified into three groups: locals born in Hong Kong, long-stay immigrants and short-stay immigrants.

**Methods:** Age-period-cohort analysis was used to examine age, period, and birth cohort effects for genders and immigration groups from 1998 to 2021. Bayesian age-period-cohort models were applied to predict the mortality rates from 2022 to 2030.

**Results:** Short-stay immigrants revealed pronounced fluctuations of mortality rates by age and of relative risks by cohort and period effects for six types of cancers than those of long-stay immigrants and locals. Decreasing trends ($p<0.05$) or plateau ($p>0.05$) of forecasting mortality rates of cancers occur for all immigration groups, except for increasing trends for short-stay male immigrants with colon cancer (16.77 deaths to 18.50 deaths/100,000 $p<0.05$) and long-stay male immigrants with pancreatic cancer (17.87 deaths to 23.49 deaths/100,000 $p<0.05$). Men will be at a higher risk of mortality from cancers than women in the future (excluding prostate cancer). Immigrants for each type of cancer and gender will be at a higher mortality risk than locals.

**Conclusions:** Findings underscore the effect of gender and immigration status in Hong Kong on mortality risks of cancers that immigrants for each type of cancer and gender will be at a higher mortality risk than locals.


***Keywords***: Age-period-cohort analysis, immigration, mortality, lung cancer, pancreatic cancer, colon cancer, liver cancer, prostate cancer, stomach cancer



For peer review only - http://bmjopen.bmj.com/site/about/guidelines.xhtml

## Strengths and limitations of this study

- This study provides new evidence regarding the relationship between immigration status and cancer mortality, given the effects of age, period, birth cohort and their predictions.

- The non-identifiability problem has not been interpreted in APC models

- The future perspective of cancer therapies and techniques have not been considered.



For peer review only

For peer review only - http://bmjopen.bmj.com/site/about/guidelines.xhtml

## Introduction

Several migration waves from mainland China to Hong Kong have occurred over the past century. These migration waves included a large-scale migration inflow from 1945 to 1950 (the Chinese Civil War) and a few small-scale inflows in the 1950s, 1970s, and 1990s [1-3]. In 2016, immigrants from mainland China formed approximately 38% of the population of Hong Kong. These inflows have led to a growing interest in research on the disparity of health conditions between the locals and immigrants.

Cancer has been one of most common causes of death, as an estimated 19.3 million new cancer cases and 9.9 million new cancer-associated deaths occurred worldwide in 2020 [4]. In Hong Kong, lung cancer is one of the most common causes of cancer deaths [5, 6]. Previous studies suggested that the primary cause of lung cancer is cigarette smoking [7-11]. Genetic factors, asbestos, radon gas, second-hand smoke, and other forms of air pollution have been proven to influence the risk of lung cancer [12-18]. The overall daily smoking rate in mainland China was approximately 23.2% in 2018 [19], whereas the daily smoking rate in Hong Kong was only 10.2% in 2019 [20]. The leading causes of liver cancer include viral infection, drinking of alcohol and polluted water and food supplies which are also culprits for colon, stomach and pancreatic cancer [21]. Alcohol consumption per capita in Hong Kong has reached to 2.37 liters in 2021 [22], compared to 7.0 liters of per capita consumption of alcohol in mainland China in 2018 [23]. As approximately 99% of prostate cancer cases occur after age 50, factors of prostate cancer have been regarded as old age, race, family history and the diet of red meat consumption [24]. In addition to these risk factors, studies have suggested that cancer mortality rates vary depending on migrant status [25-28]. According to data from the Census and Statistics Department of Hong Kong, approximately 81% of immigrants in Hong Kong immigrated from China mainland, Macau, and Taiwan. Immigrants from mainland China account for a bulk of this population. Previous studies have shown that child immigrants in Hong Kong tend to suffer from a higher risk of wheezing disorders and cardiovascular diseases, and immigrant women have higher age-specific mortality rates of breast cancer than locally-born women in Hong Kong [29, 30]. However, to date, few studies have investigated the effect of length of stay in Hong Kong and birthplace on the risk of other types of cancer.

In this study, we compared the mortality rates of lung, pancreatic, colon, liver, prostate and stomach cancers between locally born residents in Hong Kong and immigrants from mainland China. Both populations are widely considered as ethnically homogeneous with similar cultures. Nevertheless, due to different early life experiences, immigrants are exposed to more various social economy and lifestyle than locals. Therefore, it's constructive to ascertain whether immigrants from mainland China have a different mortality pattern of cancers with locals to



For peer review only - http://bmjopen.bmj.com/site/about/guidelines.xhtml

verify the significance of migration status for this health outcome. As Age-period-cohort (APC) analysis plays a vital role in studying time-specific phenomena in epidemiology, in this study, we developed APC models specified by sex and migrant status to assess the effects of age, period, birth cohort, and of the length of stay in Hong Kong on the mortality risks of cancers. Additionally, we explore the projection of mortality rates for the locally born population and immigrants in Hong Kong who were younger or older than 60 using a predictive model, taking into account age, period, and birth cohort effects as well.

## Methods

### *Data*

We obtained the death registry data in Hong Kong between 1998 and 2021 from the Census and Statistics Department of Hong Kong, as the data in 2022 has not been available up to now. The data was extracted a routine census held by Hong Kong government as subjective errors caused by resampling can be neglected. The population data were stratified by age, sex, immigration status, and length of stay in Hong Kong. We retrieved six types of cancer cases from the death registry data using ICD codes, such as ICD-9 code 162 and ICD-10 codes C34.0–C34.3, C348, and C349 for lung cancer. To assure comparability among registries, deaths from the age group of 35–85 years were selected, since cases younger than 35 and older than 85 were relatively trivial for lack of statistical interpretability [31]. Immigration status was classified into three groups: locals born in Hong Kong, immigrants who have lived in Hong Kong for >10 years before death defined as long-stay immigrants, and immigrants who have lived in Hong Kong for ≤10 years before death defined as short-stay immigrants. Notably, much focus was placed on immigrants from mainland China, because approximately 81% of immigrants in Hong Kong came from mainland China, Macau, and Taiwan based on the data from the Census and Statistics Department of Hong Kong. Moreover, few cases recorded from Macau and Taiwan are statistically insignificant in the analysis. Demographics and population projections from 2022 to 2030 were retrieved from the Census and Statistics Department of Hong Kong and estimated with cubic smoothing spline as the prerequisite of the predictive model. Codes for APC and BAPC analysis are available in the GitHub repository (https://github.com/kshz2164313/APC-population-projections-for-immigration-HK).

### *Statistical analysis*

We modeled cancer mortality rates in Hong Kong using APC analysis based on log-linear Poisson regression models. The model aimed to disentangle age, period, and cohort effects of time-varying phenomena simultaneously [32, 33], given that

$$\log(E_{ij}) = \alpha_i + \beta_j + \gamma_k + \mu + \log(\theta_{ij}) \quad (1)$$



For peer review only - http://bmjopen.bmj.com/site/about/guidelines.xhtml

where $E_{ij}$ denotes expected mortality; $\alpha_i$, $\beta_j$, and $\gamma_k$ denote age, period, and cohort effect, respectively, for $i=1,\ldots,I$, $j=1,\ldots,J$, $k=1,\ldots,K$ with $k=I-i+j$. $\log(\theta_{ij})$ is the offset. We mainly focused on the contributions of sex and immigration status due to the non-identifiability problem that the effects of these three components are collinear with each other (denoted as period – age = cohort) [34]. Birth cohort effect and period effect were assessed with relative risks. The median date of birth among cases were regarded as the reference cohort. Since cases aged at 35–85 years between 1998 and 2021 were selected, the range of birth cohort from 1913 to 1986 covered observations and further projections until 2030. The second and penultimate period effects were constrained to the reference for period. For sex and immigration status, maximum likelihood framework was applied to estimate the relative risks and 95% confidence intervals (CIs) by age groups, calendar period, and birth cohort.

Several projection approaches for future cancer mortality have been developed, but a Bayesian age-period-cohort (BAPC) model built upon integrated nested Laplace approximations (INLA) [35] yields relatively higher coverage and better performance for all evaluated parameter combinations [36]. To prevent some sampling problems caused by Markov chain Monte Carlo (MCMC), this MCMC-free BAPC approach was applied to predict future cancer mortality within a fully Bayesian inference setting and provide outputs of interest simply, such as projected age-standardized and age-specific rates. Convergence checks are not necessary for this technique [35]. The projections of age-standardized cancer mortality rates for each sex, age group (younger or older than 60 years) and migrant status, taking into account age, period, and birth cohort effects, were performed based on the weights of population age groups from the WHO World Standard population [37], with 95% prediction intervals. Mann-Kendall trend test was applied to verify the projection trend.

All analyses were performed via R version 4.2.1 (R Core Team, R Foundation for Statistical Computing, Vienna, Austria, 2013, http://www.R-project.org/). The APC models were established using the Epi package, and the projections based on Bayesian APC models were performed with the BAPC package.

*Patient and Public Involvement*

None.

## Results

Figure 1 and eFigure 1(a-e) in **Supplementary Material** illustrate the estimates of age (assessed by cancer mortality), cohort and period effects (assessed by relative risk) based on APC models among three migrant groups for men and women with six types of cancers,



For peer review only - http://bmjopen.bmj.com/site/about/guidelines.xhtml

respectively. All the mortality rates for each gender and immigration status exhibit notable increasing trends with age. Age, cohort and period effects of six types of cancer for immigrants who stayed in Hong Kong for ≤10 years revealed relatively more pronounced fluctuations and deviations from those effects in other two immigration groups. Significant increasing trends of age effect occurred in all types of cancer, regardless of gender and immigration status. For example, while relatively insignificant differences in lung cancer mortality rates by immigration status among females, male immigrants who remained in Hong Kong for >10 years had higher lung cancer mortality rates at ages above 50 years and those who arrived ≤10 years had lower lung cancer mortality at ages below 62 years compared to local men Figure 1. In addition to compatible dynamics of period effect for locals and long-stay immigrants, similar change of relative risks by birth cohort for locals and long-stay immigrants in lung, colon, liver and stomach cancers occurred before 1945, whereas significant differences of relative risks by birth cohort between these two immigration groups occurred after 1960 (Figure 1 & eFigure 1(a,b,d)). Locals and long-stay immigrants in pancreatic and prostate cancer perform almost similar change of relative risks by birth cohort effects all the time (eFigure 1(c,e)). Short-stay immigrants who have stayed in Hong Kong for ≤10 years had more fluctuating relative risks affected by period effects before 2020 than those for locals and long-stay immigrants. Lack of young cases, especially young short-stay immigrants, of prostate cancer leads to significant deviations and variances in age and cohort effects.

Figure 2-4, eFigure 2-6 in **Supplementary Material** illustrate the age-standardized mortality rates of six types of cancer from 1998 to 2021 and their projections by sex, immigrant status and age groups from 2022 to 2030, taking into account age, period, and birth cohort effects. Means and standard deviations of predictive mortality rates are shown in eTable 1-6 in **Supplementary Material**. For all ages projection (Figure 2 & eFigure 2-6), monotone decreasing trends or plateau of forecasting occur for both genders and all immigration groups in cancers, except for increasing trends for male immigrants who have stayed in Hong Kong for ≤10 years with colon cancer ($p$-value < 0.05) and immigrants who have stayed in Hong Kong for > 10 years with pancreatic cancer ($p$-values < 0.05). Most of predictive trends for younger cases (<60 years) and older cases (≥60 years) reach a consensus with those for all ages population, except for two phenomenon: 1.) mortality rates of lung cancer for men immigrants ≤10 that insignificant trend for all ages ($p$-value > 0.05) vs. decline for younger cases ($p$-value < 0.05) vs. increase for older cases ($p$-value < 0.05); 2.) mortality rates of liver cancer for men immigrants >10 that decline for all ages ($p$-value < 0.05) vs. decline for younger cases ($p$-value < 0.05) vs. insignificant trend for older cases ($p$-value > 0.05). Men will be at higher risk of mortality rates of cancers (excluding prostate cancer) than women in the future for all three age groups (all ages, young and older than 60 years). Given the future developing trends,



For peer review only - http://bmjopen.bmj.com/site/about/guidelines.xhtml

immigrants, especially the group of immigrant who have stayed in Hong Kong for >10 years with lung, liver, pancreatic, prostate and colon cancer, will have relatively higher mortality rates in each year for each gender compared with locals and pronounced decline in predictive means (all p-values < 0.05). Some particular cases occur in the projection of prostate cancer that young long-stay male immigrants (0.44 deaths/100,000) aged less than 60 will be at lower mortality rate than locals (0.69 deaths/100,000) in 2030 (eTable 6). Compared with other cancers and immigration groups, male immigrants who have stayed in Hong Kong for >10 years with lung cancer would perform the most significant decline in predictive mean from 100.18 to 79.55 deaths per 100,000 population (average 2.92 deaths/100,000 per annum) (eTable 1), while the same immigration group with pancreatic cancer would indicate the most significant uptrend in each year of 17.87 deaths and 23.49 deaths per 100,000 population in 2022 and 2030, respectively (average 0.62 deaths/100,000 per annum) (eTable 4).

## Discussion

Early detection of cancer is positive and instructive for increasing chances of cure. Nevertheless, the high mortality rate of cancer results from late diagnosis among most patients after progression to more advanced or severe stages. Individuals at high risk of cancer, such as smokers, alcoholics or those who are frequently exposed to susceptible circumstances, should be screened for early detections to increase opportunities for cure [38]. Therefore, the differences in mortality rates among immigration groups are synonymous with detection means, therapies, and social history in disparate periods and areas.

While the changes in mortality rates by age for long-stay immigrants reached approximate harmony with those for locals, the changes in mortality rates by age for short-stay immigrants revealed clear differences with those for other two populations. The group of long-stay immigrants had a higher risk of death from lung, colon and liver cancers than the other two immigration groups after the age of 60 years. Short-stay male immigrants were less likely to die from lung cancer before the age of 65 years. The contrast in age effects among the immigration groups was partially consistent with studies [25, 39] that highlighted the age effects for locals and immigrants on breast cancer mortality in Hong Kong and lung cancer incidence in Sweden, as they both showed similar trends and magnitudes between locals and immigrants before the age of 60 years. They are also compatible with the results in [40] that diagnosis of liver cancer is the most frequent among populations at 55 to 65 years old. According to these trends, young individuals, especially new young immigrant men, who have benefited from all-rounded development in mainland China and Hong Kong, are more likely to seek early detection and be treated for cancers using more advanced treatments [41].



For peer review only - http://bmjopen.bmj.com/site/about/guidelines.xhtml

Differences in birth cohort effects among immigrant groups partially comply with the interpretation above.

We observed significant trends of cohort effects among locals and immigrants. These findings are partially consistent but subtly different from previous findings, regarding the effect of immigration status on cancers. Zhao et al. [25] described multiple peaks of cohort effects on breast cancer mortality between locals and immigrants in Hong Kong, as well as a significant decline of cohort effects after 1950. In contrast, Sung et al. [42] investigated the difference in breast cancer incidence between Chinese Americans and non-Hispanic whites in the U.S. and emphasized that Chinese Americans were at lower risk of breast cancer than non-Hispanic whites born in the same year. Here, we interpret the cohort-driven trends resulting from the intricacy of social history and lifestyle. Compared to a relatively stable social development in Hong Kong, representing downward trends of relative risks for locals, wars and social instability in mainland China resulted in several immigration waves from mainland China to Hong Kong before 1950. Additionally, remarkable increasing trends were recorded for new immigrants after 1950, which corresponded to the economic downturn after wars and famine between 1959 and 1961 during their youth [43]. The increasing trends for new immigrants and similar trends for locals and long-stay immigrants were consistent with the finding that nutrient deficiency contributes to higher risk of severe mortality rates of cancers [44]. Furthermore, we speculate that these trends, especially those for locals and long-stay immigrants, are most likely attributed to social development and personal behaviors, such as daily habits, occupational history, different diagnoses and treatments, and domestic environmental exposures. It's notable that short-stay immigrants suffered from lower risk of death from colon cancer for all ages (eFigure 1a in **Supplementary Material**). As locals and immigrants in Hong Kong transitioned to a more westernized lifestyles, higher consumption of meat was associated with a higher risk of these types of cancer, whereas consumptions of vegetables had a strong protective effect against pancreatic cancer, and moderate consumption of coffee appeared to be beneficial against lung cancer [45,46]. Further studies on potential risk factors are required.

Short-stay immigrants had more fluctuating and non-stationary but inconspicuous relative risks by period effects before 2021 than locals and long-stay immigrants. Cumulatively, an arch pattern and fluctuating curve depicting period effects externally resulted in an arch pattern of age-standardized mortality rates for short-stay immigrant women and irregular rates for short-stay immigrant men before 2021. The external performance of different period effects on mortality rates could be most likely attributed to the higher effect of different lifestyles and social development on new immigrants than on long-stay immigrants and locals in Hong Kong. With respect to the age-standardized mortality rates and projections,



For peer review only - http://bmjopen.bmj.com/site/about/guidelines.xhtml

consistent with previous findings [47,48], we predict that the mortality rates of cancer in Hong Kong after 2021 will continue to decline or remain relatively stable, consistent with the trends before 2020, except for male immigrants who have stayed in Hong Kong for ≤10 years with colon cancer and male immigrants who have stayed in Hong Kong for >10 years with pancreatic cancer. Men will be at higher risk of mortality rates of cancer than women, regardless of immigration status. They are also compatible with the results in [4] that men suffer from higher risk of these types of cancer than women, excluding prostate cancer. Furthermore, new immigrant women will be at lower risk than local women, even though long-stay immigrants will suffer from higher mortality rates than locals in the future. Potential interpretations could be consistent with those for birth cohort effects, as age and period effect are considered as confounders of cohort effect.

In the past few decades, spurred by an increasing burden of high incidence and mortality rates of cancer, several studies focused on the inherent identification dilemma of three effects in the APC model. Further, complicated population distribution and immigration status in Hong Kong, one of the areas with the highest population density and migration frequency in the world, have intricate causes and inherent dynamics of cancer and other diseases. To our knowledge, few studies have assessed the relationship between immigration status and cancer mortality. Therefore, this study is original to examine the effect of the length of stay in Hong Kong and origins of previous residence on cancer deaths, which is instructive for further immigration policy making and targeted strategies of disease detection and intervention. However, this study had several limitations. Given the non-identifiability problem in age-period-cohort models, we could only depict trends and variations among different immigration and sex groups, as illustrated in figures, and insufficiently perform the estimates of the contributions of three effects or subgroups to mortality rates. Furthermore, we adopted cubic smoothing spline to estimate populations of immigrants and locals due to the large proportion of unspecified immigration status from official demographic projections. A few acceptable cases resulted in a limited type of cancer so that some common cancers such as ovary and cervix, were discarded. Since the issue of quantification, the future perspective of cancer therapies and techniques have not been considered in the model of projection.

## Conclusion

We conclude that immigrants, especially short-stay immigrants, had more pronounced fluctuations of mortality rates by age and of relative risks by cohort and period effects for six types of cancers than those of long-stay immigrants and locals. Men will be at a higher risk of mortality rates of six types of cancer than women in the future. Male immigrants who have



For peer review only - http://bmjopen.bmj.com/site/about/guidelines.xhtml

stayed in Hong Kong for ≤10 years with colon cancer and male immigrants who have stayed in Hong Kong for >10 years with pancreatic cancer would perform significant uptrend in the future, while other immigration groups for each type of cancer would continue to decline or remain relatively stable. Immigrants for each gender in Hong Kong would suffer from higher mortality risks of cancers than locals in the future.

For peer review only



For peer review only - http://bmjopen.bmj.com/site/about/guidelines.xhtml

## Declaration

### Ethical approval and consent to participate

Ethical approval and consent to participate are not applicable. This study does not involve human participants. Data was obtained from the Census and Statistics Department of Hong Kong.

### Consent for publication

Not applicable.

### Data Availability Statement

Data are available upon reasonable request.

### Author contributions

**Yanji Zhao:** Methodology, Formal analysis, Data Curation, Writing - Original Draft, Visualization
**Zian Zhuang:** Methodology, Formal analysis, Data Curation, Writing - Review & Editing
**Lin Yang:** Validation, Writing - Review & Editing
**Daihai He:** Conceptualization, Writing - Review & Editing, Supervision

### Funding

The work described in this paper was partially supported by a grant from the Research Grants Council of the Hong Kong Special Administrative Region, China (HKU C7123-20G).

### Conflict of interest

None declared.

### Acknowledgements

None.

For peer review only



For peer review only - http://bmjopen.bmj.com/site/about/guidelines.xhtml

## References

1.Fan S-C. The population projection of Hong Kong. Southeast Asian Journal of Social Science. 1974;2(1/2):105-17.

2.Department CaS. Hong Kong Statistics 1947-1967 (Report). https://www.statistics.gov.hk/pub/hist/1961_1970/B10100031967AN67E0100.pdf, Accessed 4th May 2019.

3. Department CaS. Demographic Trends in Hong Kong 1981-2011 (Report). http://www.statistics.gov.hk/pub/B1120017032012XXXXB0100.pdf, Accessed 4th May 2019.

4. Sung H, Ferlay J, Siegel RL, Laversanne M, Soerjomataram I, Jemal A, et al. Global Cancer Statistics 2020: GLOBOCAN Estimates of Incidence and Mortality Worldwide for 36 Cancers in 185 Countries. CA: A Cancer Journal for Clinicians. 2021 2021/05/01;71(3):209-49. doi: https://doi.org/10.3322/caac.21660.

5. Wang XR, Chiu YL, Qiu H, Au JSK, Yu ITS. The roles of smoking and cooking emissions in lung cancer risk among Chinese women in Hong Kong. Annals of Oncology. 2009 2009/04/01/;20(4):746-51. doi: https://doi.org/10.1093/annonc/mdn699.

6. Chiu Y-L, Wang X-R, Qiu H, Yu IT-S. Risk factors for lung cancer: a case–control study in Hong Kong women. Cancer Causes & Control. 2010 2010/05/01;21(5):777-85. doi: 10.1007/s10552-010-9506-9.

7. Office on S, Health. Publications and Reports of the Surgeon General. Women and Smoking: A Report of the Surgeon General. Atlanta (GA): Centers for Disease Control and Prevention (US); 2001.

8. Escobedo LG, Peddicord JP. Smoking prevalence in US birth cohorts: the influence of gender and education. American Journal of Public Health. 1996 1996/02/01;86(2):231-6. doi: 10.2105/AJPH.86.2.231.

9. Husten CG, Shelton DM, Chrismon JH, Lin YC, Mowery P, Powell FA. Cigarette smoking and smoking cessation among older adults: United States, 1965-94. Tobacco Control. 1997;6(3):175. doi: 10.1136/tc.6.3.175.

10. Bolego C, Poli A, Paoletti R. Smoking and gender. Cardiovascular Research. 2002;53(3):568-76. doi: 10.1016/S0008-6363(01)00520-X.

11. Doll R, Hill AB. The mortality of doctors in relation to their smoking habits; a preliminary report. Br Med J. 1954;1(4877):1451-5. PMID: 13160495. doi: 10.1136/bmj.1.4877.1451.

12. Ramada Rodilla JM, Calvo Cerrada B, Serra Pujadas C, Delclos GL, Benavides FG. Fiber burden and asbestos-related diseases: an umbrella review. Gaceta Sanitaria. 2021 2021/06/11/. doi: https://doi.org/10.1016/j.gaceta.2021.04.001.

13. Collishaw NE, Kirkbride J, Wigle DT. Tobacco smoke in the workplace: an occupational health hazard. Can Med Assoc J. 1984;131(10):1199-204. PMID: 6498670.

14. Dresler CM, Fratelli C, Babb J, Everley L, Evans AA, Clapper ML. Gender differences in genetic susceptibility for lung cancer. Lung Cancer. 2000 2000/12/01/;30(3):153-60. doi: https://doi.org/10.1016/S0169-5002(00)00163-X.

15. Alexandrov K, Cascorbi I, Rojas M, Bouvier G, Kriek E, Bartsch H. CYP1A1 and GSTM1 genotypes affect benzo[ a ]pyrene DNA adducts in smokers' lung: comparison with aromatic/hydrophobic adduct formation. Carcinogenesis. 2002;23(12):1969-77. doi:



For peer review only - http://bmjopen.bmj.com/site/about/guidelines.xhtml

10.1093/carcin/23.12.1969.

16. Samet JM. Radon and Lung Cancer. JNCI: Journal of the National Cancer Institute. 1989;81(10):745-58. doi: 10.1093/jnci/81.10.745.

17. Darby S, Hill D, Auvinen A, Barros-Dios JM, Baysson H, Bochicchio F, et al. Radon in homes and risk of lung cancer: collaborative analysis of individual data from 13 European case-control studies. BMJ. 2005;330(7485):223. doi: 10.1136/bmj.38308.477650.63.

18. Raaschou-Nielsen O, Andersen ZJ, Beelen R, Samoli E, Stafoggia M, Weinmayr G, et al. Air pollution and lung cancer incidence in 17 European cohorts: prospective analyses from the European Study of Cohorts for Air Pollution Effects (ESCAPE). The Lancet Oncology. 2013 2013/08/01/;14(9):813-22. doi: https://doi.org/10.1016/S1470-2045(13)70279-1.

19. *2018 Summary-20190719*. Retrieved August 26, 2022, from https://www.who.int/docs/default-source/wpro---documents/countries/china/2018-gats-china-factsheet-cn-en.pdf?sfvrsn=3f4e2da9_2

20. *Thematic household survey*.Retrieved August 26, 2022, from https://www.censtatd.gov.hk/en/data/stat_report/product/B1130201/att/B11302702020XXXXB0100.pdf

21. Abubakar II, Tillmann T, Banerjee A. Global, regional, and national age-sex specific all-cause and cause-specific mortality for 240 causes of death, 1990-2013: a systematic analysis for the Global Burden of Disease Study 2013. Lancet. 2015 Jan 10;385(9963):117-71.

22. Estimated alcohol consumption per capita in Hong Kong. Change4Health. (n.d.). Retrieved December 1, 2022, from https://www.change4health.gov.hk/en/alcohol_aware/figures/alcohol_consumption/index.html

23. World Health Organization. Global status report on alcohol and health 2018. World Health Organization; 2019 Feb 14.

24. Wild C. World cancer report 2014. Wild CP, Stewart BW, editors. Geneva, Switzerland: World Health Organization; 2014.

25. Zhao S, Dong H, Qin J, Liu H, Li Y, Chen Y, et al. Breast cancer mortality in Chinese women: does migrant status play a role? Annals of Epidemiology. 2019 2019/12/01/;40:28-34.e2. doi: https://doi.org/10.1016/j.annepidem.2019.10.006.

26. Gomez SL, Yang J, Lin S-W, McCusker M, Sandler A, Cheng I, et al. Incidence trends of lung cancer by immigration status among Chinese Americans. Cancer Epidemiol Biomarkers Prev. 2015;24(8):1157-64. PMID: 25990553. doi: 10.1158/1055-9965.EPI-15-0123.

27.Hemminki K, Li X, Czene K. Cancer risks in first-generation immigrants to Sweden. International Journal of Cancer. 2002 2002/05/10;99(2):218-28. doi: https://doi.org/10.1002/ijc.10322.

28. Vanthomme K, Rosskamp M, De Schutter H, Vandenheede H. Lung cancer incidence differences in migrant men in Belgium, 2004–2013: histology-specific analyses. BMC Cancer. 2021 2021/03/30;21(1):328. doi: 10.1186/s12885-021-08038-6.

29. Schooling M, Leung GM, Janus ED, Ho SY, Hedley AJ, Lam TH. Childhood migration and cardiovascular risk. International Journal of Epidemiology. 2004;33(6):1219-26. doi: 10.1093/ije/dyh221.

30. Leung JYY, Li AM, Leung GM, Schooling CM. Mode of delivery and childhood



For peer review only

For peer review only - http://bmjopen.bmj.com/site/about/guidelines.xhtml

hospitalizations for asthma and other wheezing disorders. Clinical & Experimental Allergy. 2015 2015/06/01;45(6):1109-17. doi: https://doi.org/10.1111/cea.12548.

31. Baker A, Bray I. Bayesian projections: what are the effects of excluding data from younger age groups?. American Journal of Epidemiology. 2005 Oct 15;162(8):798-805.

32. Rosenberg PS, Anderson WF. Age-Period-Cohort Models in Cancer Surveillance Research: Ready for Prime Time? APC Models. Cancer Epidemiology, Biomarkers & Prevention. 2011 Jul 1;20(7):1263-8.

33. Holford T. Analyzing the effects of age, period and cohort on incidence and mortality rates. Stat Meth Med Res. 1992;1:317-37.

34. Brookmeyer R, Stroup DF, editors. Monitoring the health of populations: statistical principles and methods for public health surveillance. Oxford University Press; 2004.

35. Riebler A, Held L. Projecting the future burden of cancer: Bayesian age–period–cohort analysis with integrated nested Laplace approximations. Biometrical Journal. 2017 May;59(3):531-49.

36. Knoll M, Furkel J, Debus J, Abdollahi A, Karch A, Stock C. An R package for an integrated evaluation of statistical approaches to cancer incidence projection. BMC medical research methodology. 2020 Dec;20(1):1-1.

37. Ahmad OB, Boschi-Pinto C, Lopez AD, Murray CJ, Lozano R, Inoue M. Age standardization of rates: a new WHO standard. Geneva: World Health Organization. 2001 Jan;9(10):1-4.

38. Sung H, Ferlay J, Siegel RL, Laversanne M, Soerjomataram I, Jemal A, Bray F. Global cancer statistics 2020: GLOBOCAN estimates of incidence and mortality worldwide for 36 cancers in 185 countries. CA: a cancer journal for clinicians. 2021 May;71(3):209-49.

39. Mousavi SM, Fallah M, Sundquist K, Hemminki K. Age-and time-dependent changes in cancer incidence among immigrants to Sweden: colorectal, lung, breast and prostate cancers. International journal of cancer. 2012 Jul 15;131(2):E122-8.

40. National Cancer Institute. SEER stat fact sheets: liver and intrahepatic bile duct cancer.

41. Wu X, Chung VC, Hui EP, Ziea ET, Ng BF, Ho RS, Tsoi KK, Wong S, Wu JC. Effectiveness of acupuncture and related therapies for palliative care of cancer: overview of systematic reviews. Scientific reports. 2015 Nov 26;5(1):1-5.

42. Sung H, Rosenberg PS, Chen WQ, Hartman M, Lim WY, Chia KS, Wai-Kong Mang O, Tse L, Anderson WF, Yang XR. The impact of breast cancer-specific birth cohort effects among younger and older C hinese populations. International journal of cancer. 2016 Aug 1;139(3):527-34.

43. The world economy volume 1: a millennial perspective, 2, Historical statistics: Academic Foundation, Gurgaon, India (2007)

44. Elias SG, Peeters PH, Grobbee DE, van Noord PA. The 1944-1945 Dutch famine and subsequent overall cancer incidence. Cancer Epidemiology Biomarkers & Prevention. 2005 Aug;14(8):1981-5.

45. Chiu YL, Wang XR, Qiu H, Yu IT. Risk factors for lung cancer: a case–control study in Hong Kong women. Cancer Causes & Control. 2010 May;21(5):777-85.

46. Li J, Lam AS, Yau ST, Yiu KK, Tsoi KK. Antihypertensive treatments and risks of lung Cancer: A large population-based cohort study in Hong Kong. BMC cancer. 2021 Dec;21(1):1-9.



For peer review only - http://bmjopen.bmj.com/site/about/guidelines.xhtml

489 47. Du J, Sun H, Sun Y, Du J, Cao W, Sun S. Assessment of age, period, and cohort effects of lung cancer incidence in Hong Kong and projection up to 2030 based on changing demographics. American Journal of Cancer Research. 2021;11(12):5902.
492 48. *Centre for Health Protection, Department of Health - Lung Cancer*. Centre for Health Protection. Retrieved August 10, 2022, from https://www.chp.gov.hk/en/healthtopics/content/25/49.html

For peer review only



For peer review only - http://bmjopen.bmj.com/site/about/guidelines.xhtml

**Figure 1**. Parameter estimates of age (a), cohort (b) and period (c) effects based on an age-period-cohort model of male and female lung cancer mortality rates by immigrant groups: locals, immigrants stay in Hong Kong for more than 10 years and immigrants stay in Hong Kong for less than or equal to 10 years. Age effect was assessed by mortality (left axis). Cohort and period effects were assessed by relative risk (right axis), 95% confidence intervals are shown as shaded bands.

For peer review only

**Figure 2.** Projections of lung cancer mortality rates by gender and immigrant status from 2022 to 2030. Observations are shown as dots with the predictive distribution between the 5% and 95% quantile, whereby each lighter shade of red represents an additional 10% predictive CI. The predictive mean is shown as black solid line and the vertical dashed line indicates where prediction started.

For peer review only



For peer review only - http://bmjopen.bmj.com/site/about/guidelines.xhtml

**Figure 3.** Projections of lung cancer mortality rates for the population younger than 60 by gender and immigrant status from 2022 to 2030. Observations are shown as dots with the predictive distribution between the 5% and 95% quantile, whereby each lighter shade of red represents an additional 10% predictive CI. The predictive mean is shown as black solid line and the vertical dashed line indicates where prediction started.

For peer review only



For peer review only - http://bmjopen.bmj.com/site/about/guidelines.xhtml

514
515 **Figure 4.** Projections of lung cancer mortality rates for the population older than or equal to 60 by gender and
516 immigrant status from 2022 to 2030. Observations are shown as dots with the predictive distribution between the 5%
517 and 95% quantile, whereby each lighter shade of red represents an additional 10% predictive CI. The predictive mean
518 is shown as black solid line and the vertical dashed line indicates where prediction started.
519

For peer review only



For peer review only - http://bmjopen.bmj.com/site/about/guidelines.xhtml

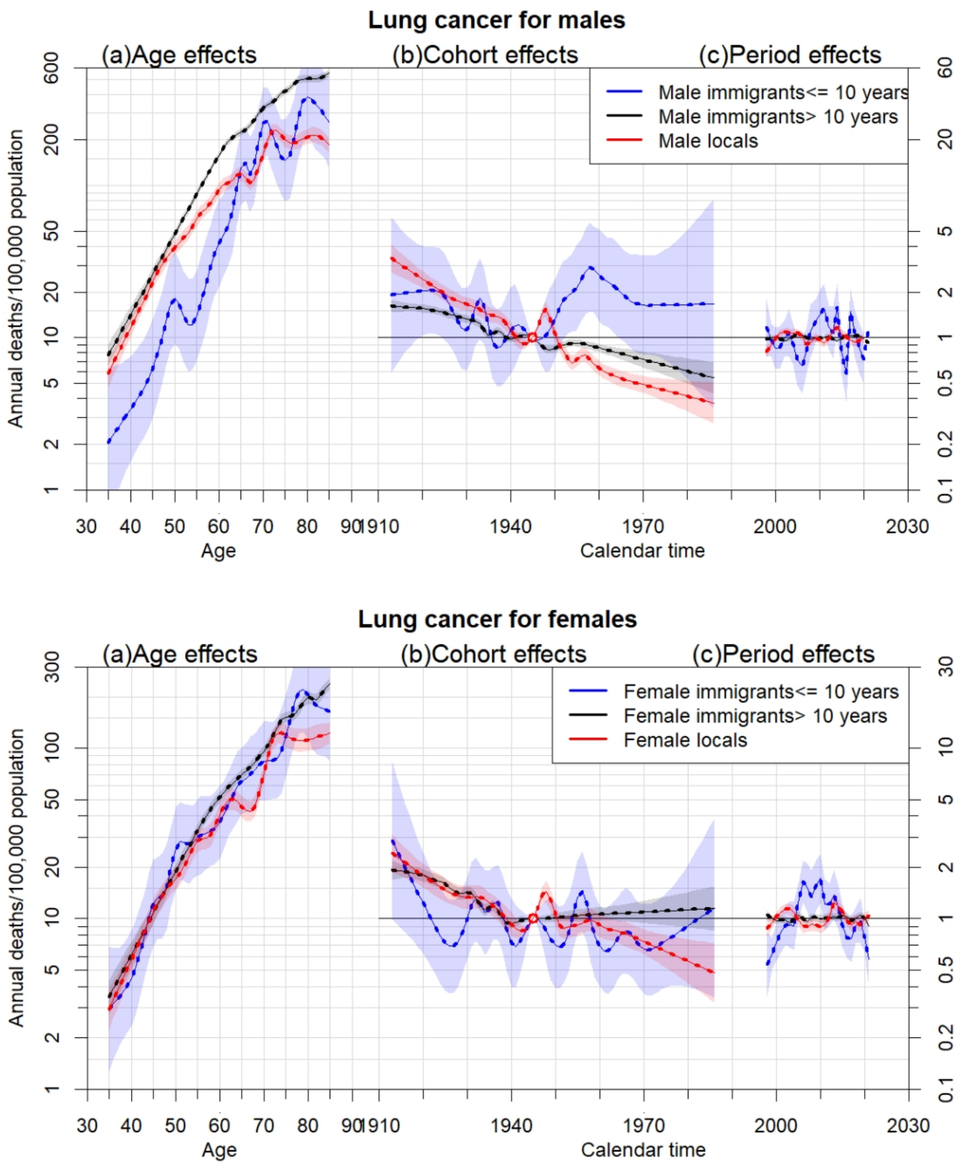


Figure 1. Parameter estimates of age (a), cohort (b) and period (c) effects based on an age-period-cohort model of male and female lung cancer mortality rates by immigrant groups: locals, immigrants stay in Hong Kong for more than 10 years and immigrants stay in Hong Kong for less than or equal to 10 years. Age effect was assessed by mortality (left axis). Cohort and period effects were assessed by relative risk (right axis), 95% confidence intervals are shown as shaded bands.

519x641mm (57 x 57 DPI)

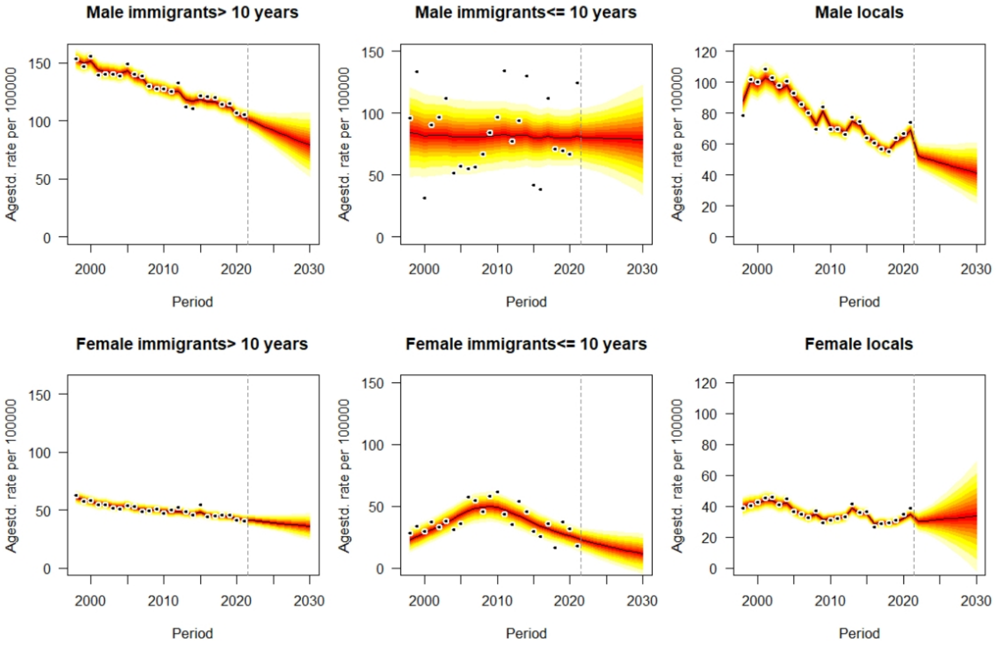


Figure 2. Projections of lung cancer mortality rates by gender and immigrant status from 2022 to 2030. Observations are shown as dots with the predictive distribution between the 5% and 95% quantile, whereby each lighter shade of red represents an additional 10% predictive CI. The predictive mean is shown as black solid line and the vertical dashed line indicates where prediction started.

519x343mm (57 x 57 DPI)

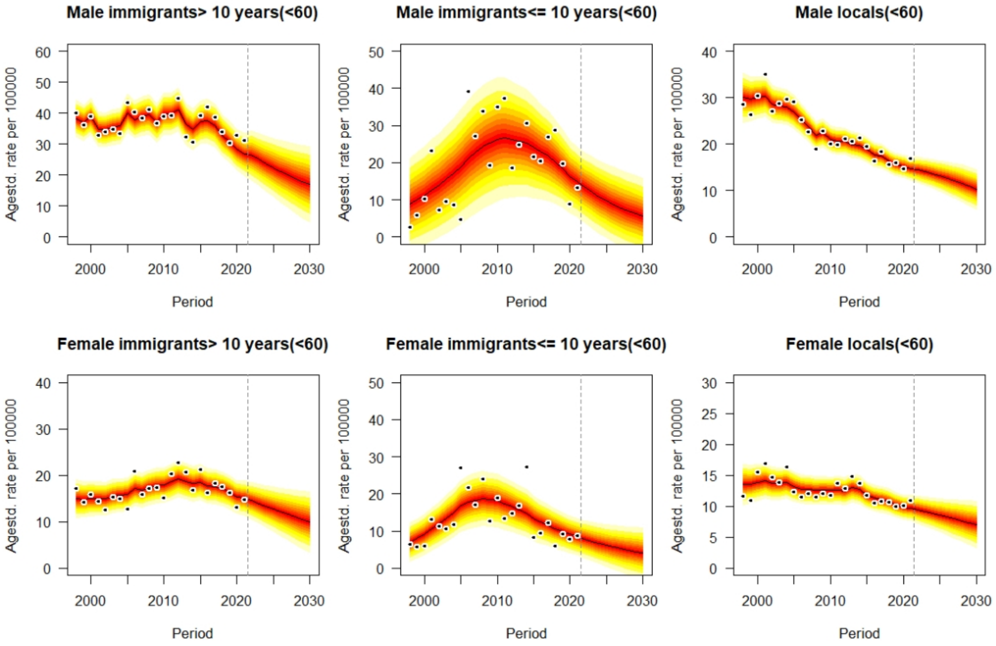


Figure 3. Projections of lung cancer mortality rates for the population younger than 60 by gender and immigrant status from 2022 to 2030. Observations are shown as dots with the predictive distribution between the 5% and 95% quantile, whereby each lighter shade of red represents an additional 10% predictive CI. The predictive mean is shown as black solid line and the vertical dashed line indicates where prediction started.

519x343mm (57 x 57 DPI)

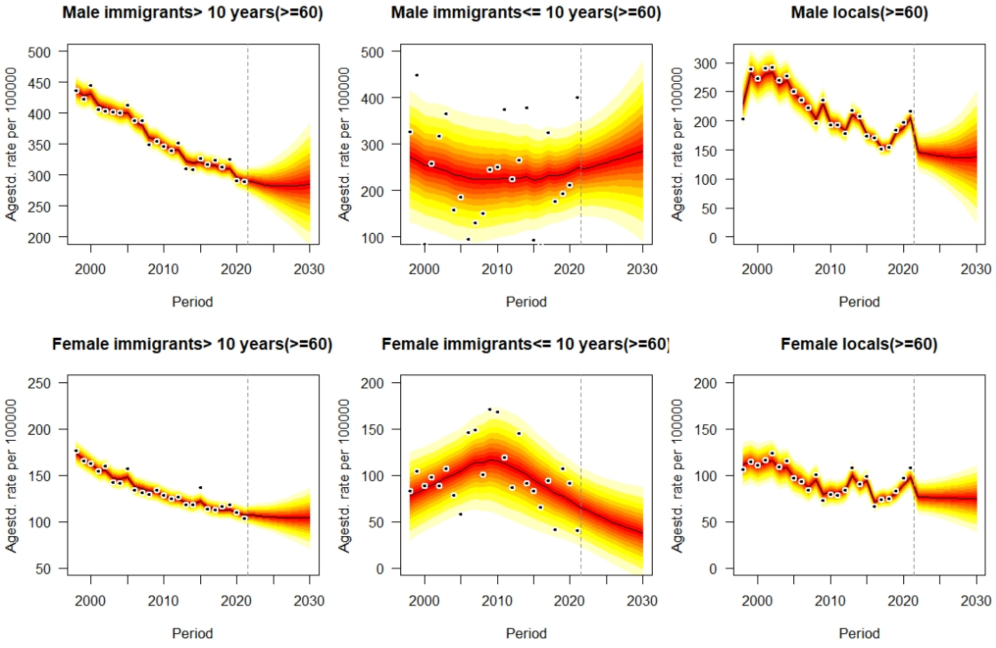


Figure 4. Projections of lung cancer mortality rates for the population older than or equal to 60 by gender and immigrant status from 2022 to 2030. Observations are shown as dots with the predictive distribution between the 5% and 95% quantile, whereby each lighter shade of red represents an additional 10% predictive CI. The predictive mean is shown as black solid line and the vertical dashed line indicates where prediction started.

519x343mm (57 x 57 DPI)

# Supplementary Material for

# "An age-period-cohort analysis and projection of cancer mortality in Hong Kong, 1998–2030"

For peer review only

## APC Figures

eFigure 1(a) Colon cancer ....................3
eFigure 1(b) Liver cancer ....................4
eFigure 1(c) Pancreatic cancer ....................5
eFigure 1(d) Stomach cancer ....................6
eFigure 1(e) Prostate cancer....................7

## Projection Figures

Colon cancer
eFigure 2(a)Projection (all ages) ....................8
eFigure 2(b)Projection (<60 years) ....................9
eFigure 2(c)Projection (⩾60 years) ....................10
Liver cancer
eFigure 3(a)Projection (all ages) ....................11
eFigure 3(b)Projection (<60 years) ....................12
eFigure 3(c)Projection (⩾60 years) ....................13
Pancreatic cancer
eFigure 4(a)Projection (all ages) ....................14
eFigure 4(b)Projection (<60 years) ....................15
eFigure 4(c)Projection (⩾60 years) ....................16
Stomach cancer
eFigure 5(a)Projection (all ages) ....................17
eFigure 5(b)Projection (<60 years) ....................18



For peer review only - http://bmjopen.bmj.com/site/about/guidelines.xhtml

eFigure 5(c)Projection (⩾60 years) ........ 19

Prostate cancer

eFigure 6 Projection (all ages, <60 years and ⩾60 years) ........ 20

## Tables

eTable 1 Lung cancer ........ 21

eTable 2 Colon cancer ........ 22

eTable 3 Liver cancer ........ 23

eTable 4 Pancreatic cancer ........ 24

eTable 5 Stomach cancer ........ 25

eTable 6 Prostate cancer ........ 26

For peer review only



For peer review only - http://bmjopen.bmj.com/site/about/guidelines.xhtml

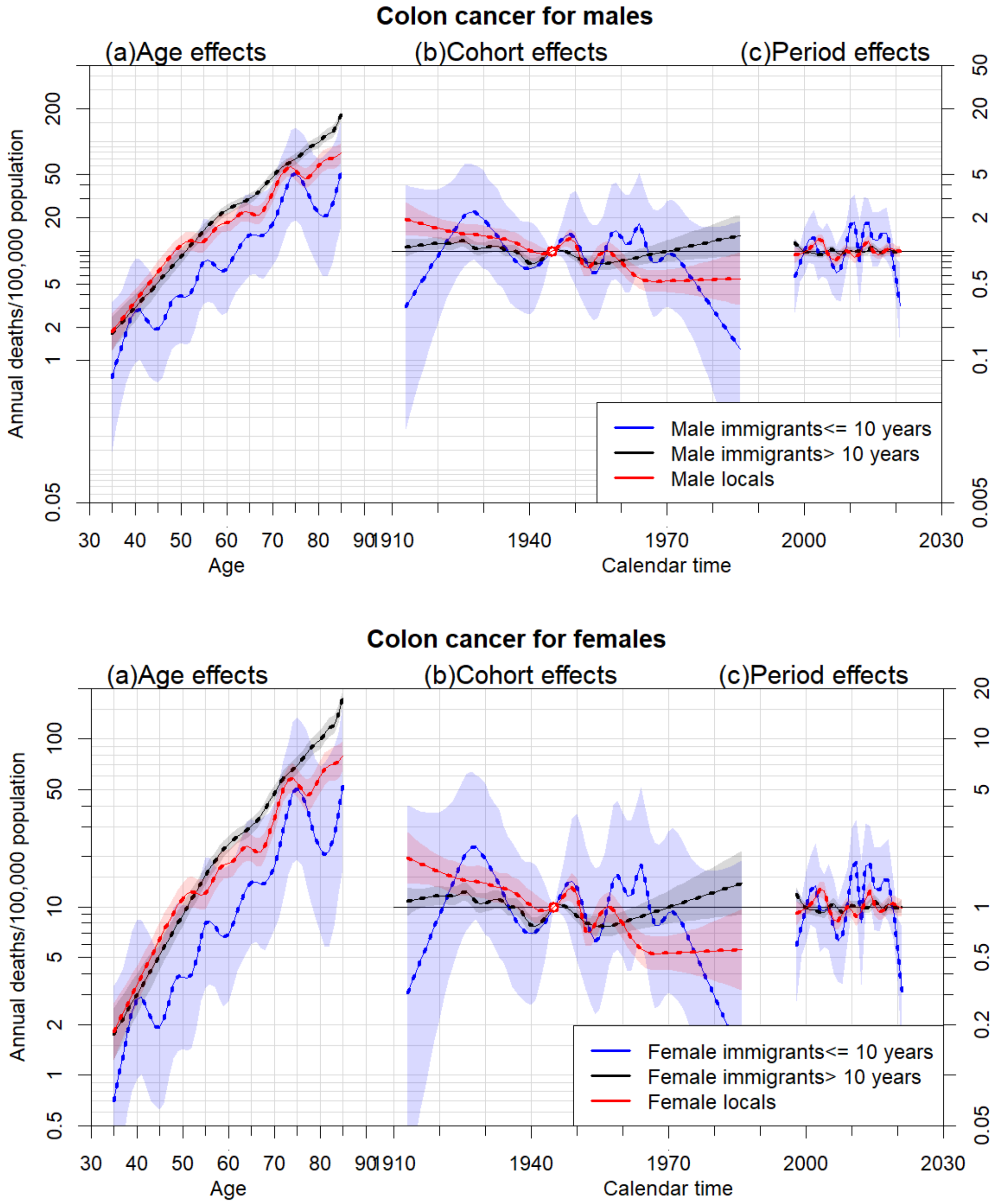


**eFigure 1(a)**. Parameter estimates of age (a), cohort (b) and period (c) effects based on an age-period-cohort model of male and female colon cancer mortality rates by immigrant groups: locals, immigrants stay in Hong Kong for more than 10 years and immigrants stay in Hong Kong for less than or equal to 10 years. Age effect was assessed by mortality (left axis). Cohort and period effects were assessed by relative risk (right axis), 95% confidence intervals are shown as shaded bands.



For peer review only - http://bmjopen.bmj.com/site/about/guidelines.xhtml

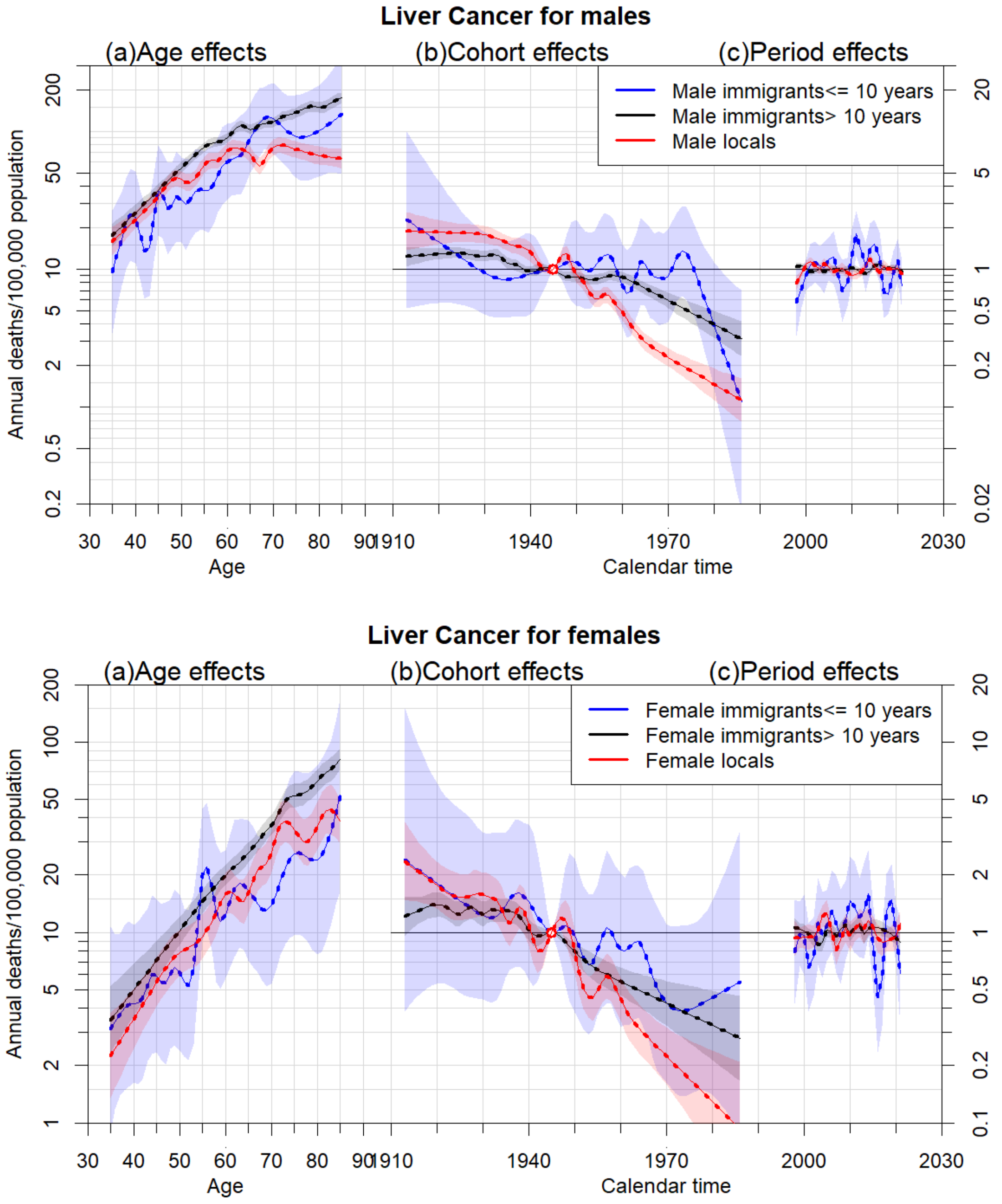


**eFigure 1(b)**. Parameter estimates of age (a), cohort (b) and period (c) effects based on an age-period-cohort model of male and female liver cancer mortality rates by immigrant groups: locals, immigrants stay in Hong Kong for more than 10 years and immigrants stay in Hong Kong for less than or equal to 10 years. Age effect was assessed by mortality (left axis). Cohort and period effects were assessed by relative risk (right axis), 95% confidence intervals are shown as shaded bands.



For peer review only - http://bmjopen.bmj.com/site/about/guidelines.xhtml

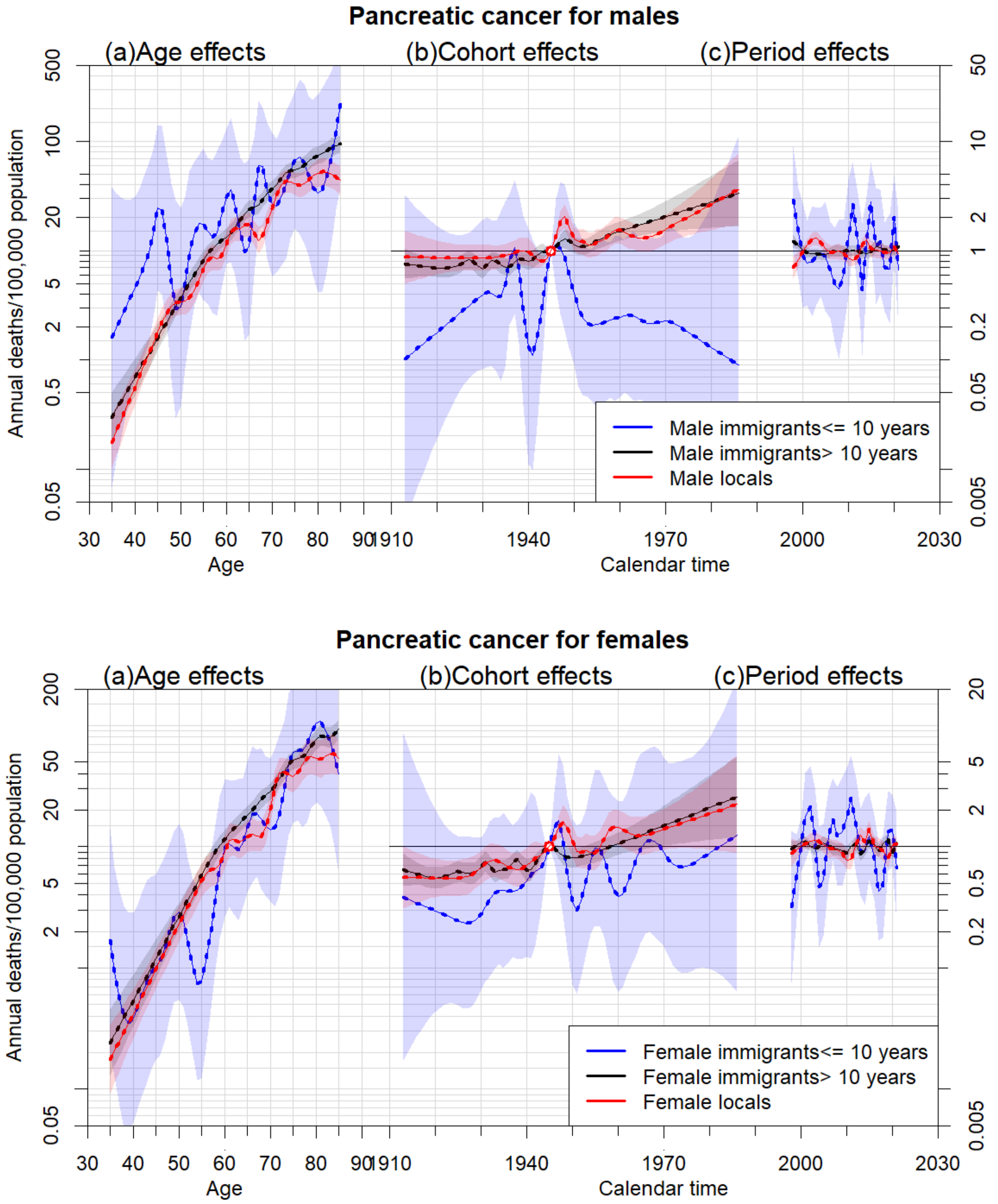


**eFigure 1(c)**. Parameter estimates of age (a), cohort (b) and period (c) effects based on an age-period-cohort model of male and female pancreatic cancer mortality rates by immigrant groups: locals, immigrants stay in Hong Kong for more than 10 years and immigrants stay in Hong Kong for less than or equal to 10 years. Age effect was assessed by mortality (left axis). Cohort and period effects were assessed by relative risk (right axis), 95% confidence intervals are shown as shaded bands.



For peer review only - http://bmjopen.bmj.com/site/about/guidelines.xhtml

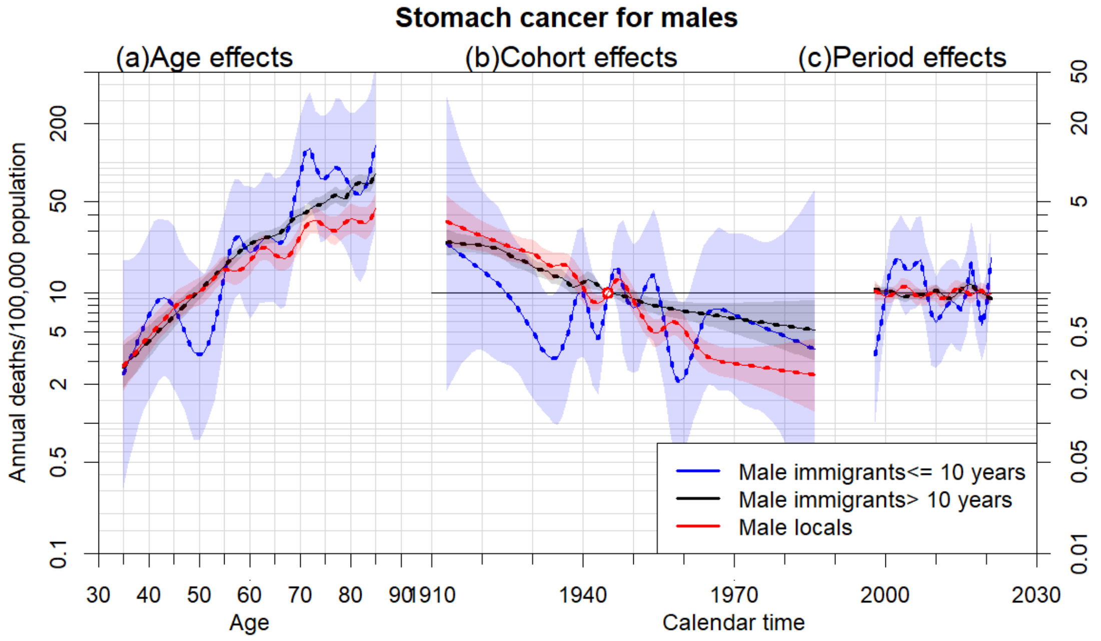


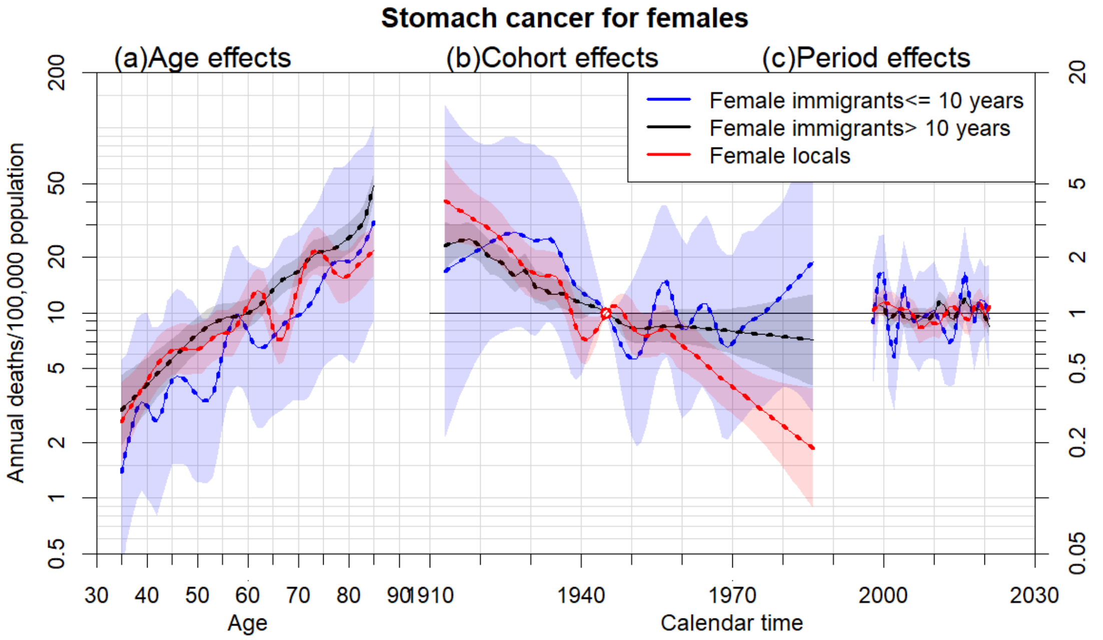


**eFigure 1(d)**. Parameter estimates of age (a), cohort (b) and period (c) effects based on an age-period-cohort model of male and female stomach cancer mortality rates by immigrant groups: locals, immigrants stay in Hong Kong for more than 10 years and immigrants stay in Hong Kong for less than or equal to 10 years. Age effect was assessed by mortality (left axis). Cohort and period effects were assessed by relative risk (right axis), 95% confidence intervals are shown as shaded bands.



For peer review only - http://bmjopen.bmj.com/site/about/guidelines.xhtml

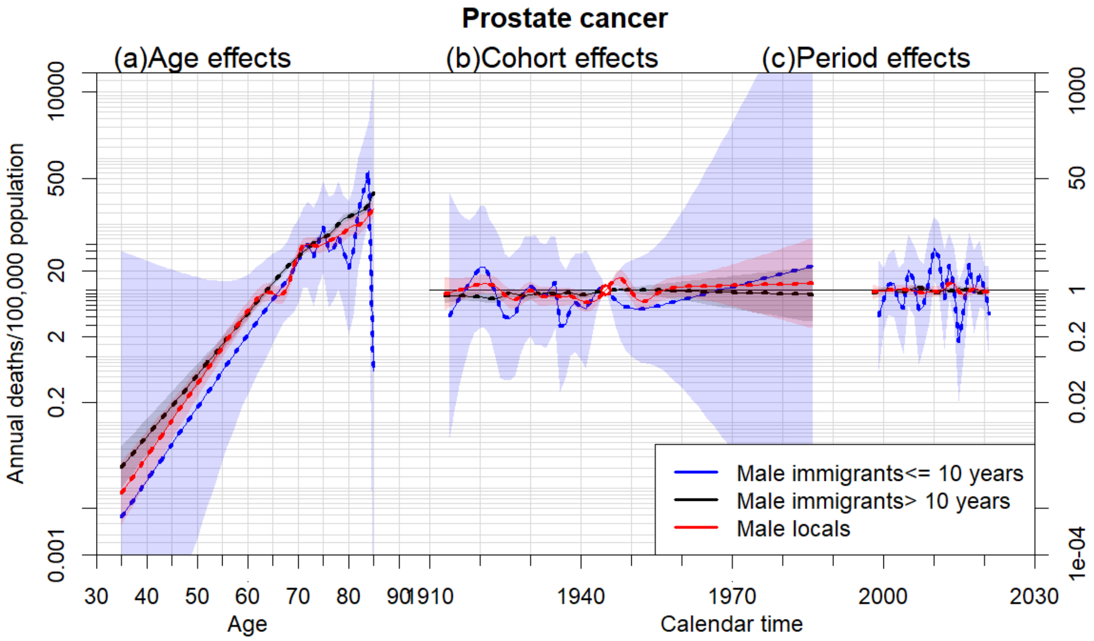


**eFigure 1(e)**. Parameter estimates of age (a), cohort (b) and period (c) effects based on an age-period-cohort model of male prostate cancer mortality rates by immigrant groups: locals, immigrants stay in Hong Kong for more than 10 years and immigrants stay in Hong Kong for less than or equal to 10 years. Age effect was assessed by mortality (left axis). Cohort and period effects were assessed by relative risk (right axis), 95% confidence intervals are shown as shaded bands.



For peer review only - http://bmjopen.bmj.com/site/about/guidelines.xhtml

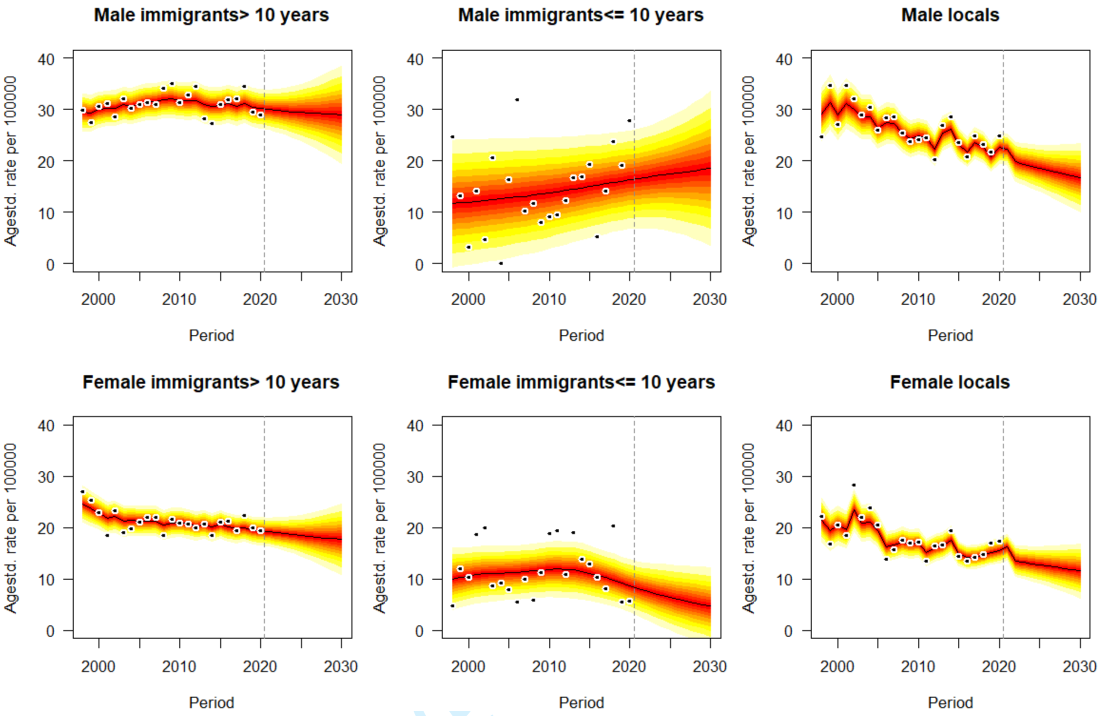


**eFigure 2(a).** Projections of colon cancer mortality rates by gender and immigrant status from 2022 to 2030. Observations are shown as dots with the predictive distribution between the 5% and 95% quantile, whereby each lighter shade of red represents an additional 10% predictive CI. The predictive mean is shown as black solid line and the vertical dashed line indicates where prediction started.

For peer review only



For peer review only - http://bmjopen.bmj.com/site/about/guidelines.xhtml

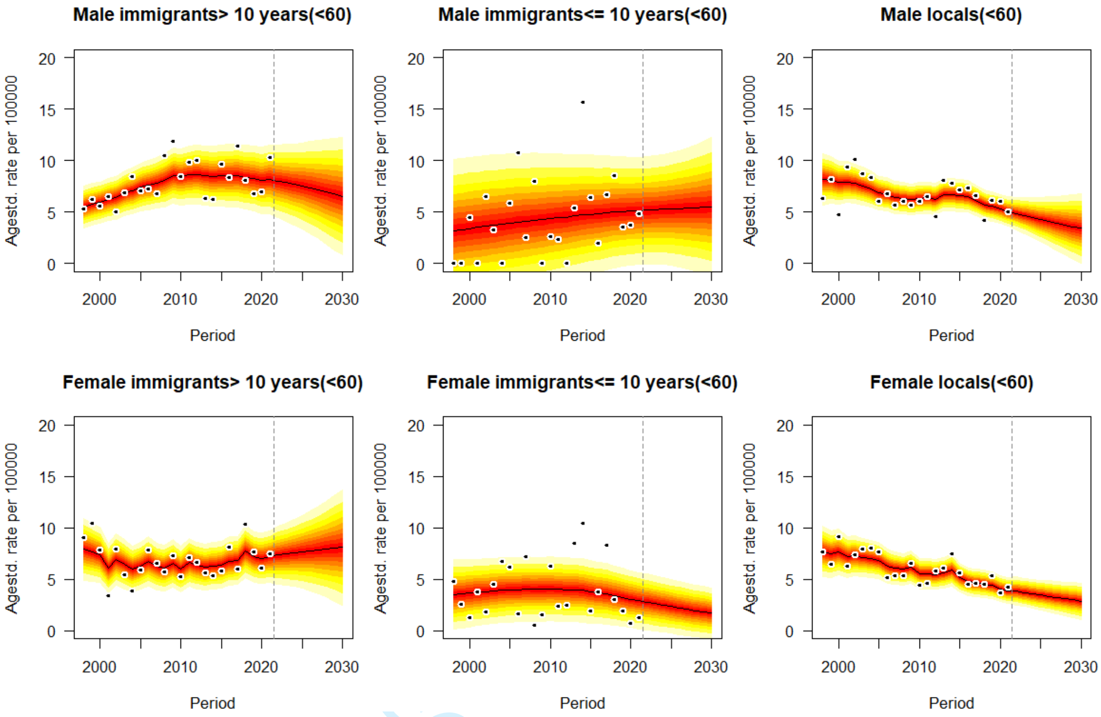


**eFigure 2(b).** Projections of colon cancer mortality rates for the population younger than 60 by gender and immigrant status from 2022 to 2030. Observations are shown as dots with the predictive distribution between the 5% and 95% quantile, whereby each lighter shade of red represents an additional 10% predictive CI. The predictive mean is shown as black solid line and the vertical dashed line indicates where prediction started.



For peer review only - http://bmjopen.bmj.com/site/about/guidelines.xhtml

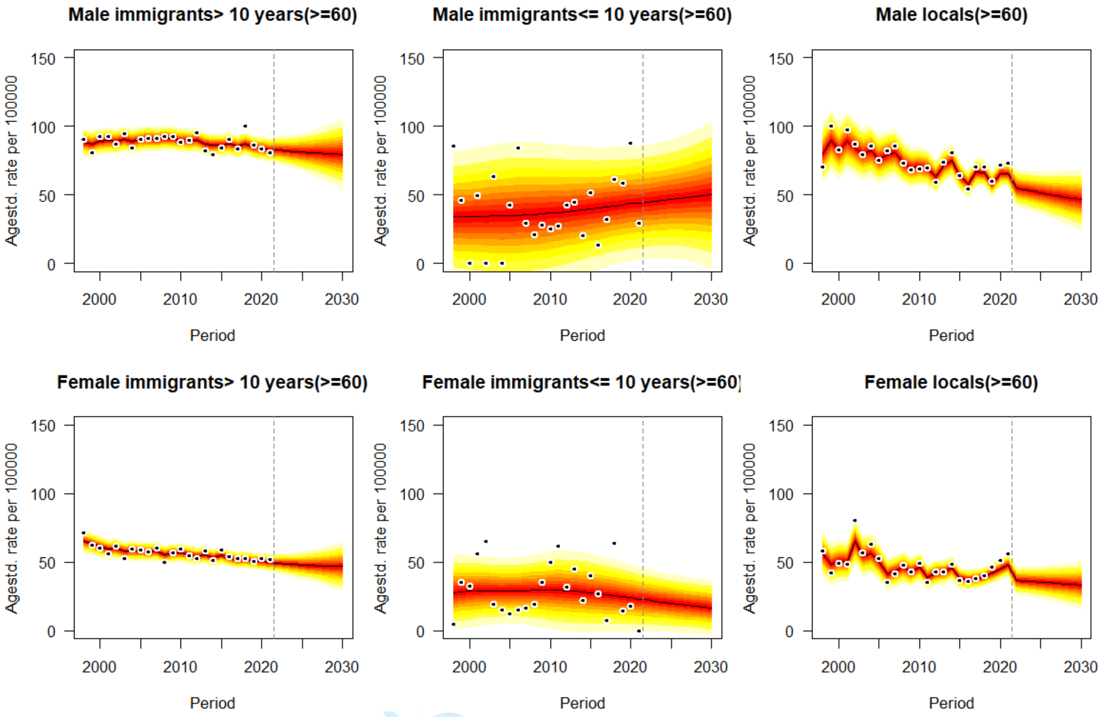


**eFigure 2(c).** Projections of colon cancer mortality rates for the population older than or equal to 60 by gender and immigrant status from 2022 to 2030. Observations are shown as dots with the predictive distribution between the 5% and 95% quantile, whereby each lighter shade of red represents an additional 10% predictive CI. The predictive mean is shown as black solid line and the vertical dashed line indicates where prediction started.



For peer review only - http://bmjopen.bmj.com/site/about/guidelines.xhtml

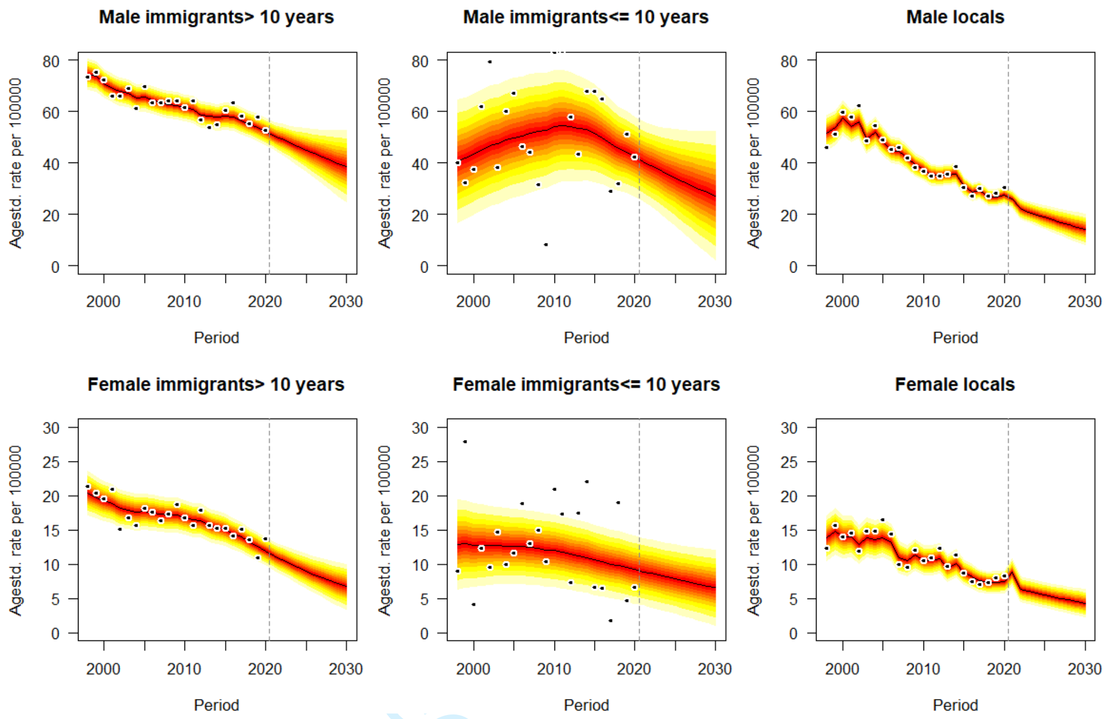


**eFigure 3(a).** Projections of liver cancer mortality rates by gender and immigrant status from 2022 to 2030. Observations are shown as dots with the predictive distribution between the 5% and 95% quantile, whereby each lighter shade of red represents an additional 10% predictive CI. The predictive mean is shown as black solid line and the vertical dashed line indicates where prediction started.



For peer review only - http://bmjopen.bmj.com/site/about/guidelines.xhtml

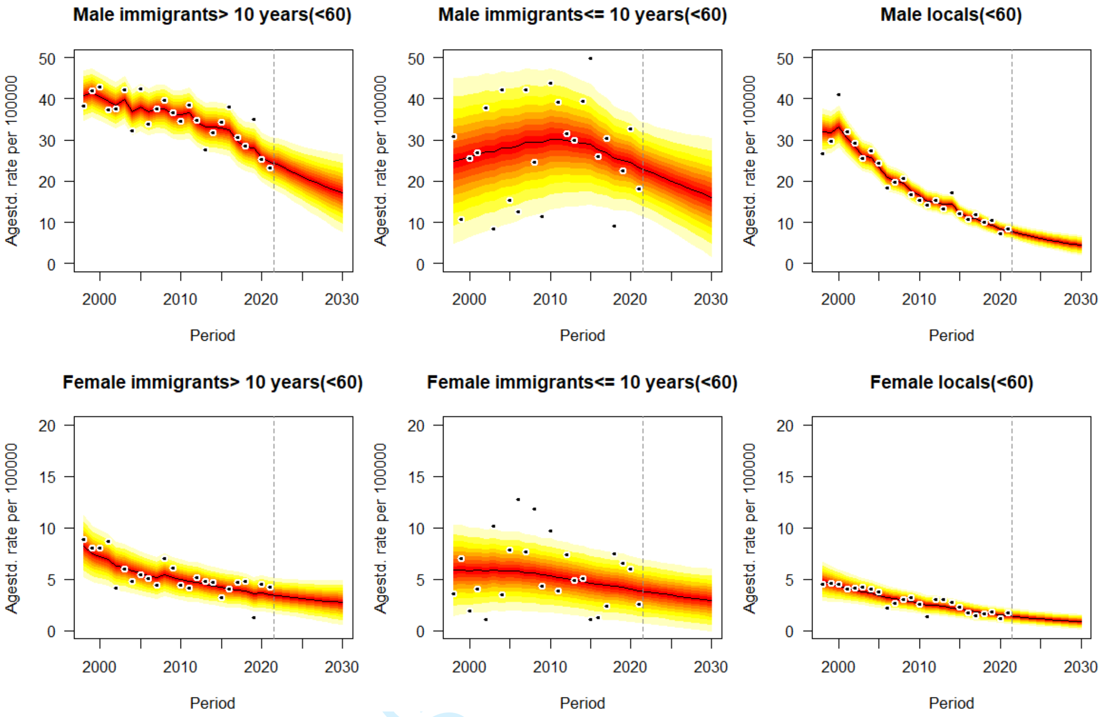


**eFigure 3(b).** Projections of liver cancer mortality rates for the population younger than 60 by gender and immigrant status from 2022 to 2030. Observations are shown as dots with the predictive distribution between the 5% and 95% quantile, whereby each lighter shade of red represents an additional 10% predictive CI. The predictive mean is shown as black solid line and the vertical dashed line indicates where prediction started.



For peer review only - http://bmjopen.bmj.com/site/about/guidelines.xhtml

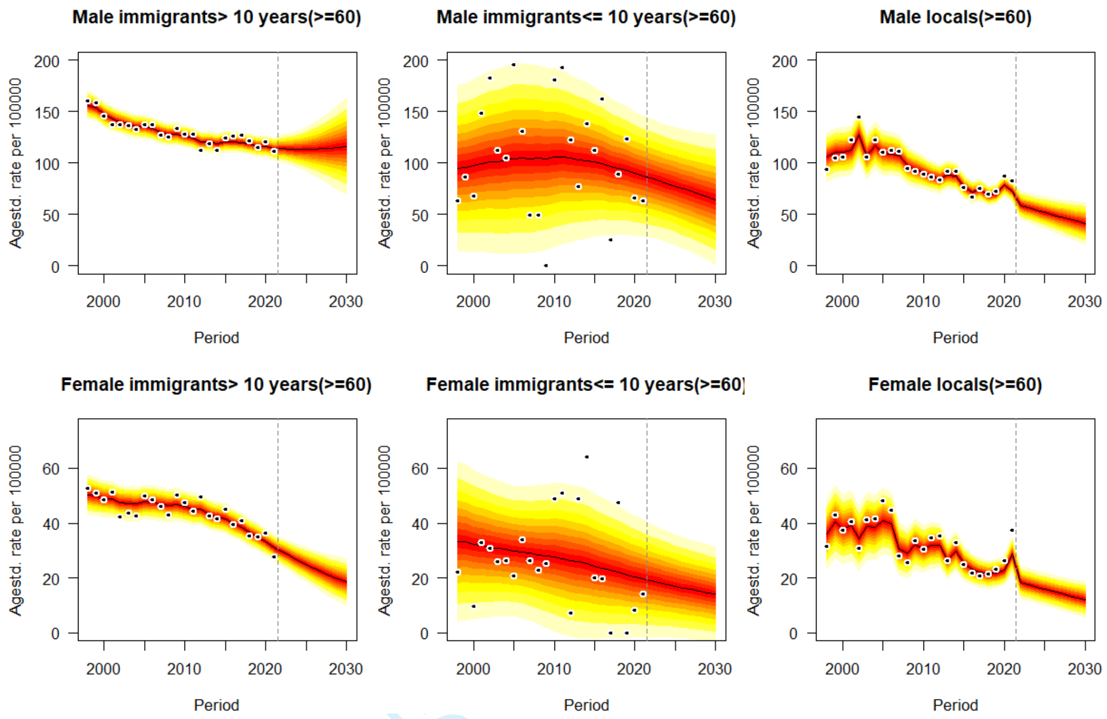


**eFigure 3(c).** Projections of liver cancer mortality rates for the population older than or equal to 60 by gender and immigrant status from 2022 to 2030. Observations are shown as dots with the predictive distribution between the 5% and 95% quantile, whereby each lighter shade of red represents an additional 10% predictive CI. The predictive mean is shown as black solid line and the vertical dashed line indicates where prediction started.



For peer review only - http://bmjopen.bmj.com/site/about/guidelines.xhtml

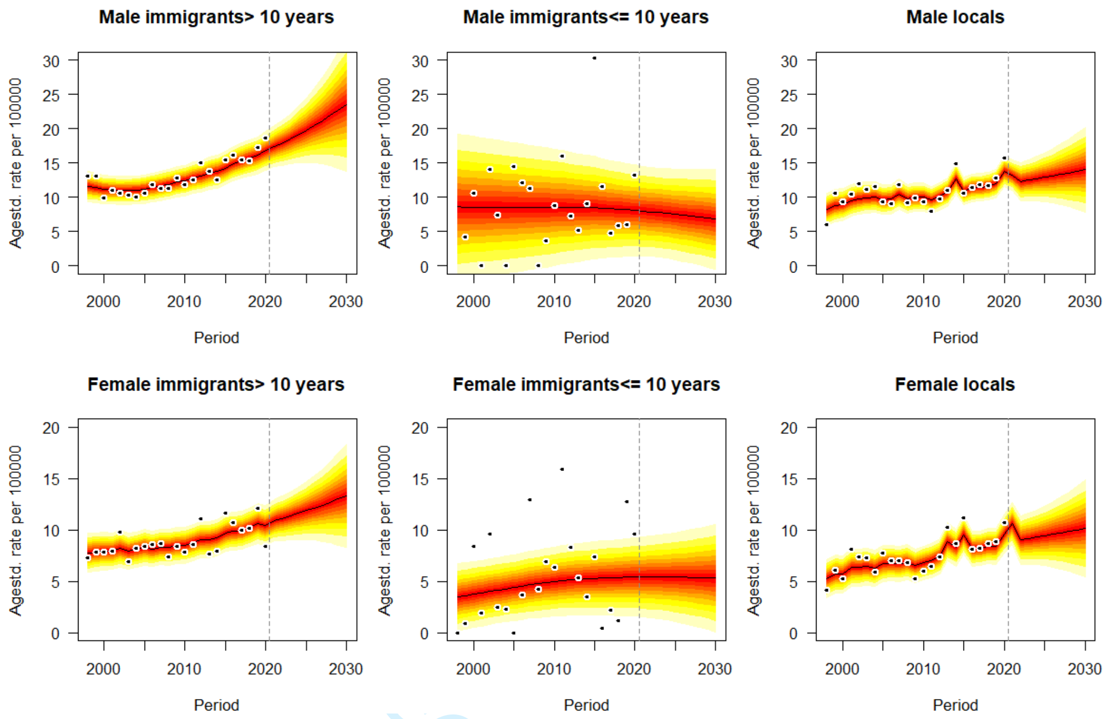


**eFigure 4(a).** Projections of pancreatic cancer mortality rates by gender and immigrant status from 2022 to 2030. Observations are shown as dots with the predictive distribution between the 5% and 95% quantile, whereby each lighter shade of red represents an additional 10% predictive CI. The predictive mean is shown as black solid line and the vertical dashed line indicates where prediction started.



For peer review only - http://bmjopen.bmj.com/site/about/guidelines.xhtml

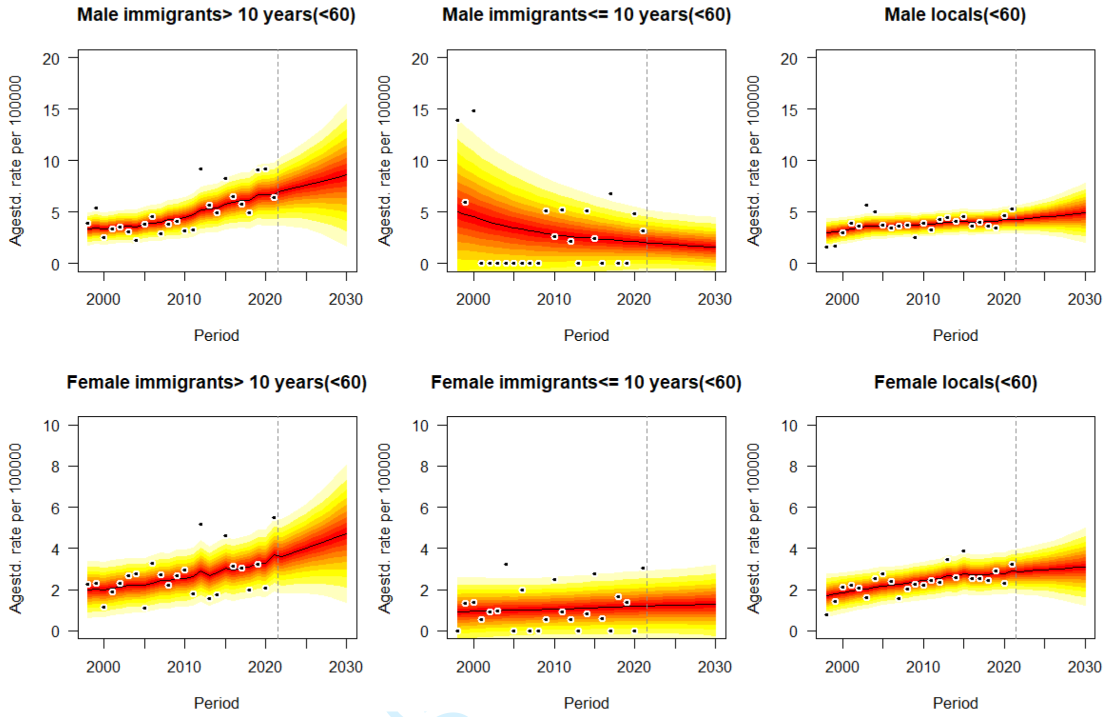


**eFigure 4(b).** Projections of pancreatic cancer mortality rates for the population younger than 60 by gender and immigrant status from 2022 to 2030. Observations are shown as dots with the predictive distribution between the 5% and 95% quantile, whereby each lighter shade of red represents an additional 10% predictive CI. The predictive mean is shown as black solid line and the vertical dashed line indicates where prediction started.



For peer review only - http://bmjopen.bmj.com/site/about/guidelines.xhtml

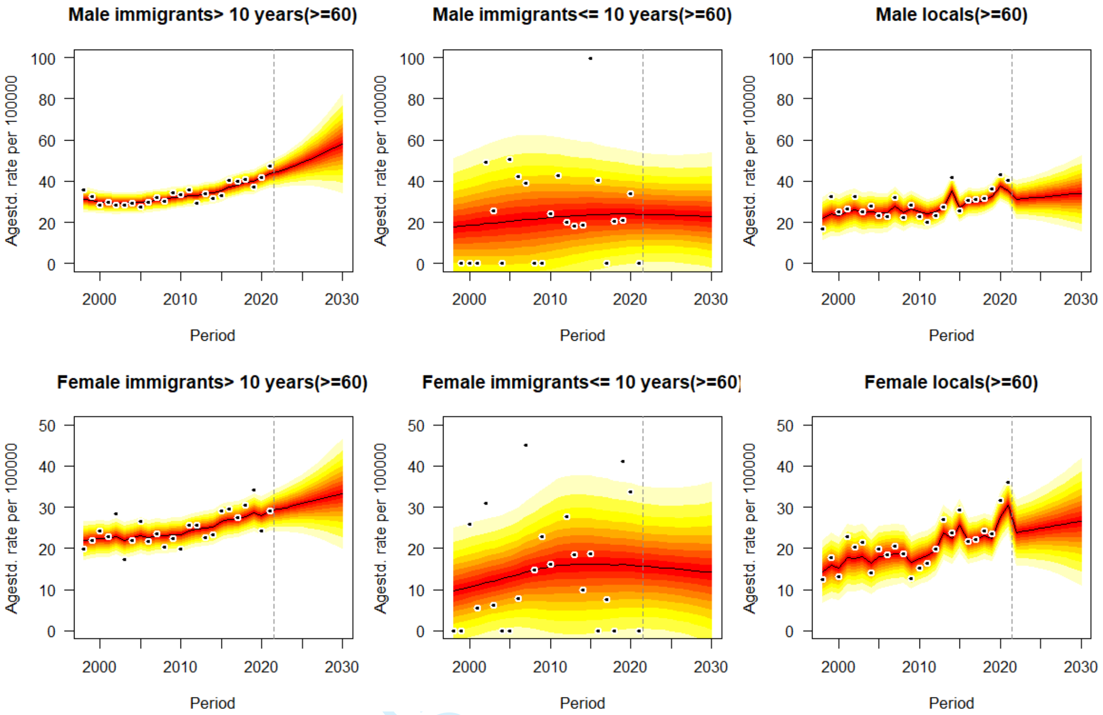


**eFigure 4(c).** Projections of pancreatic cancer mortality rates for the population older than or equal to 60 by gender and immigrant status from 2022 to 2030. Observations are shown as dots with the predictive distribution between the 5% and 95% quantile, whereby each lighter shade of red represents an additional 10% predictive CI. The predictive mean is shown as black solid line and the vertical dashed line indicates where prediction started.

For peer review only

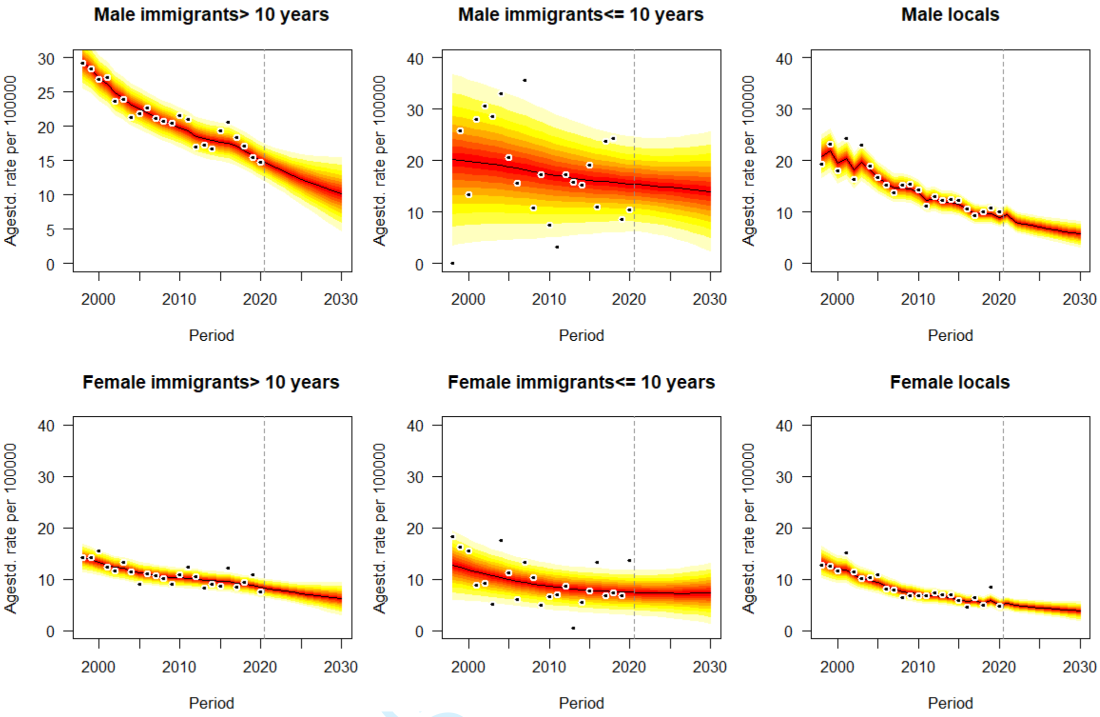


**eFigure 5(a).** Projections of stomach cancer mortality rates by gender and immigrant status from 2022 to 2030. Observations are shown as dots with the predictive distribution between the 5% and 95% quantile, whereby each lighter shade of red represents an additional 10% predictive CI. The predictive mean is shown as black solid line and the vertical dashed line indicates where prediction started.



For peer review only - http://bmjopen.bmj.com/site/about/guidelines.xhtml

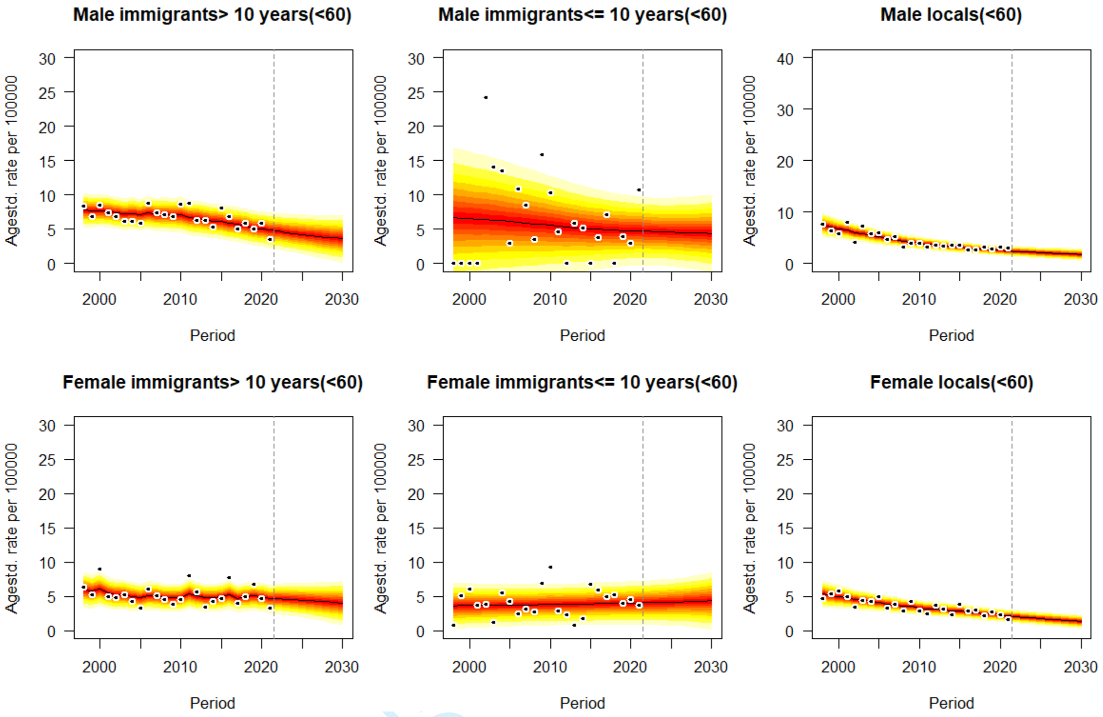


**eFigure 5(b).** Projections of stomach cancer mortality rates for the population younger than 60 by gender and immigrant status from 2022 to 2030. Observations are shown as dots with the predictive distribution between the 5% and 95% quantile, whereby each lighter shade of red represents an additional 10% predictive CI. The predictive mean is shown as black solid line and the vertical dashed line indicates where prediction started.



For peer review only - http://bmjopen.bmj.com/site/about/guidelines.xhtml

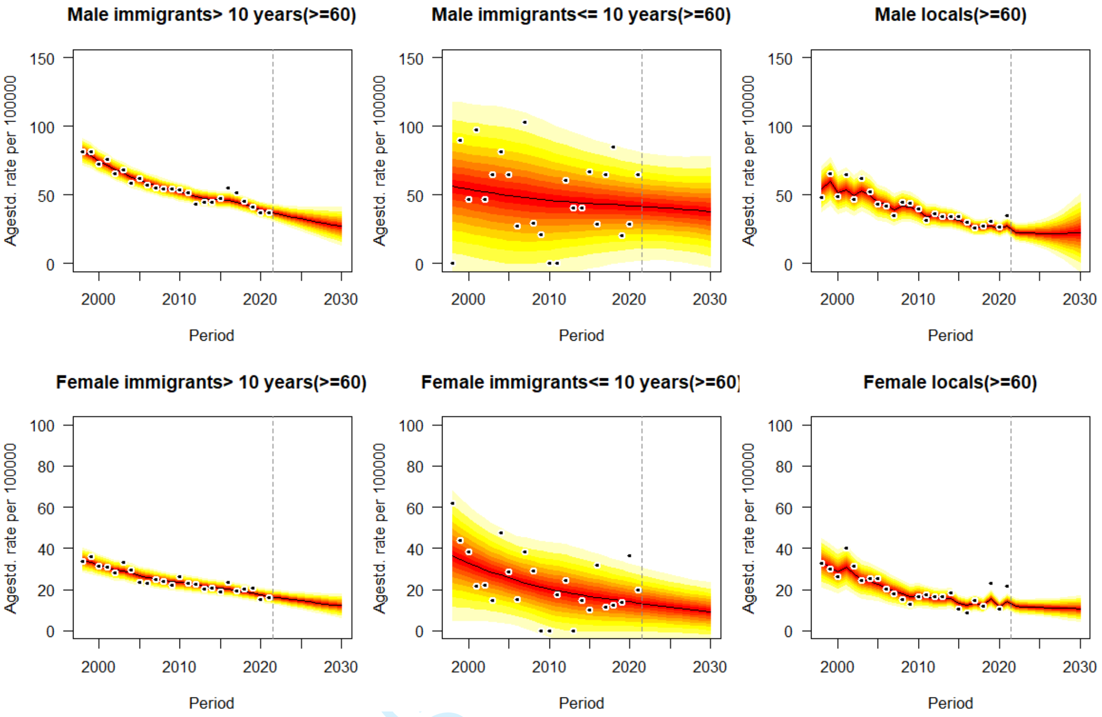


**eFigure 5(c).** Projections of stomach cancer mortality rates for the population older than or equal to 60 by gender and immigrant status from 2022 to 2030. Observations are shown as dots with the predictive distribution between the 5% and 95% quantile, whereby each lighter shade of red represents an additional 10% predictive CI. The predictive mean is shown as black solid line and the vertical dashed line indicates where prediction started.

review only



For peer review only - http://bmjopen.bmj.com/site/about/guidelines.xhtml

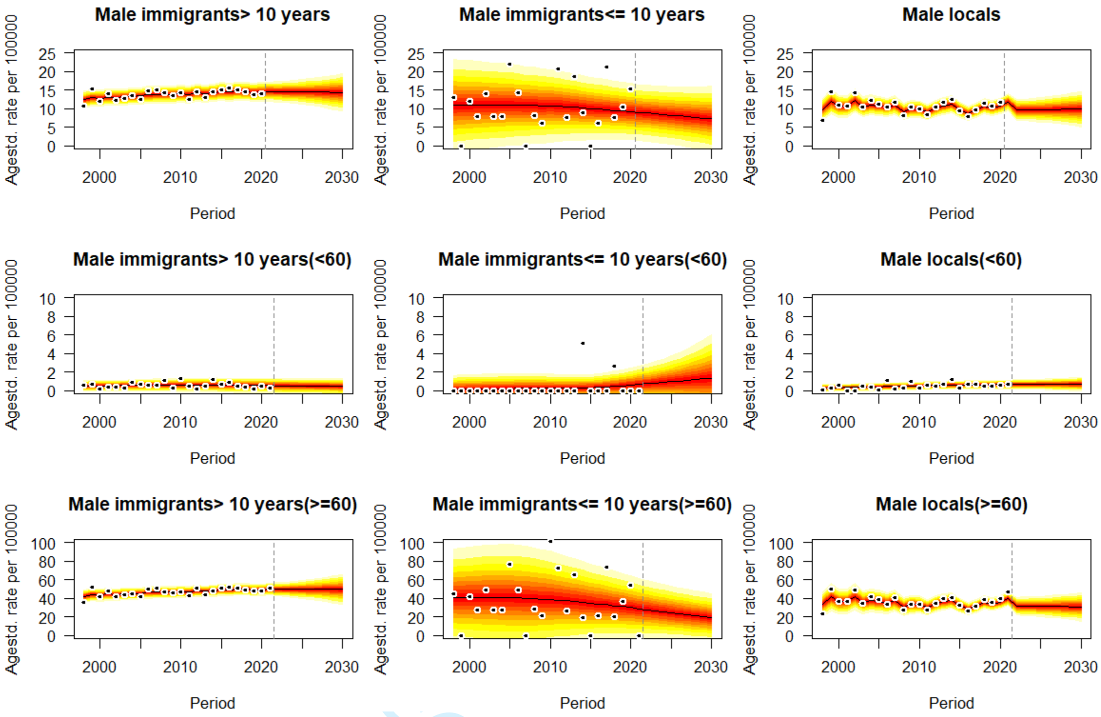


**eFigure 6.** Projections of prostate cancer mortality rates for males by immigrant status and age groups (less than, greater than or equal to 60 years old) from 2022 to 2030. Observations are shown as dots with the predictive distribution between the 5% and 95% quantile, whereby each lighter shade of red represents an additional 10% predictive CI. The predictive mean is shown as black solid line and the vertical dashed line indicates where prediction started.



For peer review only - http://bmjopen.bmj.com/site/about/guidelines.xhtml

| Predictive mean of age-standardized mortality rates of lung cancer per 100,000 population | | | | | | | | | |
|---|---|---|---|---|---|---|---|---|---|
| **Year** | **2022** | **2023** | **2024** | **2025** | **2026** | **2027** | **2028** | **2029** | **2030** |
| **Female immigrants >10** | 41.34 (1.86) | 40.58 (2.27) | 39.87 (2.75) | 39.19 (3.28) | 38.53 (3.86) | 37.89 (4.46) | 37.26 (5.09) | 36.65 (5.74) | 36.04 (6.4) |
| **Female immigrants ≤ 10** | 22.22 (4.67) | 20.56 (5.38) | 19.01 (6.10) | 17.57 (6.80) | 16.24 (7.45) | 15.00 (8.04) | 13.85 (8.56) | 12.79 (9.01) | 11.81 (9.39) |
| **Female locals** | 30.22 (3.54) | 30.63 (4.77) | 31.05 (6.38) | 31.48 (8.29) | 31.9 (10.47) | 32.32 (12.87) | 32.73 (15.48) | 33.15 (18.31) | 33.55 (21.33) |
| **Male immigrants >10** | 100.18 (4.18) | 97.18 (5.33) | 94.34 (6.72) | 91.71 (8.24) | 89.15 (9.84) | 86.66 (11.47) | 84.19 (13.11) | 81.81 (14.74) | 79.55 (16.37) |
| **Male immigrants ≤10** | 79.90 (10.41) | 79.81 (11.82) | 79.72 (13.42) | 79.62 (15.19) | 79.50 (17.09) | 79.32 (19.09) | 79.08 (21.18) | 78.78 (23.32) | 78.41 (25.53) |
| **Male locals** | 52.27 (4.86) | 50.83 (5.39) | 49.56 (6.13) | 48.18 (6.97) | 46.64 (7.84) | 45.13 (8.76) | 43.83 (9.76) | 42.67 (10.8) | 41.43 (11.8) |
| **Female immigrants>10(<60y)** | 14.51 (1.50) | 13.90 (1.76) | 13.29 (2.04) | 12.71 (2.33) | 12.13 (2.62) | 11.57 (2.91) | 11.02 (3.18) | 10.49 (3.43) | 9.98(3.68) |
| **Female immigrants ≤ 10(<60y)** | 7.79 (1.95) | 7.18(2.23) | 6.62(2.53) | 6.10(2.81) | 5.63(3.08) | 5.19(3.32) | 4.79(3.53) | 4.42(3.72) | 4.09(3.88) |
| **Female locals(<60y)** | 9.48 (0.89) | 9.17(1.02) | 8.87(1.16) | 8.57(1.32) | 8.27(1.49) | 7.97(1.65) | 7.68(1.82) | 7.38(1.98) | 7.09(2.13) |
| **Male immigrants>10(<60y)** | 26.36 (3.58) | 24.96 (3.94) | 23.64 (4.35) | 22.38 (4.79) | 21.17 (5.23) | 20.03 (5.67) | 18.96 (6.10) | 17.96 (6.51) | 17.03 (6.90) |
| **Male immigrants ≤ 10(<60y)** | 13.38 (3.71) | 12.02 (4.17) | 10.79 (4.59) | 9.68 (4.95) | 8.69 (5.24) | 7.79 (5.46) | 6.98 (5.61) | 6.25 (5.69) | 5.59 (5.72) |
| **Male locals(<60y)** | 14.45 (1.15) | 14.03 (1.29) | 13.61 (1.46) | 13.14 (1.64) | 12.65 (1.82) | 12.13 (2.01) | 11.55 (2.17) | 10.93 (2.31) | 10.26 (2.43) |
| **Female immigrants >10(≥ 60y)** | 107.21 (5.17) | 106.26 (6.24) | 105.52 (7.54) | 104.94 (9.04) | 104.51 (10.72) | 104.21 (12.57) | 104.07 (14.61) | 104.06 (16.78) | 104.16 (19.14) |
| **Female immigrants ≤ 10(≥60y)** | 63.84 (15.72) | 59.88 (17.50) | 56.14 (19.31) | 52.60 (21.03) | 49.27 (22.66) | 46.14 (24.16) | 43.20 (25.52) | 40.44 (26.74) | 37.85 (27.81) |
| **Female locals(≥60y)** | 76.53 (10.11) | 76.22 (10.85) | 75.94 (11.79) | 75.69 (12.94) | 75.49 (14.28) | 75.32 (15.80) | 75.19 (17.48) | 75.10 (19.33) | 75.03 (21.32) |
| **Male immigrants>10(≥60y)** | 289.8 (11.7) | 286.6 (15.19) | 284.28 (19.51) | 282.78 (24.49) | 281.99 (30.07) | 281.88 (36.31) | 282.31 (43.15) | 283.37 (50.66) | 285.03 (58.86) |
| **Male immigrants ≤ 10(≥60y)** | 247.01 (36.85) | 251.24 (42.94) | 255.62 (50.06) | 260.14 (58.14) | 264.82 (67.14) | 269.61 (77.01) | 274.52 (87.75) | 279.55 (99.34) | 284.69 (111.81) |
| **Male locals(≥60y)** | 146.29 (18.46) | 143.54 (20.58) | 141.84 (23.97) | 140.07 (28.24) | 138.14 (33.39) | 136.65 (39.82) | 136.49 (47.87) | 137.24 (57.47) | 138.26 (68.52) |

**eTable 1.** Predictive means and standard deviations (in brackets) of age-standardized mortality rates of lung cancer per 100,000 population for each gender, age group (less than, greater or equal to 60 years old) and immigrant status from 2022 to 2030.



For peer review only - http://bmjopen.bmj.com/site/about/guidelines.xhtml

For peer review only

| Predictive mean of age-standardized mortality rates of colon cancer per 100,000 population | | | | | | | | | |
|---|---|---|---|---|---|---|---|---|---|
| Year | 2022 | 2023 | 2024 | 2025 | 2026 | 2027 | 2028 | 2029 | 2030 |
| Female immigrants >10 | 18.95 (1.13) | 18.77 (1.37) | 18.59 (1.66) | 18.42 (1.98) | 18.27 (2.33) | 18.12 (2.71) | 17.98 (3.11) | 17.85 (3.53) | 17.73 (3.96) |
| Female immigrants ≤ 10 | 7.70 (2.51) | 7.25 (2.81) | 6.82 (3.11) | 6.42 (3.37) | 6.03 (3.61) | 5.67 (3.83) | 5.33 (4.01) | 5.01 (4.17) | 4.71 (4.31) |
| Female locals | 13.47 (1.61) | 13.24 (1.72) | 13.01 (1.87) | 12.77 (2.04) | 12.53 (2.24) | 12.29 (2.46) | 12.06 (2.68) | 11.82 (2.92) | 11.59 (3.16) |
| Male immigrants >10 | 29.82 (1.46) | 29.66 (1.79) | 29.52 (2.19) | 29.41 (2.63) | 29.30 (3.11) | 29.21 (3.64) | 29.14 (4.19) | 29.06 (4.78) | 28.98 (5.39) |
| Male immigrants ≤10 | 16.77 (3.77) | 17.02 (4.18) | 17.23 (4.64) | 17.45 (5.14) | 17.67 (5.69) | 17.88 (6.27) | 18.09 (6.91) | 18.31 (7.56) | 18.50 (8.26) |
| Male locals | 19.81 (2.07) | 19.39 (2.22) | 18.97 (2.42) | 18.57 (2.61) | 18.18 (2.85) | 17.81 (3.12) | 17.43 (3.40) | 17.06 (3.71) | 16.71 (4.03) |
| Female immigrants >10(<60y) | 7.36 (1.12) | 7.46 (1.28) | 7.56 (1.46) | 7.65 (1.68) | 7.74 (1.92) | 7.83 (2.19) | 7.92 (2.48) | 8.01 (2.79) | 8.09 (3.13) |
| Female immigrants ≤ 10(<60y) | 2.82 (0.86) | 2.65 (0.91) | 2.51 (0.97) | 2.36 (1.02) | 2.22 (1.07) | 2.08 (1.11) | 1.95 (1.14) | 1.83 (1.18) | 1.72 (1.22) |
| Female locals(<60y) | 3.87 (0.50) | 3.73 (0.54) | 3.61 (0.59) | 3.47 (0.65) | 3.34 (0.70) | 3.22 (0.76) | 3.11 (0.82) | 2.99 (0.88) | 2.88 (0.94) |
| Male immigrants >10(<60y) | 7.98(1.17) | 7.85 (1.38) | 7.71 (1.60) | 7.54(1.83) | 7.36(2.08) | 7.17(2.32) | 6.97(2.57) | 6.76(2.81) | 6.55(3.05) |
| Male immigrants ≤ 10(<60y) | 5.18 (1.58) | 5.22 (1.75) | 5.26 (1.93) | 5.30(2.14) | 5.34(2.36) | 5.38(2.59) | 5.43(2.84) | 5.47(3.11) | 5.51(3.38) |
| Male locals(<60y) | 4.88 (0.63) | 4.66 (0.79) | 4.46 (0.96) | 4.26(1.13) | 4.08(1.31) | 3.91(1.48) | 3.73(1.65) | 3.57(1.82) | 3.42(1.97) |
| Female immigrants >10(≥60y) | 49.21 (2.99) | 48.70 (3.56) | 48.26 (4.26) | 47.87 (5.05) | 47.54 (5.94) | 47.26 (6.90) | 47.05 (7.94) | 46.91 (9.06) | 46.81 (10.26) |
| Female immigrants ≤ 10(≥60y) | 22.44 (6.56) | 21.69 (6.96) | 20.95 (7.38) | 20.23 (7.80) | 19.52 (8.23) | 18.84 (8.66) | 18.17 (9.08) | 17.51 (9.49) | 16.86 (9.90) |
| Female locals(≥60y) | 36.69 (5.74) | 36.29 (6.06) | 35.87 (6.46) | 35.46 (6.95) | 35.04(7.5) | 34.61 (8.12) | 34.19 (8.79) | 33.77 (9.51) | 33.34 (10.27) |
| Male immigrants >10(≥60y) | 82.72 (4.09) | 82.16 (4.95) | 81.64 (5.97) | 81.19 (7.12) | 80.81 (8.39) | 80.47 (9.77) | 80.15 (11.24) | 79.85 (12.81) | 79.56 (14.45) |
| Male immigrants ≤ 10(≥60y) | 44.93 (13.09) | 45.62 (14.52) | 46.30 (16.09) | 46.96 (17.80) | 47.61 (19.64) | 48.25 (21.62) | 48.88 (23.73) | 49.51 (25.97) | 50.13 (28.34) |
| Male locals(≥60y) | 54.89 (7.65) | 53.75 (8.03) | 52.63 (8.52) | 51.54 (9.12) | 50.47 (9.8) | 49.43 (10.55) | 48.42 (11.37) | 47.42 (12.25) | 46.44 (13.16) |

**eTable 2.** Predictive means and standard deviations (in brackets) of age-standardized mortality rates of colon cancer per 100,000 population for each gender, age group (less than, greater or equal to 60 years old) and immigrant status from 2022 to 2030.

For peer review only



For peer review only - http://bmjopen.bmj.com/site/about/guidelines.xhtml

| Predictive mean of age-standardized mortality rates of liver cancer per 100,000 population | | | | | | | | | |
|---|---|---|---|---|---|---|---|---|---|
| **Year** | **2022** | **2023** | **2024** | **2025** | **2026** | **2027** | **2028** | **2029** | **2030** |
| **Female immigrants >10** | 10.68 (0.71) | 10.09 (0.85) | 9.54 (1.01) | 9.01 (1.16) | 8.50 (1.31) | 8.02(1.45) | 7.57(1.59) | 7.14(1.72) | 6.74(1.83) |
| **Female immigrants ≤10** | 8.66 (1.82) | 8.38 (1.95) | 8.11 (2.08) | 7.84 (2.22) | 7.58 (2.36) | 7.32(2.49) | 7.07(2.63) | 6.82(2.76) | 6.58(2.88) |
| **Female locals** | 6.36 (0.88) | 6.08 (0.90) | 5.81 (0.93) | 5.53 (0.97) | 5.26 (1.01) | 5.01(1.06) | 4.77(1.11) | 4.53(1.15) | 4.3(1.21) |
| **Male immigrants >10** | 49.22 (2.36) | 47.76 (2.93) | 46.35 (3.59) | 45.01 (4.31) | 43.67 (5.05) | 42.37 (5.81) | 41.1(6.56) | 39.89 (7.33) | 38.71 (8.08) |
| **Male immigrants ≤10** | 39.03 (6.49) | 37.39 (7.47) | 35.81 (8.51) | 34.26 (9.58) | 32.76 (10.63) | 31.31 (11.65) | 29.91 (12.62) | 28.56 (13.54) | 27.25 (14.40) |
| **Male locals** | 22.16 (2.09) | 21.02 (2.22) | 19.91 (2.39) | 18.85 (2.58) | 17.83 (2.79) | 16.85 (3.03) | 15.92 (3.21) | 15.03 (3.40) | 14.18 (3.59) |
| **Female immigrants >10(<60y)** | 3.39 (0.52) | 3.29 (0.57) | 3.20 (0.63) | 3.12 (0.69) | 3.04 (0.75) | 2.96(0.82) | 2.89(0.89) | 2.82(0.96) | 2.75(1.03) |
| **Female immigrants ≤ 10(<60y)** | 3.81 (0.91) | 3.69 (0.96) | 3.57 (1.02) | 3.46 (1.08) | 3.36 (1.15) | 3.25(1.22) | 3.15(1.29) | 3.06(1.36) | 2.97(1.43) |
| **Female locals(<60y)** | 1.37 (0.2) | 1.29 (0.21) | 1.22 (0.23) | 1.16 (0.24) | 1.10 (0.26) | 1.04(0.27) | 0.99(0.29) | 0.94(0.30) | 0.89(0.31) |
| **Male immigrants >10(<60y)** | 24.04 (2.35) | 23.02 (2.63) | 22.05 (2.94) | 21.13 (3.27) | 20.25 (3.61) | 19.41 (3.95) | 18.62 (4.30) | 17.86 (4.64) | 17.14 (4.98) |
| **Male immigrants ≤ 10(<60y)** | 22.56 (3.96) | 21.71 (4.44) | 20.87 (4.94) | 20.04 (5.45) | 19.22 (5.95) | 18.42 (6.45) | 17.63 (6.91) | 16.86 (7.36) | 16.11 (7.78) |
| **Male locals(<60y)** | 7.47 (0.74) | 6.97 (0.79) | 6.52 (0.86) | 6.11 (0.93) | 5.73 (1.01) | 5.38(1.08) | 5.04(1.15) | 4.73(1.21) | 4.44(1.27) |
| **Female immigrants >10(≥ 60y)** | 29.63 (2.01) | 27.99 (2.36) | 26.42 (2.75) | 24.92 (3.14) | 23.49 (3.52) | 22.13 (3.88) | 20.85 (4.23) | 19.64 (4.55) | 18.50 (4.85) |
| **Female immigrants ≤10(≥ 60y)** | 19.08 (5.81) | 18.38 (6.14) | 17.71 (6.48) | 17.03 (6.83) | 16.39 (7.16) | 15.76 (7.49) | 15.16 (7.80) | 14.57 (8.11) | 14.01 (8.39) |
| **Female locals(≥60y)** | 18.41 (3.23) | 17.55 (3.26) | 16.72 (3.32) | 15.91 (3.40) | 15.11 (3.49) | 14.34 (3.59) | 13.59 (3.69) | 12.87 (3.81) | 12.17 (3.93) |
| **Male immigrants >10(≥ 60y)** | 113.96 (5.95) | 113.43 (7.65) | 113.17 (9.70) | 113.16 (12.04) | 113.37 (14.66) | 113.79 (17.56) | 114.39 (20.73) | 115.19 (24.18) | 116.17 (27.91) |
| **Male immigrants ≤10(≥ 60y)** | 85.14 (18.85) | 82.59 (20.6) | 80.02 (22.44) | 77.42 (24.34) | 74.83 (26.24) | 72.23 (28.12) | 69.64 (29.94) | 67.07 (31.70) | 64.52 (33.38) |
| **Male locals(≥60y)** | 58.95 (7.91) | 56.51 (8.20) | 54.14 (8.61) | 51.84 (9.12) | 49.61 (9.70) | 47.46 (10.33) | 45.38 (11.01) | 43.38 (11.68) | 41.45(12.36) |

**eTable 3.** Predictive means and standard deviations (in brackets) of age-standardized mortality rates of liver cancer per 100,000 population for each gender, age group (less than, greater or equal to 60 years old) and immigrant status from 2022 to 2030.

For peer review only



For peer review only - http://bmjopen.bmj.com/site/about/guidelines.xhtml

| Predictive mean of age-standardized mortality rates of pancreatic cancer per 100,000 population | | | | | | | | | |
|---|---|---|---|---|---|---|---|---|---|
| **Year** | **2022** | **2023** | **2024** | **2025** | **2026** | **2027** | **2028** | **2029** | **2030** |
| **Female immigrants >10** | 11.11 (0.75) | 11.36 (0.91) | 11.61 (1.09) | 11.87 (1.31) | 12.14 (1.56) | 12.42 (1.84) | 12.71 (2.15) | 13.01 (2.48) | 13.3(2.85) |
| **Female immigrants ≤ 10** | 5.44 (1.56) | 5.44 (1.69) | 5.43(1.84) | 5.42(1.99) | 5.41(2.15) | 5.39(2.32) | 5.36(2.49) | 5.34(2.66) | 5.31(2.84) |
| **Female locals** | 9.01 (1.22) | 9.15 (1.34) | 9.29(1.48) | 9.43(1.64) | 9.57(1.83) | 9.71(2.05) | 9.85(2.28) | 9.99(2.54) | 10.14 (2.83) |
| **Male immigrants >10** | 17.87 (1.19) | 18.48 (1.49) | 19.11 (1.87) | 19.78 (2.32) | 20.47 (2.83) | 21.18 (3.42) | 21.92 (4.07) | 22.69 (4.81) | 23.49 (5.61) |
| **Male immigrants ≤10** | 7.87 (2.37) | 7.76 (2.53) | 7.64(2.70) | 7.51(2.87) | 7.38(3.05) | 7.24(3.23) | 7.09(3.41) | 6.95(3.58) | 6.81(3.75) |
| **Male locals** | 12.29 (1.49) | 12.49 (1.64) | 12.69 (1.83) | 12.91 (2.06) | 13.11 (2.33) | 13.33 (2.63) | 13.55 (2.97) | 13.78 (3.34) | 14.02 (3.74) |
| **Female immigrants >10(<60y)** | 3.62 (0.57) | 3.74 (0.66) | 3.87(0.77) | 4.01(0.89) | 4.14(1.02) | 4.28(1.18) | 4.42(1.34) | 4.57(1.53) | 4.72(1.73) |
| **Female immigrants ≤ 10(<60y)** | 1.21 (0.48) | 1.22 (0.52) | 1.23(0.56) | 1.24(0.61) | 1.25(0.66) | 1.26(0.71) | 1.26(0.77) | 1.27(0.83) | 1.28(0.89) |
| **Female locals(<60y)** | 2.88 (0.36) | 2.91 (0.41) | 2.93(0.48) | 2.96(0.55) | 2.99(0.63) | 3.02(0.71) | 3.04(0.81) | 3.07(0.90) | 3.10(1.01) |
| **Male immigrants >10(<60y)** | 7.05 (1.11) | 7.24 (1.32) | 7.43(1.56) | 7.62(1.84) | 7.82(2.16) | 8.01(2.50) | 8.21(2.88) | 8.40(3.30) | 8.61(3.75) |
| **Male immigrants ≤ 10(<60y)** | 2.01 (0.85) | 1.95 (0.91) | 1.9(0.94) | 1.84(0.99) | 1.79(1.04) | 1.74(1.09) | 1.69(1.14) | 1.64(1.19) | 1.60(1.24) |
| **Male locals(<60y)** | 4.33 (0.48) | 4.41 (0.57) | 4.46(0.68) | 4.53(0.81) | 4.61(0.94) | 4.69(1.09) | 4.77(1.26) | 4.85(1.44) | 4.93(1.63) |
| **Female immigrants >10(≥60y)** | 29.45 (2.11) | 29.91 (2.54) | 30.38 (3.06) | 30.85 (3.66) | 31.33 (4.33) | 31.81 (5.08) | 32.29 (5.91) | 32.78 (6.79) | 33.27 (7.74) |
| **Female immigrants ≤ 10(≥60y)** | 15.65 (6.08) | 15.49 (6.71) | 15.33 (7.36) | 15.16 (8.03) | 14.97 (8.73) | 14.79 (9.43) | 14.59 (10.14) | 14.39 (10.86) | 14.19 (11.58) |
| **Female locals(≥60y)** | 23.85 (4.46) | 24.21 (4.81) | 24.56 (5.23) | 24.91 (5.73) | 25.25 (6.30) | 25.58 (6.95) | 25.90 (7.67) | 26.22 (8.47) | 26.54 (9.34) |
| **Male immigrants >10(≥60y)** | 44.36 (3.02) | 45.85 (3.76) | 47.41 (4.69) | 49.04 (5.78) | 50.73 (7.05) | 52.48 (8.50) | 54.28 (10.13) | 56.16 (11.95) | 58.11 (13.98) |
| **Male immigrants ≤ 10(≥60y)** | 23.96 (9.01) | 23.87 (9.74) | 23.75 (10.52) | 23.61 (11.33) | 23.45 (12.17) | 23.28 (13.04) | 23.09 (13.93) | 22.89 (14.83) | 22.68 (15.75) |
| **Male locals(≥60y)** | 31.17 (5.22) | 31.55 (5.63) | 31.93 (6.14) | 32.30 (6.75) | 32.66 (7.45) | 33.01 (8.23) | 33.35 (9.11) | 33.69 (10.08) | 34.03 (11.12) |

**eTable 4.** Predictive means and standard deviations (in brackets) of age-standardized mortality rates of pancreatic cancer per 100,000 population for each gender, age group (less than, greater or equal to 60 years old) and immigrant status from 2022 to 2030.

For peer review only



For peer review only - http://bmjopen.bmj.com/site/about/guidelines.xhtml

| Predictive mean of age-standardized mortality rates of stomach cancer per 100,000 population | | | | | | | | | |
|---|---|---|---|---|---|---|---|---|---|
| Year | 2022 | 2023 | 2024 | 2025 | 2026 | 2027 | 2028 | 2029 | 2030 |
| Female immigrants >10 | 7.95 (0.62) | 7.71 (0.74) | 7.47 (0.87) | 7.25 (1.01) | 7.03 (1.15) | 6.83 (1.29) | 6.62 (1.43) | 6.43 (1.57) | 6.24 (1.71) |
| Female immigrants ≤10 | 7.36 (1.56) | 7.33 (1.69) | 7.30 (1.85) | 7.28 (2.01) | 7.27 (2.20) | 7.27 (2.40) | 7.28 (2.61) | 7.31 (2.84) | 7.33 (3.09) |
| Female locals | 4.91 (0.52) | 4.75 (0.57) | 4.61 (0.63) | 4.47 (0.71) | 4.34 (0.77) | 4.21 (0.84) | 4.08 (0.91) | 3.95 (0.99) | 3.83 (1.06) |
| Male immigrants >10 | 13.89 (0.97) | 13.34(1.21) | 12.81 (1.46) | 12.31 (1.73) | 11.83 (1.99) | 11.38 (2.26) | 10.95 (2.51) | 10.54 (2.76) | 10.15 (3.01) |
| Male immigrants ≤10 | 15.21 (3.38) | 15.07 (3.67) | 14.93 (3.98) | 14.79 (4.31) | 14.64 (4.65) | 14.51 (5.02) | 14.35 (5.39) | 14.19 (5.78) | 14.03 (6.17) |
| Male locals | 8.07 (0.99) | 7.73 (1.03) | 7.41(1.07) | 7.10 (1.13) | 6.81 (1.19) | 6.51 (1.26) | 6.23 (1.33) | 5.97 (1.39) | 5.71 (1.46) |
| Female immigrants >10(<60y) | 4.69 (0.79) | 4.62 (0.87) | 4.55 (0.96) | 4.47 (1.07) | 4.39 (1.17) | 4.31 (1.29) | 4.22 (1.41) | 4.13 (1.52) | 4.03 (1.64) |
| Female immigrants ≤ 10(<60y) | 4.08 (0.93) | 4.10 (1.03) | 4.13 (1.14) | 4.17 (1.27) | 4.21 (1.41) | 4.24 (1.55) | 4.28 (1.70) | 4.32 (1.87) | 4.36 (2.05) |
| Female locals(<60y) | 2.08 (0.27) | 1.98 (0.29) | 1.88 (0.32) | 1.79 (0.35) | 1.71 (0.37) | 1.61 (0.41) | 1.53 (0.43) | 1.44 (0.45) | 1.37 (0.47) |
| Male immigrants >10(<60y) | 4.71 (0.79) | 4.55 (0.89) | 4.41 (0.99) | 4.25 (1.10) | 4.12 (1.21) | 3.98 (1.32) | 3.86 (1.43) | 3.74 (1.54) | 3.63 (1.65) |
| Male immigrants ≤ 10(<60y) | 4.70 (1.42) | 4.66 (1.55) | 4.63 (1.69) | 4.59 (1.83) | 4.55 (1.99) | 4.52 (2.15) | 4.48 (2.32) | 4.44 (2.50) | 4.41 (2.68) |
| Male locals(<60y) | 2.37 (0.29) | 2.28 (0.32) | 2.21 (0.35) | 2.12 (0.38) | 2.04 (0.42) | 1.97 (0.45) | 1.91 (0.49) | 1.83 (0.52) | 1.77(0.55) |
| Female immigrants >10(≥60y) | 16.23 (1.26) | 15.65 (1.47) | 15.08 (1.70) | 14.55 (1.94) | 14.03 (2.18) | 13.54 (2.43) | 13.07 (2.68) | 12.62 (2.92) | 12.19 (3.16) |
| Female immigrants ≤ 10(≥60y) | 13.01 (4.83) | 12.52 (5.11) | 12.03 (5.37) | 11.55 (5.63) | 11.08 (5.88) | 10.63 (6.12) | 10.19 (6.35) | 9.76(6.56) | 9.34 (6.75) |
| Female locals(≥60y) | 11.86 (1.84) | 11.67 (1.98) | 11.49 (2.15) | 11.33 (2.35) | 11.18 (2.58) | 11.04 (2.84) | 10.91 (3.11) | 10.79(3.4) | 10.68 (3.71) |
| Male immigrants >10(≥ 60y) | 36.59 (2.56) | 35.17(3.18) | 33.82 (3.86) | 32.55 (4.57) | 31.34 (5.28) | 30.19 (6.01) | 29.08 (6.70) | 28.02 (7.40) | 27.01 (8.07) |
| Male immigrants ≤10(≥ 60y) | 41.43 (11.78) | 41.03 (12.71) | 40.61 (13.70) | 40.17 (14.75) | 39.71 (15.85) | 39.24 (16.99) | 38.75 (18.16) | 38.23 (19.35) | 37.71 (20.57) |
| Male locals(≥60y) | 22.69 (3.56) | 22.37(4.07) | 22.16(4.84) | 21.89 (5.86) | 21.61 (7.22) | 21.52 (9.02) | 21.74 (11.29) | 22.17 (14.03) | 22.73 (17.28) |

**eTable 5.** Predictive means and standard deviations (in brackets) of age-standardized mortality rates of stomach cancer per 100,000 population for each gender, age group (less than, greater or equal to 60 years old) and immigrant status from 2022 to 2030.

For peer review only



For peer review only - http://bmjopen.bmj.com/site/about/guidelines.xhtml

| Predictive mean of age-standardized mortality rates of prostate cancer per 100,000 population | | | | | | | | | |
|---|---|---|---|---|---|---|---|---|---|
| Year | 2022 | 2023 | 2024 | 2025 | 2026 | 2027 | 2028 | 2029 | 2030 |
| Male immigrants >10 | 14.59 (0.79) | 14.57 (0.96) | 14.56 (1.15) | 14.54 (1.37) | 14.51 (1.61) | 14.48 (1.86) | 14.45 (2.13) | 14.42 (2.42) | 14.38 (2.72) |
| Male immigrants ≤ 10 | 8.78 (3.11) | 8.58(3.29) | 8.39 (3.49) | 8.19(3.69) | 8.10(3.89) | 7.82(4.11) | 7.63(4.31) | 7.45(4.51) | 7.27(4.72) |
| Male locals | 9.66 (1.57) | 9.67(1.66) | 9.69 (1.77) | 9.72(1.91) | 9.75(2.06) | 9.78(2.23) | 9.82(2.43) | 9.86(2.64) | 9.9(2.88) |
| Male immigrants >10(<60 y) | 0.52 (0.17) | 0.51(0.19) | 0.50 (0.21) | 0.49(0.22) | 0.48(0.24) | 0.47(0.25) | 0.46(0.27) | 0.45(0.29) | 0.44(0.31) |
| Male immigrants ≤ 10(<60y) | 0.73 (0.77) | 0.81(0.93) | 0.87 (1.10) | 0.94(1.31) | 1.01(1.51) | 1.09(1.75) | 1.16(2.02) | 1.24(2.32) | 1.33(2.64) |
| Male locals(<60y) | 0.66 (0.14) | 0.66(0.16) | 0.66 (0.19) | 0.67(0.21) | 0.67(0.24) | 0.67(0.27) | 0.68(0.31) | 0.68(0.33) | 0.69(0.37) |
| Male immigrants >10(≥ 60y) | 49.61 (2.73) | 49.63 (3.29) | 49.64 (3.94) | 49.64 (4.68) | 49.64 (5.51) | 49.63 (6.38) | 49.62 (7.32) | 49.61 (8.32) | 49.58(9.37) |
| Male immigrants ≤ 10(≥60y) | 27.66 (9.78) | 26.53 (10.21) | 25.4 (10.63) | 24.28 (11.03) | 23.16 (11.41) | 22.07 (11.76) | 21.01 (12.09) | 19.96 (12.38) | 18.95(12.63) |
| Male locals(≥60y) | 31.48 (5.49) | 31.40 (5.76) | 31.32 (6.09) | 31.24 (6.48) | 31.15 (6.94) | 31.06 (7.44) | 30.96 (8.01) | 30.86 (8.61) | 30.74(9.26) |

**eTable 6.** Predictive means and standard deviations (in brackets) of age-standardized mortality rates of prostate cancer per 100,000 population for each age group (less than, greater or equal to 60 years old) and immigrant status from 2022 to 2030.

For peer review only



For peer review only - http://bmjopen.bmj.com/site/about/guidelines.xhtml

# Reporting checklist for cohort study.

Based on the STROBE cohort guidelines.

## Instructions to authors

Complete this checklist by entering the page numbers from your manuscript where readers will find each of the items listed below.

Your article may not currently address all the items on the checklist. Please modify your text to include the missing information. If you are certain that an item does not apply, please write "n/a" and provide a short explanation.

Upload your completed checklist as an extra file when you submit to a journal.

In your methods section, say that you used the STROBE cohortreporting guidelines, and cite them as:

von Elm E, Altman DG, Egger M, Pocock SJ, Gotzsche PC, Vandenbroucke JP. The Strengthening the Reporting of Observational Studies in Epidemiology (STROBE) Statement: guidelines for reporting observational studies.

| | | Reporting Item | Page Number |
|---|---|---|---|
| **Title and abstract** | | | |
| Title | #1a | Indicate the study’s design with a commonly used term in the title or the abstract | 1 |
| Abstract | #1b | Provide in the abstract an informative and balanced summary of what was done and what was found | 2 |
| **Introduction** | | | |
| Background / rationale | #2 | Explain the scientific background and rationale for the investigation being reported | 4 |
| Objectives | #3 | State specific objectives, including any prespecified hypotheses | 4 |
| **Methods** | | | |
| Study design | #4 | Present key elements of study design early in the paper | 5 |
| Setting | #5 | Describe the setting, locations, and relevant dates, including periods | 5 |

For peer review only

| | | | |
|---|---|---|---|
| | | of recruitment, exposure, follow-up, and data collection | |
| Eligibility criteria | #6a | Give the eligibility criteria, and the sources and methods of selection of participants. Describe methods of follow-up. | 5 |
| Eligibility criteria | #6b | For matched studies, give matching criteria and number of exposed and unexposed | n/a |
| Variables | #7 | Clearly define all outcomes, exposures, predictors, potential confounders, and effect modifiers. Give diagnostic criteria, if applicable | 5 |
| Data sources / measurement | #8 | For each variable of interest give sources of data and details of methods of assessment (measurement). Describe comparability of assessment methods if there is more than one group. Give information separately for for exposed and unexposed groups if applicable. | 5 |
| Bias | #9 | Describe any efforts to address potential sources of bias | 5 |
| Study size | #10 | Explain how the study size was arrived at | 5 |
| Quantitative variables | #11 | Explain how quantitative variables were handled in the analyses. If applicable, describe which groupings were chosen, and why | 5 |
| Statistical methods | #12a | Describe all statistical methods, including those used to control for confounding | |
| 5 | | | |
| Statistical methods | #12b | Describe any methods used to examine subgroups and interactions | 5 |
| Statistical methods | #12c | Explain how missing data were addressed | 5 |
| Statistical methods | #12d | If applicable, explain how loss to follow-up was addressed | n/a |
| Statistical methods | #12e | Describe any sensitivity analyses | |
| n/a | | | |
| **Results** | | | |
| Participants | #13a | Report numbers of individuals at each stage of study—eg numbers potentially eligible, examined for eligibility, confirmed eligible, | n/a |

For peer review only

| | | | |
|---|---|---|---|
| | | included in the study, completing follow-up, and analysed. Give information separately for for exposed and unexposed groups if applicable. | |
| Participants | #13b | Give reasons for non-participation at each stage | 5 |
| Participants | #13c | Consider use of a flow diagram | |
| n/a | | | |
| Descriptive data | #14a | Give characteristics of study participants (eg demographic, clinical, social) and information on exposures and potential confounders. Give information separately for exposed and unexposed groups if applicable. | 5 |
| Descriptive data | #14b | Indicate number of participants with missing data for each variable of interest | |
| n/a | | | |
| Descriptive data | #14c | Summarise follow-up time (eg, average and total amount) | |
| n/a | | | |
| Outcome data | #15 | Report numbers of outcome events or summary measures over time. Give information separately for exposed and unexposed groups if applicable. | |
| n/a | | | |
| Main results | #16a | Give unadjusted estimates and, if applicable, confounder-adjusted estimates and their precision (eg, 95% confidence interval). Make clear which confounders were adjusted for and why they were included | 6 |
| Main results | #16b | Report category boundaries when continuous variables were categorized | n/a |
| Main results | #16c | If relevant, consider translating estimates of relative risk into absolute risk for a meaningful time period | |
| n/a | | | |
| Other analyses | #17 | Report other analyses done—eg analyses of subgroups and interactions, and sensitivity analyses | 7 |

**Discussion**

For peer review only

| | | | |
|---|---|---|---|
| Key results | #18 | Summarise key results with reference to study objectives | 8 |
| Limitations | #19 | Discuss limitations of the study, taking into account sources of potential bias or imprecision. Discuss both direction and magnitude of any potential bias. | 10 |
| Interpretation | #20 | Give a cautious overall interpretation considering objectives, limitations, multiplicity of analyses, results from similar studies, and other relevant evidence. | 8 |
| Generalisability | #21 | Discuss the generalisability (external validity) of the study results | 9 |
| **Other Information** | | | |
| Funding | #22 | Give the source of funding and the role of the funders for the present study and, if applicable, for the original study on which the present article is based | 11 |

The STROBE checklist is distributed under the terms of the Creative Commons Attribution License CC-BY. This checklist was completed on 11. February 2023 using https://www.goodreports.org/, a tool made by the EQUATOR Network in collaboration with Penelope.ai

For peer review only

BMJ Open

# An age-period-cohort analysis and projection of cancer mortality in Hong Kong, 1998–2030



| | |
|---|---|
| Journal: | *BMJ Open* |
| Manuscript ID | bmjopen-2023-072751.R2 |
| Article Type: | Original research |
| Date Submitted by the Author: | 04-Aug-2023 |



| | |
|---|---|
| Complete List of Authors: | Zhao, Yanji; The Hong Kong Polytechnic University, Department of Applied Mathematics<br>Zhuang, Zian; The Hong Kong Polytechnic University, Department of Applied Mathematics; University of California Los Angeles, Department of Biostatistics<br>Yang, Lin; The Hong Kong Polytechnic University, School of Nursing<br>He, Daihai; The Hong Kong Polytechnic University, Department of Applied Mathematics; The Hong Kong Polytechnic University, Research Institute for Future Food |



| | |
|---|---|
| <b>Primary Subject Heading</b>: | Research methods |
| Secondary Subject Heading: | Public health, Oncology |
| Keywords: | STATISTICS & RESEARCH METHODS, ONCOLOGY, Risk Factors |
| | |


SCHOLARONE™ Manuscripts

BMJ

*I, the Submitting Author has the right to grant and does grant on behalf of all authors of the Work (as defined in the below author licence), an exclusive licence and/or a non-exclusive licence for contributions from authors who are: i) UK Crown employees; ii) where BMJ has agreed a CC-BY licence shall apply, and/or iii) in accordance with the terms applicable for US Federal Government officers or employees acting as part of their official duties; on a worldwide, perpetual, irrevocable, royalty-free basis to BMJ Publishing Group Ltd ("BMJ") its licensees and where the relevant Journal is co-owned by BMJ to the co-owners of the Journal, to publish the Work in this journal and any other BMJ products and to exploit all rights, as set out in our licence.*

*The Submitting Author accepts and understands that any supply made under these terms is made by BMJ to the Submitting Author unless you are acting as an employee on behalf of your employer or a postgraduate student of an affiliated institution which is paying any applicable article publishing charge ("APC") for Open Access articles. Where the Submitting Author wishes to make the Work available on an Open Access basis (and intends to pay the relevant APC), the terms of reuse of such Open Access shall be governed by a Creative Commons licence – details of these licences and which Creative Commons licence will apply to this Work are set out in our licence referred to above.*

*Other than as permitted in any relevant BMJ Author's Self Archiving Policies, I confirm this Work has not been accepted for publication elsewhere, is not being considered for publication elsewhere and does not duplicate material already published. I confirm all authors consent to publication of this Work and authorise the granting of this licence.*

For peer review only

# An age-period-cohort analysis and projection of cancer mortality in Hong Kong, 1998–2030

Yanji Zhao[1, #], Zian Zhuang[1,2, #], Lin Yang[3] and Daihai He [1,4*]

1 Department of Applied Mathematics, Hong Kong Polytechnic University, Hong Kong, China

2 Department of Biostatistics, University of California, Los Angeles, CA, United States

3 School of Nursing, Hong Kong Polytechnic University, Hong Kong, China

4 Research Institute for Future Food, Hong Kong Polytechnic University, Hong Kong, China

# These authors contributed equally to this work.

* To whom correspondence should be addressed:

Department of Applied Mathematics, The Hong Kong Polytechnic University, Hong Kong, China
Tel: 852-27667864; daihai.he@polyu.edu.hk (DH).

**Email of all authors**

Yanji Zhao: yan-ji.zhao@connect.polyu.hk; Zian Zhuang: zianzhuang@ucla.edu;

Lin Yang: l.yang@polyu.edu.hk; Daihai He: daihai.he@polyu.edu.hk.

*Word count:3342*



For peer review only - http://bmjopen.bmj.com/site/about/guidelines.xhtml

# Abstract

**Objectives:** To explore the relationship between immigration groups and cancer mortality, this study aimed to explore age, period, birth cohort effects and effects across genders and immigration groups on mortality rates of lung, pancreatic, colon, liver, prostate and stomach cancers and their projections.

**Design, Setting, and Participants:** Death registry data in Hong Kong between 1998 and 2021, which were stratified by age, sex and immigration status. Immigration status was classified into three groups: locals born in Hong Kong, long-stay immigrants and short-stay immigrants.

**Methods:** Age-period-cohort analysis was used to examine age, period, and birth cohort effects for genders and immigration groups from 1998 to 2021. Bayesian age-period-cohort models were applied to predict the mortality rates from 2022 to 2030.

**Results:** Short-stay immigrants revealed pronounced fluctuations of mortality rates by age and of relative risks by cohort and period effects for six types of cancers than those of long-stay immigrants and locals. Immigrants for each type of cancer and gender will be at a higher mortality risk than locals, as men will be at a higher risk of mortality from cancers than women in the future (excluding prostate cancer). After 2021, decreasing trends ($p<0.05$) or plateau ($p>0.05$) of forecasting mortality rates of cancers occur for all immigration groups, except for increasing trends for short-stay male immigrants with colon cancer ($p<0.05$, Avg +0.30 deaths/100,000 per annum from 15.47 to 18.50 deaths/100,000) and long-stay male immigrants with pancreatic cancer ($p<0.05$, Avg +0.72 deaths/100,000 per annum from 16.30 to 23.49 deaths/100,000).,

**Conclusions:** Findings underscore the effect of gender and immigration status in Hong Kong on mortality risks of cancers that immigrants for each type of cancer and gender will be at a higher mortality risk than locals.


***Keywords***: Age-period-cohort analysis, immigration, mortality, lung cancer, pancreatic cancer, colon cancer, liver cancer, prostate cancer, stomach cancer



For peer review only - http://bmjopen.bmj.com/site/about/guidelines.xhtml

## Strengths and limitations of this study

- This study provides new evidence regarding the relationship between immigration status and cancer mortality, given the effects of age, period, birth cohort and their predictions.

- The non-identifiability problem has not been interpreted in APC models

- The future perspective of cancer therapies and techniques have not been considered.

For peer review only

# Introduction

Several migration waves from mainland China to Hong Kong have occurred over the past century. These migration waves included a large-scale migration inflow from 1945 to 1950 (the Chinese Civil War) and a few small-scale inflows in the 1950s, 1970s, and 1990s [1-3]. In 2016, immigrants from mainland China formed approximately 38% of the population of Hong Kong. These inflows have led to a growing interest in research on the disparity of health conditions between the locals and immigrants.

Cancer has been one of the most common causes of death, as an estimated 19.3 million new cancer cases and 9.9 million new cancer-associated deaths occurred worldwide in 2020 [4]. In Hong Kong, lung cancer is one of the most common causes of cancer deaths [5, 6]. Previous studies suggested that the primary cause of lung cancer is cigarette smoking [7-11]. Genetic factors, asbestos, radon gas, second-hand smoke, and other forms of air pollution have been proven to influence the risk of lung cancer [12-18]. The overall daily smoking rate in mainland China was approximately 23.2% in 2018 [19], whereas the daily smoking rate in Hong Kong was only 10.2% in 2019 [20]. The leading causes of liver cancer include viral infection, drinking of alcohol and polluted water and food supplies which are also culprits for colon, stomach and pancreatic cancer [21]. Alcohol consumption per capita in Hong Kong has reached 2.37 liters in 2021 [22], compared to 7.0 liters of per capita consumption of alcohol in mainland China in 2018 [23]. As approximately 99% of prostate cancer cases occur after age 50, factors of prostate cancer have been regarded as old age, race, family history and the diet of red meat consumption [24]. In addition to these risk factors, studies have suggested that cancer mortality rates vary depending on migrant status [25-28]. According to data from the Census and Statistics Department of Hong Kong, approximately 81% of immigrants in Hong Kong immigrated from mainland China, Macau, and Taiwan. Immigrants from mainland China account for the bulk of this population. Previous studies have shown that child immigrants in Hong Kong tend to suffer from a higher risk of wheezing disorders and cardiovascular diseases, and immigrant women have higher age-specific mortality rates of breast cancer than locally-born women in Hong Kong [29, 30]. However, to date, few studies have investigated the effect of length of stay in Hong Kong and birthplace on the risk of other types of cancer.

In this study, we compared the mortality rates of lung, pancreatic, colon, liver, prostate and stomach cancers between locally born residents in Hong Kong and immigrants from mainland China. Both populations are widely considered as ethnically homogeneous with similar cultures. Nevertheless, due to different early life experiences, immigrants are exposed to more various social economy and lifestyles than locals. Therefore, it's constructive to ascertain whether immigrants from mainland China have a different mortality pattern of cancers from locals to



For peer review only - http://bmjopen.bmj.com/site/about/guidelines.xhtml

verify the significance of migration status for this health outcome. As Age-period-cohort (APC) analysis plays a vital role in studying time-specific phenomena in epidemiology, in this study, to evaluate the effect of immigration on cancer mortality in the past and future, we developed APC models specified by sex and migrant status to assess the effects of age, period, birth cohort, and of the length of stay in Hong Kong on the mortality risks of cancers. Additionally, we explore the projection of mortality rates for the locally born population and immigrants in Hong Kong who were younger or older than 60 using a predictive model, taking into account age, period, and birth cohort effects as well.

# Methods

## *Data*

We obtained the death registry data in Hong Kong between 1998 and 2021 from the Census and Statistics Department of Hong Kong, as the data in 2022 has not been available up to now. The data was extracted from a routine census held by the Hong Kong government as subjective errors caused by resampling can be neglected. The population data were stratified by age, sex, immigration status, and length of stay in Hong Kong. We retrieved six types of cancer cases from the death registry data using ICD codes, such as ICD-9 code 162 and ICD-10 codes C34.0–C34.3, C348, and C349 for lung cancer. To assure comparability among registries, deaths from the age group of 35–85 years were selected, since cases younger than 35 and older than 85 were relatively trivial for lack of statistical interpretability [31]. Immigration status was classified into three groups: locals born in Hong Kong, immigrants who have lived in Hong Kong for >10 years before death defined as long-stay immigrants, and immigrants who have lived in Hong Kong for ≤10 years before death defined as short-stay immigrants. Notably, much focus was placed on immigrants from mainland China, because approximately 81% of immigrants in Hong Kong came from mainland China, Macau, and Taiwan based on the data from the Census and Statistics Department of Hong Kong. Moreover, few cases recorded from Macau and Taiwan are statistically insignificant in the analysis. Demographics and population projections from 2022 to 2030 were retrieved from the Census and Statistics Department of Hong Kong and estimated with cubic smoothing spline as the prerequisite of the predictive model. Codes for APC and BAPC analysis are available in the GitHub repository (https://github.com/kshz2164313/APC-population-projections-for-immigration-HK).

## *Statistical analysis*

We modeled cancer mortality rates in Hong Kong using APC analysis based on log-linear Poisson regression models. The model aimed to disentangle age, period, and cohort effects of time-varying phenomena simultaneously [32, 33], given that



For peer review only - http://bmjopen.bmj.com/site/about/guidelines.xhtml

$$\log(E_{ij}) = \alpha_i + \beta_j + \gamma_k + \mu + \log(\theta_{ij}) \quad (1)$$

where $E_{ij}$ denotes expected mortality; $\alpha_i$, $\beta_j$, and $\gamma_k$ denote age, period, and cohort effect, respectively, for $i=1,...,I$, $j=1,...,J$, $k=1,...,K$ with $k=I-i+j$. $\log(\theta_{ij})$ is the offset. We mainly focused on the contributions of sex and immigration status due to the non-identifiability problem that the effects of these three components are collinear with each other (denoted as period – age = cohort) [34]. Birth cohort effect and period effect were assessed with relative risks to evaluate the effect of three components. The median year of birth among cases was regarded as the reference cohort [35,36]. Since death cases aged at 35–85 years between 1998 and 2021 were selected, the range of birth cohort from 1913 to 1986 covered observations and further projections until 2030. The second and penultimate period effects were constrained to the reference for period. For sex and immigration status, maximum likelihood framework was applied to estimate the relative risks and 95% confidence intervals (CIs) by age groups, calendar period, and birth cohort.

Several projection approaches for future cancer mortality have been developed, but a Bayesian age-period-cohort (BAPC) model built upon integrated nested Laplace approximations (INLA) [37] yields relatively higher coverage and better performance for all evaluated parameter combinations [38]. To prevent some sampling problems caused by Markov chain Monte Carlo (MCMC), this MCMC-free BAPC approach was applied to predict future cancer mortality within a fully Bayesian inference setting and provide outputs of interest simply, such as projected age-standardized and age-specific rates. Convergence checks are not necessary for this technique [37]. The projections of age-standardized cancer mortality rates for each sex, age group (younger or older than 60 years) and migrant status, taking into account age, period, and birth cohort effects, were performed based on the weights of population age groups from the WHO World Standard population [39], with 95% prediction intervals. Mann-Kendall trend test was applied to verify the projection trend.

All analyses were performed via R version 4.2.1 (R Core Team, R Foundation for Statistical Computing, Vienna, Austria, 2013, http://www.R-project.org/). The APC models were established using the Epi package, and the projections based on Bayesian APC models were performed with the BAPC package.

*Patient and Public Involvement*

None.

## Results

Figure 1 & 2 and eFigure 1(a-e) in **Supplementary Material** illustrate the estimates of age



For peer review only - http://bmjopen.bmj.com/site/about/guidelines.xhtml

For peer review only

(assessed by cancer mortality), cohort and period effects (assessed by relative risk) based on APC models among three migrant groups for men and women with six types of cancers, respectively. All the mortality rates for each gender and immigration status exhibit notable increasing trends with age. Age, cohort and period effects of six types of cancer for immigrants who stayed in Hong Kong for ≤10 years revealed relatively more pronounced fluctuations and deviations from those effects in the other two immigration groups. Significant increasing trends of age effect occurred in all types of cancer, regardless of gender and immigration status. For example, while relatively insignificant differences in lung cancer mortality rates by immigration status among females have performed, male immigrants who remained in Hong Kong for >10 years had higher lung cancer mortality rates at ages above 50 years and those who arrived ≤10 years had lower lung cancer mortality at ages below 62 years compared to local men Figure 1. In addition to compatible dynamics of period effect for locals and long-stay immigrants, similar changes of relative risks by birth cohort for locals and long-stay immigrants in lung, colon, liver and stomach cancers occurred before 1945, whereas significant differences of relative risks by birth cohort between these two immigration groups occurred after 1960 (Figure 1 & eFigure 1(a,b,d)). Locals and long-stay immigrants in pancreatic and prostate cancer perform almost similar changes of relative risks by birth cohort effects all the time (eFigure 1(c,e)). Short-stay immigrants who have stayed in Hong Kong for ≤10 years had more fluctuating relative risks affected by period effects before 2020 than those for locals and long-stay immigrants. Lack of young cases, especially young short-stay immigrants, of prostate cancer leads to significant deviations and variances in age and cohort effects.

Figure 3-5, eFigure 2-6 in **Supplementary Material** illustrate the age-standardized mortality rates of six types of cancer from 1998 to 2021 and their projections by sex, immigrant status and age groups from 2022 to 2030, taking into account age, period, and birth cohort effects. Means and standard deviations of predictive mortality rates are shown in eTable 1-6 in **Supplementary Material**. For all ages projection (Figure 2 & eFigure 2-6), as men will be at higher risk of mortality rates of cancers (excluding prostate cancer) than women in the future for all three age groups (all ages, young and older than 60 years), given the projected trends, immigrants for each gender, especially who have stayed in Hong Kong for > 10 years will suffer from higher mortality rates of cancer in each year than locals. Monotone decreasing trends or plateau of forecasting occur for both genders and all immigration groups in cancers, except for increasing trends for male immigrants who have stayed in Hong Kong for ≤10 years with colon cancer ($p < 0.05$, Avg +0.30 deaths/100,000 per annum) from 15.47 deaths/100,000 (95% CI: 11.28, 19.66) in 2021 to 18.50 deaths/100,000 (95% CI: 2.31, 34.69) in 2030, and male immigrants who have stayed in Hong Kong for > 10 years with pancreatic cancer ($p < 0.05$, Avg +0.72 deaths/100,000 per annum) from 16.30 deaths/100,000 (95% CI: 14.38,17.26) in 2021 to 23.49 deaths/100,000 (95% CI: 12.49, 34.49) in 2030. Most of predictive trends for



For peer review only - http://bmjopen.bmj.com/site/about/guidelines.xhtml

younger cases (<60 years) and older cases (≥60 years) reach a consensus with those for all ages population, except for two phenomena: 1.) mortality rates of lung cancer for men immigrants ≤10 that insignificant trend for all ages ($p > 0.05$) vs. decline for younger cases ($p < 0.05$) vs. increase for older cases ($p < 0.05$); 2.) mortality rates of liver cancer for men immigrants >10 that decline for all ages ($p < 0.05$) vs. decline for younger cases ($p < 0.05$) vs. insignificant trend for older cases ($p > 0.05$). Some particular cases occur in the projection of prostate cancer that young long-stay male immigrants (0.44 deaths/100,000, 95% CI: 0, 1.05) aged less than 60 will be at lower mortality rate than locals (0.69 deaths/100,000, 95% CI: 0, 1.42) in 2030 (eTable 6). Compared with other cancers and immigration groups, male immigrants who have stayed in Hong Kong for >10 years with lung cancer would perform the most significant decline in predictive mean from 102.90 (95% CI: 98.14, 107.66) to 79.55 (95% CI: 47.46, 111.64) deaths per 100,000 population (Avg -2.34 deaths/100,000 per annum) (eTable 1), while the same immigration group with pancreatic cancer would indicate the most significant uptrend in each year of 16.30 (95% CI: 14.38,17.26) and 23.49 (95% CI: 12.49, 34.49) deaths per 100,000 population in 2021 and 2030, respectively (Avg +0.72 deaths/100,000 per annum) (eTable 4).

## Discussion

Early detection of cancer is positive and instructive for increasing chances of cure. Nevertheless, the high mortality rate of cancer results from late diagnosis among most patients after progression to more advanced or severe stages. Individuals at high risk of cancer, such as smokers, alcoholics or those who are frequently exposed to susceptible circumstances, should be screened for early detections to increase opportunities for cure [40]. Therefore, the differences in mortality rates among immigration groups are synonymous with detection means, therapies, and social history in disparate periods and areas.

While the changes in mortality rates by age for long-stay immigrants reached approximate harmony with those for locals, the changes in mortality rates by age for short-stay immigrants revealed clear differences with those for the other two populations. The group of long-stay immigrants had a higher risk of death from lung, colon and liver cancers than the other two immigration groups after the age of 60 years. Short-stay male immigrants were less likely to die from lung cancer before the age of 65 years. The contrast in age effects among the immigration groups was partially consistent with studies [25, 41] that highlighted the age effects for locals and immigrants on breast cancer mortality in Hong Kong and lung cancer incidence in Sweden, as they both showed similar trends and magnitudes between locals and immigrants before the age of 60 years. They are also compatible with the results in [42] that



For peer review only - http://bmjopen.bmj.com/site/about/guidelines.xhtml

diagnosis of liver cancer is the most frequent among populations at 55 to 65 years old. According to these trends, young individuals, especially new young immigrant men, who have benefited from all-rounded development in mainland China and Hong Kong, are more likely to seek early detection and be treated for cancers using more advanced treatments [43]. Differences in birth cohort effects among immigrant groups partially comply with the interpretation above.

We observed significant trends of cohort effects among locals and immigrants. These findings are partially consistent but subtly different from previous findings, regarding the effect of immigration status on cancers. Zhao et al. [25] described multiple peaks of cohort effects on breast cancer mortality between locals and immigrants in Hong Kong, as well as a significant decline in cohort effects after 1950. In contrast, Sung et al. [44] investigated the difference in breast cancer incidence between Chinese Americans and non-Hispanic whites in the U.S. and emphasized that Chinese Americans were at lower risk of breast cancer than non-Hispanic whites born in the same year. Here, we interpret the cohort-driven trends resulting from the intricacy of social history and lifestyle. Compared to a relatively stable social development in Hong Kong, representing downward trends of relative risks for locals, wars and social instability in mainland China resulted in several immigration waves from mainland China to Hong Kong before 1950. Additionally, remarkable increasing trends were recorded for new immigrants after 1950, which corresponded to the economic downturn after wars and famine between 1959 and 1961 during their youth [45]. The increasing trends for new immigrants and similar trends for locals and long-stay immigrants were consistent with the finding that nutrient deficiency contributes to a higher risk of severe mortality rates of cancers [46]. Furthermore, we speculate that these trends, especially those for locals and long-stay immigrants, are most likely attributed to social development and personal behaviors, such as daily habits, occupational history, different diagnoses and treatments, and domestic environmental exposures. Notably, short-stay immigrants suffered from a lower risk of death from colon cancer for all ages (eFigure 1a in **Supplementary Material**). As locals and immigrants in Hong Kong transitioned to more westernized lifestyles, higher consumption of meat was associated with a higher risk of these types of cancer, whereas consumption of vegetables had a strong protective effect against pancreatic cancer, and moderate consumption of coffee appeared to be beneficial against lung cancer [47,48]. Further studies on potential risk factors are required.

Short-stay immigrants had more fluctuating and non-stationary but inconspicuous relative risks by period effects before 2021 than locals and long-stay immigrants. Cumulatively, an arch pattern and fluctuating curve depicting period effects externally resulted in an arch pattern of age-standardized mortality rates for short-stay immigrant women and irregular rates



For peer review only - http://bmjopen.bmj.com/site/about/guidelines.xhtml

for short-stay immigrant men before 2021. The external performance of different period effects on mortality rates could be most likely attributed to the higher effect of different lifestyles and social development on new immigrants than on long-stay immigrants and locals in Hong Kong. For the age-standardized mortality rates and projections, consistent with previous findings [49,50], we predict that the mortality rates of cancer in Hong Kong after 2021 will continue to decline or remain relatively stable, consistent with the trends before 2020, except for male immigrants who have stayed in Hong Kong for ≤10 years with colon cancer and male immigrants who have stayed in Hong Kong for >10 years with pancreatic cancer. Men will be at higher risk of mortality rates of cancer than women, regardless of immigration status. They are also compatible with the results in [4] that men suffer from a higher risk of these types of cancer than women, excluding prostate cancer. Furthermore, new immigrant women will be at lower risk than local women, even though long-stay immigrants will suffer from higher mortality rates than locals in the future. Potential interpretations could be consistent with those for birth cohort effects, as age and period effects are considered as confounders of cohort effect.

In the past few decades, spurred by an increasing burden of high incidence and mortality rates of cancer, several studies focused on the inherent identification dilemma of three effects in the APC model. Further, complicated population distribution and immigration status in Hong Kong, one of the areas with the highest population density and migration frequency in the world, have intricate causes and inherent dynamics of cancer and other diseases. To our knowledge, few studies have assessed the relationship between immigration status and cancer mortality. Therefore, this study is original to examine the effect of the length of stay in Hong Kong and origins of previous residence on cancer deaths, which is instructive for further immigration policy-making and targeted strategies of disease detection and intervention. However, this study had several limitations. Given the non-identifiability problem in age-period-cohort models, we could only depict trends and variations among different immigration and sex groups, as illustrated in figures, and insufficiently perform the estimates of the contributions of three effects or subgroups to mortality rates. Furthermore, we adopted a cubic smoothing spline to estimate populations of immigrants and locals due to the large proportion of unspecified immigration status from official demographic projections. A few acceptable cases resulted in a limited type of cancer so that some common cancers, such as the ovary and cervix, were discarded. Since the issue of quantification, the future perspective of cancer therapies and techniques have not been considered in the model of projection.

## Conclusion



For peer review only - http://bmjopen.bmj.com/site/about/guidelines.xhtml

326 We conclude that immigrants, especially short-stay immigrants, had more pronounced fluctuations of mortality rates by age and of relative risks by cohort and period effects for six types of cancers than those of long-stay immigrants and locals. Men will be at a higher risk of mortality rates of six types of cancer than women in the future. Male immigrants who have stayed in Hong Kong for ≤10 years with colon cancer and male immigrants who have stayed in Hong Kong for >10 years with pancreatic cancer would perform significant uptrend in the future, while other immigration groups for each type of cancer would continue to decline or remain relatively stable. Immigrants for each gender in Hong Kong would suffer from higher mortality risks of cancers than locals in the future.

For peer review only



For peer review only - http://bmjopen.bmj.com/site/about/guidelines.xhtml

## Declaration

### Ethical approval and consent to participate

Ethical approval and consent to participate are not applicable. This study does not involve human participants. Data was obtained from the Census and Statistics Department of Hong Kong.

### Consent for publication

Not applicable.

### Data Availability Statement

Data are available upon reasonable request.

### Author contributions

**Yanji Zhao:** Methodology, Formal analysis, Data Curation, Writing - Original Draft, Visualization
**Zian Zhuang:** Methodology, Formal analysis, Data Curation, Writing - Review & Editing
**Lin Yang:** Validation, Writing - Review & Editing
**Daihai He:** Conceptualization, Writing - Review & Editing, Supervision

### Funding

The work described in this paper was partially supported by a grant from the Research Grants Council of the Hong Kong Special Administrative Region, China (HKU C7123-20G).

### Conflict of interest

None declared.

### Acknowledgements

None.

For peer review only

## References

1.Fan S-C. The population projection of Hong Kong. Southeast Asian Journal of Social Science. 1974;2(1/2):105-17.

2.Department CaS. Hong Kong Statistics 1947-1967 (Report). https://www.statistics.gov.hk/pub/hist/1961_1970/B10100031967AN67E0100.pdf, Accessed 4th May 2019.

3. Department CaS. Demographic Trends in Hong Kong 1981-2011 (Report). http://www.statistics.gov.hk/pub/B1120017032012XXXXB0100.pdf, Accessed 4th May 2019.

4. Sung H, Ferlay J, Siegel RL, Laversanne M, Soerjomataram I, Jemal A, et al. Global Cancer Statistics 2020: GLOBOCAN Estimates of Incidence and Mortality Worldwide for 36 Cancers in 185 Countries. CA: A Cancer Journal for Clinicians. 2021 2021/05/01;71(3):209-49. doi: https://doi.org/10.3322/caac.21660.

5. Wang XR, Chiu YL, Qiu H, Au JSK, Yu ITS. The roles of smoking and cooking emissions in lung cancer risk among Chinese women in Hong Kong. Annals of Oncology. 2009 2009/04/01/;20(4):746-51. doi: https://doi.org/10.1093/annonc/mdn699.

6. Chiu Y-L, Wang X-R, Qiu H, Yu IT-S. Risk factors for lung cancer: a case–control study in Hong Kong women. Cancer Causes & Control. 2010 2010/05/01;21(5):777-85. doi: 10.1007/s10552-010-9506-9.

7. Office on S, Health. Publications and Reports of the Surgeon General. Women and Smoking: A Report of the Surgeon General. Atlanta (GA): Centers for Disease Control and Prevention (US); 2001.

8. Escobedo LG, Peddicord JP. Smoking prevalence in US birth cohorts: the influence of gender and education. American Journal of Public Health. 1996 1996/02/01;86(2):231-6. doi: 10.2105/AJPH.86.2.231.

9. Husten CG, Shelton DM, Chrismon JH, Lin YC, Mowery P, Powell FA. Cigarette smoking and smoking cessation among older adults: United States, 1965-94. Tobacco Control. 1997;6(3):175. doi: 10.1136/tc.6.3.175.

10. Bolego C, Poli A, Paoletti R. Smoking and gender. Cardiovascular Research. 2002;53(3):568-76. doi: 10.1016/S0008-6363(01)00520-X.

11. Doll R, Hill AB. The mortality of doctors in relation to their smoking habits; a preliminary report. Br Med J. 1954;1(4877):1451-5. PMID: 13160495. doi: 10.1136/bmj.1.4877.1451.

12. Ramada Rodilla JM, Calvo Cerrada B, Serra Pujadas C, Delclos GL, Benavides FG. Fiber burden and asbestos-related diseases: an umbrella review. Gaceta Sanitaria. 2021 2021/06/11/. doi: https://doi.org/10.1016/j.gaceta.2021.04.001.

13. Collishaw NE, Kirkbride J, Wigle DT. Tobacco smoke in the workplace: an occupational health hazard. Can Med Assoc J. 1984;131(10):1199-204. PMID: 6498670.

14. Dresler CM, Fratelli C, Babb J, Everley L, Evans AA, Clapper ML. Gender differences in genetic susceptibility for lung cancer. Lung Cancer. 2000 2000/12/01/;30(3):153-60. doi: https://doi.org/10.1016/S0169-5002(00)00163-X.

15. Alexandrov K, Cascorbi I, Rojas M, Bouvier G, Kriek E, Bartsch H. CYP1A1 and GSTM1 genotypes affect benzo[ a ]pyrene DNA adducts in smokers' lung: comparison with aromatic/hydrophobic adduct formation. Carcinogenesis. 2002;23(12):1969-77. doi:



For peer review only - http://bmjopen.bmj.com/site/about/guidelines.xhtml

10.1093/carcin/23.12.1969.

16. Samet JM. Radon and Lung Cancer. JNCI: Journal of the National Cancer Institute. 1989;81(10):745-58. doi: 10.1093/jnci/81.10.745.

17. Darby S, Hill D, Auvinen A, Barros-Dios JM, Baysson H, Bochicchio F, et al. Radon in homes and risk of lung cancer: collaborative analysis of individual data from 13 European case-control studies. BMJ. 2005;330(7485):223. doi: 10.1136/bmj.38308.477650.63.

18. Raaschou-Nielsen O, Andersen ZJ, Beelen R, Samoli E, Stafoggia M, Weinmayr G, et al. Air pollution and lung cancer incidence in 17 European cohorts: prospective analyses from the European Study of Cohorts for Air Pollution Effects (ESCAPE). The Lancet Oncology. 2013 2013/08/01/;14(9):813-22. doi: https://doi.org/10.1016/S1470-2045(13)70279-1.

19. *2018 Summary-20190719*. Retrieved August 26, 2022, from https://www.who.int/docs/default-source/wpro---documents/countries/china/2018-gats-china-factsheet-cn-en.pdf?sfvrsn=3f4e2da9_2

20. *Thematic household survey*.Retrieved August 26, 2022, from https://www.censtatd.gov.hk/en/data/stat_report/product/B1130201/att/B11302702020XXXXB0100.pdf

21. Abubakar II, Tillmann T, Banerjee A. Global, regional, and national age-sex specific all-cause and cause-specific mortality for 240 causes of death, 1990-2013: a systematic analysis for the Global Burden of Disease Study 2013. Lancet. 2015 Jan 10;385(9963):117-71.

22. Estimated alcohol consumption per capita in Hong Kong. Change4Health. (n.d.). Retrieved December 1, 2022, from https://www.change4health.gov.hk/en/alcohol_aware/figures/alcohol_consumption/index.html

23. World Health Organization. Global status report on alcohol and health 2018. World Health Organization; 2019 Feb 14.

24. Wild C. World cancer report 2014. Wild CP, Stewart BW, editors. Geneva, Switzerland: World Health Organization; 2014.

25. Zhao S, Dong H, Qin J, Liu H, Li Y, Chen Y, et al. Breast cancer mortality in Chinese women: does migrant status play a role? Annals of Epidemiology. 2019 2019/12/01/;40:28-34.e2. doi: https://doi.org/10.1016/j.annepidem.2019.10.006.

26. Gomez SL, Yang J, Lin S-W, McCusker M, Sandler A, Cheng I, et al. Incidence trends of lung cancer by immigration status among Chinese Americans. Cancer Epidemiol Biomarkers Prev. 2015;24(8):1157-64. PMID: 25990553. doi: 10.1158/1055-9965.EPI-15-0123.

27.Hemminki K, Li X, Czene K. Cancer risks in first-generation immigrants to Sweden. International Journal of Cancer. 2002 2002/05/10;99(2):218-28. doi: https://doi.org/10.1002/ijc.10322.

28. Vanthomme K, Rosskamp M, De Schutter H, Vandenheede H. Lung cancer incidence differences in migrant men in Belgium, 2004–2013: histology-specific analyses. BMC Cancer. 2021 2021/03/30;21(1):328. doi: 10.1186/s12885-021-08038-6.

29. Schooling M, Leung GM, Janus ED, Ho SY, Hedley AJ, Lam TH. Childhood migration and cardiovascular risk. International Journal of Epidemiology. 2004;33(6):1219-26. doi: 10.1093/ije/dyh221.

30. Leung JYY, Li AM, Leung GM, Schooling CM. Mode of delivery and childhood

For peer review only



For peer review only - http://bmjopen.bmj.com/site/about/guidelines.xhtml

hospitalizations for asthma and other wheezing disorders. Clinical & Experimental Allergy. 2015 2015/06/01;45(6):1109-17. doi: https://doi.org/10.1111/cea.12548.

31. Baker A, Bray I. Bayesian projections: what are the effects of excluding data from younger age groups?. American Journal of Epidemiology. 2005 Oct 15;162(8):798-805.

32. Rosenberg PS, Anderson WF. Age-Period-Cohort Models in Cancer Surveillance Research: Ready for Prime Time? APC Models. Cancer Epidemiology, Biomarkers & Prevention. 2011 Jul 1;20(7):1263-8.

33. Holford T. Analyzing the effects of age, period and cohort on incidence and mortality rates. Stat Meth Med Res. 1992;1:317-37.

34. Brookmeyer R, Stroup DF, editors. Monitoring the health of populations: statistical principles and methods for public health surveillance. Oxford University Press; 2004.

35. Yang Y, Land KC. Age-period-cohort analysis: New models, methods, and empirical applications. Taylor & Francis; 2013.

36. Robertson C, Gandini S, Boyle P. Age-period-cohort models: a comparative study of available methodologies. Journal of clinical epidemiology. 1999 Jun 1;52(6):569-83.

37. Riebler A, Held L. Projecting the future burden of cancer: Bayesian age–period–cohort analysis with integrated nested Laplace approximations. Biometrical Journal. 2017 May;59(3):531-49.

38. Knoll M, Furkel J, Debus J, Abdollahi A, Karch A, Stock C. An R package for an integrated evaluation of statistical approaches to cancer incidence projection. BMC medical research methodology. 2020 Dec;20(1):1-1.

39. Ahmad OB, Boschi-Pinto C, Lopez AD, Murray CJ, Lozano R, Inoue M. Age standardization of rates: a new WHO standard. Geneva: World Health Organization. 2001 Jan;9(10):1-4.

40. Sung H, Ferlay J, Siegel RL, Laversanne M, Soerjomataram I, Jemal A, Bray F. Global cancer statistics 2020: GLOBOCAN estimates of incidence and mortality worldwide for 36 cancers in 185 countries. CA: a cancer journal for clinicians. 2021 May;71(3):209-49.

41. Mousavi SM, Fallah M, Sundquist K, Hemminki K. Age-and time-dependent changes in cancer incidence among immigrants to Sweden: colorectal, lung, breast and prostate cancers. International journal of cancer. 2012 Jul 15;131(2):E122-8.

42. National Cancer Institute. SEER stat fact sheets: liver and intrahepatic bile duct cancer.

43. Wu X, Chung VC, Hui EP, Ziea ET, Ng BF, Ho RS, Tsoi KK, Wong S, Wu JC. Effectiveness of acupuncture and related therapies for palliative care of cancer: overview of systematic reviews. Scientific reports. 2015 Nov 26;5(1):1-5.

44. Sung H, Rosenberg PS, Chen WQ, Hartman M, Lim WY, Chia KS, Wai-Kong Mang O, Tse L, Anderson WF, Yang XR. The impact of breast cancer-specific birth cohort effects among younger and older C hinese populations. International journal of cancer. 2016 Aug 1;139(3):527-34.

45. The world economy volume 1: a millennial perspective, 2, Historical statistics: Academic Foundation, Gurgaon, India (2007)

46. Elias SG, Peeters PH, Grobbee DE, van Noord PA. The 1944-1945 Dutch famine and subsequent overall cancer incidence. Cancer Epidemiology Biomarkers & Prevention. 2005 Aug;14(8):1981-5.

47. Chiu YL, Wang XR, Qiu H, Yu IT. Risk factors for lung cancer: a case–control study in



For peer review only - http://bmjopen.bmj.com/site/about/guidelines.xhtml

491 Hong Kong women. Cancer Causes & Control. 2010 May;21(5):777-85.
492 48. Li J, Lam AS, Yau ST, Yiu KK, Tsoi KK. Antihypertensive treatments and risks of lung Cancer: A large population-based cohort study in Hong Kong. BMC cancer. 2021 Dec;21(1):1-9.
495 49. Du J, Sun H, Sun Y, Du J, Cao W, Sun S. Assessment of age, period, and cohort effects of lung cancer incidence in Hong Kong and projection up to 2030 based on changing demographics. American Journal of Cancer Research. 2021;11(12):5902.
498 50. *Centre for Health Protection, Department of Health - Lung Cancer*. Centre for Health Protection. Retrieved August 10, 2022, from https://www.chp.gov.hk/en/healthtopics/content/25/49.html

For peer review only



For peer review only - http://bmjopen.bmj.com/site/about/guidelines.xhtml

**Figure 1**. Parameter estimates of age (a), cohort (b) and period (c) effects based on an age-period-cohort model of male lung cancer mortality rates by immigrant groups: locals, immigrants stay in Hong Kong for more than 10 years and immigrants stay in Hong Kong for less than or equal to 10 years. Age effect was assessed by mortality (left axis). Cohort and period effects were assessed by relative risk (right axis), 95% confidence intervals are shown as shaded bands.

For peer review only



For peer review only - http://bmjopen.bmj.com/site/about/guidelines.xhtml

**Figure 2**. Parameter estimates of age (a), cohort (b) and period (c) effects based on an age-period-cohort model of female lung cancer mortality rates by immigrant groups: locals, immigrants stay in Hong Kong for more than 10 years and immigrants stay in Hong Kong for less than or equal to 10 years. Age effect was assessed by mortality (left axis). Cohort and period effects were assessed by relative risk (right axis), 95% confidence intervals are shown as shaded bands.

For peer review only



For peer review only - http://bmjopen.bmj.com/site/about/guidelines.xhtml

**Figure 3.** Projections of lung cancer mortality rates by gender and immigrant status from 2022 to 2030. Observations are shown as dots with the predictive distribution between the 5% and 95% quantile, whereby each lighter shade of red represents an additional 10% predictive CI. The predictive mean is shown as black solid line and the vertical dashed line indicates where prediction started.

For peer review only



For peer review only - http://bmjopen.bmj.com/site/about/guidelines.xhtml

**Figure 4.** Projections of lung cancer mortality rates for the population younger than 60 by gender and immigrant status from 2022 to 2030. Observations are shown as dots with the predictive distribution between the 5% and 95% quantile, whereby each lighter shade of red represents an additional 10% predictive CI. The predictive mean is shown as black solid line and the vertical dashed line indicates where prediction started.

For peer review only



For peer review only - http://bmjopen.bmj.com/site/about/guidelines.xhtml

**Figure 5.** Projections of lung cancer mortality rates for the population older than or equal to 60 by gender and immigrant status from 2022 to 2030. Observations are shown as dots with the predictive distribution between the 5% and 95% quantile, whereby each lighter shade of red represents an additional 10% predictive CI. The predictive mean is shown as black solid line and the vertical dashed line indicates where prediction started.

For peer review only



For peer review only - http://bmjopen.bmj.com/site/about/guidelines.xhtml

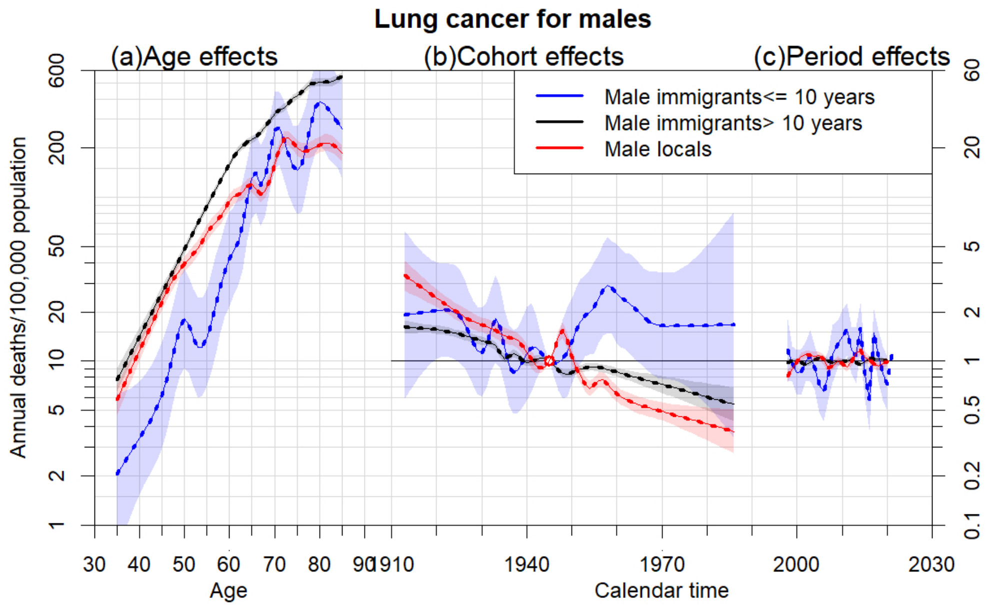


Figure 1. Parameter estimates of age (a), cohort (b) and period (c) effects based on an age-period-cohort model of male lung cancer mortality rates by immigrant groups: locals, immigrants stay in Hong Kong for more than 10 years and immigrants stay in Hong Kong for less than or equal to 10 years. Age effect was assessed by mortality (left axis). Cohort and period effects were assessed by relative risk (right axis), 95% confidence intervals are shown as shaded bands.

495x343mm (57 x 57 DPI)

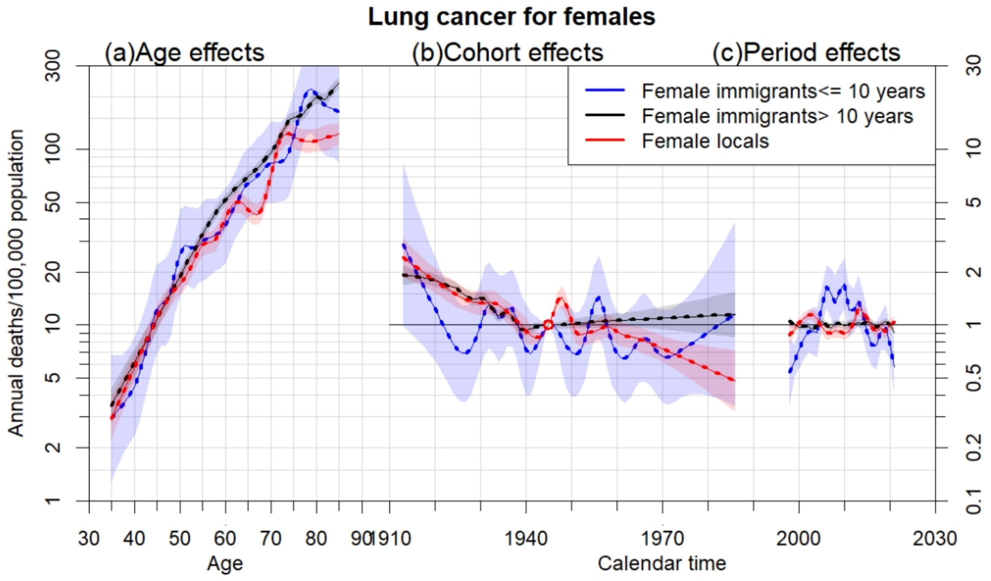


Figure 2. Parameter estimates of age (a), cohort (b) and period (c) effects based on an age-period-cohort model of female lung cancer mortality rates by immigrant groups: locals, immigrants stay in Hong Kong for more than 10 years and immigrants stay in Hong Kong for less than or equal to 10 years. Age effect was assessed by mortality (left axis). Cohort and period effects were assessed by relative risk (right axis), 95% confidence intervals are shown as shaded bands.

519x343mm (57 x 57 DPI)

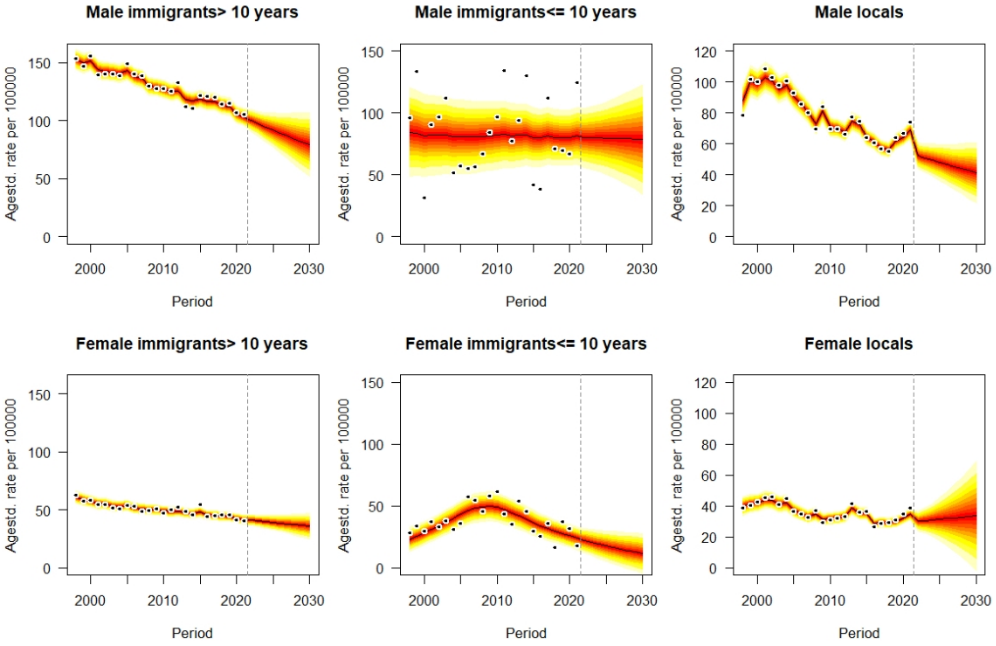


Figure 3. Projections of lung cancer mortality rates by gender and immigrant status from 2022 to 2030. Observations are shown as dots with the predictive distribution between the 5% and 95% quantile, whereby each lighter shade of red represents an additional 10% predictive CI. The predictive mean is shown as black solid line and the vertical dashed line indicates where prediction started.

519x343mm (57 x 57 DPI)

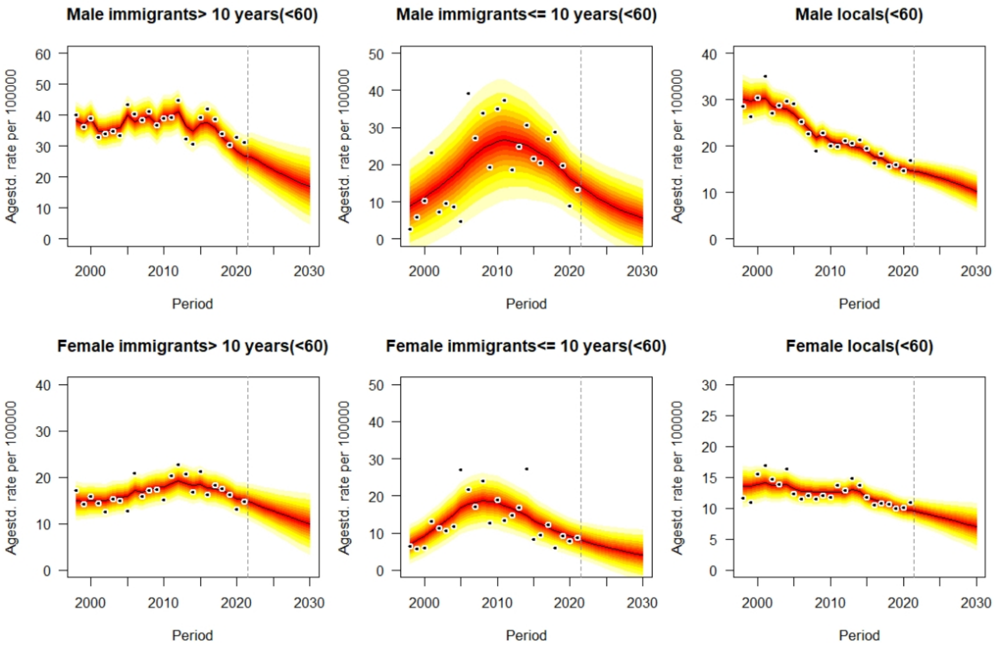


Figure 4. Projections of lung cancer mortality rates for the population younger than 60 by gender and immigrant status from 2022 to 2030. Observations are shown as dots with the predictive distribution between the 5% and 95% quantile, whereby each lighter shade of red represents an additional 10% predictive CI. The predictive mean is shown as black solid line and the vertical dashed line indicates where prediction started.

519x343mm (57 x 57 DPI)

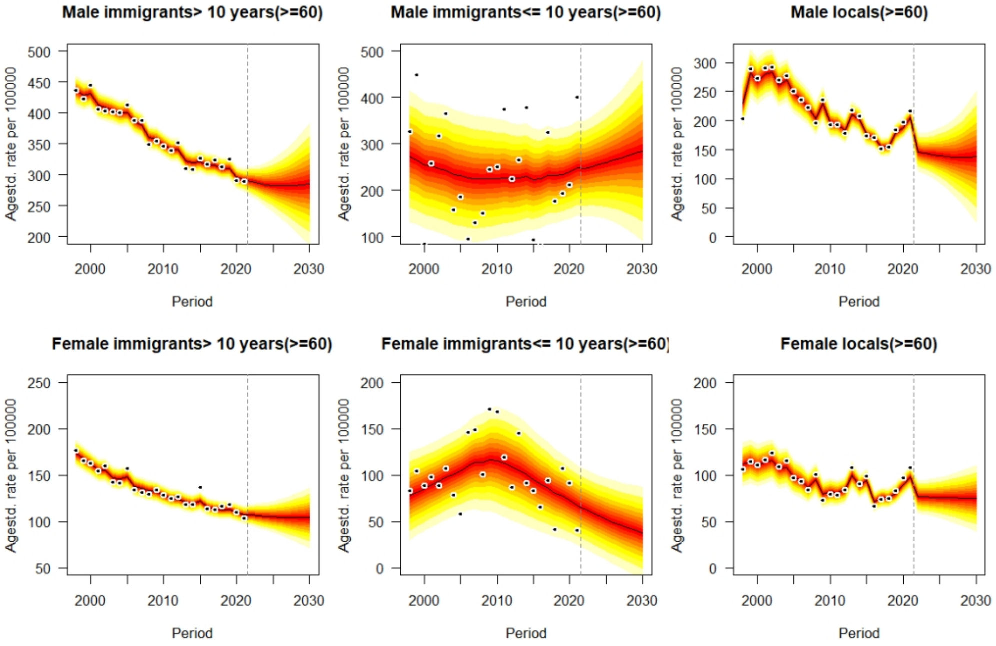


Figure 5. Projections of lung cancer mortality rates for the population older than or equal to 60 by gender and immigrant status from 2022 to 2030. Observations are shown as dots with the predictive distribution between the 5% and 95% quantile, whereby each lighter shade of red represents an additional 10% predictive CI. The predictive mean is shown as black solid line and the vertical dashed line indicates where prediction started.

519x343mm (57 x 57 DPI)

# Supplementary Material for

# "An age-period-cohort analysis and projection of cancer mortality in Hong Kong, 1998–2030"

For peer review only

## APC Figures

eFigure 1(a) Colon cancer ........ 3
eFigure 1(b) Liver cancer ........ 4
eFigure 1(c) Pancreatic cancer ........ 5
eFigure 1(d) Stomach cancer ........ 6
eFigure 1(e) Prostate cancer ........ 7

## Projection Figures

Colon cancer
eFigure 2(a)Projection (all ages) ........ 8
eFigure 2(b)Projection (<60 years) ........ 9
eFigure 2(c)Projection (⩾60 years) ........ 10
Liver cancer
eFigure 3(a)Projection (all ages) ........ 11
eFigure 3(b)Projection (<60 years) ........ 12
eFigure 3(c)Projection (⩾60 years) ........ 13
Pancreatic cancer
eFigure 4(a)Projection (all ages) ........ 14
eFigure 4(b)Projection (<60 years) ........ 15
eFigure 4(c)Projection (⩾60 years) ........ 16
Stomach cancer
eFigure 5(a)Projection (all ages) ........ 17
eFigure 5(b)Projection (<60 years) ........ 18



For peer review only - http://bmjopen.bmj.com/site/about/guidelines.xhtml

eFigure 5(c)Projection (⩾60 years) ........ 19

Prostate cancer

eFigure 6 Projection (all ages, <60 years and ⩾60 years) ........ 20

## Tables

eTable 1 Lung cancer ........ 21

eTable 2 Colon cancer ........ 22

eTable 3 Liver cancer ........ 23

eTable 4 Pancreatic cancer ........ 24

eTable 5 Stomach cancer ........ 25

eTable 6 Prostate cancer ........ 26



For peer review only - http://bmjopen.bmj.com/site/about/guidelines.xhtml

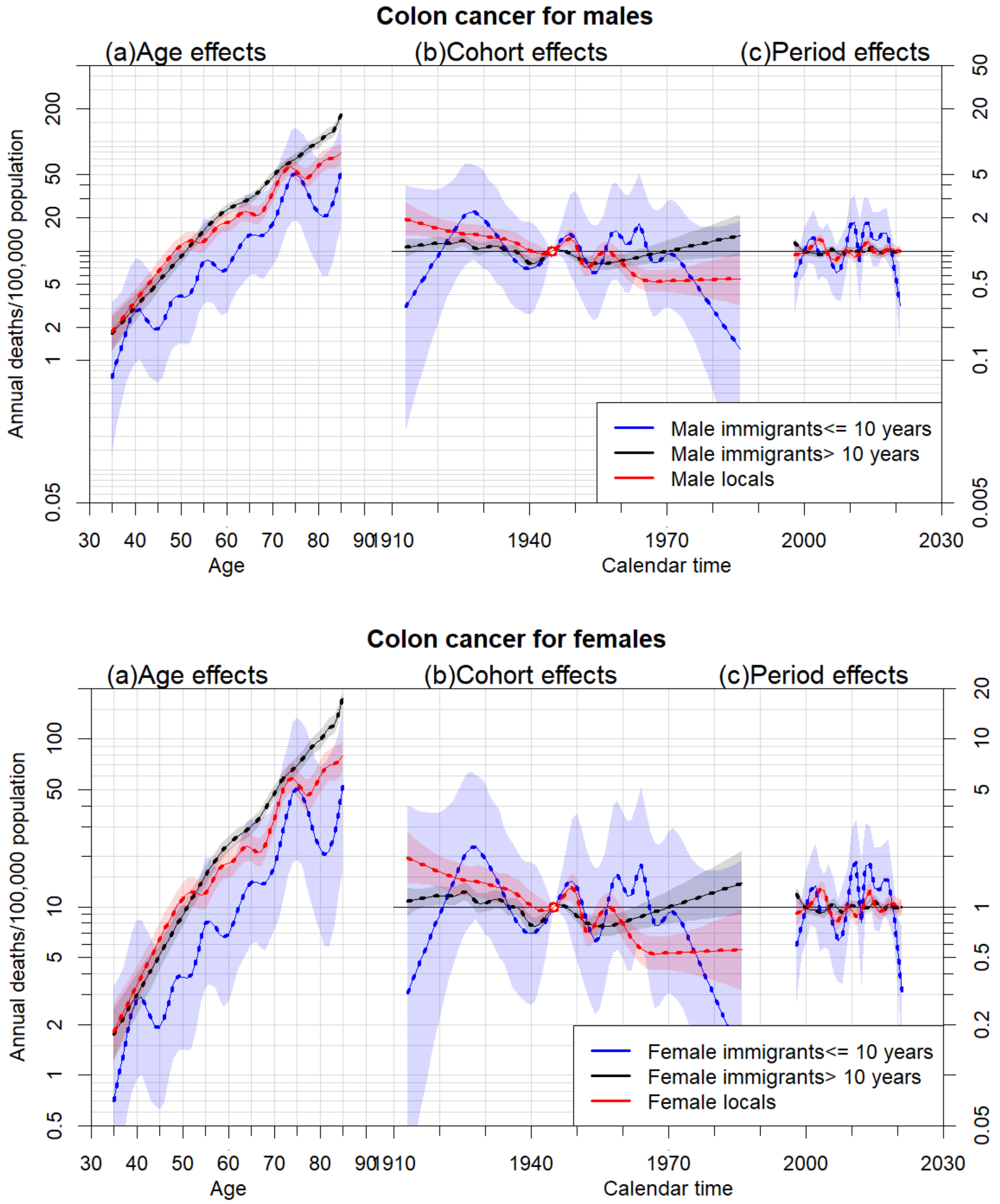


**eFigure 1(a)**. Parameter estimates of age (a), cohort (b) and period (c) effects based on an age-period-cohort model of male and female colon cancer mortality rates by immigrant groups: locals, immigrants stay in Hong Kong for more than 10 years and immigrants stay in Hong Kong for less than or equal to 10 years. Age effect was assessed by mortality (left axis). Cohort and period effects were assessed by relative risk (right axis), 95% confidence intervals are shown as shaded bands.



For peer review only - http://bmjopen.bmj.com/site/about/guidelines.xhtml

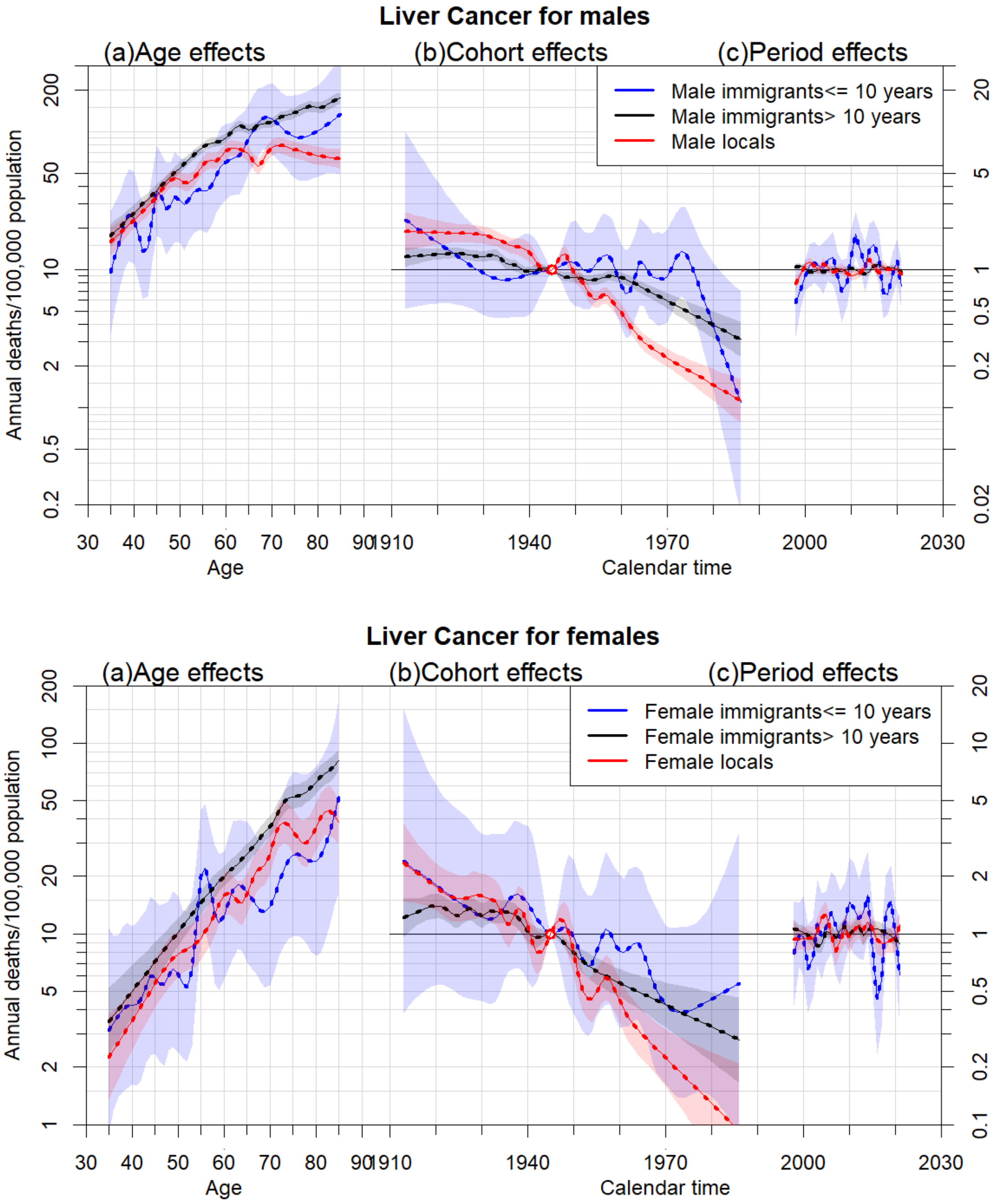


**eFigure 1(b)**. Parameter estimates of age (a), cohort (b) and period (c) effects based on an age-period-cohort model of male and female liver cancer mortality rates by immigrant groups: locals, immigrants stay in Hong Kong for more than 10 years and immigrants stay in Hong Kong for less than or equal to 10 years. Age effect was assessed by mortality (left axis). Cohort and period effects were assessed by relative risk (right axis), 95% confidence intervals are shown as shaded bands.



For peer review only - http://bmjopen.bmj.com/site/about/guidelines.xhtml

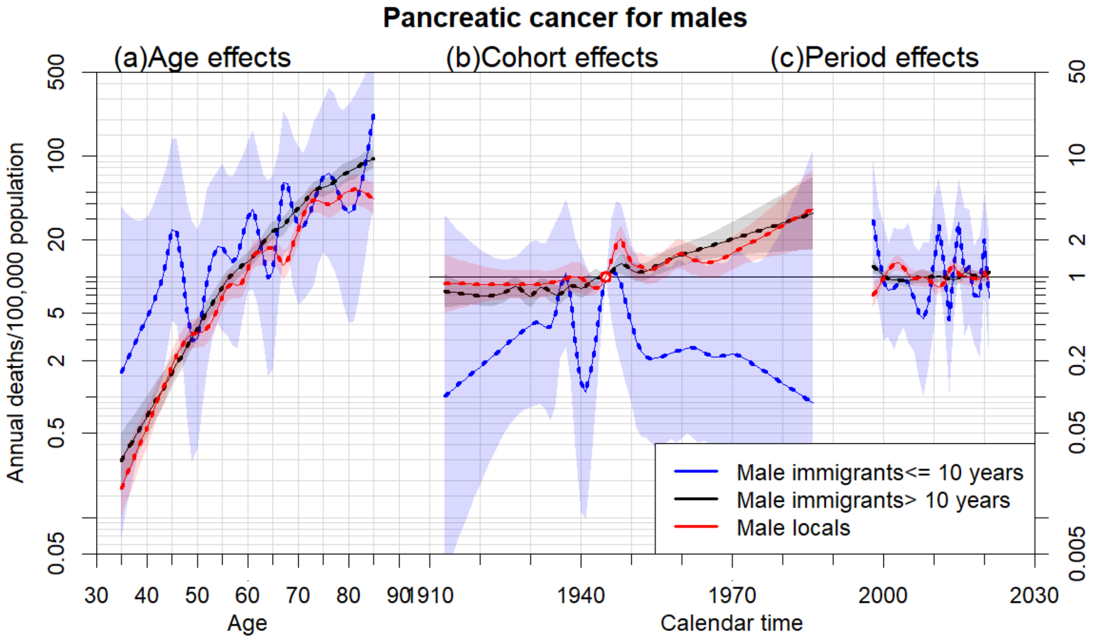


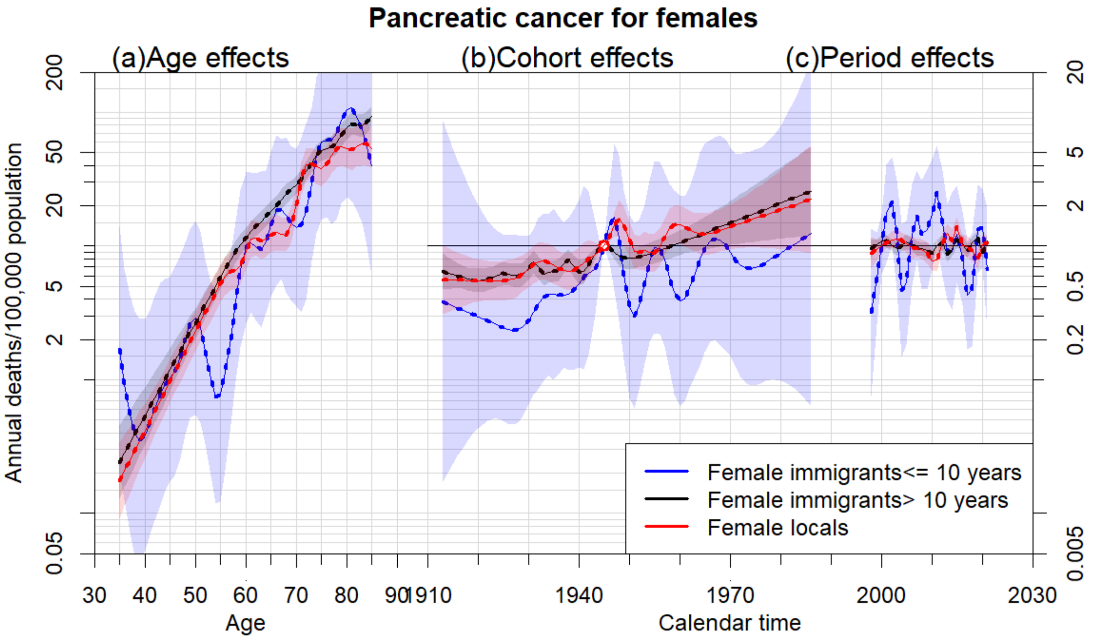


**eFigure 1(c)**. Parameter estimates of age (a), cohort (b) and period (c) effects based on an age-period-cohort model of male and female pancreatic cancer mortality rates by immigrant groups: locals, immigrants stay in Hong Kong for more than 10 years and immigrants stay in Hong Kong for less than or equal to 10 years. Age effect was assessed by mortality (left axis). Cohort and period effects were assessed by relative risk (right axis), 95% confidence intervals are shown as shaded bands.



For peer review only - http://bmjopen.bmj.com/site/about/guidelines.xhtml

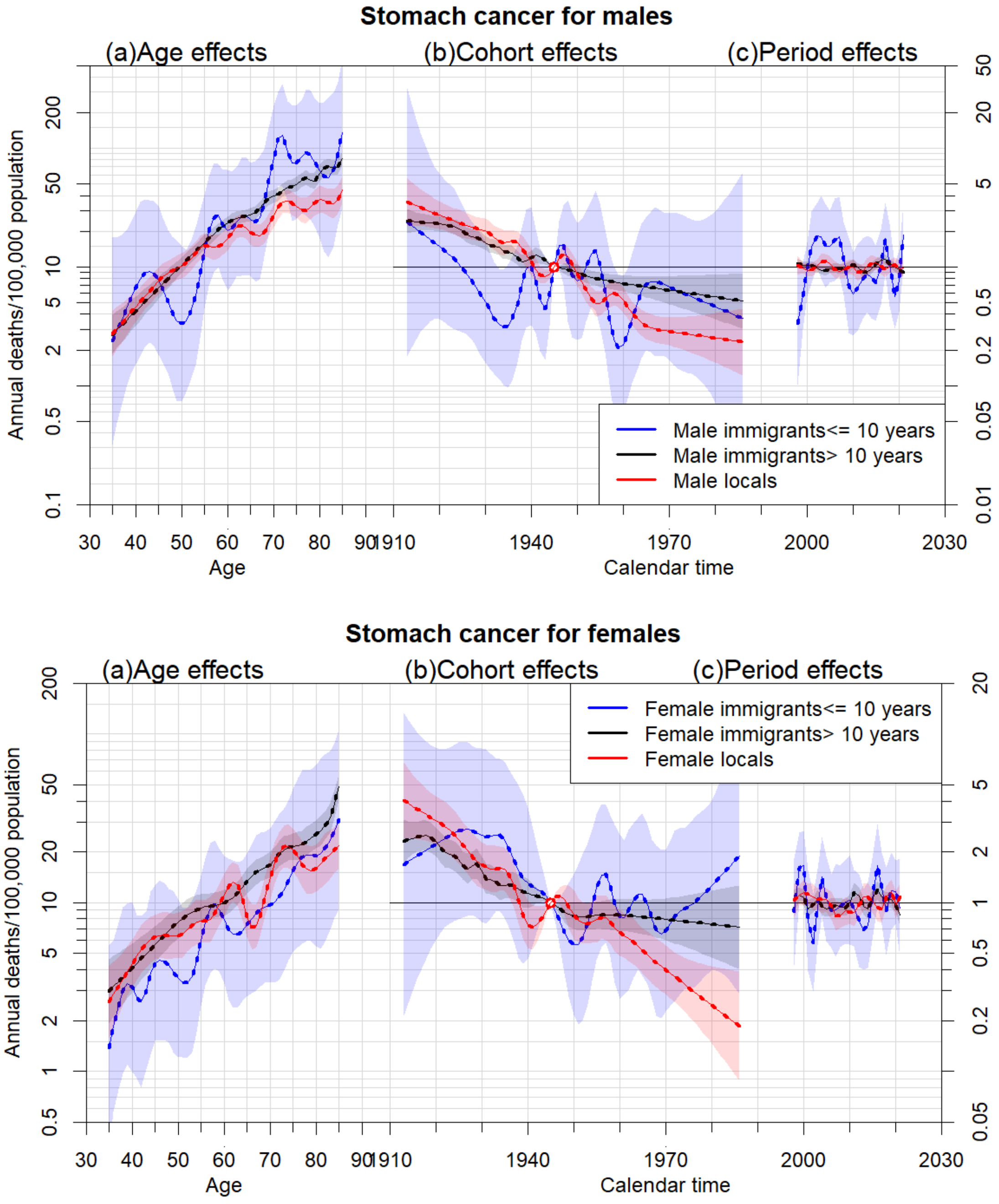


**eFigure 1(d)**. Parameter estimates of age (a), cohort (b) and period (c) effects based on an age-period-cohort model of male and female stomach cancer mortality rates by immigrant groups: locals, immigrants stay in Hong Kong for more than 10 years and immigrants stay in Hong Kong for less than or equal to 10 years. Age effect was assessed by mortality (left axis). Cohort and period effects were assessed by relative risk (right axis), 95% confidence intervals are shown as shaded bands.



For peer review only - http://bmjopen.bmj.com/site/about/guidelines.xhtml

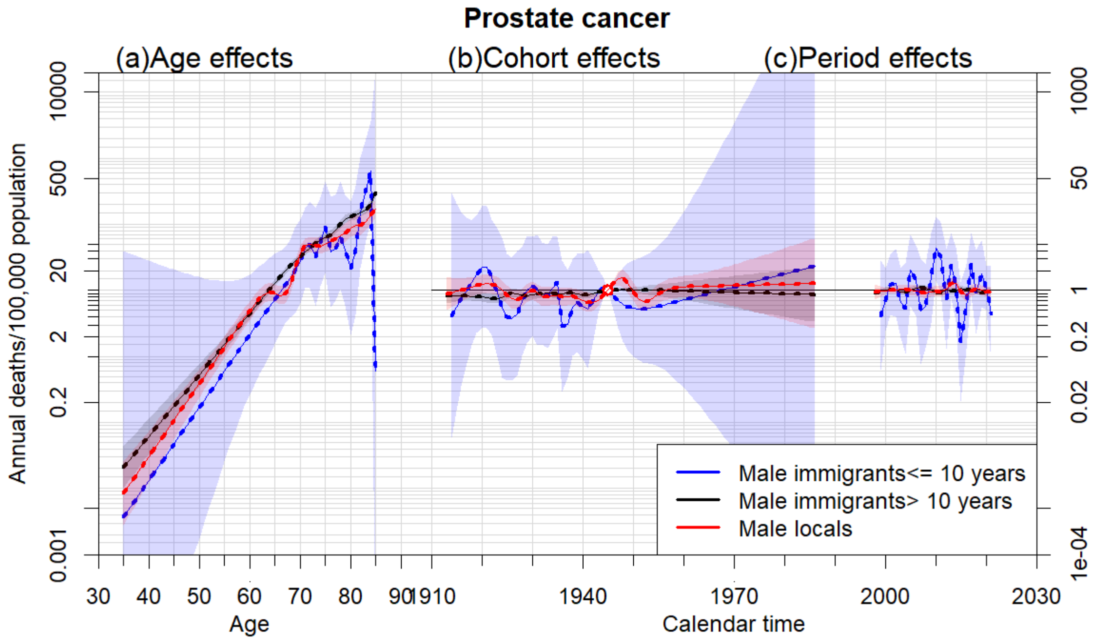


**eFigure 1(e)**. Parameter estimates of age (a), cohort (b) and period (c) effects based on an age-period-cohort model of male prostate cancer mortality rates by immigrant groups: locals, immigrants stay in Hong Kong for more than 10 years and immigrants stay in Hong Kong for less than or equal to 10 years. Age effect was assessed by mortality (left axis). Cohort and period effects were assessed by relative risk (right axis), 95% confidence intervals are shown as shaded bands.



For peer review only - http://bmjopen.bmj.com/site/about/guidelines.xhtml

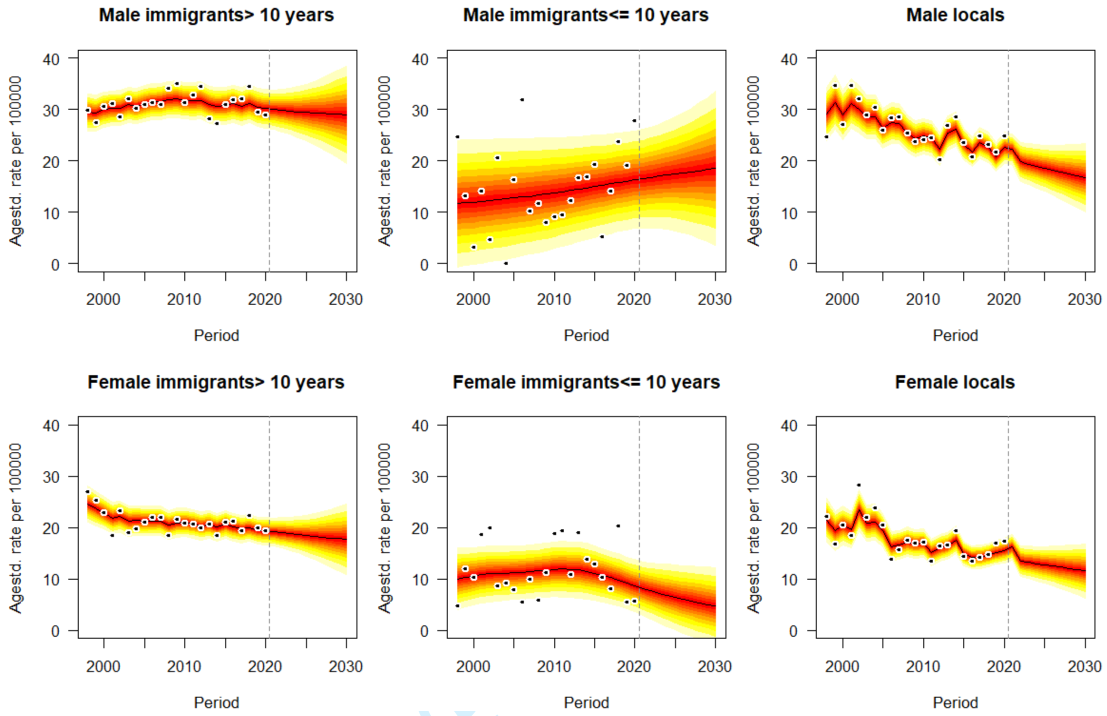


**eFigure 2(a).** Projections of colon cancer mortality rates by gender and immigrant status from 2022 to 2030. Observations are shown as dots with the predictive distribution between the 5% and 95% quantile, whereby each lighter shade of red represents an additional 10% predictive CI. The predictive mean is shown as black solid line and the vertical dashed line indicates where prediction started.



For peer review only - http://bmjopen.bmj.com/site/about/guidelines.xhtml

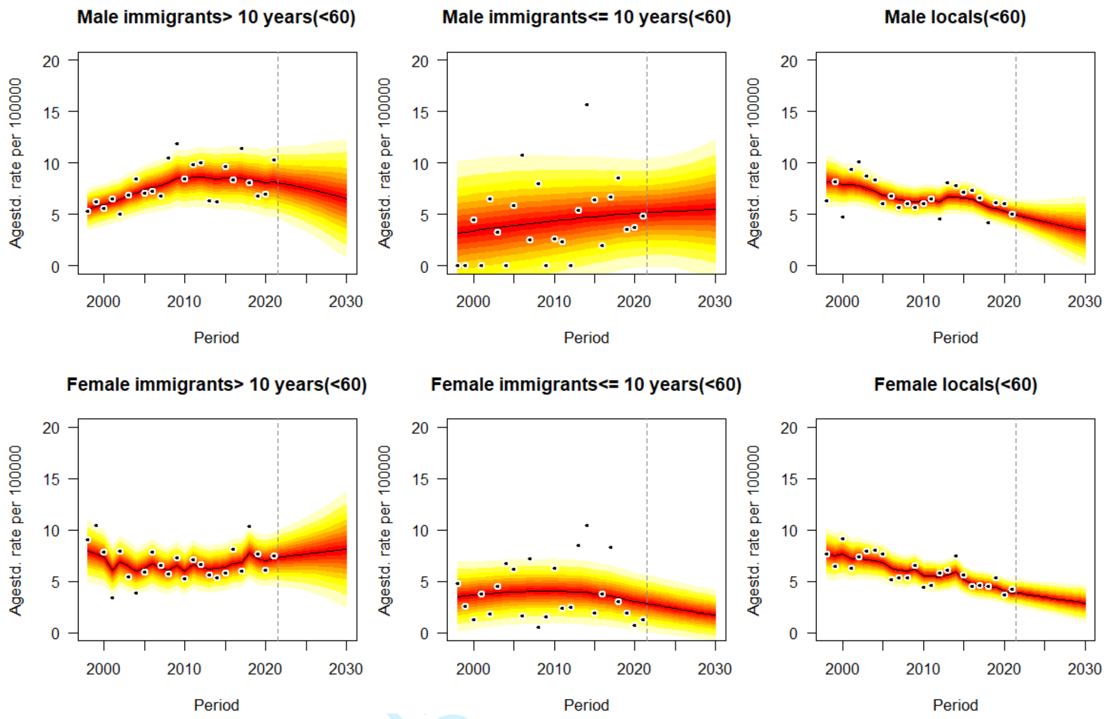


**eFigure 2(b).** Projections of colon cancer mortality rates for the population younger than 60 by gender and immigrant status from 2022 to 2030. Observations are shown as dots with the predictive distribution between the 5% and 95% quantile, whereby each lighter shade of red represents an additional 10% predictive CI. The predictive mean is shown as black solid line and the vertical dashed line indicates where prediction started.

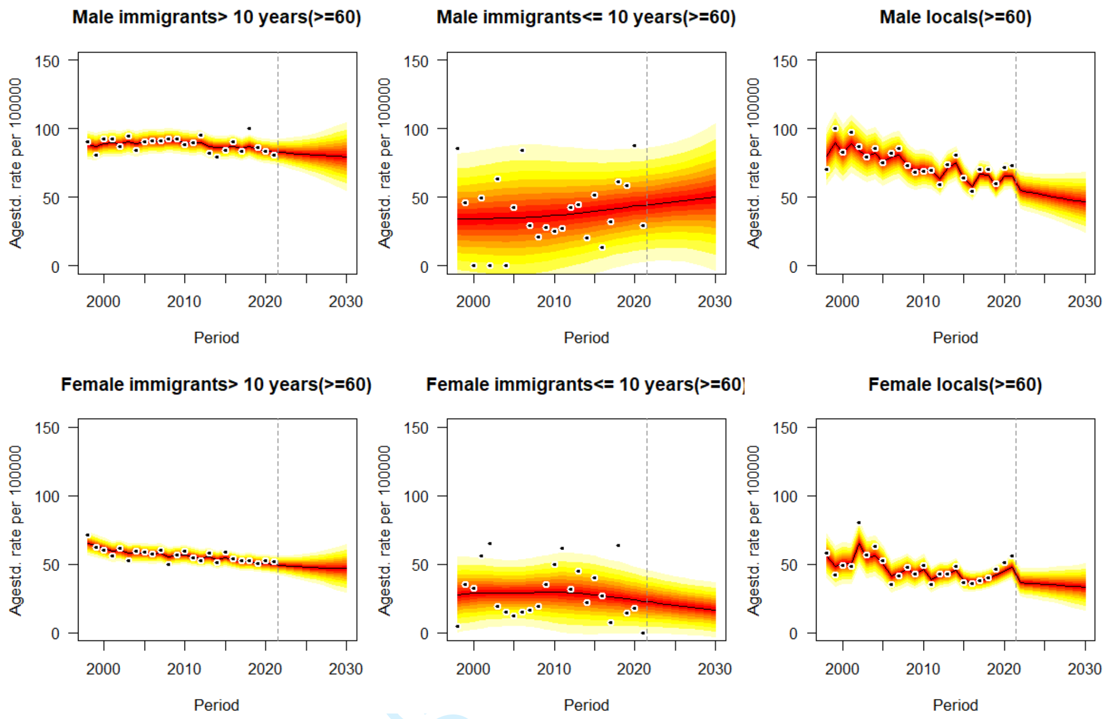


**eFigure 2(c).** Projections of colon cancer mortality rates for the population older than or equal to 60 by gender and immigrant status from 2022 to 2030. Observations are shown as dots with the predictive distribution between the 5% and 95% quantile, whereby each lighter shade of red represents an additional 10% predictive CI. The predictive mean is shown as black solid line and the vertical dashed line indicates where prediction started.



For peer review only - http://bmjopen.bmj.com/site/about/guidelines.xhtml

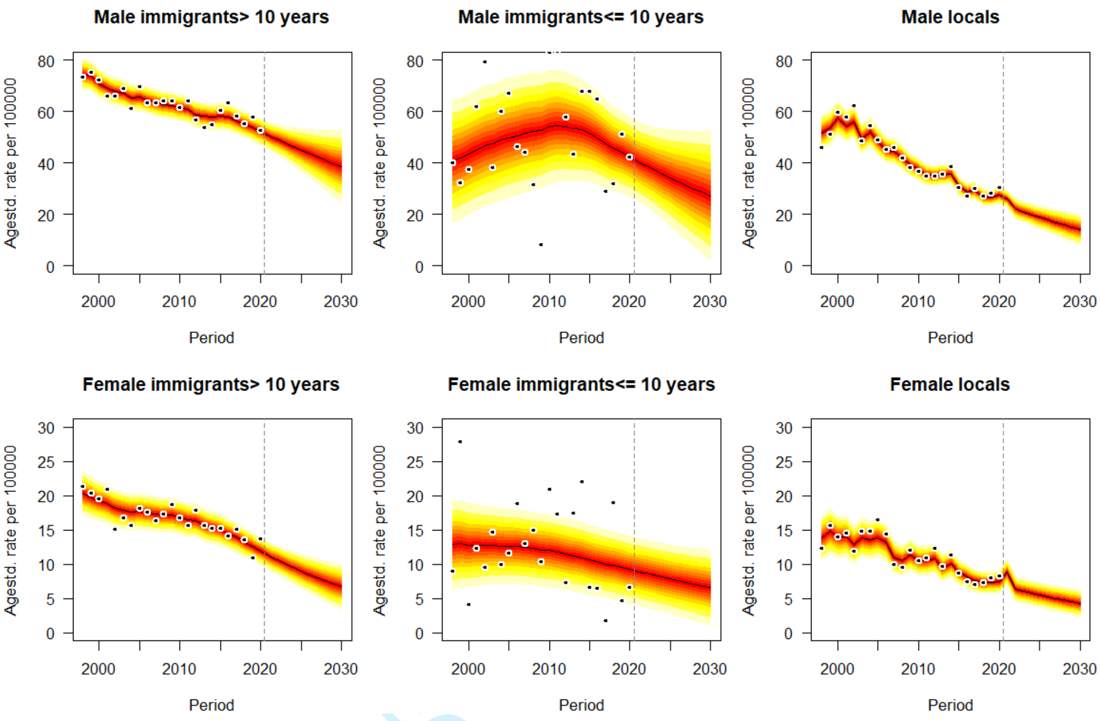


**eFigure 3(a).** Projections of liver cancer mortality rates by gender and immigrant status from 2022 to 2030. Observations are shown as dots with the predictive distribution between the 5% and 95% quantile, whereby each lighter shade of red represents an additional 10% predictive CI. The predictive mean is shown as black solid line and the vertical dashed line indicates where prediction started.

For peer review only



For peer review only - http://bmjopen.bmj.com/site/about/guidelines.xhtml

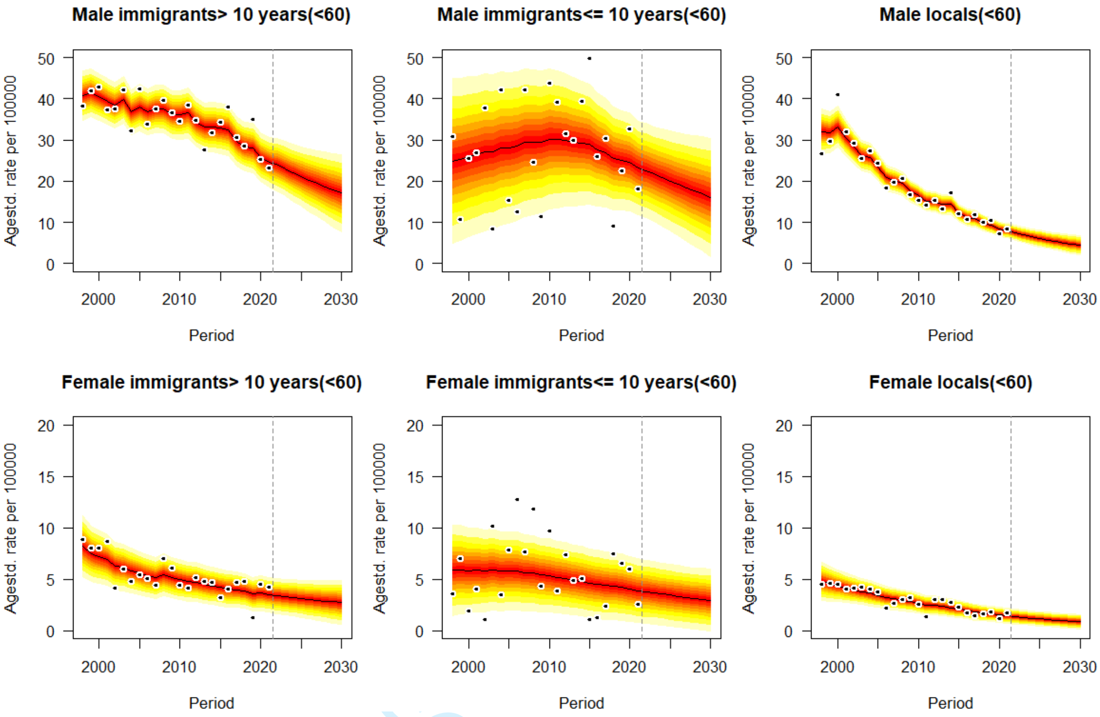


**eFigure 3(b).** Projections of liver cancer mortality rates for the population younger than 60 by gender and immigrant status from 2022 to 2030. Observations are shown as dots with the predictive distribution between the 5% and 95% quantile, whereby each lighter shade of red represents an additional 10% predictive CI. The predictive mean is shown as black solid line and the vertical dashed line indicates where prediction started.



For peer review only - http://bmjopen.bmj.com/site/about/guidelines.xhtml

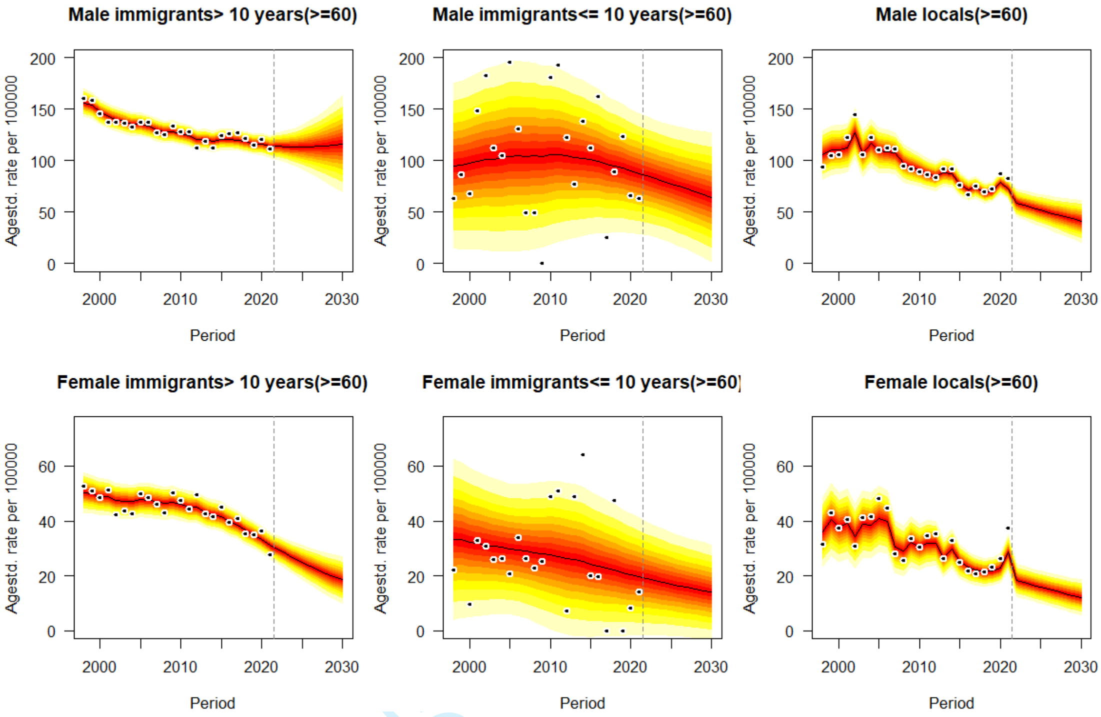


**eFigure 3(c).** Projections of liver cancer mortality rates for the population older than or equal to 60 by gender and immigrant status from 2022 to 2030. Observations are shown as dots with the predictive distribution between the 5% and 95% quantile, whereby each lighter shade of red represents an additional 10% predictive CI. The predictive mean is shown as black solid line and the vertical dashed line indicates where prediction started.



For peer review only - http://bmjopen.bmj.com/site/about/guidelines.xhtml

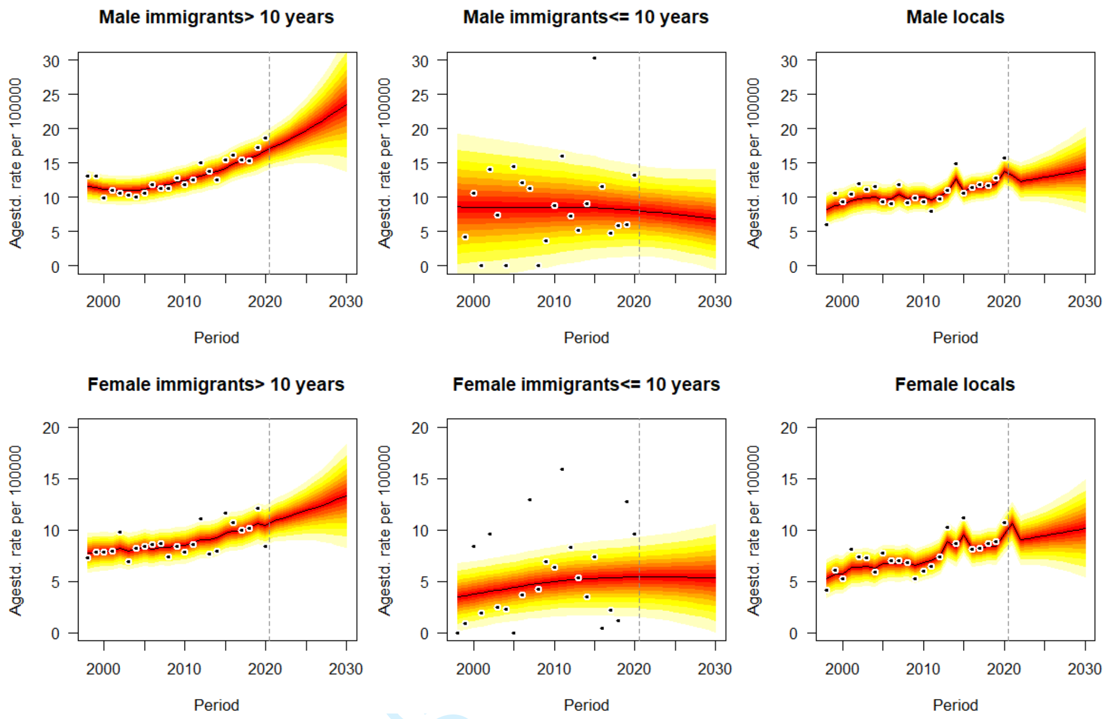


**eFigure 4(a).** Projections of pancreatic cancer mortality rates by gender and immigrant status from 2022 to 2030. Observations are shown as dots with the predictive distribution between the 5% and 95% quantile, whereby each lighter shade of red represents an additional 10% predictive CI. The predictive mean is shown as black solid line and the vertical dashed line indicates where prediction started.



For peer review only - http://bmjopen.bmj.com/site/about/guidelines.xhtml

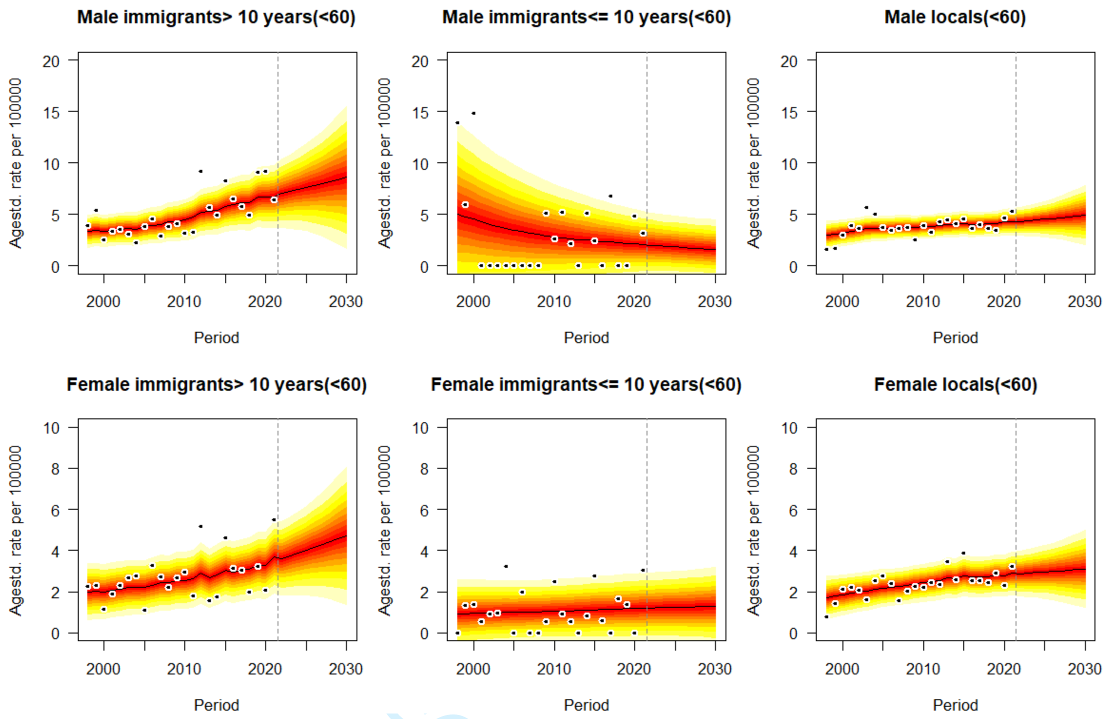


**eFigure 4(b).** Projections of pancreatic cancer mortality rates for the population younger than 60 by gender and immigrant status from 2022 to 2030. Observations are shown as dots with the predictive distribution between the 5% and 95% quantile, whereby each lighter shade of red represents an additional 10% predictive CI. The predictive mean is shown as black solid line and the vertical dashed line indicates where prediction started.



For peer review only - http://bmjopen.bmj.com/site/about/guidelines.xhtml

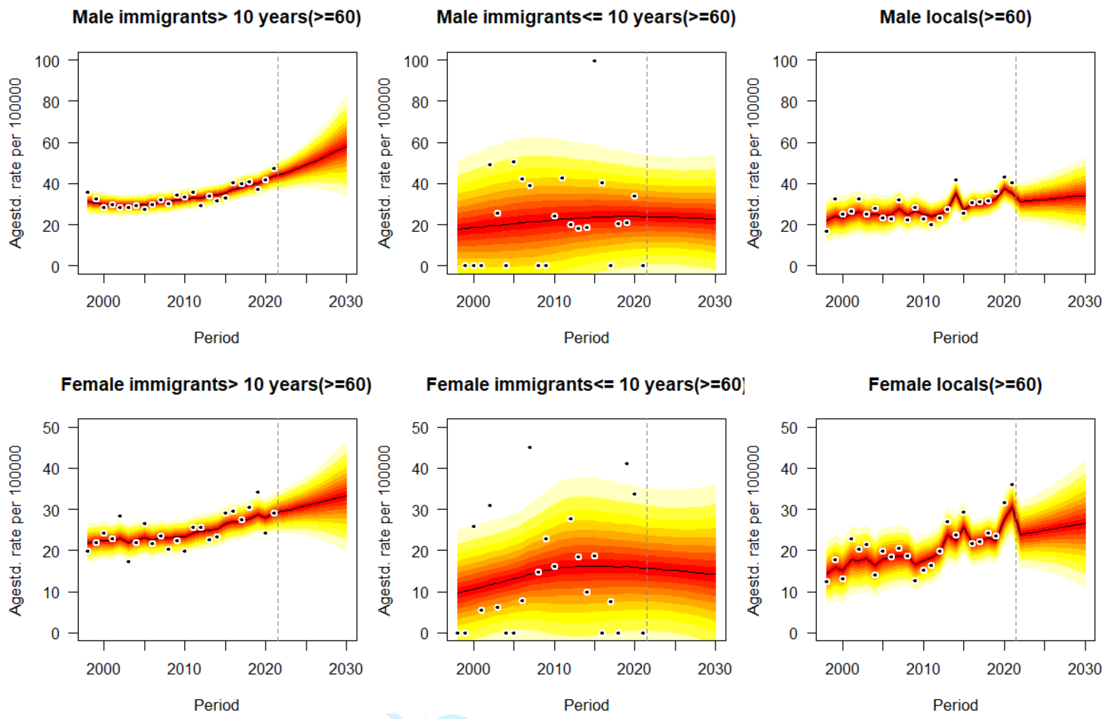


**eFigure 4(c).** Projections of pancreatic cancer mortality rates for the population older than or equal to 60 by gender and immigrant status from 2022 to 2030. Observations are shown as dots with the predictive distribution between the 5% and 95% quantile, whereby each lighter shade of red represents an additional 10% predictive CI. The predictive mean is shown as black solid line and the vertical dashed line indicates where prediction started.



For peer review only - http://bmjopen.bmj.com/site/about/guidelines.xhtml

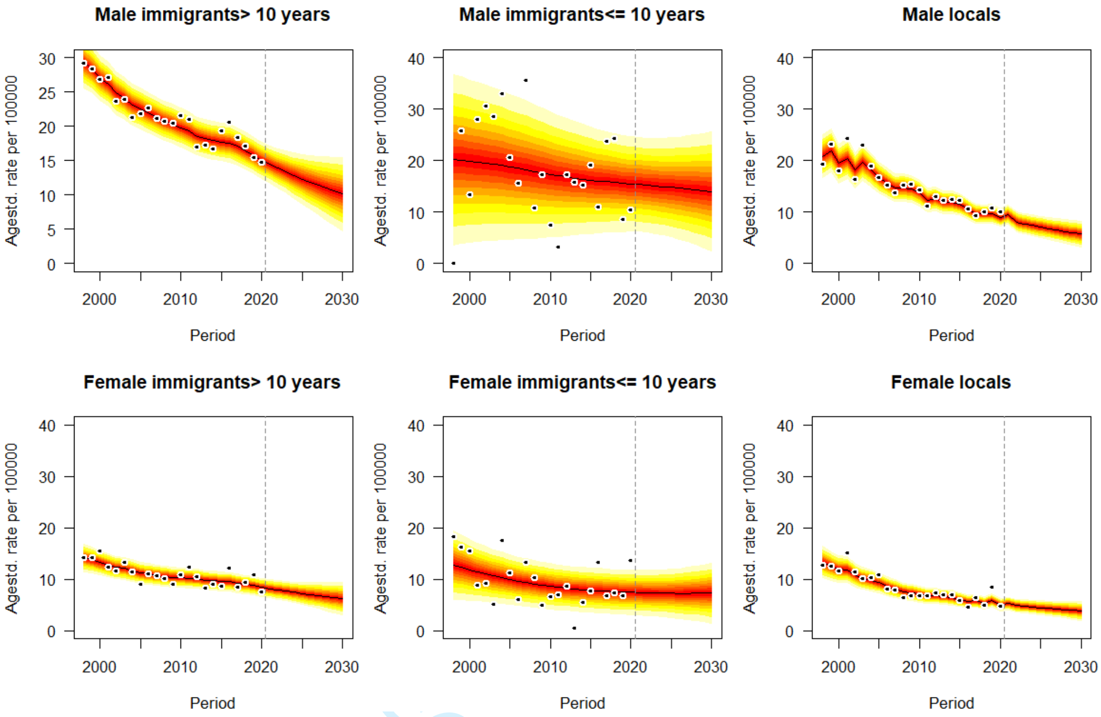


**eFigure 5(a).** Projections of stomach cancer mortality rates by gender and immigrant status from 2022 to 2030. Observations are shown as dots with the predictive distribution between the 5% and 95% quantile, whereby each lighter shade of red represents an additional 10% predictive CI. The predictive mean is shown as black solid line and the vertical dashed line indicates where prediction started.



For peer review only - http://bmjopen.bmj.com/site/about/guidelines.xhtml

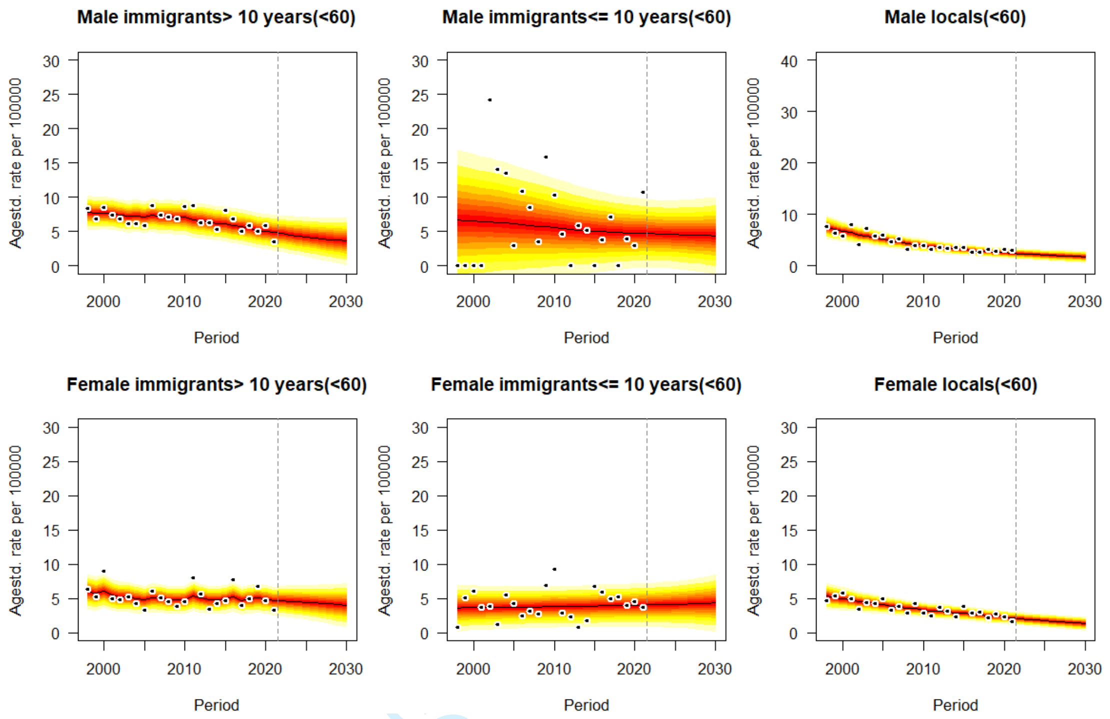


**eFigure 5(b).** Projections of stomach cancer mortality rates for the population younger than 60 by gender and immigrant status from 2022 to 2030. Observations are shown as dots with the predictive distribution between the 5% and 95% quantile, whereby each lighter shade of red represents an additional 10% predictive CI. The predictive mean is shown as black solid line and the vertical dashed line indicates where prediction started.



For peer review only - http://bmjopen.bmj.com/site/about/guidelines.xhtml

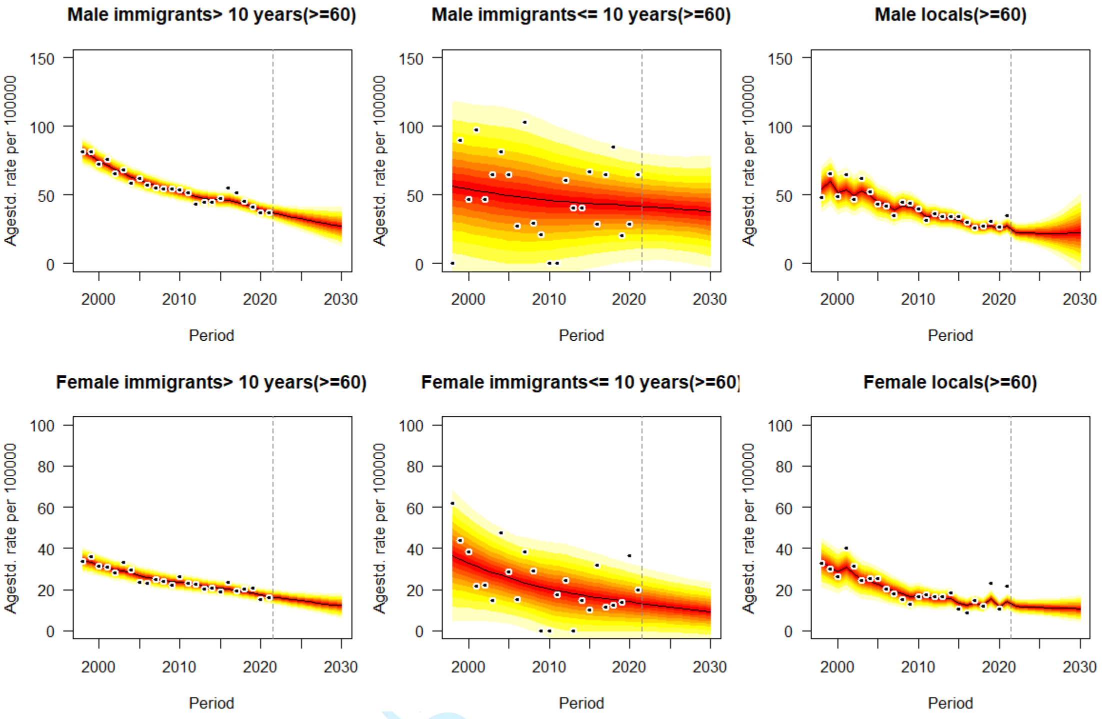


**eFigure 5(c).** Projections of stomach cancer mortality rates for the population older than or equal to 60 by gender and immigrant status from 2022 to 2030. Observations are shown as dots with the predictive distribution between the 5% and 95% quantile, whereby each lighter shade of red represents an additional 10% predictive CI. The predictive mean is shown as black solid line and the vertical dashed line indicates where prediction started.

For peer review only

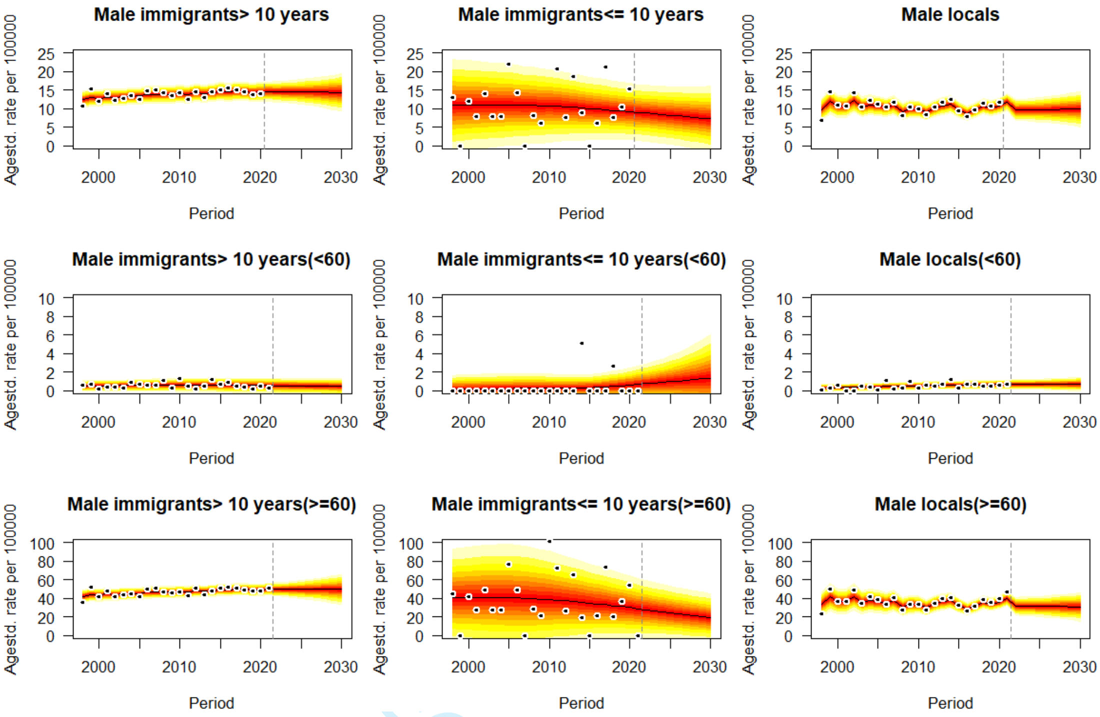


**eFigure 6.** Projections of prostate cancer mortality rates for males by immigrant status and age groups (less than, greater than or equal to 60 years old) from 2022 to 2030. Observations are shown as dots with the predictive distribution between the 5% and 95% quantile, whereby each lighter shade of red represents an additional 10% predictive CI. The predictive mean is shown as black solid line and the vertical dashed line indicates where prediction started.



For peer review only - http://bmjopen.bmj.com/site/about/guidelines.xhtml

| Predictive mean of age-standardized mortality rates of lung cancer per 100,000 population | | | | | | | | | | |
|---|---|---|---|---|---|---|---|---|---|---|
| **Year** | **2021** | **2022** | **2023** | **2024** | **2025** | **2026** | **2027** | **2028** | **2029** | **2030** |
| **Female immigrants >10** | 41.80 (1.27) | 41.34 (1.86) | 40.58 (2.27) | 39.87 (2.75) | 39.19 (3.28) | 38.53 (3.86) | 37.89 (4.46) | 37.26 (5.09) | 36.65 (5.74) | 36.04 (6.4) |
| **Female immigrants ≤ 10** | 23.92 (4.00) | 22.22 (4.67) | 20.56 (5.38) | 19.01 (6.10) | 17.57 (6.80) | 16.24 (7.45) | 15.00 (8.04) | 13.85 (8.56) | 12.79 (9.01) | 11.81 (9.39) |
| **Female locals** | 34.67 (1.76) | 30.22 (3.54) | 30.63 (4.77) | 31.05 (6.38) | 31.48 (8.29) | 31.9 (10.47) | 32.32 (12.87) | 32.73 (15.48) | 33.15 (18.31) | 33.55 (21.33) |
| **Male immigrants >10** | 102.90 (2.43) | 100.18 (4.18) | 97.18 (5.33) | 94.34 (6.72) | 91.71 (8.24) | 89.15 (9.84) | 86.66 (11.47) | 84.19 (13.11) | 81.81 (14.74) | 79.55 (16.37) |
| **Male immigrants ≤10** | 81.26 (9.21) | 79.90 (10.41) | 79.81 (11.82) | 79.72 (13.42) | 79.62 (15.19) | 79.50 (17.09) | 79.32 (19.09) | 79.08 (21.18) | 78.78 (23.32) | 78.41 (25.53) |
| **Male locals** | 60.96 (2.82) | 52.27 (4.86) | 50.83 (5.39) | 49.56 (6.13) | 48.18 (6.97) | 46.64 (7.84) | 45.13 (8.76) | 43.83 (9.76) | 42.67 (10.8) | 41.43 (11.8) |
| **Female immigrants>10(<60y)** | 15.51 (1.12) | 14.51 (1.50) | 13.90 (1.76) | 13.29 (2.04) | 12.71 (2.33) | 12.13 (2.62) | 11.57 (2.91) | 11.02 (3.18) | 10.49 (3.43) | 9.98 (3.68) |
| **Female immigrants ≤ 10(<60y)** | 8.14 (1.91) | 7.79 (1.95) | 7.18(2.23) | 6.62(2.53) | 6.10(2.81) | 5.63(3.08) | 5.19(3.32) | 4.79 (3.53) | 4.42 (3.72) | 4.09 (3.88) |
| **Female locals(<60y)** | 10.25 (0.77) | 9.48 (0.89) | 9.17(1.02) | 8.87(1.16) | 8.57(1.32) | 8.27(1.49) | 7.97(1.65) | 7.68 (1.82) | 7.38 (1.98) | 7.09 (2.13) |
| **Male immigrants>10(<60y)** | 27.81 (2.10) | 26.36 (3.58) | 24.96 (3.94) | 23.64 (4.35) | 22.38 (4.79) | 21.17 (5.23) | 20.03 (5.67) | 18.96 (6.10) | 17.96 (6.51) | 17.03 (6.90) |
| **Male immigrants ≤ 10(<60y)** | 15.01 (2.98) | 13.38 (3.71) | 12.02 (4.17) | 10.79 (4.59) | 9.68 (4.95) | 8.69 (5.24) | 7.79 (5.46) | 6.98 (5.61) | 6.25 (5.69) | 5.59 (5.72) |
| **Male locals(<60y)** | 15.19 (0.78) | 14.45 (1.15) | 14.03 (1.29) | 13.61 (1.46) | 13.14 (1.64) | 12.65 (1.82) | 12.13 (2.01) | 11.55 (2.17) | 10.93 (2.31) | 10.26 (2.43) |
| **Female immigrants >10(≥ 60y)** | 108.85 (4.80) | 107.21 (5.17) | 106.26 (6.24) | 105.52 (7.54) | 104.94 (9.04) | 104.51 (10.72) | 104.21 (12.57) | 104.07 (14.61) | 104.06 (16.78) | 104.16 (19.14) |
| **Female immigrants ≤ 10(≥60y)** | 66.16 (13.25) | 63.84 (15.72) | 59.88 (17.50) | 56.14 (19.31) | 52.60 (21.03) | 49.27 (22.66) | 46.14 (24.16) | 43.20 (25.52) | 40.44 (26.74) | 37.85 (27.81) |
| **Female locals(≥60y)** | 77.33 (9.40) | 76.53 (10.11) | 76.22 (10.85) | 75.94 (11.79) | 75.69 (12.94) | 75.49 (14.28) | 75.32 (15.80) | 75.19 (17.48) | 75.10 (19.33) | 75.03 (21.32) |
| **Male immigrants>10(≥60y)** | 293.56 (9.13) | 289.8 (11.7) | 286.6 (15.19) | 284.28 (19.51) | 282.78 (24.49) | 281.99 (30.07) | 281.88 (36.31) | 282.31 (43.15) | 283.37 (50.66) | 285.03 (58.86) |
| **Male immigrants ≤ 10(≥60y)** | 244.88 (30.29) | 247.01 (36.85) | 251.24 (42.94) | 255.62 (50.06) | 260.14 (58.14) | 264.82 (67.14) | 269.61 (77.01) | 274.52 (87.75) | 279.55 (99.34) | 284.69 (111.81) |
| **Male locals(≥60y)** | 150.75 (16.22) | 146.29 (18.46) | 143.54 (20.58) | 141.84 (23.97) | 140.07 (28.24) | 138.14 (33.39) | 136.65 (39.82) | 136.49 (47.87) | 137.24 (57.47) | 138.26 (68.52) |

**eTable 1.** Predictive means and standard deviations (in brackets) of age-standardized mortality rates of lung cancer per 100,000 population for each gender, age group (less than, greater or equal to 60 years old) and immigrant status from 2022 to 2030. Reported means and standard deviations (in brackets) of age-standardized mortality rates in 2021 are also listed.



For peer review only - http://bmjopen.bmj.com/site/about/guidelines.xhtml

| Predictive mean of age-standardized mortality rates of colon cancer per 100,000 population | | | | | | | | | | |
|---|---|---|---|---|---|---|---|---|---|---|
| **Year** | **2021** | **2022** | **2023** | **2024** | **2025** | **2026** | **2027** | **2028** | **2029** | **2030** |
| **Female immigrants >10** | 20.03 (0.95) | 18.95 (1.13) | 18.77 (1.37) | 18.59 (1.66) | 18.42 (1.98) | 18.27 (2.33) | 18.12 (2.71) | 17.98 (3.11) | 17.85 (3.53) | 17.73 (3.96) |
| **Female immigrants ≤ 10** | 8.11 (2.19) | 7.70 (2.51) | 7.25 (2.81) | 6.82 (3.11) | 6.42 (3.37) | 6.03 (3.61) | 5.67 (3.83) | 5.33 (4.01) | 5.01 (4.17) | 4.71 (4.31) |
| **Female locals** | 13.77 (1.30) | 13.47 (1.61) | 13.24 (1.72) | 13.01 (1.87) | 12.77 (2.04) | 12.53 (2.24) | 12.29 (2.46) | 12.06 (2.68) | 11.82 (2.92) | 11.59 (3.16) |
| **Male immigrants >10** | 31.22 (1.28) | 29.82 (1.46) | 29.66 (1.79) | 29.52 (2.19) | 29.41 (2.63) | 29.30 (3.11) | 29.21 (3.64) | 29.14 (4.19) | 29.06 (4.78) | 28.98 (5.39) |
| **Male immigrants ≤10** | 15.47 (2.14) | 16.77 (3.77) | 17.02 (4.18) | 17.23 (4.64) | 17.45 (5.14) | 17.67 (5.69) | 17.88 (6.27) | 18.09 (6.91) | 18.31 (7.56) | 18.50 (8.26) |
| **Male locals** | 21.28 (1.38) | 19.81 (2.07) | 19.39 (2.22) | 18.97 (2.42) | 18.57 (2.61) | 18.18 (2.85) | 17.81 (3.12) | 17.43 (3.40) | 17.06 (3.71) | 16.71 (4.03) |
| **Female immigrants >10(<60y)** | 7.09 (0.99) | 7.36 (1.12) | 7.46 (1.28) | 7.56 (1.46) | 7.65 (1.68) | 7.74 (1.92) | 7.83 (2.19) | 7.92 (2.48) | 8.01 (2.79) | 8.09 (3.13) |
| **Female immigrants ≤ 10(<60y)** | 3.11 (0.67) | 2.82 (0.86) | 2.65 (0.91) | 2.51 (0.97) | 2.36 (1.02) | 2.22 (1.07) | 2.08 (1.11) | 1.95 (1.14) | 1.83 (1.18) | 1.72 (1.22) |
| **Female locals(<60y)** | 4.10 (0.41) | 3.87 (0.50) | 3.73 (0.54) | 3.61 (0.59) | 3.47 (0.65) | 3.34 (0.70) | 3.22 (0.76) | 3.11 (0.82) | 2.99 (0.88) | 2.88 (0.94) |
| **Male immigrants >10(<60y)** | 8.29 (0.91) | 7.98 (1.17) | 7.85 (1.38) | 7.71 (1.60) | 7.54 (1.83) | 7.36 (2.08) | 7.17(2.32) | 6.97(2.57) | 6.76(2.81) | 6.55(3.05) |
| **Male immigrants ≤ 10(<60y)** | 5.03 (1.44) | 5.18 (1.58) | 5.22 (1.75) | 5.26 (1.93) | 5.30 (2.14) | 5.34 (2.36) | 5.38(2.59) | 5.43(2.84) | 5.47(3.11) | 5.51(3.38) |
| **Male locals(<60y)** | 5.14 (0.43) | 4.88 (0.63) | 4.66 (0.79) | 4.46 (0.96) | 4.26 (1.13) | 4.08 (1.31) | 3.91(1.48) | 3.73(1.65) | 3.57(1.82) | 3.42(1.97) |
| **Female immigrants >10(≥60y)** | 52.16 (2.59) | 49.21 (2.99) | 48.70 (3.56) | 48.26 (4.26) | 47.87 (5.05) | 47.54 (5.94) | 47.26 (6.90) | 47.05 (7.94) | 46.91 (9.06) | 46.81 (10.26) |
| **Female immigrants ≤ 10(≥60y)** | 24.01 (5.83) | 22.44 (6.56) | 21.69 (6.96) | 20.95 (7.38) | 20.23 (7.80) | 19.52 (8.23) | 18.84 (8.66) | 18.17 (9.08) | 17.51 (9.49) | 16.86 (9.90) |
| **Female locals(≥60y)** | 37.42 (5.31) | 36.69 (5.74) | 36.29 (6.06) | 35.87 (6.46) | 35.46 (6.95) | 35.04 (7.5) | 34.61 (8.12) | 34.19 (8.79) | 33.77 (9.51) | 33.34 (10.27) |
| **Male immigrants >10(≥60y)** | 84.17 (3.55) | 82.72 (4.09) | 82.16 (4.95) | 81.64 (5.97) | 81.19 (7.12) | 80.81 (8.39) | 80.47 (9.77) | 80.15 (11.24) | 79.85 (12.81) | 79.56 (14.45) |
| **Male immigrants ≤ 10(≥60y)** | 43.25 (11.07) | 44.93 (13.09) | 45.62 (14.52) | 46.30 (16.09) | 46.96 (17.80) | 47.61 (19.64) | 48.25 (21.62) | 48.88 (23.73) | 49.51 (25.97) | 50.13 (28.34) |
| **Male locals(≥60y)** | 55.79 (6.86) | 54.89 (7.65) | 53.75 (8.03) | 52.63 (8.52) | 51.54 (9.12) | 50.47 (9.8) | 49.43 (10.55) | 48.42 (11.37) | 47.42 (12.25) | 46.44 (13.16) |

**eTable 2.** Predictive means and standard deviations (in brackets) of age-standardized mortality rates of colon cancer per 100,000 population for each gender, age group (less than, greater or equal to 60 years old) and immigrant status from 2022 to 2030. Reported means and standard deviations (in brackets) of age-standardized mortality rates in 2021 are also listed.



For peer review only - http://bmjopen.bmj.com/site/about/guidelines.xhtml

| Predictive mean of age-standardized mortality rates of liver cancer per 100,000 population | | | | | | | | | | |
|---|---|---|---|---|---|---|---|---|---|---|
| **Year** | **2021** | **2022** | **2023** | **2024** | **2025** | **2026** | **2027** | **2028** | **2029** | **2030** |
| **Female immigrants >10** | 11.34 (0.66) | 10.68 (0.71) | 10.09 (0.85) | 9.54 (1.01) | 9.01 (1.16) | 8.50 (1.31) | 8.02(1.45) | 7.57(1.59) | 7.14(1.72) | 6.74(1.83) |
| **Female immigrants ≤10** | 9.15 (1.55) | 8.66 (1.82) | 8.38 (1.95) | 8.11 (2.08) | 7.84 (2.22) | 7.58 (2.36) | 7.32(2.49) | 7.07(2.63) | 6.82(2.76) | 6.58(2.88) |
| **Female locals** | 6.72 (0.69) | 6.36 (0.88) | 6.08 (0.90) | 5.81 (0.93) | 5.53 (0.97) | 5.26 (1.01) | 5.01(1.06) | 4.77(1.11) | 4.53(1.15) | 4.3(1.21) |
| **Male immigrants >10** | 52.17 (1.78) | 49.22 (2.36) | 47.76 (2.93) | 46.35 (3.59) | 45.01 (4.31) | 43.67 (5.05) | 42.37 (5.81) | 41.1(6.56) | 39.89 (7.33) | 38.71 (8.08) |
| **Male immigrants ≤10** | 42.33 (5.87) | 39.03 (6.49) | 37.39 (7.47) | 35.81 (8.51) | 34.26 (9.58) | 32.76 (10.63) | 31.31 (11.65) | 29.91 (12.62) | 28.56 (13.54) | 27.25 (14.40) |
| **Male locals** | 24.22 (1.77) | 22.16 (2.09) | 21.02 (2.22) | 19.91 (2.39) | 18.85 (2.58) | 17.83 (2.79) | 16.85 (3.03) | 15.92 (3.21) | 15.03 (3.40) | 14.18 (3.59) |
| **Female immigrants >10(<60y)** | 3.62 (0.45) | 3.39 (0.52) | 3.29 (0.57) | 3.20 (0.63) | 3.12 (0.69) | 3.04 (0.75) | 2.96(0.82) | 2.89(0.89) | 2.82(0.96) | 2.75(1.03) |
| **Female immigrants ≤ 10(<60y)** | 4.10 (0.79) | 3.81 (0.91) | 3.69 (0.96) | 3.57 (1.02) | 3.46 (1.08) | 3.36 (1.15) | 3.25(1.22) | 3.15(1.29) | 3.06(1.36) | 2.97(1.43) |
| **Female locals(<60y)** | 1.50 (0.13) | 1.37 (0.2) | 1.29 (0.21) | 1.22 (0.23) | 1.16 (0.24) | 1.10 (0.26) | 1.04(0.27) | 0.99(0.29) | 0.94(0.30) | 0.89(0.31) |
| **Male immigrants >10(<60y)** | 26.32 (2.11) | 24.04 (2.35) | 23.02 (2.63) | 22.05 (2.94) | 21.13 (3.27) | 20.25 (3.61) | 19.41 (3.95) | 18.62 (4.30) | 17.86 (4.64) | 17.14 (4.98) |
| **Male immigrants ≤ 10(<60y)** | 25.52 (2.99) | 22.56 (3.96) | 21.71 (4.44) | 20.87 (4.94) | 20.04 (5.45) | 19.22 (5.95) | 18.42 (6.45) | 17.63 (6.91) | 16.86 (7.36) | 16.11 (7.78) |
| **Male locals(<60y)** | 8.25 (0.69) | 7.47 (0.74) | 6.97 (0.79) | 6.52 (0.86) | 6.11 (0.93) | 5.73 (1.01) | 5.38(1.08) | 5.04(1.15) | 4.73(1.21) | 4.44(1.27) |
| **Female immigrants >10(≥ 60y)** | 33.67 (1.88) | 29.63 (2.01) | 27.99 (2.36) | 26.42 (2.75) | 24.92 (3.14) | 23.49 (3.52) | 22.13 (3.88) | 20.85 (4.23) | 19.64 (4.55) | 18.50 (4.85) |
| **Female immigrants ≤10(≥ 60y)** | 21.72 (5.11) | 19.08 (5.81) | 18.38 (6.14) | 17.71 (6.48) | 17.03 (6.83) | 16.39 (7.16) | 15.76 (7.49) | 15.16 (7.80) | 14.57 (8.11) | 14.01 (8.39) |
| **Female locals(≥60y)** | 20.63 (3.03) | 18.41 (3.23) | 17.55 (3.26) | 16.72 (3.32) | 15.91 (3.40) | 15.11 (3.49) | 14.34 (3.59) | 13.59 (3.69) | 12.87 (3.81) | 12.17 (3.93) |
| **Male immigrants >10(≥ 60y)** | 115.39 (4.54) | 113.96 (5.95) | 113.43 (7.65) | 113.17 (9.70) | 113.16 (12.04) | 113.37 (14.66) | 113.79 (17.56) | 114.39 (20.73) | 115.19 (24.18) | 116.17 (27.91) |
| **Male immigrants ≤10(≥ 60y)** | 88.61 (15.58) | 85.14 (18.85) | 82.59 (20.6) | 80.02 (22.44) | 77.42 (24.34) | 74.83 (26.24) | 72.23 (28.12) | 69.64 (29.94) | 67.07 (31.70) | 64.52 (33.38) |
| **Male locals(≥60y)** | 62.88 (5.97) | 58.95 (7.91) | 56.51 (8.20) | 54.14 (8.61) | 51.84 (9.12) | 49.61 (9.70) | 47.46 (10.33) | 45.38 (11.01) | 43.38 (11.68) | 41.45 (12.36) |

**eTable 3.** Predictive means and standard deviations (in brackets) of age-standardized mortality rates of liver cancer per 100,000 population for each gender, age group (less than, greater or equal to 60 years old) and immigrant status from 2022 to 2030. Reported means and standard deviations (in brackets) of age-standardized mortality rates in 2021 are also listed.



For peer review only - http://bmjopen.bmj.com/site/about/guidelines.xhtml

| Predictive mean of age-standardized mortality rates of pancreatic cancer per 100,000 population | | | | | | | | | | |
|---|---|---|---|---|---|---|---|---|---|---|
| Year | 2021 | 2022 | 2023 | 2024 | 2025 | 2026 | 2027 | 2028 | 2029 | 2030 |
| Female immigrants >10 | 10.89 (0.62) | 11.11 (0.75) | 11.36 (0.91) | 11.61 (1.09) | 11.87 (1.31) | 12.14 (1.56) | 12.42 (1.84) | 12.71 (2.15) | 13.01 (2.48) | 13.3(2.85) |
| Female immigrants ≤ 10 | 5.51 (1.44) | 5.44 (1.56) | 5.44 (1.69) | 5.43(1.84) | 5.42(1.99) | 5.41(2.15) | 5.39(2.32) | 5.36(2.49) | 5.34(2.66) | 5.31(2.84) |
| Female locals | 8.79 (1.10) | 9.01 (1.22) | 9.15 (1.34) | 9.29(1.48) | 9.43(1.64) | 9.57(1.83) | 9.71(2.05) | 9.85(2.28) | 9.99(2.54) | 10.14 (2.83) |
| Male immigrants >10 | 16.30 (0.98) | 17.87 (1.19) | 18.48 (1.49) | 19.11 (1.87) | 19.78 (2.32) | 20.47 (2.83) | 21.18 (3.42) | 21.92 (4.07) | 22.69 (4.81) | 23.49 (5.61) |
| Male immigrants ≤10 | 8.10 (2.02) | 7.87 (2.37) | 7.76 (2.53) | 7.64(2.70) | 7.51(2.87) | 7.38(3.05) | 7.24(3.23) | 7.09(3.41) | 6.95(3.58) | 6.81(3.75) |
| Male locals | 11.97 (1.26) | 12.29 (1.49) | 12.49 (1.64) | 12.69 (1.83) | 12.91 (2.06) | 13.11 (2.33) | 13.33 (2.63) | 13.55 (2.97) | 13.78 (3.34) | 14.02 (3.74) |
| Female immigrants >10(<60y) | 3.47 (0.33) | 3.62 (0.57) | 3.74 (0.66) | 3.87(0.77) | 4.01(0.89) | 4.14(1.02) | 4.28(1.18) | 4.42(1.34) | 4.57(1.53) | 4.72(1.73) |
| Female immigrants ≤ 10(<60y) | 1.12 (0.33) | 1.21 (0.48) | 1.22 (0.52) | 1.23(0.56) | 1.24(0.61) | 1.25(0.66) | 1.26(0.71) | 1.26(0.77) | 1.27(0.83) | 1.28(0.89) |
| Female locals(<60y) | 2.76 (0.27) | 2.88 (0.36) | 2.91 (0.41) | 2.93(0.48) | 2.96(0.55) | 2.99(0.63) | 3.02(0.71) | 3.04(0.81) | 3.07(0.90) | 3.10(1.01) |
| Male immigrants >10(<60y) | 6.88 (0.98) | 7.05 (1.11) | 7.24 (1.32) | 7.43(1.56) | 7.62(1.84) | 7.82(2.16) | 8.01(2.50) | 8.21(2.88) | 8.40(3.30) | 8.61(3.75) |
| Male immigrants ≤ 10(<60y) | 2.20 (0.71) | 2.01 (0.85) | 1.95 (0.91) | 1.9(0.94) | 1.84(0.99) | 1.79(1.04) | 1.74(1.09) | 1.69(1.14) | 1.64(1.19) | 1.60(1.24) |
| Male locals(<60y) | 4.16 (0.35) | 4.33 (0.48) | 4.41 (0.57) | 4.46(0.68) | 4.53(0.81) | 4.61(0.94) | 4.69(1.09) | 4.77(1.26) | 4.85(1.44) | 4.93(1.63) |
| Female immigrants >10(≥60y) | 28.58 (1.83) | 29.45 (2.11) | 29.91 (2.54) | 30.38 (3.06) | 30.85 (3.66) | 31.33 (4.33) | 31.81 (5.08) | 32.29 (5.91) | 32.78 (6.79) | 33.27 (7.74) |
| Female immigrants ≤ 10(≥60y) | 16.79 (5.29) | 15.65 (6.08) | 15.49 (6.71) | 15.33 (7.36) | 15.16 (8.03) | 14.97 (8.73) | 14.79 (9.43) | 14.59 (10.14) | 14.39 (10.86) | 14.19 (11.58) |
| Female locals(≥60y) | 22.80 (4.23) | 23.85 (4.46) | 24.21 (4.81) | 24.56 (5.23) | 24.91 (5.73) | 25.25 (6.30) | 25.58 (6.95) | 25.90 (7.67) | 26.22 (8.47) | 26.54 (9.34) |
| Male immigrants >10(≥60y) | 42.70 (2.55) | 44.36 (3.02) | 45.85 (3.76) | 47.41 (4.69) | 49.04 (5.78) | 50.73 (7.05) | 52.48 (8.50) | 54.28 (10.13) | 56.16 (11.95) | 58.11 (13.98) |
| Male immigrants ≤ 10(≥60y) | 24.68 (8.21) | 23.96 (9.01) | 23.87 (9.74) | 23.75 (10.52) | 23.61 (11.33) | 23.45 (12.17) | 23.28 (13.04) | 23.09 (13.93) | 22.89 (14.83) | 22.68 (15.75) |
| Male locals(≥60y) | 30.10 (4.68) | 31.17 (5.22) | 31.55 (5.63) | 31.93 (6.14) | 32.30 (6.75) | 32.66 (7.45) | 33.01 (8.23) | 33.35 (9.11) | 33.69 (10.08) | 34.03 (11.12) |

**eTable 4.** Predictive means and standard deviations (in brackets) of age-standardized mortality rates of pancreatic cancer per 100,000 population for each gender, age group (less than, greater or equal to 60 years old) and immigrant status from 2022 to 2030. Reported means and standard deviations (in brackets) of age-standardized mortality rates in 2021 are also listed.



For peer review only - http://bmjopen.bmj.com/site/about/guidelines.xhtml

| Predictive mean of age-standardized mortality rates of stomach cancer per 100,000 population | | | | | | | | | | |
|---|---|---|---|---|---|---|---|---|---|---|
| Year | 2021 | 2022 | 2023 | 2024 | 2025 | 2026 | 2027 | 2028 | 2029 | 2030 |
| Female immigrants >10 | 8.20 (0.55) | 7.95 (0.62) | 7.71 (0.74) | 7.47 (0.87) | 7.25 (1.01) | 7.03 (1.15) | 6.83 (1.29) | 6.62 (1.43) | 6.43 (1.57) | 6.24 (1.71) |
| Female immigrants ≤ 10 | 7.51 (1.44) | 7.36 (1.56) | 7.33 (1.69) | 7.30 (1.85) | 7.28 (2.01) | 7.27 (2.20) | 7.27 (2.40) | 7.28 (2.61) | 7.31 (2.84) | 7.33 (3.09) |
| Female locals | 5.26 (0.40) | 4.91 (0.52) | 4.75 (0.57) | 4.61 (0.63) | 4.47 (0.71) | 4.34 (0.77) | 4.21 (0.84) | 4.08 (0.91) | 3.95 (0.99) | 3.83 (1.06) |
| Male immigrants >10 | 15.22 (0.64) | 13.89 (0.97) | 13.34(1.21) | 12.81 (1.46) | 12.31 (1.73) | 11.83 (1.99) | 11.38 (2.26) | 10.95 (2.51) | 10.54 (2.76) | 10.15 (3.01) |
| Male immigrants ≤10 | 15.83 (3.04) | 15.21 (3.38) | 15.07 (3.67) | 14.93 (3.98) | 14.79 (4.31) | 14.64 (4.65) | 14.51 (5.02) | 14.35 (5.39) | 14.19 (5.78) | 14.03 (6.17) |
| Male locals | 8.14 (0.89) | 8.07 (0.99) | 7.73 (1.03) | 7.41(1.07) | 7.10 (1.13) | 6.81 (1.19) | 6.51 (1.26) | 6.23 (1.33) | 5.97 (1.39) | 5.71 (1.46) |
| Female immigrants >10(<60y) | 4.81 (0.56) | 4.69 (0.79) | 4.62 (0.87) | 4.55 (0.96) | 4.47 (1.07) | 4.39 (1.17) | 4.31 (1.29) | 4.22 (1.41) | 4.13 (1.52) | 4.03 (1.64) |
| Female immigrants ≤ 10(<60y) | 3.89 (0.80) | 4.08 (0.93) | 4.10 (1.03) | 4.13 (1.14) | 4.17 (1.27) | 4.21 (1.41) | 4.24 (1.55) | 4.28 (1.70) | 4.32 (1.87) | 4.36 (2.05) |
| Female locals(<60y) | 2.28 (0.21) | 2.08 (0.27) | 1.98 (0.29) | 1.88 (0.32) | 1.79 (0.35) | 1.71 (0.37) | 1.61 (0.41) | 1.53 (0.43) | 1.44 (0.45) | 1.37 (0.47) |
| Male immigrants >10(<60y) | 4.94 (0.57) | 4.71 (0.79) | 4.55 (0.89) | 4.41 (0.99) | 4.25 (1.10) | 4.12 (1.21) | 3.98 (1.32) | 3.86 (1.43) | 3.74 (1.54) | 3.63 (1.65) |
| Male immigrants ≤ 10(<60y) | 4.81 (1.31) | 4.70 (1.42) | 4.66 (1.55) | 4.63 (1.69) | 4.59 (1.83) | 4.55 (1.99) | 4.52 (2.15) | 4.48 (2.32) | 4.44 (2.50) | 4.41 (2.68) |
| Male locals(<60y) | 2.48 (0.21) | 2.37 (0.29) | 2.28 (0.32) | 2.21 (0.35) | 2.12 (0.38) | 2.04 (0.42) | 1.97 (0.45) | 1.91 (0.49) | 1.83 (0.52) | 1.77(0.55) |
| Female immigrants >10(≥60y) | 17.80 (1.04) | 16.23 (1.26) | 15.65 (1.47) | 15.08 (1.70) | 14.55 (1.94) | 14.03 (2.18) | 13.54 (2.43) | 13.07 (2.68) | 12.62 (2.92) | 12.19 (3.16) |
| Female immigrants ≤ 10(≥60y) | 14.72 (4.29) | 13.01 (4.83) | 12.52 (5.11) | 12.03 (5.37) | 11.55 (5.63) | 11.08 (5.88) | 10.63 (6.12) | 10.19 (6.35) | 9.76(6.56) | 9.34 (6.75) |
| Female locals(≥60y) | 12.20 (1.66) | 11.86 (1.84) | 11.67 (1.98) | 11.49 (2.15) | 11.33 (2.35) | 11.18 (2.58) | 11.04 (2.84) | 10.91 (3.11) | 10.79(3.4) | 10.68 (3.71) |
| Male immigrants >10(≥60y) | 37.23 (2.29) | 36.59 (2.56) | 35.17(3.18) | 33.82 (3.86) | 32.55 (4.57) | 31.34 (5.28) | 30.19 (6.01) | 29.08 (6.70) | 28.02 (7.40) | 27.01 (8.07) |
| Male immigrants ≤ 10(≥60y) | 42.30 (10.88) | 41.43 (11.78) | 41.03 (12.71) | 40.61 (13.70) | 40.17 (14.75) | 39.71 (15.85) | 39.24 (16.99) | 38.75 (18.16) | 38.23 (19.35) | 37.71 (20.57) |
| Male locals(≥60y) | 23.04 (3.29) | 22.69 (3.56) | 22.37(4.07) | 22.16(4.84) | 21.89 (5.86) | 21.61 (7.22) | 21.52 (9.02) | 21.74 (11.29) | 22.17 (14.03) | 22.73 (17.28) |

**eTable 5.** Predictive means and standard deviations (in brackets) of age-standardized mortality rates of stomach cancer per 100,000 population for each gender, age group (less than, greater or equal to 60 years old) and immigrant status from 2022 to 2030. Reported means and standard deviations (in brackets) of age-standardized mortality rates in 2021 are also listed.



For peer review only - http://bmjopen.bmj.com/site/about/guidelines.xhtml

| Predictive mean of age-standardized mortality rates of prostate cancer per 100,000 population | | | | | | | | | | |
|---|---|---|---|---|---|---|---|---|---|---|
| Year | 2021 | 2022 | 2023 | 2024 | 2025 | 2026 | 2027 | 2028 | 2029 | 2030 |
| Male immigrants >10 | 14.81 (0.61) | 14.59 (0.79) | 14.57 (0.96) | 14.56 (1.15) | 14.54 (1.37) | 14.51 (1.61) | 14.48 (1.86) | 14.45 (2.13) | 14.42 (2.42) | 14.38 (2.72) |
| Male immigrants ≤10 | 9.03 (2.95) | 8.78 (3.11) | 8.58 (3.29) | 8.39 (3.49) | 8.19(3.69) | 8.10(3.89) | 7.82(4.11) | 7.63(4.31) | 7.45(4.51) | 7.27(4.72) |
| Male locals | 9.54 (1.40) | 9.66 (1.57) | 9.67 (1.66) | 9.69 (1.77) | 9.72(1.91) | 9.75(2.06) | 9.78(2.23) | 9.82(2.43) | 9.86(2.64) | 9.9(2.88) |
| Male immigrants >10(<60y) | 0.57 (0.12) | 0.52 (0.17) | 0.51 (0.19) | 0.50 (0.21) | 0.49(0.22) | 0.48(0.24) | 0.47(0.25) | 0.46(0.27) | 0.45(0.29) | 0.44(0.31) |
| Male immigrants ≤ 10(<60y) | 0.65 (0.59) | 0.73 (0.77) | 0.81 (0.93) | 0.87 (1.10) | 0.94(1.31) | 1.01(1.51) | 1.09(1.75) | 1.16(2.02) | 1.24(2.32) | 1.33(2.64) |
| Male locals(<60y) | 0.63 (0.12) | 0.66 (0.14) | 0.66 (0.16) | 0.66 (0.19) | 0.67(0.21) | 0.67(0.24) | 0.67(0.27) | 0.68(0.31) | 0.68(0.33) | 0.69(0.37) |
| Male immigrants >10(≥ 60y) | 49.43 (2.59) | 49.61 (2.73) | 49.63 (3.29) | 49.64 (3.94) | 49.64 (4.68) | 49.64 (5.51) | 49.63 (6.38) | 49.62 (7.32) | 49.61 (8.32) | 49.58(9.3 7) |
| Male immigrants ≤ 10(≥60y) | 28.29 (9.15) | 27.66 (9.78) | 26.53 (10.21) | 25.4 (10.63) | 24.28 (11.03) | 23.16 (11.41) | 22.07 (11.76) | 21.01 (12.09) | 19.96 (12.38) | 18.95(12. 63) |
| Male locals(≥60y) | 31.57 (5.17) | 31.48 (5.49) | 31.40 (5.76) | 31.32 (6.09) | 31.24 (6.48) | 31.15 (6.94) | 31.06 (7.44) | 30.96 (8.01) | 30.86 (8.61) | 30.74(9.2 6) |

**eTable 6.** Predictive means and standard deviations (in brackets) of age-standardized mortality rates of prostate cancer per 100,000 population for each age group (less than, greater or equal to 60 years old) and immigrant status from 2022 to 2030. Reported means and standard deviations (in brackets) of age-standardized mortality rates in 2021 are also listed.

For peer review only



For peer review only - http://bmjopen.bmj.com/site/about/guidelines.xhtml

# Reporting checklist for cohort study.

Based on the STROBE cohort guidelines.

## Instructions to authors

Complete this checklist by entering the page numbers from your manuscript where readers will find each of the items listed below.

Your article may not currently address all the items on the checklist. Please modify your text to include the missing information. If you are certain that an item does not apply, please write "n/a" and provide a short explanation.

Upload your completed checklist as an extra file when you submit to a journal.

In your methods section, say that you used the STROBE cohortreporting guidelines, and cite them as:

von Elm E, Altman DG, Egger M, Pocock SJ, Gotzsche PC, Vandenbroucke JP. The Strengthening the Reporting of Observational Studies in Epidemiology (STROBE) Statement: guidelines for reporting observational studies.

| | | Reporting Item | Page Number |
|---|---|---|---|
| **Title and abstract** | | | |
| Title | #1a | Indicate the study's design with a commonly used term in the title or the abstract | 1 |
| Abstract | #1b | Provide in the abstract an informative and balanced summary of what was done and what was found | 2 |
| **Introduction** | | | |
| Background / rationale | #2 | Explain the scientific background and rationale for the investigation being reported | 4 |
| Objectives | #3 | State specific objectives, including any prespecified hypotheses | 4 |
| **Methods** | | | |
| Study design | #4 | Present key elements of study design early in the paper | 5 |
| Setting | #5 | Describe the setting, locations, and relevant dates, including periods | 5 |

For peer review only

| | | | |
|---|---|---|---|
| | | of recruitment, exposure, follow-up, and data collection | |
| Eligibility criteria | #6a | Give the eligibility criteria, and the sources and methods of selection of participants. Describe methods of follow-up. | 5 |
| Eligibility criteria | #6b | For matched studies, give matching criteria and number of exposed and unexposed | n/a |
| Variables | #7 | Clearly define all outcomes, exposures, predictors, potential confounders, and effect modifiers. Give diagnostic criteria, if applicable | 5 |
| Data sources / measurement | #8 | For each variable of interest give sources of data and details of methods of assessment (measurement). Describe comparability of assessment methods if there is more than one group. Give information separately for for exposed and unexposed groups if applicable. | 5 |
| Bias | #9 | Describe any efforts to address potential sources of bias | 5 |
| Study size | #10 | Explain how the study size was arrived at | 5 |
| Quantitative variables | #11 | Explain how quantitative variables were handled in the analyses. If applicable, describe which groupings were chosen, and why | 5 |
| Statistical methods | #12a | Describe all statistical methods, including those used to control for confounding | |
| 5 | | | |
| Statistical methods | #12b | Describe any methods used to examine subgroups and interactions | 5 |
| Statistical methods | #12c | Explain how missing data were addressed | 5 |
| Statistical methods | #12d | If applicable, explain how loss to follow-up was addressed | n/a |
| Statistical methods | #12e | Describe any sensitivity analyses | |
| n/a | | | |
| **Results** | | | |
| Participants | #13a | Report numbers of individuals at each stage of study—eg numbers potentially eligible, examined for eligibility, confirmed eligible, | n/a |

For peer review only

| | | | |
|---|---|---|---|
| | | included in the study, completing follow-up, and analysed. Give information separately for for exposed and unexposed groups if applicable. | |
| Participants | #13b | Give reasons for non-participation at each stage | 5 |
| Participants | #13c | Consider use of a flow diagram | |
| n/a | | | |
| Descriptive data | #14a | Give characteristics of study participants (eg demographic, clinical, social) and information on exposures and potential confounders. Give information separately for exposed and unexposed groups if applicable. | 5 |
| Descriptive data | #14b | Indicate number of participants with missing data for each variable of interest | |
| n/a | | | |
| Descriptive data | #14c | Summarise follow-up time (eg, average and total amount) | |
| n/a | | | |
| Outcome data | #15 | Report numbers of outcome events or summary measures over time. Give information separately for exposed and unexposed groups if applicable. | |
| n/a | | | |
| Main results | #16a | Give unadjusted estimates and, if applicable, confounder-adjusted estimates and their precision (eg, 95% confidence interval). Make clear which confounders were adjusted for and why they were included | 6 |
| Main results | #16b | Report category boundaries when continuous variables were categorized | n/a |
| Main results | #16c | If relevant, consider translating estimates of relative risk into absolute risk for a meaningful time period | |
| n/a | | | |
| Other analyses | #17 | Report other analyses done—eg analyses of subgroups and interactions, and sensitivity analyses | 7 |

**Discussion**

For peer review only

| | | | |
|---|---|---|---|
| Key results | #18 | Summarise key results with reference to study objectives | 8 |
| Limitations | #19 | Discuss limitations of the study, taking into account sources of potential bias or imprecision. Discuss both direction and magnitude of any potential bias. | 10 |
| Interpretation | #20 | Give a cautious overall interpretation considering objectives, limitations, multiplicity of analyses, results from similar studies, and other relevant evidence. | 8 |
| Generalisability | #21 | Discuss the generalisability (external validity) of the study results | 9 |
| **Other Information** | | | |
| Funding | #22 | Give the source of funding and the role of the funders for the present study and, if applicable, for the original study on which the present article is based | 11 |

The STROBE checklist is distributed under the terms of the Creative Commons Attribution License CC-BY. This checklist was completed on 11. February 2023 using https://www.goodreports.org/, a tool made by the EQUATOR Network in collaboration with Penelope.ai

For peer review only

BMJ Open

# An age-period-cohort analysis and projection of cancer mortality in Hong Kong, 1998–2030

| | |
|---|---|
| Journal: | *BMJ Open* |
| Manuscript ID | bmjopen-2023-072751.R3 |
| Article Type: | Original research |
| Date Submitted by the Author: | 30-Aug-2023 |
| Complete List of Authors: | Zhao, Yanji; The Hong Kong Polytechnic University, Department of Applied Mathematics<br>Zhuang, Zian; The Hong Kong Polytechnic University, Department of Applied Mathematics; University of California Los Angeles, Department of Biostatistics<br>Yang, Lin; The Hong Kong Polytechnic University, School of Nursing<br>He, Daihai; The Hong Kong Polytechnic University, Department of Applied Mathematics; The Hong Kong Polytechnic University, Research Institute for Future Food |
| <b>Primary Subject Heading</b>: | Research methods |
| Secondary Subject Heading: | Public health, Oncology |
| Keywords: | STATISTICS & RESEARCH METHODS, ONCOLOGY, Risk Factors |
| | |

SCHOLARONE™
Manuscripts

BMJ

*I, the Submitting Author has the right to grant and does grant on behalf of all authors of the Work (as defined in the below author licence), an exclusive licence and/or a non-exclusive licence for contributions from authors who are: i) UK Crown employees; ii) where BMJ has agreed a CC-BY licence shall apply, and/or iii) in accordance with the terms applicable for US Federal Government officers or employees acting as part of their official duties; on a worldwide, perpetual, irrevocable, royalty-free basis to BMJ Publishing Group Ltd ("BMJ") its licensees and where the relevant Journal is co-owned by BMJ to the co-owners of the Journal, to publish the Work in this journal and any other BMJ products and to exploit all rights, as set out in our licence.*

*The Submitting Author accepts and understands that any supply made under these terms is made by BMJ to the Submitting Author unless you are acting as an employee on behalf of your employer or a postgraduate student of an affiliated institution which is paying any applicable article publishing charge ("APC") for Open Access articles. Where the Submitting Author wishes to make the Work available on an Open Access basis (and intends to pay the relevant APC), the terms of reuse of such Open Access shall be governed by a Creative Commons licence – details of these licences and which Creative Commons licence will apply to this Work are set out in our licence referred to above.*

*Other than as permitted in any relevant BMJ Author's Self Archiving Policies, I confirm this Work has not been accepted for publication elsewhere, is not being considered for publication elsewhere and does not duplicate material already published. I confirm all authors consent to publication of this Work and authorise the granting of this licence.*

For peer review only

# An age-period-cohort analysis and projection of cancer mortality in Hong Kong, 1998–2030

Yanji Zhao[1,#], Zian Zhuang[1,2,#], Lin Yang[3] and Daihai He [1,4*]

1 Department of Applied Mathematics, Hong Kong Polytechnic University, Hong Kong, China

2 Department of Biostatistics, University of California, Los Angeles, CA, United States

3 School of Nursing, Hong Kong Polytechnic University, Hong Kong, China

4 Research Institute for Future Food, Hong Kong Polytechnic University, Hong Kong, China

# These authors contributed equally to this work.

* To whom correspondence should be addressed:

Department of Applied Mathematics, The Hong Kong Polytechnic University, Hong Kong, China

Tel: 852-27667864; daihai.he@polyu.edu.hk (DH).

**Email of all authors**

Yanji Zhao: yan-ji.zhao@connect.polyu.hk; Zian Zhuang: zianzhuang@ucla.edu;

Lin Yang: l.yang@polyu.edu.hk; Daihai He: daihai.he@polyu.edu.hk.

*Word count:3379*



For peer review only - http://bmjopen.bmj.com/site/about/guidelines.xhtml

# Abstract

**Objectives:** To explore the relationship between immigration groups and cancer mortality, this study aimed to explore age, period, birth cohort effects and effects across genders and immigration groups on mortality rates of lung, pancreatic, colon, liver, prostate and stomach cancers and their projections.

**Design, Setting, and Participants:** Death registry data in Hong Kong between 1998 and 2021, which were stratified by age, sex and immigration status. Immigration status was classified into three groups: locals born in Hong Kong, long-stay immigrants and short-stay immigrants.

**Methods:** Age-period-cohort analysis was used to examine age, period, and birth cohort effects for genders and immigration groups from 1998 to 2021. Bayesian age-period-cohort models were applied to predict the mortality rates from 2022 to 2030.

**Results:** Short-stay immigrants revealed pronounced fluctuations of mortality rates by age and of relative risks by cohort and period effects for six types of cancers than those of long-stay immigrants and locals. Immigrants for each type of cancer and gender will be at a higher mortality risk than locals, as men will be at a higher risk of mortality from cancers than women in the future (excluding prostate cancer). After 2021, decreasing trends ($p<0.05$) or plateau ($p>0.05$) of forecasting mortality rates of cancers occur for all immigration groups, except for increasing trends for short-stay male immigrants with colon cancer ($p<0.05$, Avg +0.30 deaths/100,000 per annum from 15.47 to 18.50 deaths/100,000) and long-stay male immigrants with pancreatic cancer ($p<0.05$, Avg +0.72 deaths/100,000 per annum from 16.30 to 23.49 deaths/100,000).,

**Conclusions:** Findings underscore the effect of gender and immigration status in Hong Kong on mortality risks of cancers that immigrants for each type of cancer and gender will be at a higher mortality risk than locals.


***Keywords***: Age-period-cohort analysis, immigration, mortality, lung cancer, pancreatic cancer, colon cancer, liver cancer, prostate cancer, stomach cancer



For peer review only - http://bmjopen.bmj.com/site/about/guidelines.xhtml

## Strengths and limitations of this study

- This study provides new evidence regarding the relationship between immigration status and cancer mortality, given the effects of age, period, birth cohort and their predictions.

- The non-identifiability problem has not been interpreted in APC models

- The future perspective of cancer therapies and techniques have not been considered.

For peer review only



For peer review only - http://bmjopen.bmj.com/site/about/guidelines.xhtml

# Introduction

Several migration waves from mainland China to Hong Kong have occurred over the past century. These migration waves included a large-scale migration inflow from 1945 to 1950 (the Chinese Civil War) and a few small-scale inflows in the 1950s, 1970s, and 1990s [1-3]. In 2016, immigrants from mainland China formed approximately 38% of the population of Hong Kong. These inflows have led to a growing interest in research on the disparity of health conditions between the locals and immigrants.

Cancer has been one of the most common causes of death, as an estimated 19.3 million new cancer cases and 9.9 million new cancer-associated deaths occurred worldwide in 2020 [4]. In Hong Kong, lung cancer is one of the most common causes of cancer deaths [5, 6]. Previous studies suggested that the primary cause of lung cancer is cigarette smoking [7-11]. Genetic factors, asbestos, radon gas, second-hand smoke, and other forms of air pollution have been proven to influence the risk of lung cancer [12-18]. The overall daily smoking rate in mainland China was approximately 23.2% in 2018 [19], whereas the daily smoking rate in Hong Kong was only 10.2% in 2019 [20]. The leading causes of liver cancer include viral infection, drinking of alcohol and polluted water and food supplies which are also culprits for colon, stomach and pancreatic cancer [21]. Alcohol consumption per capita in Hong Kong has reached 2.37 liters in 2021 [22], compared to 7.0 liters of per capita consumption of alcohol in mainland China in 2018 [23]. As approximately 99% of prostate cancer cases occur after age 50, factors of prostate cancer have been regarded as old age, race, family history and the diet of red meat consumption [24]. In addition to these risk factors, studies have suggested that cancer mortality rates vary depending on migrant status [25-28]. According to data from the Census and Statistics Department of Hong Kong, approximately 81% of immigrants in Hong Kong immigrated from mainland China, Macau, and Taiwan. Immigrants from mainland China account for the bulk of this population. Previous studies have shown that child immigrants in Hong Kong tend to suffer from a higher risk of wheezing disorders and cardiovascular diseases, and immigrant women have higher age-specific mortality rates of breast cancer than locally-born women in Hong Kong [29, 30]. However, to date, few studies have investigated the effect of length of stay in Hong Kong and birthplace on the risk of other types of cancer.

In this study, we compared the mortality rates of lung, pancreatic, colon, liver, prostate and stomach cancers between locally born residents in Hong Kong and immigrants from mainland China. Both populations are widely considered as ethnically homogeneous with similar cultures. Nevertheless, due to different early life experiences, immigrants are exposed to more various social economy and lifestyles than locals. Therefore, it's constructive to ascertain whether immigrants from mainland China have a different mortality pattern of cancers from locals to



For peer review only - http://bmjopen.bmj.com/site/about/guidelines.xhtml

verify the significance of migration status for this health outcome. As Age-period-cohort (APC) analysis plays a vital role in studying time-specific phenomena in epidemiology, in this study, to evaluate the effect of immigration on cancer mortality in the past and future, we developed APC models specified by sex and migrant status to assess the effects of age, period, birth cohort, and of the length of stay in Hong Kong on the mortality risks of cancers. Additionally, we explore the projection of mortality rates for the locally born population and immigrants in Hong Kong who were younger or older than 60 using a predictive model, taking into account age, period, and birth cohort effects as well.

## Methods

### *Data*

We obtained the death registry data in Hong Kong between 1998 and 2021 from the Census and Statistics Department of Hong Kong, as the data in 2022 has not been available up to now. The data was extracted from a routine census held by the Hong Kong government as subjective errors caused by resampling can be neglected. The population data were stratified by age, sex, immigration status, and length of stay in Hong Kong. We retrieved six types of cancer cases from the death registry data using ICD codes, such as ICD-9 code 162 and ICD-10 codes C34.0–C34.3, C348, and C349 for lung cancer. To assure comparability among registries, deaths from the age group of 35–85 years were selected, since cases younger than 35 and older than 85 were relatively trivial for lack of statistical interpretability [31]. Immigration status was classified into three groups: locals born in Hong Kong, immigrants who have lived in Hong Kong for >10 years before death defined as long-stay immigrants, and immigrants who have lived in Hong Kong for ≤10 years before death defined as short-stay immigrants. Notably, much focus was placed on immigrants from mainland China, because approximately 81% of immigrants in Hong Kong came from mainland China, Macau, and Taiwan based on the data from the Census and Statistics Department of Hong Kong. Moreover, few cases recorded from Macau and Taiwan are statistically insignificant in the analysis. Demographics and population projections from 2022 to 2030 were retrieved from the Census and Statistics Department of Hong Kong and estimated with cubic smoothing spline as the prerequisite of the predictive model. Codes for APC and BAPC analysis are available in the GitHub repository (https://github.com/kshz2164313/APC-population-projections-for-immigration-HK).

### *Statistical analysis*

We modeled cancer mortality rates in Hong Kong using APC analysis based on log-linear Poisson regression models. The model aimed to disentangle age, period, and cohort effects of time-varying phenomena simultaneously [32, 33], given that



For peer review only - http://bmjopen.bmj.com/site/about/guidelines.xhtml

$$\log(E_{ij}) = \alpha_i + \beta_j + \gamma_k + \mu + \log(\theta_{ij}) \quad (1)$$

where $E_{ij}$ denotes expected mortality; $\alpha_i$, $\beta_j$, and $\gamma_k$ denote age, period, and cohort effect, respectively, for $i=1,\ldots,I$, $j=1,\ldots,J$, $k=1,\ldots,K$ with $k=I-i+j$. $\log(\theta_{ij})$ is the offset. We mainly focused on the contributions of sex and immigration status due to the non-identifiability problem that the effects of these three components are collinear with each other (denoted as period – age = cohort) [34]. Birth cohort effect and period effect were assessed with relative risks to evaluate the effect of three components. The median year of birth among cases was regarded as the reference cohort [35,36]. Since death cases aged at 35–85 years between 1998 and 2021 were selected, the range of birth cohort from 1913 to 1986 covered observations and further projections until 2030. The second and penultimate period effects were constrained to the reference for period. For sex and immigration status, maximum likelihood framework was applied to estimate the relative risks and 95% confidence intervals (CIs) by age groups, calendar period, and birth cohort.

Several projection approaches for future cancer mortality have been developed, but a Bayesian age-period-cohort (BAPC) model built upon integrated nested Laplace approximations (INLA) [37] yields relatively higher coverage and better performance for all evaluated parameter combinations [38]. To prevent some sampling problems caused by Markov chain Monte Carlo (MCMC), this MCMC-free BAPC approach was applied to predict future cancer mortality within a fully Bayesian inference setting and provide outputs of interest simply, such as projected age-standardized and age-specific rates. Convergence checks are not necessary for this technique [37]. The projections of age-standardized cancer mortality rates for each sex, age group (younger or older than 60 years) and migrant status, taking into account age, period, and birth cohort effects, were performed based on the weights of population age groups from the WHO World Standard population [39], with 95% prediction intervals. Mann-Kendall trend test was applied to verify the projection trend. Friedman's Two-Way Analysis of Variance was applied to test interactions between gender and immigration groups for each year.

All analyses were performed via R version 4.2.1 (R Core Team, R Foundation for Statistical Computing, Vienna, Austria, 2013, http://www.R-project.org/). The APC models were established using the Epi package, and the projections based on Bayesian APC models were performed with the BAPC package.

*Patient and Public Involvement*

None.

## Results

Figure 1 & 2 and eFigure 1(a-e) in **Supplementary Material** illustrate the estimates of age



For peer review only - http://bmjopen.bmj.com/site/about/guidelines.xhtml

(assessed by cancer mortality), cohort and period effects (assessed by relative risk) based on APC models among three migrant groups for men and women with six types of cancers, respectively. All the mortality rates for each gender and immigration status exhibit notable increasing trends with age. Age, cohort and period effects of six types of cancer for immigrants who stayed in Hong Kong for ≤10 years revealed relatively more pronounced fluctuations and deviations from those effects in the other two immigration groups. Significant increasing trends of age effect occurred in all types of cancer, regardless of gender and immigration status. For example, while relatively insignificant differences in lung cancer mortality rates by immigration status among females have performed, male immigrants who remained in Hong Kong for >10 years had higher lung cancer mortality rates at ages above 50 years and those who arrived ≤10 years had lower lung cancer mortality at ages below 62 years compared to local men Figure 1. In addition to compatible dynamics of period effect for locals and long-stay immigrants, similar changes of relative risks by birth cohort for locals and long-stay immigrants in lung, colon, liver and stomach cancers occurred before 1945, whereas significant differences of relative risks by birth cohort between these two immigration groups occurred after 1960 (Figure 1 & eFigure 1(a,b,d)). Locals and long-stay immigrants in pancreatic and prostate cancer perform almost similar changes of relative risks by birth cohort effects all the time (eFigure 1(c,e)). Short-stay immigrants who have stayed in Hong Kong for ≤10 years had more fluctuating relative risks affected by period effects before 2020 than those for locals and long-stay immigrants. Lack of young cases, especially young short-stay immigrants, of prostate cancer leads to significant deviations and variances in age and cohort effects.

Figure 3-5, eFigure 2-6 in **Supplementary Material** illustrate the age-standardized mortality rates of six types of cancer from 1998 to 2021 and their projections by sex, immigrant status and age groups from 2022 to 2030, taking into account age, period, and birth cohort effects. Means and standard deviations of predictive mortality rates are shown in eTable 1-6 in **Supplementary Material**. For all ages projection (Figure 2 & eFigure 2-6), as men will be at higher risk of mortality rates of cancers (excluding prostate cancer) than women in the future for all three age groups (all ages, young and older than 60 years) and approximately significant interactions between gender and immigration groups emerge for each type of cancer in each year ($p<0.05$), given the projected trends, immigrants for each gender, especially who have stayed in Hong Kong for > 10 years will suffer from higher mortality rates of cancer in each year than locals. Monotone decreasing trends or plateau of forecasting occur for both genders and all immigration groups in cancers, except for increasing trends for male immigrants who have stayed in Hong Kong for ≤10 years with colon cancer ($p < 0.05$, Avg +0.30 deaths/100,000 per annum) from 15.47 deaths/100,000 (95% CI: 11.28, 19.66) in 2021 to 18.50 deaths/100,000 (95% CI: 2.31, 34.69) in 2030, and male immigrants who have stayed in Hong Kong for > 10 years with pancreatic cancer ($p < 0.05$, Avg +0.72 deaths/100,000 per annum) from 16.30



For peer review only - http://bmjopen.bmj.com/site/about/guidelines.xhtml

deaths/100,000 (95% CI: 14.38,17.26) in 2021 to 23.49 deaths/100,000 (95% CI: 12.49, 34.49) in 2030. Most of predictive trends for younger cases (<60 years) and older cases (≥60 years) reach a consensus with those for all ages population, except for two phenomena: 1.) mortality rates of lung cancer for men immigrants ≤10 that insignificant trend for all ages ($p > 0.05$) vs. decline for younger cases ($p < 0.05$) vs. increase for older cases ($p < 0.05$); 2.) mortality rates of liver cancer for men immigrants >10 that decline for all ages ($p < 0.05$) vs. decline for younger cases ($p < 0.05$) vs. insignificant trend for older cases ($p > 0.05$). Some particular cases occur in the projection of prostate cancer that young long-stay male immigrants (0.44 deaths/100,000, 95% CI: 0, 1.05) aged less than 60 will be at lower mortality rate than locals (0.69 deaths/100,000, 95% CI: 0, 1.42) in 2030 (eTable 6). Compared with other cancers and immigration groups, male immigrants who have stayed in Hong Kong for >10 years with lung cancer would perform the most significant decline in predictive mean from 102.90 (95% CI: 98.14, 107.66) to 79.55 (95% CI: 47.46, 111.64) deaths per 100,000 population (Avg -2.34 deaths/100,000 per annum) (eTable 1), while the same immigration group with pancreatic cancer would indicate the most significant uptrend in each year of 16.30 (95% CI: 14.38,17.26) and 23.49 (95% CI: 12.49, 34.49) deaths per 100,000 population in 2021 and 2030, respectively (Avg +0.72 deaths/100,000 per annum) (eTable 4).

## Discussion

Early detection of cancer is positive and instructive for increasing chances of cure. Nevertheless, the high mortality rate of cancer results from late diagnosis among most patients after progression to more advanced or severe stages. Individuals at high risk of cancer, such as smokers, alcoholics or those who are frequently exposed to susceptible circumstances, should be screened for early detections to increase opportunities for cure [40]. Therefore, the differences in mortality rates among immigration groups are synonymous with detection means, therapies, and social history in disparate periods and areas.

While the changes in mortality rates by age for long-stay immigrants reached approximate harmony with those for locals, the changes in mortality rates by age for short-stay immigrants revealed clear differences with those for the other two populations. The group of long-stay immigrants had a higher risk of death from lung, colon and liver cancers than the other two immigration groups after the age of 60 years. Short-stay male immigrants were less likely to die from lung cancer before the age of 65 years. The contrast in age effects among the immigration groups was partially consistent with studies [25, 41] that highlighted the age effects for locals and immigrants on breast cancer mortality in Hong Kong and lung cancer



For peer review only - http://bmjopen.bmj.com/site/about/guidelines.xhtml

incidence in Sweden, as they both showed similar trends and magnitudes between locals and immigrants before the age of 60 years. They are also compatible with the results in [42] that diagnosis of liver cancer is the most frequent among populations at 55 to 65 years old. According to these trends, young individuals, especially new young immigrant men, who have benefited from all-rounded development in mainland China and Hong Kong, are more likely to seek early detection and be treated for cancers using more advanced treatments [43]. Differences in birth cohort effects among immigrant groups partially comply with the interpretation above.

We observed significant trends of cohort effects among locals and immigrants. These findings are partially consistent but subtly different from previous findings, regarding the effect of immigration status on cancers. Zhao et al. [25] described multiple peaks of cohort effects on breast cancer mortality between locals and immigrants in Hong Kong, as well as a significant decline in cohort effects after 1950. In contrast, Sung et al. [44] investigated the difference in breast cancer incidence between Chinese Americans and non-Hispanic whites in the U.S. and emphasized that Chinese Americans were at lower risk of breast cancer than non-Hispanic whites born in the same year. Here, we interpret the cohort-driven trends resulting from the intricacy of social history and lifestyle. Compared to a relatively stable social development in Hong Kong, representing downward trends of relative risks for locals, wars and social instability in mainland China resulted in several immigration waves from mainland China to Hong Kong before 1950. Additionally, remarkable increasing trends were recorded for new immigrants after 1950, which corresponded to the economic downturn after wars and famine between 1959 and 1961 during their youth [45]. The increasing trends for new immigrants and similar trends for locals and long-stay immigrants were consistent with the finding that nutrient deficiency contributes to a higher risk of severe mortality rates of cancers [46]. Furthermore, we speculate that these trends, especially those for locals and long-stay immigrants, are most likely attributed to social development and personal behaviors, such as daily habits, occupational history, different diagnoses and treatments, and domestic environmental exposures. Notably, short-stay immigrants suffered from a lower risk of death from colon cancer for all ages (eFigure 1a in **Supplementary Material**). As locals and immigrants in Hong Kong transitioned to more westernized lifestyles, higher consumption of meat was associated with a higher risk of these types of cancer, whereas consumption of vegetables had a strong protective effect against pancreatic cancer, and moderate consumption of coffee appeared to be beneficial against lung cancer [47,48]. Further studies on potential risk factors are required.

Short-stay immigrants had more fluctuating and non-stationary but inconspicuous relative risks by period effects before 2021 than locals and long-stay immigrants. Cumulatively, an



For peer review only - http://bmjopen.bmj.com/site/about/guidelines.xhtml

arch pattern and fluctuating curve depicting period effects externally resulted in an arch pattern of age-standardized mortality rates for short-stay immigrant women and irregular rates for short-stay immigrant men before 2021. The external performance of different period effects on mortality rates could be most likely attributed to the higher effect of different lifestyles and social development on new immigrants than on long-stay immigrants and locals in Hong Kong. For the age-standardized mortality rates and projections, consistent with previous findings [49,50], we predict that the mortality rates of cancer in Hong Kong after 2021 will continue to decline or remain relatively stable, consistent with the trends before 2020, except for male immigrants who have stayed in Hong Kong for ≤10 years with colon cancer and male immigrants who have stayed in Hong Kong for >10 years with pancreatic cancer. Men will be at higher risk of mortality rates of cancer than women, regardless of immigration status. They are also compatible with the results in [4] that men suffer from a higher risk of these types of cancer than women, excluding prostate cancer. Furthermore, new immigrant women will be at lower risk than local women, even though long-stay immigrants will suffer from higher mortality rates than locals in the future. Potential interpretations could be consistent with those for birth cohort effects, as age and period effects are considered as confounders of cohort effect.

In the past few decades, spurred by an increasing burden of high incidence and mortality rates of cancer, several studies focused on the inherent identification dilemma of three effects in the APC model. Further, complicated population distribution and immigration status in Hong Kong, one of the areas with the highest population density and migration frequency in the world, have intricate causes and inherent dynamics of cancer and other diseases. To our knowledge, few studies have assessed the relationship between immigration status and cancer mortality. Therefore, this study is original to examine the effect of the length of stay in Hong Kong and origins of previous residence on cancer deaths, which is instructive for further immigration policy-making and targeted strategies of disease detection and intervention. However, this study had several limitations. Given the non-identifiability problem in age-period-cohort models, we could only depict trends and variations among different immigration and sex groups, as illustrated in figures, and insufficiently perform the estimates of the contributions of three effects or subgroups to mortality rates. Furthermore, we adopted a cubic smoothing spline to estimate populations of immigrants and locals due to the large proportion of unspecified immigration status from official demographic projections. A few acceptable cases resulted in a limited type of cancer so that some common cancers, such as the ovary and cervix, were discarded. Since the issue of quantification, the future perspective of cancer therapies and techniques have not been considered in the model of projection.



For peer review only - http://bmjopen.bmj.com/site/about/guidelines.xhtml

## Conclusion

We conclude that immigrants, especially short-stay immigrants, had more pronounced fluctuations of mortality rates by age and of relative risks by cohort and period effects for six types of cancers than those of long-stay immigrants and locals. Men will be at a higher risk of mortality rates of six types of cancer than women in the future. Male immigrants who have stayed in Hong Kong for ≤10 years with colon cancer and male immigrants who have stayed in Hong Kong for >10 years with pancreatic cancer would perform significant uptrend in the future, while other immigration groups for each type of cancer would continue to decline or remain relatively stable. Immigrants for each gender in Hong Kong would suffer from higher mortality risks of cancers than locals in the future.

For peer review only



For peer review only - http://bmjopen.bmj.com/site/about/guidelines.xhtml

## Declaration

### Ethical approval and consent to participate

Ethical approval and consent to participate are not applicable. This study does not involve human participants. Data was obtained from the Census and Statistics Department of Hong Kong.

### Consent for publication

Not applicable.

### Data Availability Statement

Data are available upon reasonable request.

### Author contributions

**Yanji Zhao:** Methodology, Formal analysis, Data Curation, Writing - Original Draft, Visualization
**Zian Zhuang:** Methodology, Formal analysis, Data Curation, Writing - Review & Editing
**Lin Yang:** Validation, Writing - Review & Editing
**Daihai He:** Conceptualization, Writing - Review & Editing, Supervision

### Funding

The work described in this paper was partially supported by a grant from the Research Grants Council of the Hong Kong Special Administrative Region, China (HKU C7123-20G).

### Conflict of interest

None declared.

### Acknowledgements

None.

For peer review only



For peer review only - http://bmjopen.bmj.com/site/about/guidelines.xhtml

## References


1.Fan S-C. The population projection of Hong Kong. Southeast Asian Journal of Social Science. 1974;2(1/2):105-17.

2.Department CaS. Hong Kong Statistics 1947-1967 (Report). https://www.statistics.gov.hk/pub/hist/1961_1970/B10100031967AN67E0100.pdf, Accessed 4th May 2019.

3. Department CaS. Demographic Trends in Hong Kong 1981-2011 (Report). http://www.statistics.gov.hk/pub/B1120017032012XXXXB0100.pdf, Accessed 4th May 2019.

4. Sung H, Ferlay J, Siegel RL, Laversanne M, Soerjomataram I, Jemal A, et al. Global Cancer Statistics 2020: GLOBOCAN Estimates of Incidence and Mortality Worldwide for 36 Cancers in 185 Countries. CA: A Cancer Journal for Clinicians. 2021 2021/05/01;71(3):209-49. doi: https://doi.org/10.3322/caac.21660.

5. Wang XR, Chiu YL, Qiu H, Au JSK, Yu ITS. The roles of smoking and cooking emissions in lung cancer risk among Chinese women in Hong Kong. Annals of Oncology. 2009 2009/04/01/;20(4):746-51. doi: https://doi.org/10.1093/annonc/mdn699.

6. Chiu Y-L, Wang X-R, Qiu H, Yu IT-S. Risk factors for lung cancer: a case–control study in Hong Kong women. Cancer Causes & Control. 2010 2010/05/01;21(5):777-85. doi: 10.1007/s10552-010-9506-9.

7. Office on S, Health. Publications and Reports of the Surgeon General. Women and Smoking: A Report of the Surgeon General. Atlanta (GA): Centers for Disease Control and Prevention (US); 2001.

8. Escobedo LG, Peddicord JP. Smoking prevalence in US birth cohorts: the influence of gender and education. American Journal of Public Health. 1996 1996/02/01;86(2):231-6. doi: 10.2105/AJPH.86.2.231.

9. Husten CG, Shelton DM, Chrismon JH, Lin YC, Mowery P, Powell FA. Cigarette smoking and smoking cessation among older adults: United States, 1965-94. Tobacco Control. 1997;6(3):175. doi: 10.1136/tc.6.3.175.

10. Bolego C, Poli A, Paoletti R. Smoking and gender. Cardiovascular Research. 2002;53(3):568-76. doi: 10.1016/S0008-6363(01)00520-X.

11. Doll R, Hill AB. The mortality of doctors in relation to their smoking habits; a preliminary report. Br Med J. 1954;1(4877):1451-5. PMID: 13160495. doi: 10.1136/bmj.1.4877.1451.

12. Ramada Rodilla JM, Calvo Cerrada B, Serra Pujadas C, Delclos GL, Benavides FG. Fiber burden and asbestos-related diseases: an umbrella review. Gaceta Sanitaria. 2021 2021/06/11/. doi: https://doi.org/10.1016/j.gaceta.2021.04.001.

13. Collishaw NE, Kirkbride J, Wigle DT. Tobacco smoke in the workplace: an occupational health hazard. Can Med Assoc J. 1984;131(10):1199-204. PMID: 6498670.

14. Dresler CM, Fratelli C, Babb J, Everley L, Evans AA, Clapper ML. Gender differences in genetic susceptibility for lung cancer. Lung Cancer. 2000 2000/12/01/;30(3):153-60. doi: https://doi.org/10.1016/S0169-5002(00)00163-X.

15. Alexandrov K, Cascorbi I, Rojas M, Bouvier G, Kriek E, Bartsch H. CYP1A1 and GSTM1 genotypes affect benzo[ a ]pyrene DNA adducts in smokers' lung: comparison with aromatic/hydrophobic adduct formation. Carcinogenesis. 2002;23(12):1969-77. doi:




For peer review only - http://bmjopen.bmj.com/site/about/guidelines.xhtml

10.1093/carcin/23.12.1969.
16. Samet JM. Radon and Lung Cancer. JNCI: Journal of the National Cancer Institute. 1989;81(10):745-58. doi: 10.1093/jnci/81.10.745.
17. Darby S, Hill D, Auvinen A, Barros-Dios JM, Baysson H, Bochicchio F, et al. Radon in homes and risk of lung cancer: collaborative analysis of individual data from 13 European case-control studies. BMJ. 2005;330(7485):223. doi: 10.1136/bmj.38308.477650.63.
18. Raaschou-Nielsen O, Andersen ZJ, Beelen R, Samoli E, Stafoggia M, Weinmayr G, et al. Air pollution and lung cancer incidence in 17 European cohorts: prospective analyses from the European Study of Cohorts for Air Pollution Effects (ESCAPE). The Lancet Oncology. 2013 2013/08/01/;14(9):813-22. doi: https://doi.org/10.1016/S1470-2045(13)70279-1.
19. *2018 Summary-20190719*. Retrieved August 26, 2022, from https://www.who.int/docs/default-source/wpro---documents/countries/china/2018-gats-china-factsheet-cn-en.pdf?sfvrsn=3f4e2da9_2
20. *Thematic household survey*.Retrieved August 26, 2022, from https://www.censtatd.gov.hk/en/data/stat_report/product/B1130201/att/B11302702020XXXXB0100.pdf
21. Abubakar II, Tillmann T, Banerjee A. Global, regional, and national age-sex specific all-cause and cause-specific mortality for 240 causes of death, 1990-2013: a systematic analysis for the Global Burden of Disease Study 2013. Lancet. 2015 Jan 10;385(9963):117-71.
22. Estimated alcohol consumption per capita in Hong Kong. Change4Health. (n.d.). Retrieved December 1, 2022, from https://www.change4health.gov.hk/en/alcohol_aware/figures/alcohol_consumption/index.html
23. World Health Organization. Global status report on alcohol and health 2018. World Health Organization; 2019 Feb 14.
24. Wild C. World cancer report 2014. Wild CP, Stewart BW, editors. Geneva, Switzerland: World Health Organization; 2014.

25. Zhao S, Dong H, Qin J, Liu H, Li Y, Chen Y, et al. Breast cancer mortality in Chinese women: does migrant status play a role? Annals of Epidemiology. 2019 2019/12/01/;40:28-34.e2. doi: https://doi.org/10.1016/j.annepidem.2019.10.006.
26. Gomez SL, Yang J, Lin S-W, McCusker M, Sandler A, Cheng I, et al. Incidence trends of lung cancer by immigration status among Chinese Americans. Cancer Epidemiol Biomarkers Prev. 2015;24(8):1157-64. PMID: 25990553. doi: 10.1158/1055-9965.EPI-15-0123.
27.Hemminki K, Li X, Czene K. Cancer risks in first-generation immigrants to Sweden. International Journal of Cancer. 2002 2002/05/10;99(2):218-28. doi: https://doi.org/10.1002/ijc.10322.
28. Vanthomme K, Rosskamp M, De Schutter H, Vandenheede H. Lung cancer incidence differences in migrant men in Belgium, 2004–2013: histology-specific analyses. BMC Cancer. 2021 2021/03/30;21(1):328. doi: 10.1186/s12885-021-08038-6.
29. Schooling M, Leung GM, Janus ED, Ho SY, Hedley AJ, Lam TH. Childhood migration and cardiovascular risk. International Journal of Epidemiology. 2004;33(6):1219-26. doi: 10.1093/ije/dyh221.
30. Leung JYY, Li AM, Leung GM, Schooling CM. Mode of delivery and childhood



For peer review only - http://bmjopen.bmj.com/site/about/guidelines.xhtml

hospitalizations for asthma and other wheezing disorders. Clinical & Experimental Allergy. 2015 2015/06/01;45(6):1109-17. doi: https://doi.org/10.1111/cea.12548.

31. Baker A, Bray I. Bayesian projections: what are the effects of excluding data from younger age groups?. American Journal of Epidemiology. 2005 Oct 15;162(8):798-805.

32. Rosenberg PS, Anderson WF. Age-Period-Cohort Models in Cancer Surveillance Research: Ready for Prime Time? APC Models. Cancer Epidemiology, Biomarkers & Prevention. 2011 Jul 1;20(7):1263-8.

33. Holford T. Analyzing the effects of age, period and cohort on incidence and mortality rates. Stat Meth Med Res. 1992;1:317-37.

34. Brookmeyer R, Stroup DF, editors. Monitoring the health of populations: statistical principles and methods for public health surveillance. Oxford University Press; 2004.

35. Yang Y, Land KC. Age-period-cohort analysis: New models, methods, and empirical applications. Taylor & Francis; 2013.

36. Robertson C, Gandini S, Boyle P. Age-period-cohort models: a comparative study of available methodologies. Journal of clinical epidemiology. 1999 Jun 1;52(6):569-83.

37. Riebler A, Held L. Projecting the future burden of cancer: Bayesian age–period–cohort analysis with integrated nested Laplace approximations. Biometrical Journal. 2017 May;59(3):531-49.

38. Knoll M, Furkel J, Debus J, Abdollahi A, Karch A, Stock C. An R package for an integrated evaluation of statistical approaches to cancer incidence projection. BMC medical research methodology. 2020 Dec;20(1):1-1.

39. Ahmad OB, Boschi-Pinto C, Lopez AD, Murray CJ, Lozano R, Inoue M. Age standardization of rates: a new WHO standard. Geneva: World Health Organization. 2001 Jan;9(10):1-4.

40. Sung H, Ferlay J, Siegel RL, Laversanne M, Soerjomataram I, Jemal A, Bray F. Global cancer statistics 2020: GLOBOCAN estimates of incidence and mortality worldwide for 36 cancers in 185 countries. CA: a cancer journal for clinicians. 2021 May;71(3):209-49.

41. Mousavi SM, Fallah M, Sundquist K, Hemminki K. Age-and time-dependent changes in cancer incidence among immigrants to Sweden: colorectal, lung, breast and prostate cancers. International journal of cancer. 2012 Jul 15;131(2):E122-8.

42. National Cancer Institute. SEER stat fact sheets: liver and intrahepatic bile duct cancer.

43. Wu X, Chung VC, Hui EP, Ziea ET, Ng BF, Ho RS, Tsoi KK, Wong S, Wu JC. Effectiveness of acupuncture and related therapies for palliative care of cancer: overview of systematic reviews. Scientific reports. 2015 Nov 26;5(1):1-5.

44. Sung H, Rosenberg PS, Chen WQ, Hartman M, Lim WY, Chia KS, Wai-Kong Mang O, Tse L, Anderson WF, Yang XR. The impact of breast cancer-specific birth cohort effects among younger and older C hinese populations. International journal of cancer. 2016 Aug 1;139(3):527-34.

45. The world economy volume 1: a millennial perspective, 2, Historical statistics: Academic Foundation, Gurgaon, India (2007)

46. Elias SG, Peeters PH, Grobbee DE, van Noord PA. The 1944-1945 Dutch famine and subsequent overall cancer incidence. Cancer Epidemiology Biomarkers & Prevention. 2005 Aug;14(8):1981-5.

47. Chiu YL, Wang XR, Qiu H, Yu IT. Risk factors for lung cancer: a case–control study in



For peer review only - http://bmjopen.bmj.com/site/about/guidelines.xhtml

495 Hong Kong women. Cancer Causes & Control. 2010 May;21(5):777-85.
496 48. Li J, Lam AS, Yau ST, Yiu KK, Tsoi KK. Antihypertensive treatments and risks of lung Cancer: A large population-based cohort study in Hong Kong. BMC cancer. 2021 Dec;21(1):1-9.
499 49. Du J, Sun H, Sun Y, Du J, Cao W, Sun S. Assessment of age, period, and cohort effects of lung cancer incidence in Hong Kong and projection up to 2030 based on changing demographics. American Journal of Cancer Research. 2021;11(12):5902.
502 50. *Centre for Health Protection, Department of Health - Lung Cancer*. Centre for Health Protection. Retrieved August 10, 2022, from https://www.chp.gov.hk/en/healthtopics/content/25/49.html

For peer review only



For peer review only - http://bmjopen.bmj.com/site/about/guidelines.xhtml

**Figure 1**. Parameter estimates of age (a), cohort (b) and period (c) effects based on an age-period-cohort model of male lung cancer mortality rates by immigrant groups: locals, immigrants stay in Hong Kong for more than 10 years and immigrants stay in Hong Kong for less than or equal to 10 years. Age effect was assessed by mortality (left axis). Cohort and period effects were assessed by relative risk (right axis), 95% confidence intervals are shown as shaded bands.

For peer review only



For peer review only - http://bmjopen.bmj.com/site/about/guidelines.xhtml

**Figure 2**. Parameter estimates of age (a), cohort (b) and period (c) effects based on an age-period-cohort model of female lung cancer mortality rates by immigrant groups: locals, immigrants stay in Hong Kong for more than 10 years and immigrants stay in Hong Kong for less than or equal to 10 years. Age effect was assessed by mortality (left axis). Cohort and period effects were assessed by relative risk (right axis), 95% confidence intervals are shown as shaded bands.

For peer review only



For peer review only - http://bmjopen.bmj.com/site/about/guidelines.xhtml

**Figure 3.** Projections of lung cancer mortality rates by gender and immigrant status from 2022 to 2030. Observations are shown as dots with the predictive distribution between the 5% and 95% quantile, whereby each lighter shade of red represents an additional 10% predictive CI. The predictive mean is shown as black solid line and the vertical dashed line indicates where prediction started.

For peer review only

**Figure 4.** Projections of lung cancer mortality rates for the population younger than 60 by gender and immigrant status from 2022 to 2030. Observations are shown as dots with the predictive distribution between the 5% and 95% quantile, whereby each lighter shade of red represents an additional 10% predictive CI. The predictive mean is shown as black solid line and the vertical dashed line indicates where prediction started.

For peer review only



For peer review only - http://bmjopen.bmj.com/site/about/guidelines.xhtml

**Figure 5.** Projections of lung cancer mortality rates for the population older than or equal to 60 by gender and immigrant status from 2022 to 2030. Observations are shown as dots with the predictive distribution between the 5% and 95% quantile, whereby each lighter shade of red represents an additional 10% predictive CI. The predictive mean is shown as black solid line and the vertical dashed line indicates where prediction started.

For peer review only



For peer review only - http://bmjopen.bmj.com/site/about/guidelines.xhtml

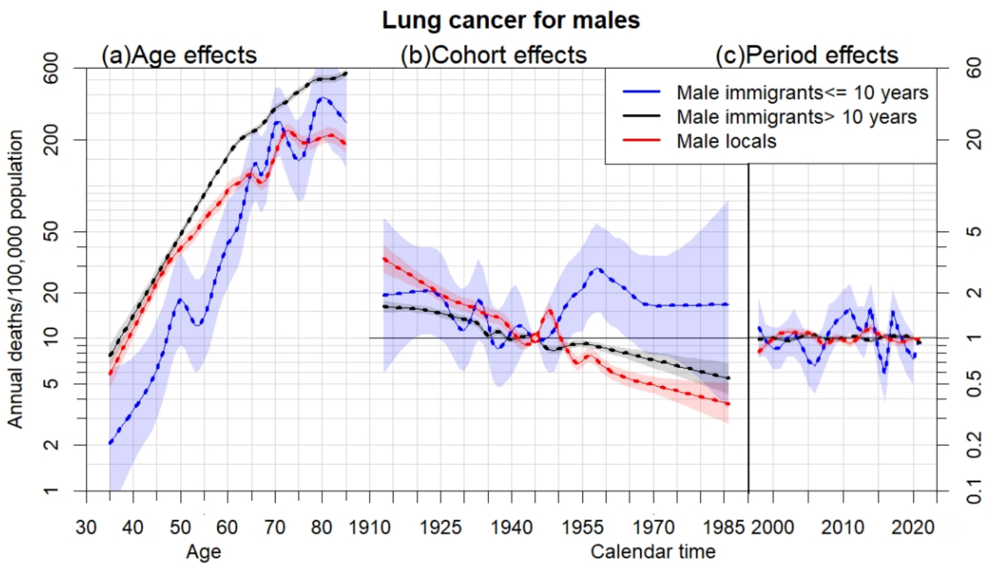


Figure 1. Parameter estimates of age (a), cohort (b) and period (c) effects based on an age-period-cohort model of male lung cancer mortality rates by immigrant groups: locals, immigrants stay in Hong Kong for more than 10 years and immigrants stay in Hong Kong for less than or equal to 10 years. Age effect was assessed by mortality (left axis). Cohort and period effects were assessed by relative risk (right axis), 95% confidence intervals are shown as shaded bands.

533x343mm (57 x 57 DPI)

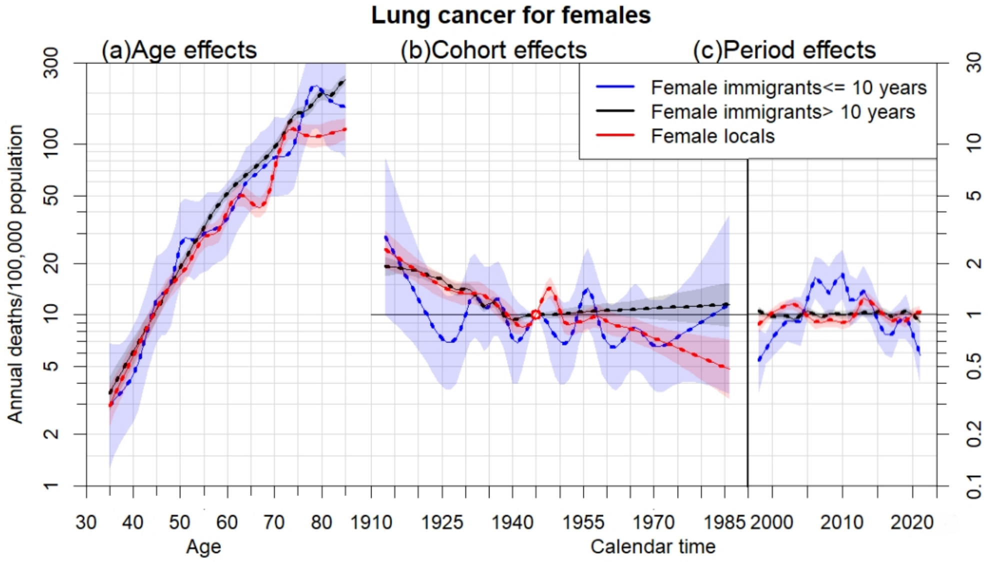


Figure 2. Parameter estimates of age (a), cohort (b) and period (c) effects based on an age-period-cohort model of female lung cancer mortality rates by immigrant groups: locals, immigrants stay in Hong Kong for more than 10 years and immigrants stay in Hong Kong for less than or equal to 10 years. Age effect was assessed by mortality (left axis). Cohort and period effects were assessed by relative risk (right axis), 95% confidence intervals are shown as shaded bands.

533x343mm (57 x 57 DPI)

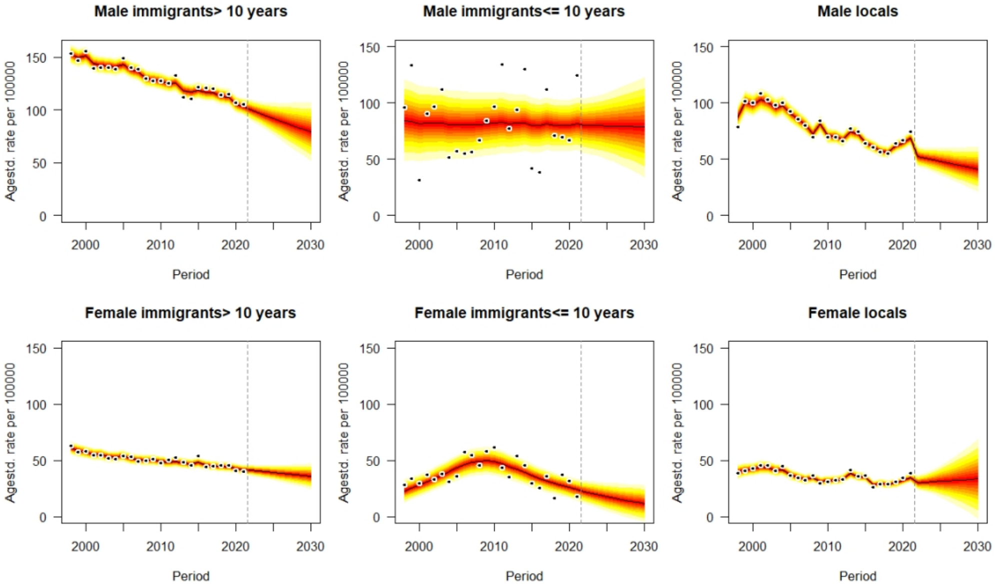


Figure 3. Projections of lung cancer mortality rates by gender and immigrant status from 2022 to 2030. Observations are shown as dots with the predictive distribution between the 5% and 95% quantile, whereby each lighter shade of red represents an additional 10% predictive CI. The predictive mean is shown as black solid line and the vertical dashed line indicates where prediction started.

570x343mm (57 x 57 DPI)

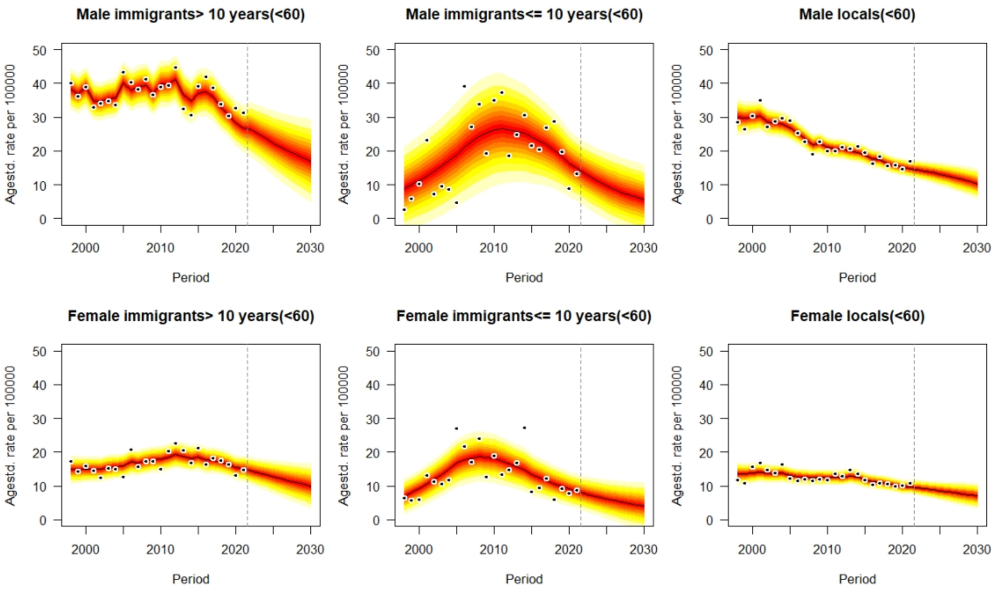


Figure 4. Projections of lung cancer mortality rates for the population younger than 60 by gender and immigrant status from 2022 to 2030. Observations are shown as dots with the predictive distribution between the 5% and 95% quantile, whereby each lighter shade of red represents an additional 10% predictive CI. The predictive mean is shown as black solid line and the vertical dashed line indicates where prediction started.

570x343mm (57 x 57 DPI)

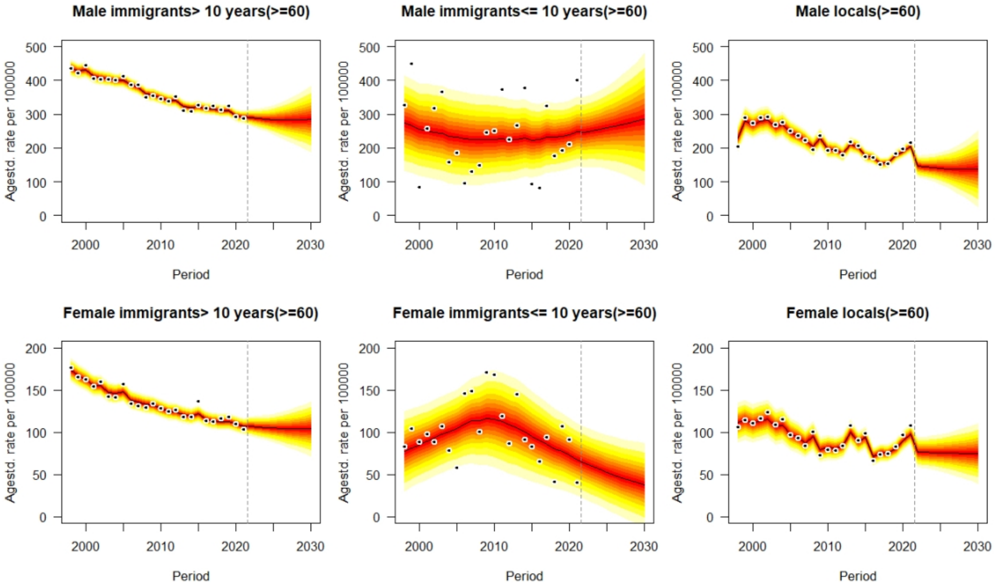


Figure 5. Projections of lung cancer mortality rates for the population older than or equal to 60 by gender and immigrant status from 2022 to 2030. Observations are shown as dots with the predictive distribution between the 5% and 95% quantile, whereby each lighter shade of red represents an additional 10% predictive CI. The predictive mean is shown as black solid line and the vertical dashed line indicates where prediction started.

570x343mm (57 x 57 DPI)

# Supplementary Material for

# "An age-period-cohort analysis and projection of cancer mortality in Hong Kong, 1998–2030"

For peer review only

## APC Figures

eFigure 1(a) Colon cancer ........ 3
eFigure 1(b) Liver cancer ........ 4
eFigure 1(c) Pancreatic cancer ........ 5
eFigure 1(d) Stomach cancer ........ 6
eFigure 1(e) Prostate cancer ........ 7

## Projection Figures

Colon cancer
eFigure 2(a)Projection (all ages) ........ 8
eFigure 2(b)Projection (<60 years) ........ 9
eFigure 2(c)Projection (⩾60 years) ........ 10
Liver cancer
eFigure 3(a)Projection (all ages) ........ 11
eFigure 3(b)Projection (<60 years) ........ 12
eFigure 3(c)Projection (⩾60 years) ........ 13
Pancreatic cancer
eFigure 4(a)Projection (all ages) ........ 14
eFigure 4(b)Projection (<60 years) ........ 15
eFigure 4(c)Projection (⩾60 years) ........ 16
Stomach cancer
eFigure 5(a)Projection (all ages) ........ 17
eFigure 5(b)Projection (<60 years) ........ 18



For peer review only - http://bmjopen.bmj.com/site/about/guidelines.xhtml

eFigure 5(c)Projection (⩾60 years) ........ 19

Prostate cancer

eFigure 6 Projection (all ages, <60 years and ⩾60 years) ........ 20

## Tables

eTable 1 Lung cancer ........ 21

eTable 2 Colon cancer ........ 22

eTable 3 Liver cancer ........ 23

eTable 4 Pancreatic cancer ........ 24

eTable 5 Stomach cancer ........ 25

eTable 6 Prostate cancer ........ 26

For peer review only



For peer review only - http://bmjopen.bmj.com/site/about/guidelines.xhtml

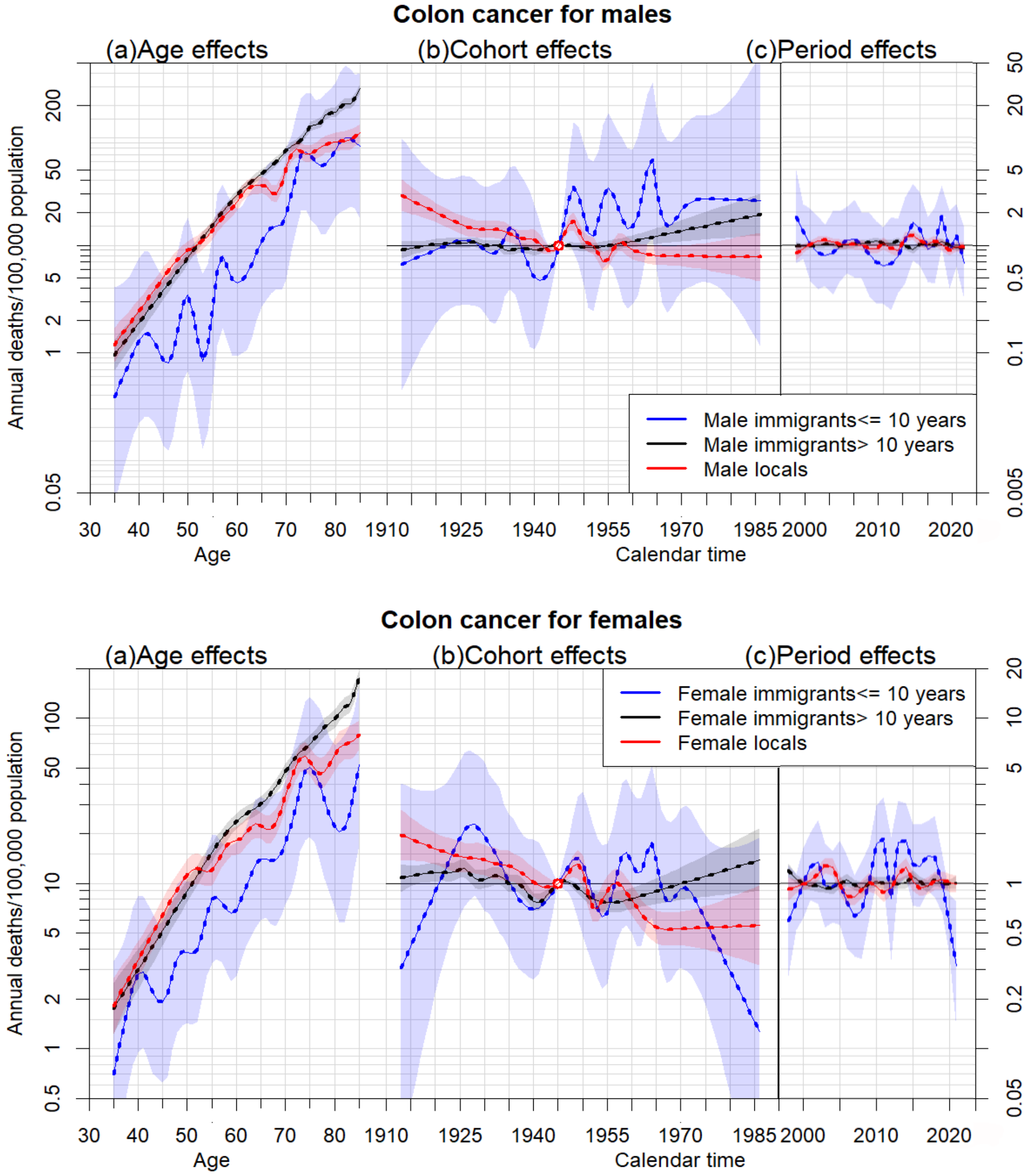


**eFigure 1(a)**. Parameter estimates of age (a), cohort (b) and period (c) effects based on an age-period-cohort model of male and female colon cancer mortality rates by immigrant groups: locals, immigrants stay in Hong Kong for more than 10 years and immigrants stay in Hong Kong for less than or equal to 10 years. Age effect was assessed by mortality (left axis). Cohort and period effects were assessed by relative risk (right axis), 95% confidence intervals are shown as shaded bands.



For peer review only - http://bmjopen.bmj.com/site/about/guidelines.xhtml

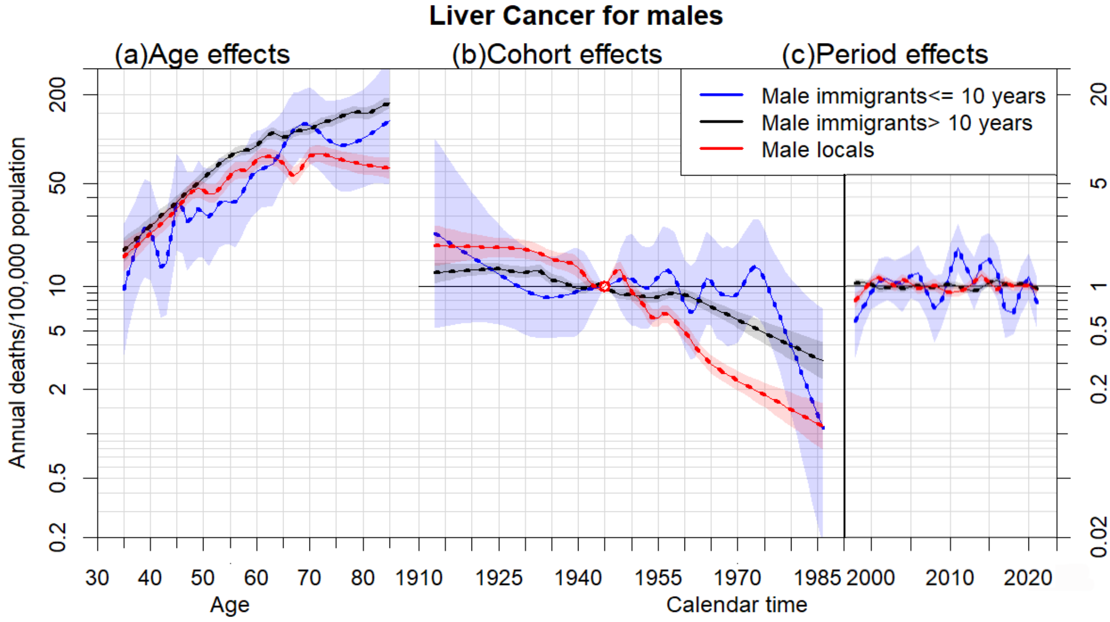


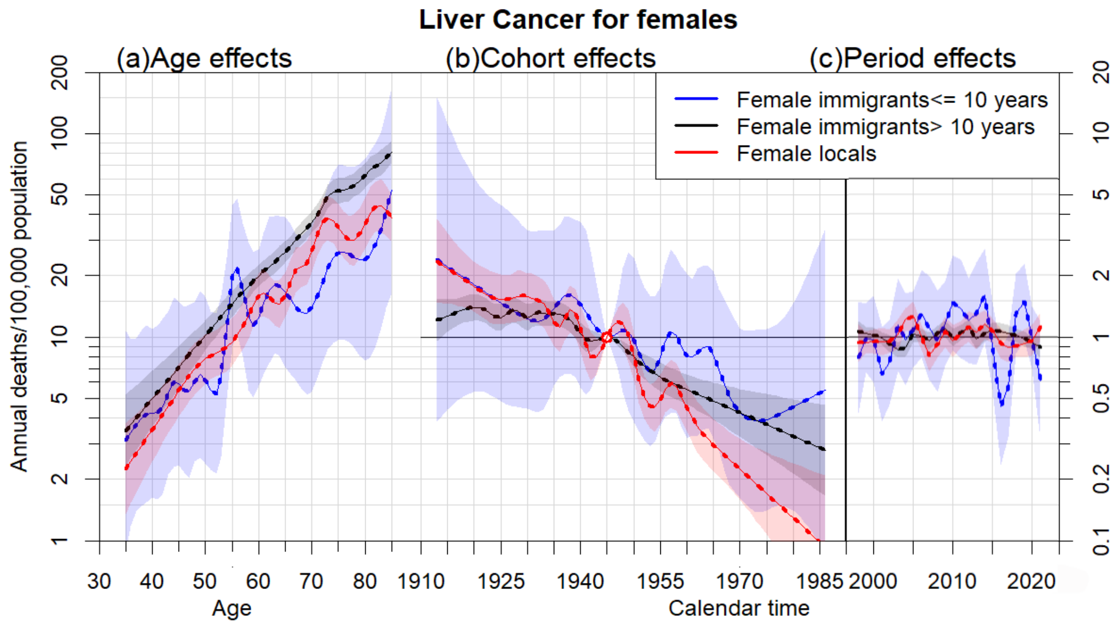


**eFigure 1(b)**. Parameter estimates of age (a), cohort (b) and period (c) effects based on an age-period-cohort model of male and female liver cancer mortality rates by immigrant groups: locals, immigrants stay in Hong Kong for more than 10 years and immigrants stay in Hong Kong for less than or equal to 10 years. Age effect was assessed by mortality (left axis). Cohort and period effects were assessed by relative risk (right axis), 95% confidence intervals are shown as shaded bands.



For peer review only - http://bmjopen.bmj.com/site/about/guidelines.xhtml

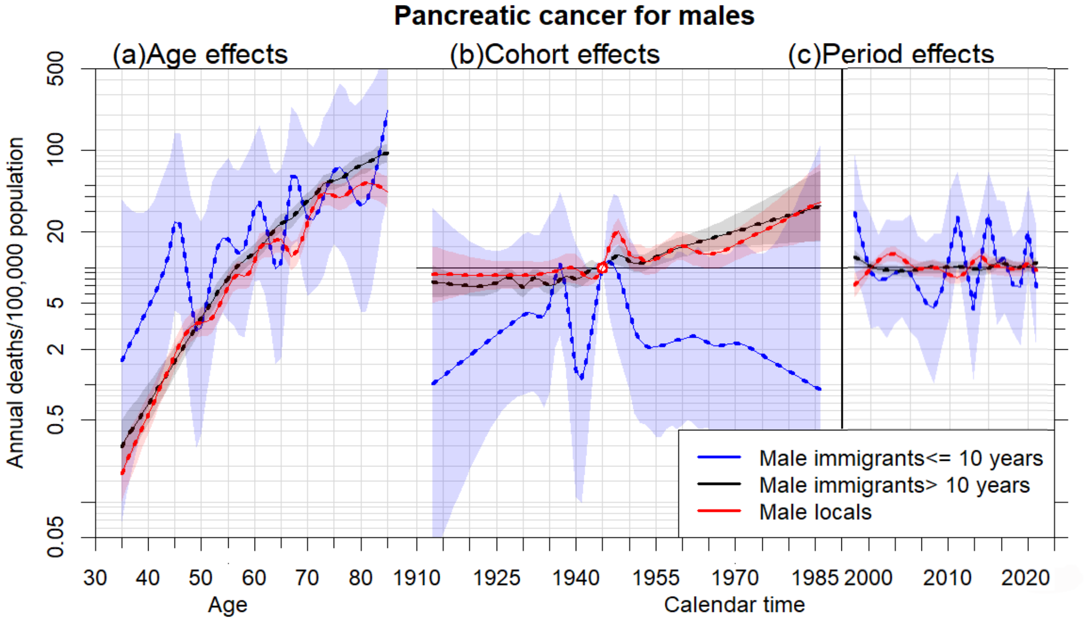


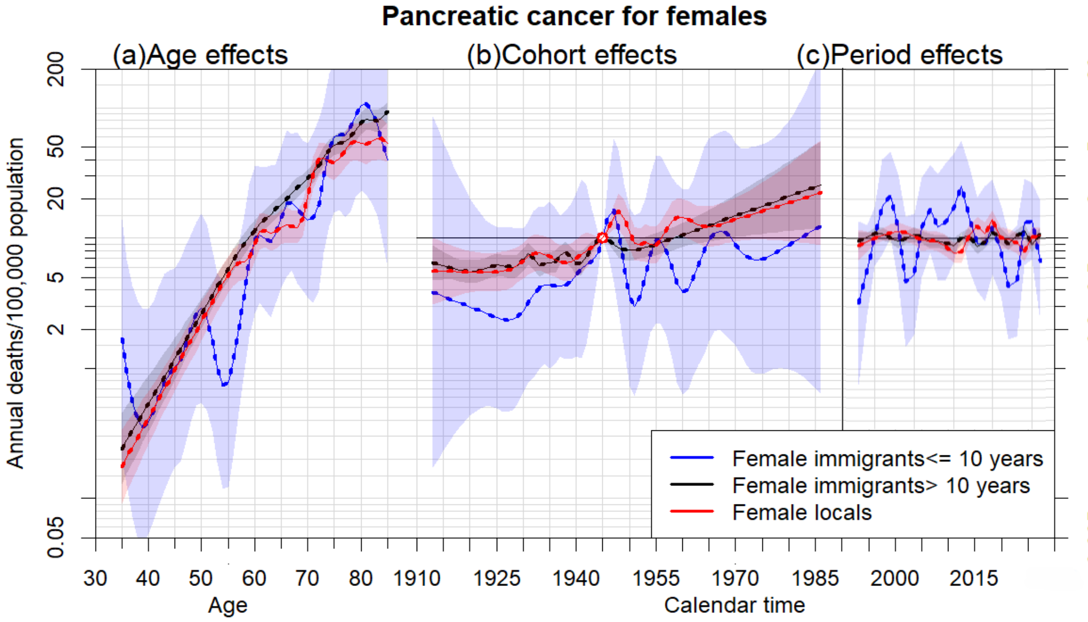


**eFigure 1(c)**. Parameter estimates of age (a), cohort (b) and period (c) effects based on an age-period-cohort model of male and female pancreatic cancer mortality rates by immigrant groups: locals, immigrants stay in Hong Kong for more than 10 years and immigrants stay in Hong Kong for less than or equal to 10 years. Age effect was assessed by mortality (left axis). Cohort and period effects were assessed by relative risk (right axis), 95% confidence intervals are shown as shaded bands.



For peer review only - http://bmjopen.bmj.com/site/about/guidelines.xhtml

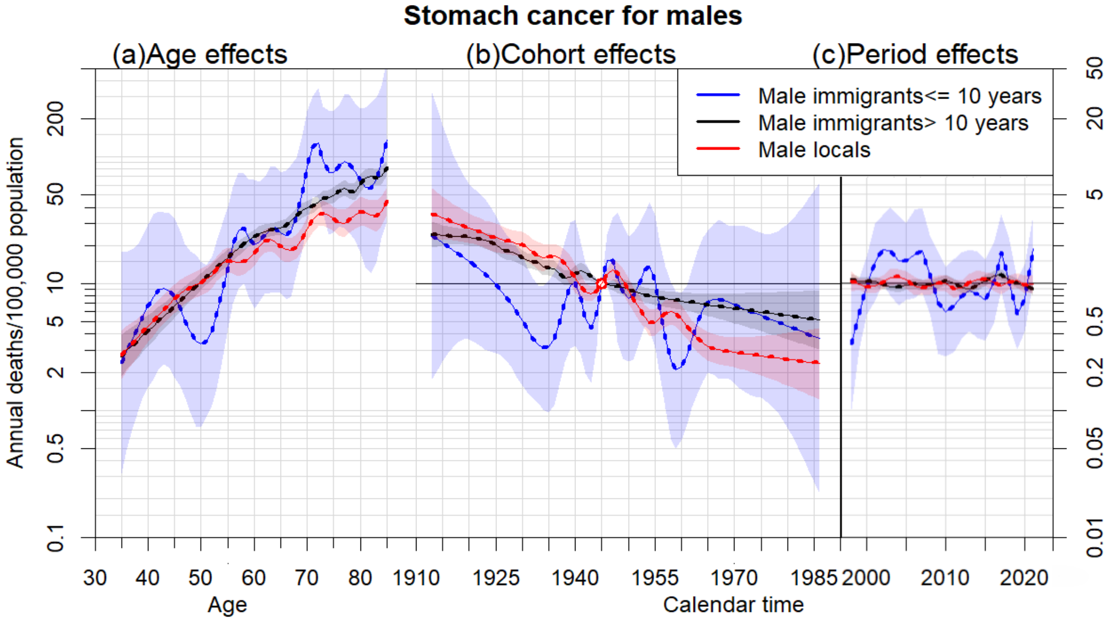


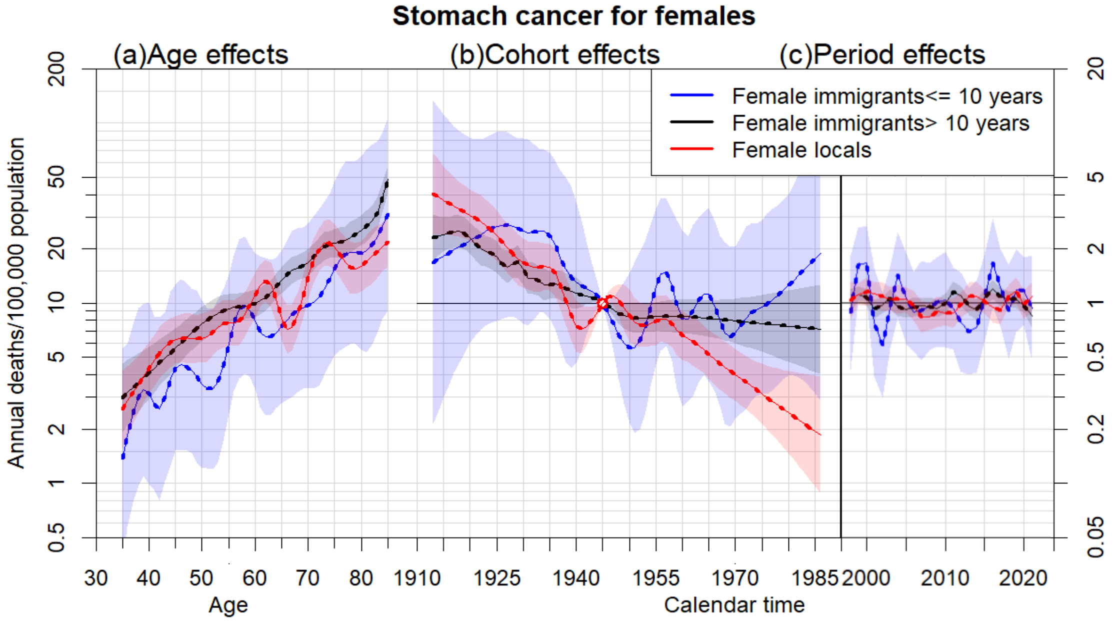


**eFigure 1(d)**. Parameter estimates of age (a), cohort (b) and period (c) effects based on an age-period-cohort model of male and female stomach cancer mortality rates by immigrant groups: locals, immigrants stay in Hong Kong for more than 10 years and immigrants stay in Hong Kong for less than or equal to 10 years. Age effect was assessed by mortality (left axis). Cohort and period effects were assessed by relative risk (right axis), 95% confidence intervals are shown as shaded bands.



For peer review only - http://bmjopen.bmj.com/site/about/guidelines.xhtml

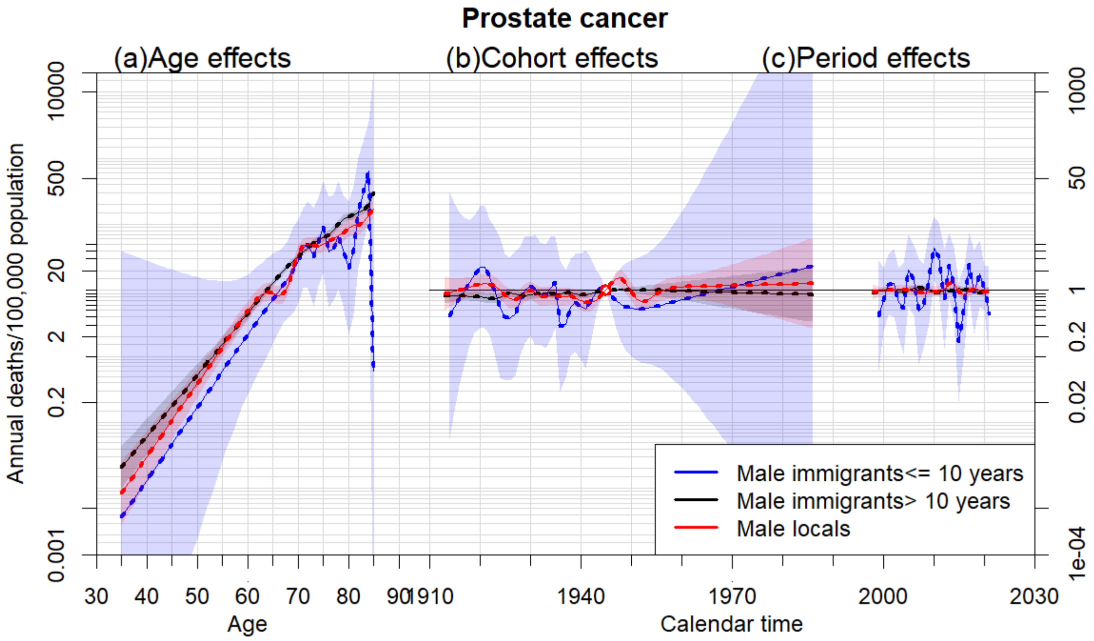


**eFigure 1(e)**. Parameter estimates of age (a), cohort (b) and period (c) effects based on an age-period-cohort model of male prostate cancer mortality rates by immigrant groups: locals, immigrants stay in Hong Kong for more than 10 years and immigrants stay in Hong Kong for less than or equal to 10 years. Age effect was assessed by mortality (left axis). Cohort and period effects were assessed by relative risk (right axis), 95% confidence intervals are shown as shaded bands.



For peer review only - http://bmjopen.bmj.com/site/about/guidelines.xhtml

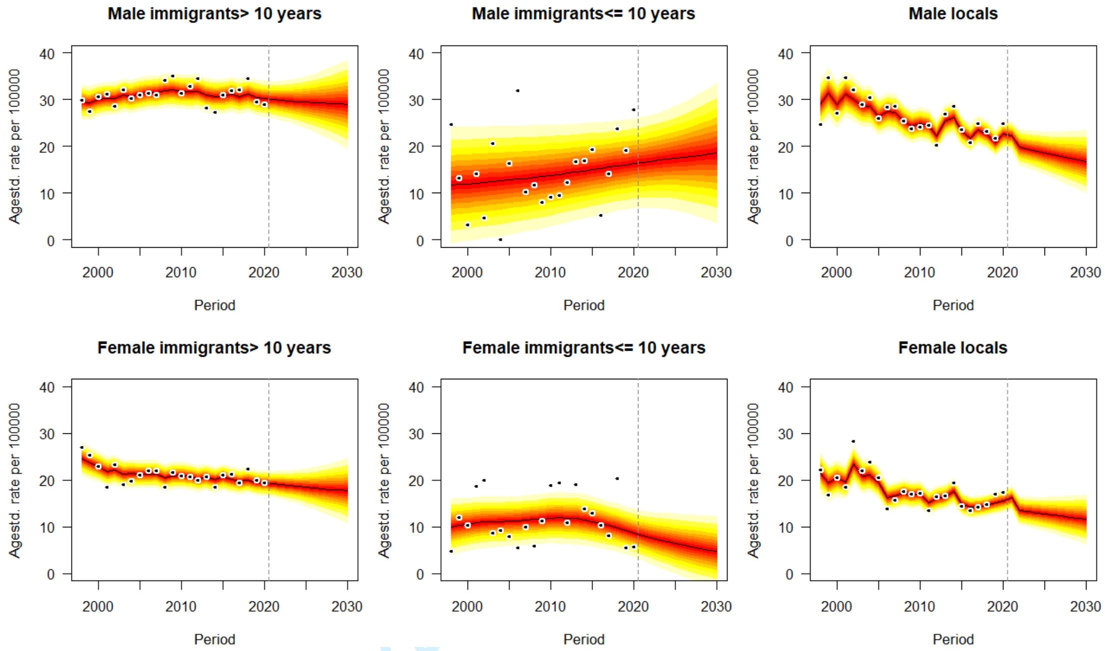


**eFigure 2(a).** Projections of colon cancer mortality rates by gender and immigrant status from 2022 to 2030. Observations are shown as dots with the predictive distribution between the 5% and 95% quantile, whereby each lighter shade of red represents an additional 10% predictive CI. The predictive mean is shown as black solid line and the vertical dashed line indicates where prediction started.



For peer review only - http://bmjopen.bmj.com/site/about/guidelines.xhtml

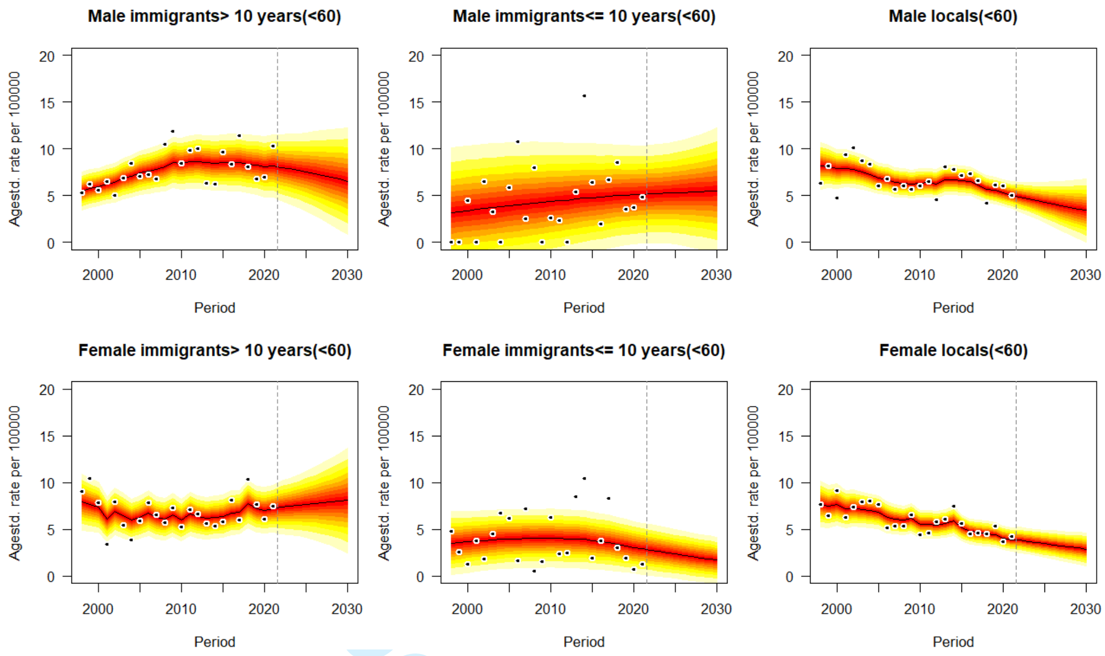


**eFigure 2(b).** Projections of colon cancer mortality rates for the population younger than 60 by gender and immigrant status from 2022 to 2030. Observations are shown as dots with the predictive distribution between the 5% and 95% quantile, whereby each lighter shade of red represents an additional 10% predictive CI. The predictive mean is shown as black solid line and the vertical dashed line indicates where prediction started.



For peer review only - http://bmjopen.bmj.com/site/about/guidelines.xhtml

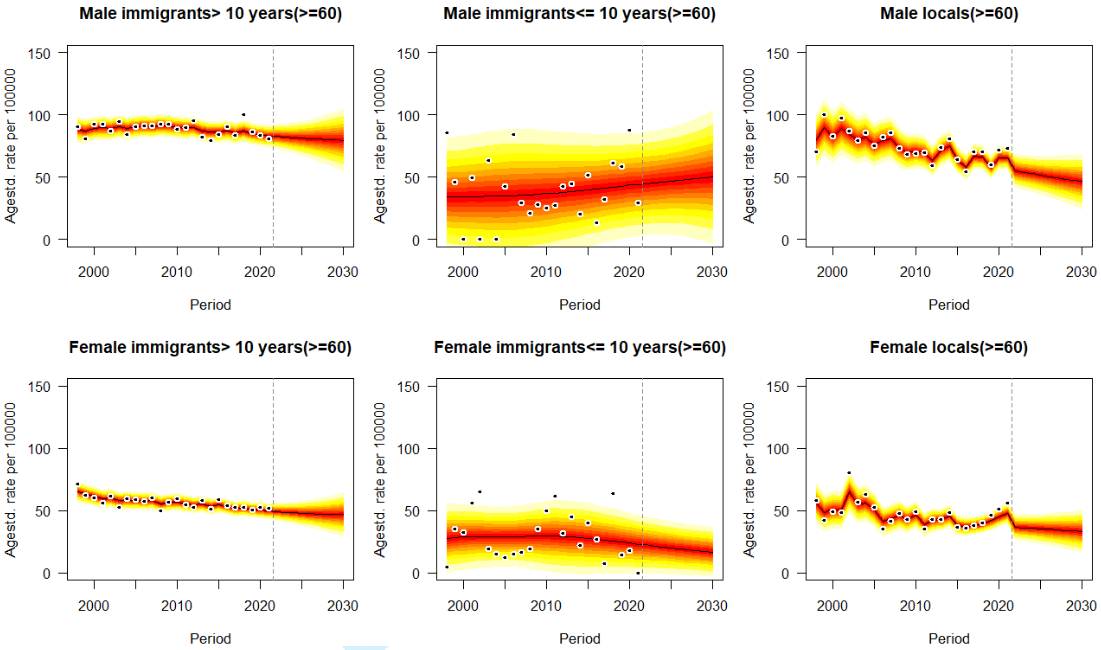


**eFigure 2(c).** Projections of colon cancer mortality rates for the population older than or equal to 60 by gender and immigrant status from 2022 to 2030. Observations are shown as dots with the predictive distribution between the 5% and 95% quantile, whereby each lighter shade of red represents an additional 10% predictive CI. The predictive mean is shown as black solid line and the vertical dashed line indicates where prediction started.



For peer review only - http://bmjopen.bmj.com/site/about/guidelines.xhtml

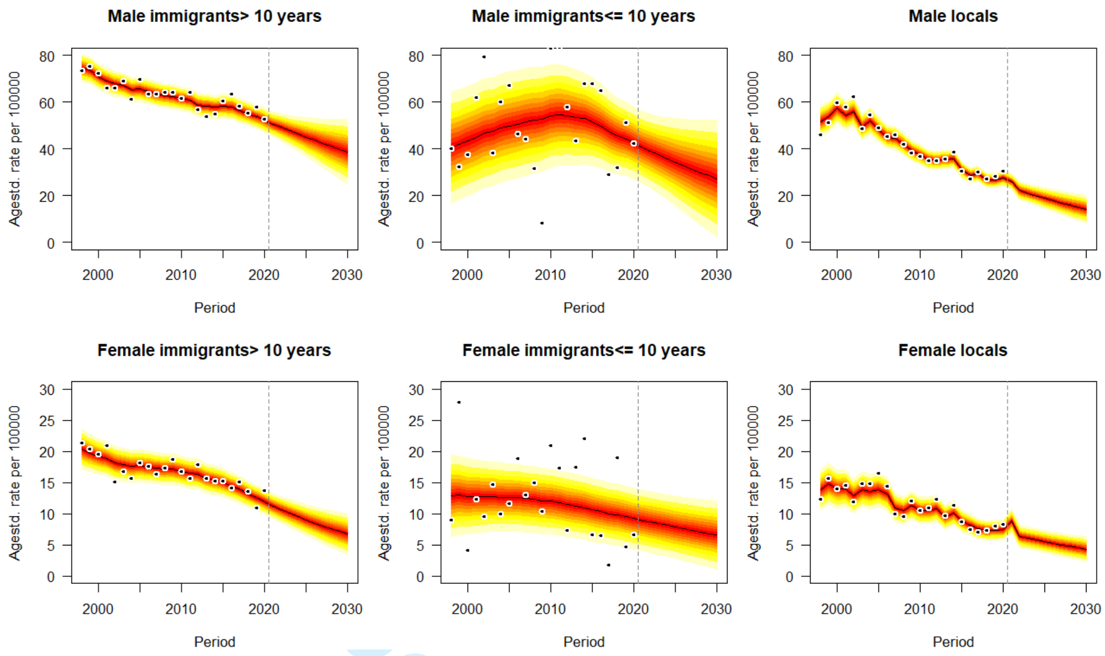


**eFigure 3(a).** Projections of liver cancer mortality rates by gender and immigrant status from 2022 to 2030. Observations are shown as dots with the predictive distribution between the 5% and 95% quantile, whereby each lighter shade of red represents an additional 10% predictive CI. The predictive mean is shown as black solid line and the vertical dashed line indicates where prediction started.



For peer review only - http://bmjopen.bmj.com/site/about/guidelines.xhtml

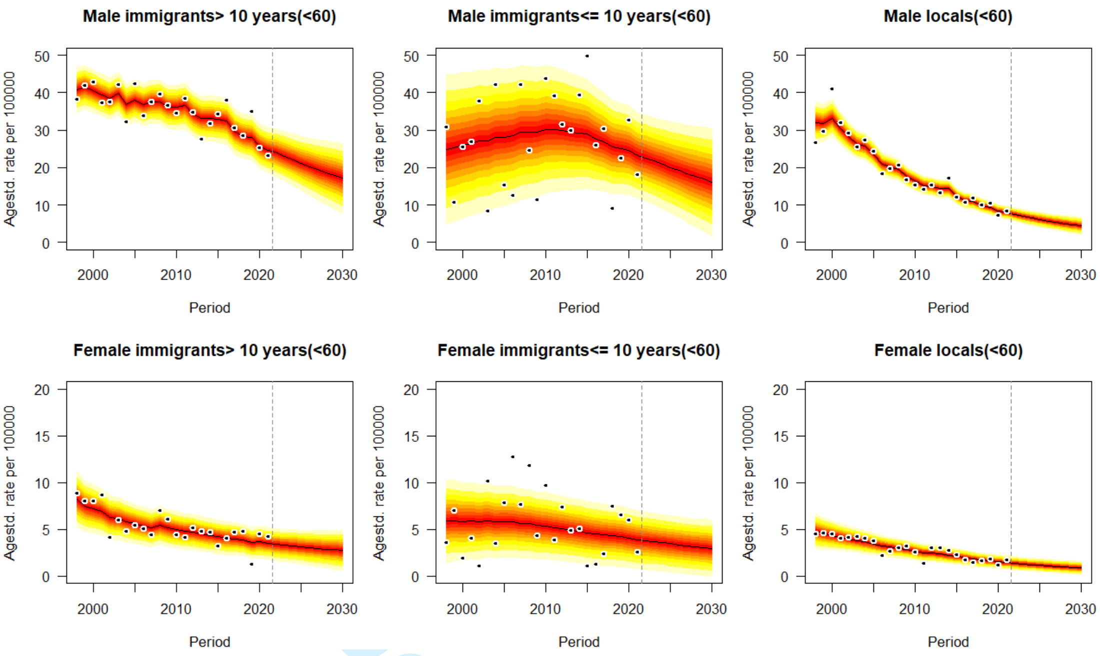


**eFigure 3(b).** Projections of liver cancer mortality rates for the population younger than 60 by gender and immigrant status from 2022 to 2030. Observations are shown as dots with the predictive distribution between the 5% and 95% quantile, whereby each lighter shade of red represents an additional 10% predictive CI. The predictive mean is shown as black solid line and the vertical dashed line indicates where prediction started.

eer review only



For peer review only - http://bmjopen.bmj.com/site/about/guidelines.xhtml

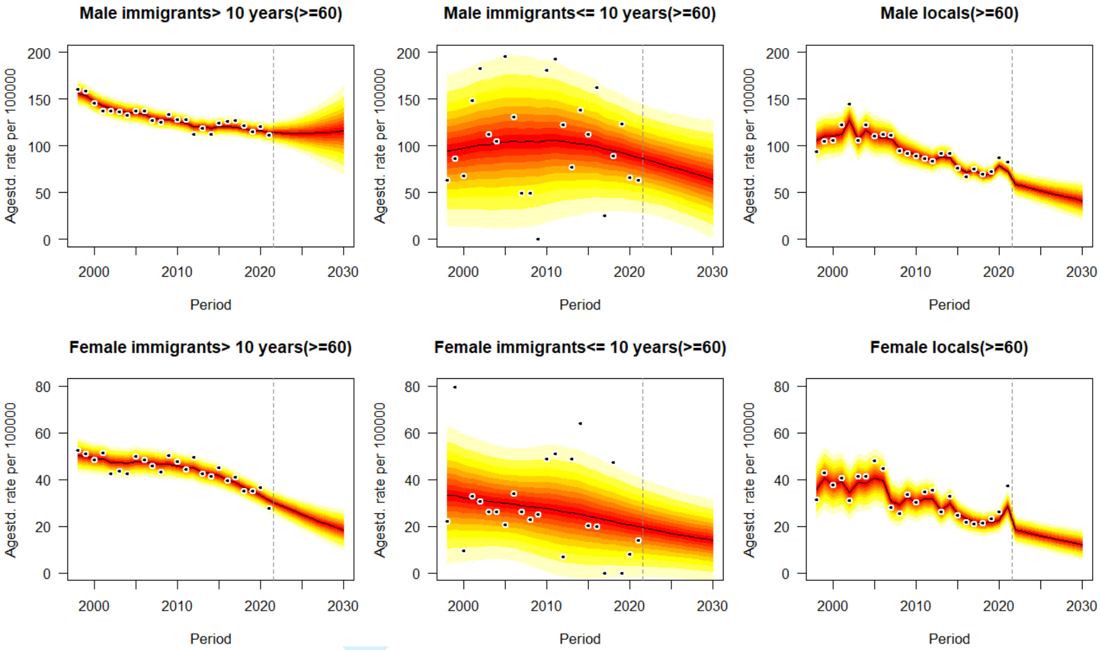


**eFigure 3(c).** Projections of liver cancer mortality rates for the population older than or equal to 60 by gender and immigrant status from 2022 to 2030. Observations are shown as dots with the predictive distribution between the 5% and 95% quantile, whereby each lighter shade of red represents an additional 10% predictive CI. The predictive mean is shown as black solid line and the vertical dashed line indicates where prediction started.



For peer review only - http://bmjopen.bmj.com/site/about/guidelines.xhtml

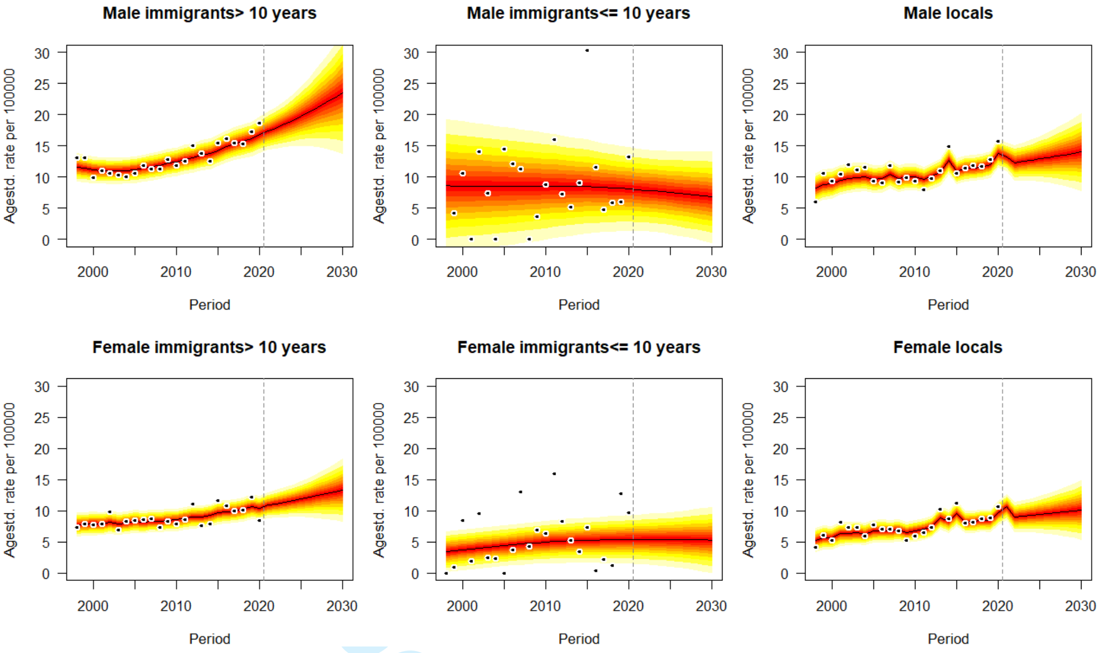


**eFigure 4(a).** Projections of pancreatic cancer mortality rates by gender and immigrant status from 2022 to 2030. Observations are shown as dots with the predictive distribution between the 5% and 95% quantile, whereby each lighter shade of red represents an additional 10% predictive CI. The predictive mean is shown as black solid line and the vertical dashed line indicates where prediction started.

For peer review only



For peer review only - http://bmjopen.bmj.com/site/about/guidelines.xhtml

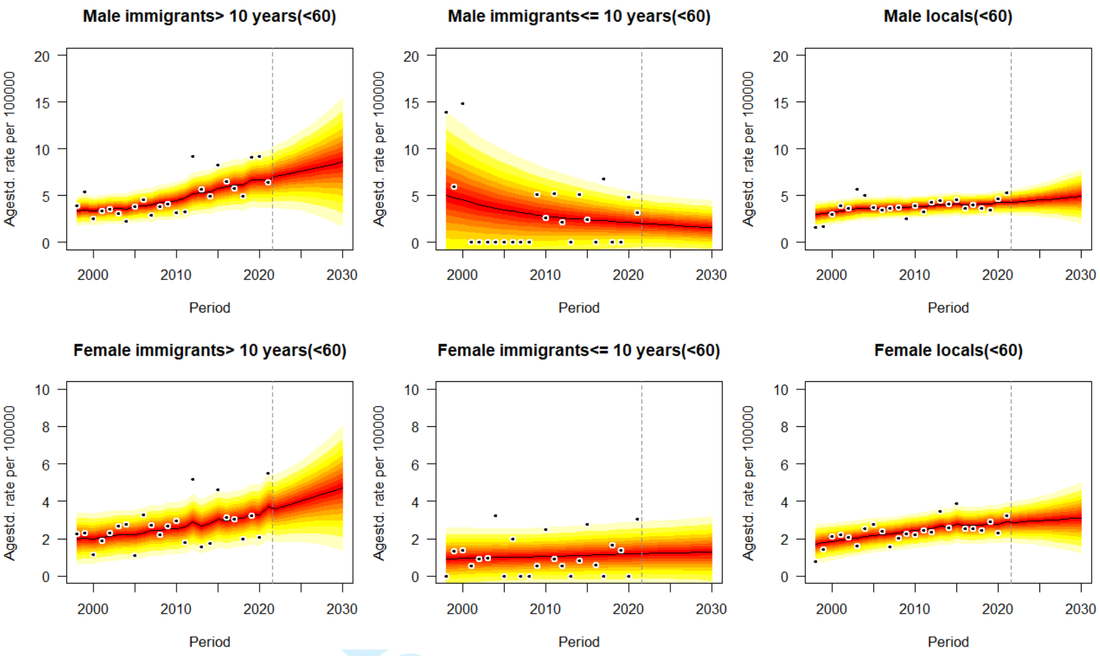


**eFigure 4(b).** Projections of pancreatic cancer mortality rates for the population younger than 60 by gender and immigrant status from 2022 to 2030. Observations are shown as dots with the predictive distribution between the 5% and 95% quantile, whereby each lighter shade of red represents an additional 10% predictive CI. The predictive mean is shown as black solid line and the vertical dashed line indicates where prediction started.

For peer review only



For peer review only - http://bmjopen.bmj.com/site/about/guidelines.xhtml

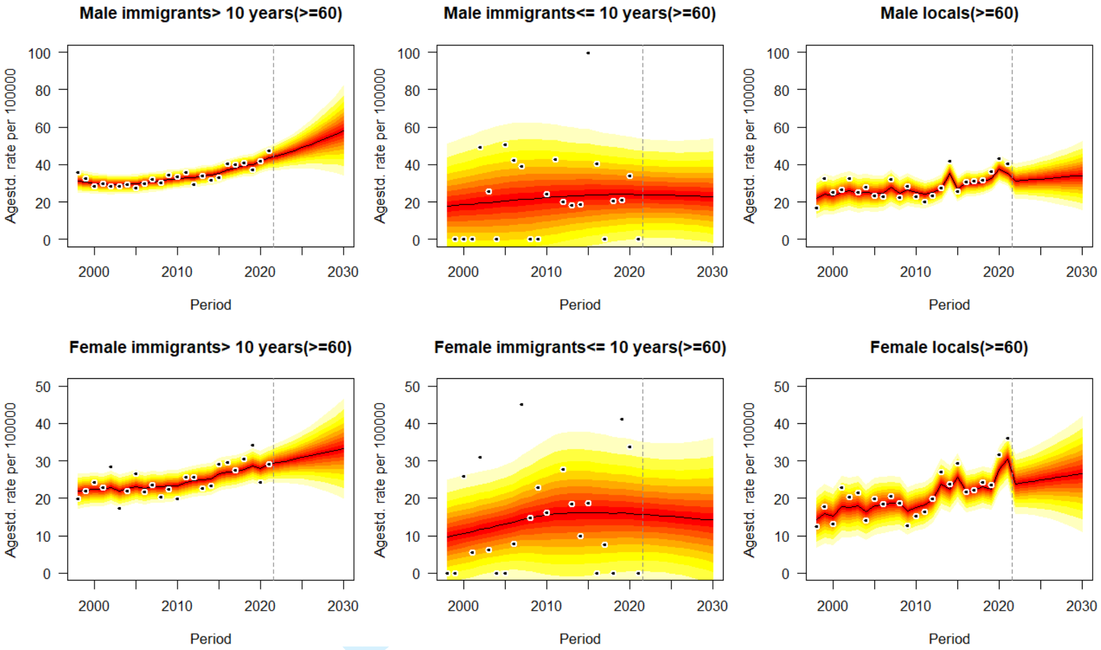


**eFigure 4(c).** Projections of pancreatic cancer mortality rates for the population older than or equal to 60 by gender and immigrant status from 2022 to 2030. Observations are shown as dots with the predictive distribution between the 5% and 95% quantile, whereby each lighter shade of red represents an additional 10% predictive CI. The predictive mean is shown as black solid line and the vertical dashed line indicates where prediction started.



For peer review only - http://bmjopen.bmj.com/site/about/guidelines.xhtml

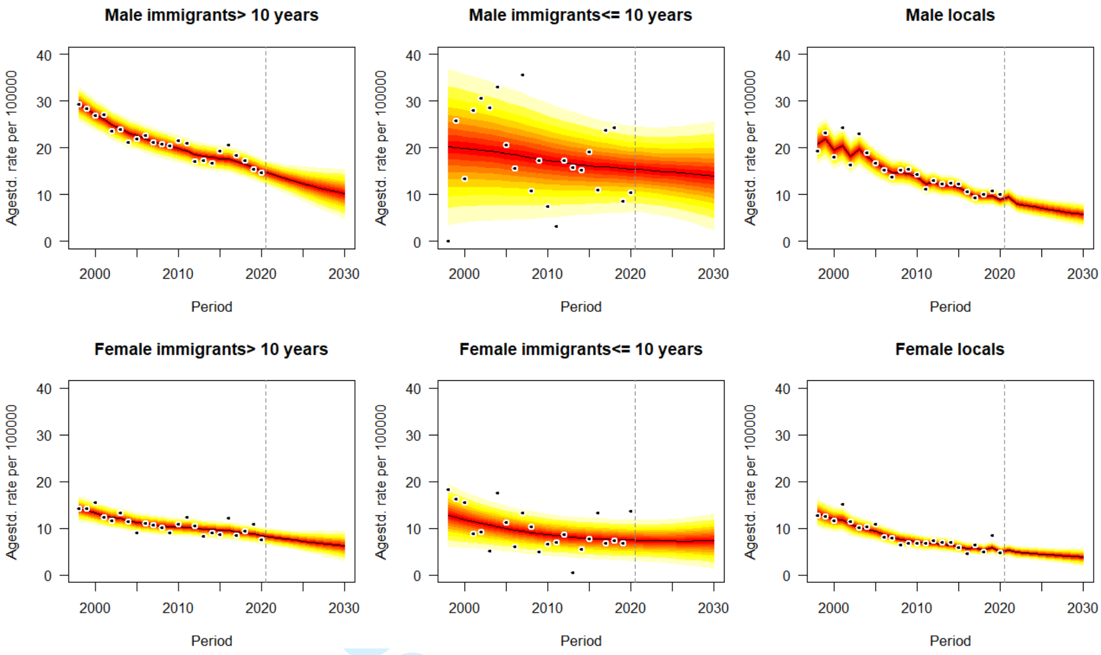


**eFigure 5(a).** Projections of stomach cancer mortality rates by gender and immigrant status from 2022 to 2030. Observations are shown as dots with the predictive distribution between the 5% and 95% quantile, whereby each lighter shade of red represents an additional 10% predictive CI. The predictive mean is shown as black solid line and the vertical dashed line indicates where prediction started.



For peer review only - http://bmjopen.bmj.com/site/about/guidelines.xhtml

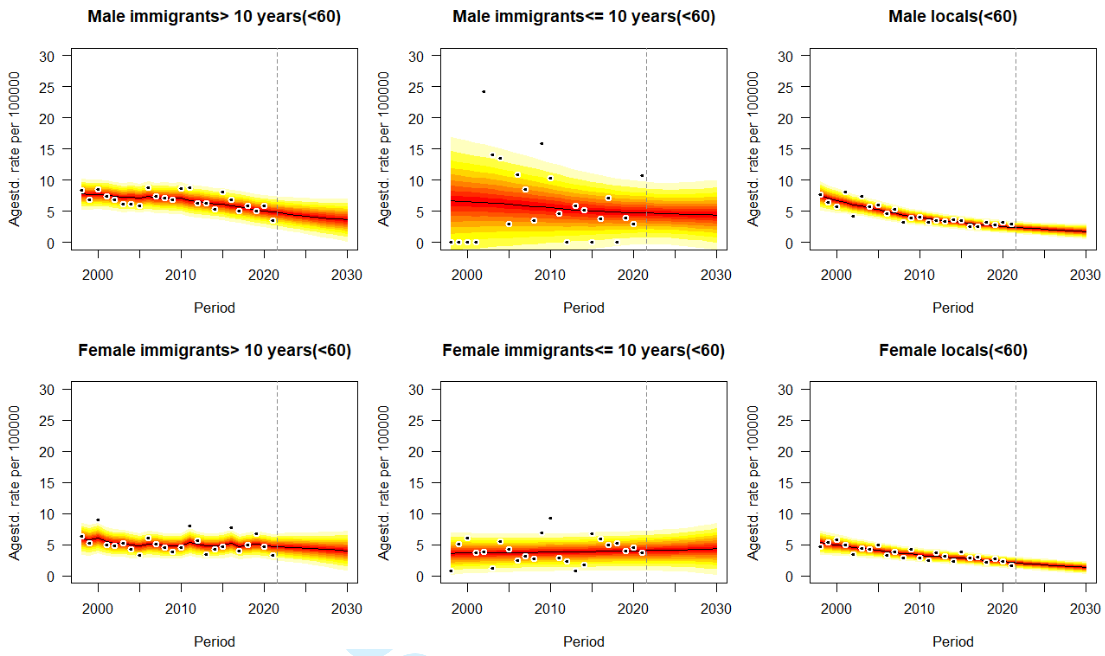


**eFigure 5(b).** Projections of stomach cancer mortality rates for the population younger than 60 by gender and immigrant status from 2022 to 2030. Observations are shown as dots with the predictive distribution between the 5% and 95% quantile, whereby each lighter shade of red represents an additional 10% predictive CI. The predictive mean is shown as black solid line and the vertical dashed line indicates where prediction started.



For peer review only - http://bmjopen.bmj.com/site/about/guidelines.xhtml

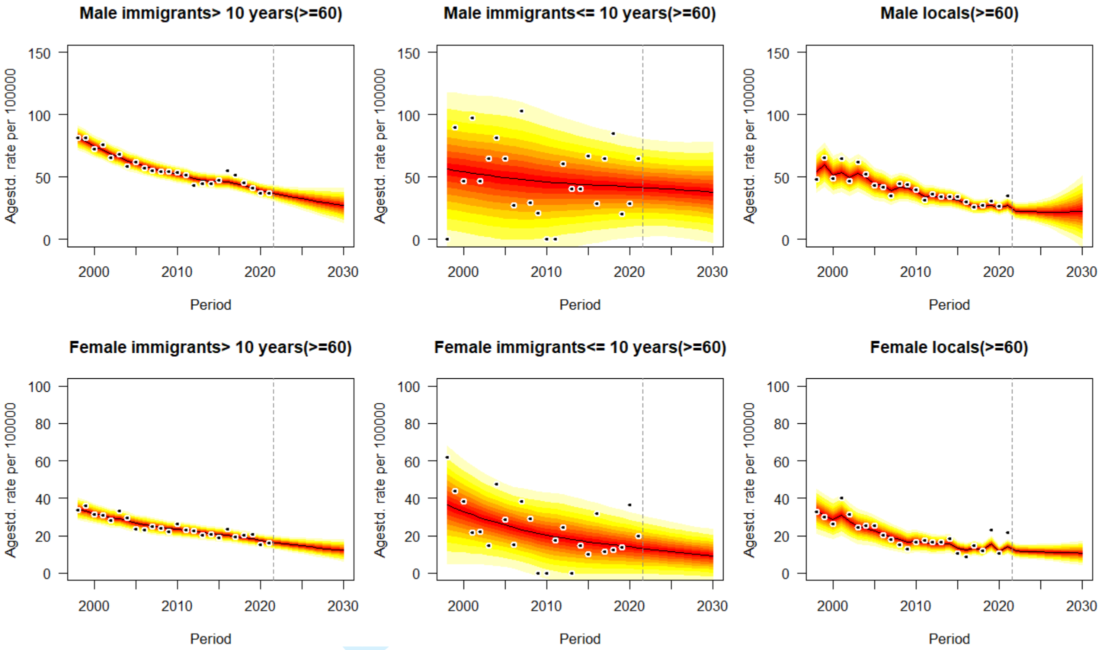


**eFigure 5(c).** Projections of stomach cancer mortality rates for the population older than or equal to 60 by gender and immigrant status from 2022 to 2030. Observations are shown as dots with the predictive distribution between the 5% and 95% quantile, whereby each lighter shade of red represents an additional 10% predictive CI. The predictive mean is shown as black solid line and the vertical dashed line indicates where prediction started.



For peer review only - http://bmjopen.bmj.com/site/about/guidelines.xhtml

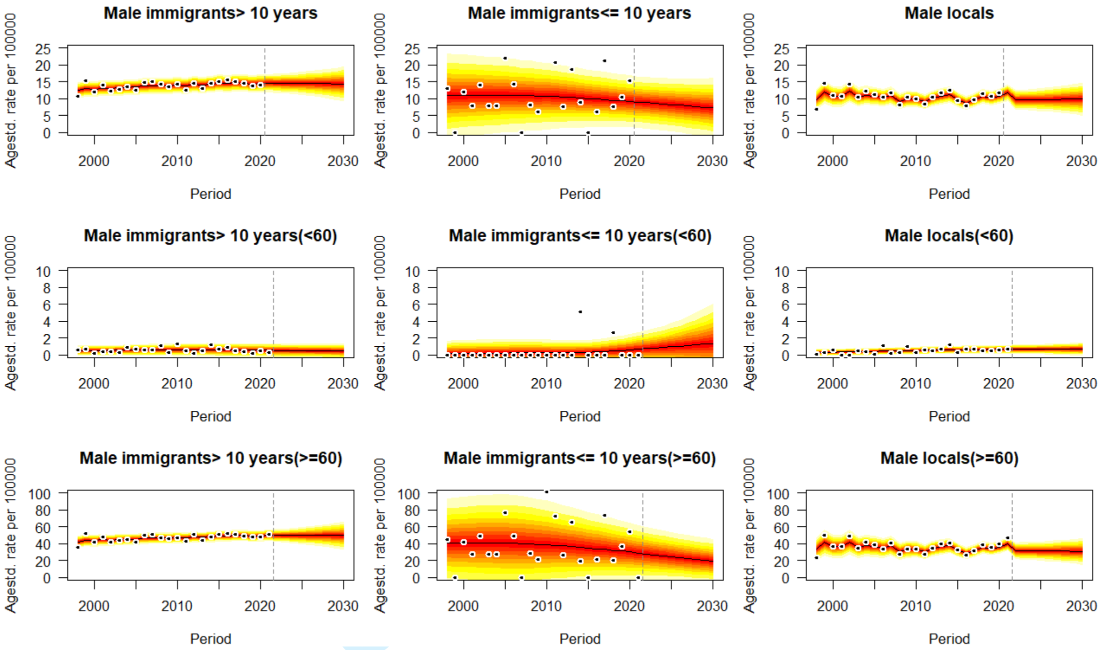


**eFigure 6.** Projections of prostate cancer mortality rates for males by immigrant status and age groups (less than, greater than or equal to 60 years old) from 2022 to 2030. Observations are shown as dots with the predictive distribution between the 5% and 95% quantile, whereby each lighter shade of red represents an additional 10% predictive CI. The predictive mean is shown as black solid line and the vertical dashed line indicates where prediction started.



For peer review only - http://bmjopen.bmj.com/site/about/guidelines.xhtml

| Predictive mean of age-standardized mortality rates of lung cancer per 100,000 population | | | | | | | | | | |
|---|---|---|---|---|---|---|---|---|---|---|
| **Year** | **2021** | **2022** | **2023** | **2024** | **2025** | **2026** | **2027** | **2028** | **2029** | **2030** |
| **Female immigrants >10** | 41.80 (1.27) | 41.34 (1.86) | 40.58 (2.27) | 39.87 (2.75) | 39.19 (3.28) | 38.53 (3.86) | 37.89 (4.46) | 37.26 (5.09) | 36.65 (5.74) | 36.04 (6.4) |
| **Female immigrants ≤ 10** | 23.92 (4.00) | 22.22 (4.67) | 20.56 (5.38) | 19.01 (6.10) | 17.57 (6.80) | 16.24 (7.45) | 15.00 (8.04) | 13.85 (8.56) | 12.79 (9.01) | 11.81 (9.39) |
| **Female locals** | 34.67 (1.76) | 30.22 (3.54) | 30.63 (4.77) | 31.05 (6.38) | 31.48 (8.29) | 31.9 (10.47) | 32.32 (12.87) | 32.73 (15.48) | 33.15 (18.31) | 33.55 (21.33) |
| **Male immigrants >10** | 102.90 (2.43) | 100.18 (4.18) | 97.18 (5.33) | 94.34 (6.72) | 91.71 (8.24) | 89.15 (9.84) | 86.66 (11.47) | 84.19 (13.11) | 81.81 (14.74) | 79.55 (16.37) |
| **Male immigrants ≤10** | 81.26 (9.21) | 79.90 (10.41) | 79.81 (11.82) | 79.72 (13.42) | 79.62 (15.19) | 79.50 (17.09) | 79.32 (19.09) | 79.08 (21.18) | 78.78 (23.32) | 78.41 (25.53) |
| **Male locals** | 60.96 (2.82) | 52.27 (4.86) | 50.83 (5.39) | 49.56 (6.13) | 48.18 (6.97) | 46.64 (7.84) | 45.13 (8.76) | 43.83 (9.76) | 42.67 (10.8) | 41.43 (11.8) |
| **Female immigrants>10(<60y)** | 15.51 (1.12) | 14.51 (1.50) | 13.90 (1.76) | 13.29 (2.04) | 12.71 (2.33) | 12.13 (2.62) | 11.57 (2.91) | 11.02 (3.18) | 10.49 (3.43) | 9.98 (3.68) |
| **Female immigrants ≤ 10(<60y)** | 8.14 (1.91) | 7.79 (1.95) | 7.18(2.23) | 6.62(2.53) | 6.10(2.81) | 5.63(3.08) | 5.19(3.32) | 4.79 (3.53) | 4.42 (3.72) | 4.09 (3.88) |
| **Female locals(<60y)** | 10.25 (0.77) | 9.48 (0.89) | 9.17(1.02) | 8.87(1.16) | 8.57(1.32) | 8.27(1.49) | 7.97(1.65) | 7.68 (1.82) | 7.38 (1.98) | 7.09 (2.13) |
| **Male immigrants>10(<60y)** | 27.81 (2.10) | 26.36 (3.58) | 24.96 (3.94) | 23.64 (4.35) | 22.38 (4.79) | 21.17 (5.23) | 20.03 (5.67) | 18.96 (6.10) | 17.96 (6.51) | 17.03 (6.90) |
| **Male immigrants ≤ 10(<60y)** | 15.01 (2.98) | 13.38 (3.71) | 12.02 (4.17) | 10.79 (4.59) | 9.68 (4.95) | 8.69 (5.24) | 7.79 (5.46) | 6.98 (5.61) | 6.25 (5.69) | 5.59 (5.72) |
| **Male locals(<60y)** | 15.19 (0.78) | 14.45 (1.15) | 14.03 (1.29) | 13.61 (1.46) | 13.14 (1.64) | 12.65 (1.82) | 12.13 (2.01) | 11.55 (2.17) | 10.93 (2.31) | 10.26 (2.43) |
| **Female immigrants >10(≥ 60y)** | 108.85 (4.80) | 107.21 (5.17) | 106.26 (6.24) | 105.52 (7.54) | 104.94 (9.04) | 104.51 (10.72) | 104.21 (12.57) | 104.07 (14.61) | 104.06 (16.78) | 104.16 (19.14) |
| **Female immigrants ≤ 10(≥60y)** | 66.16 (13.25) | 63.84 (15.72) | 59.88 (17.50) | 56.14 (19.31) | 52.60 (21.03) | 49.27 (22.66) | 46.14 (24.16) | 43.20 (25.52) | 40.44 (26.74) | 37.85 (27.81) |
| **Female locals(≥60y)** | 77.33 (9.40) | 76.53 (10.11) | 76.22 (10.85) | 75.94 (11.79) | 75.69 (12.94) | 75.49 (14.28) | 75.32 (15.80) | 75.19 (17.48) | 75.10 (19.33) | 75.03 (21.32) |
| **Male immigrants>10(≥60y)** | 293.56 (9.13) | 289.8 (11.7) | 286.6 (15.19) | 284.28 (19.51) | 282.78 (24.49) | 281.99 (30.07) | 281.88 (36.31) | 282.31 (43.15) | 283.37 (50.66) | 285.03 (58.86) |
| **Male immigrants ≤ 10(≥60y)** | 244.88 (30.29) | 247.01 (36.85) | 251.24 (42.94) | 255.62 (50.06) | 260.14 (58.14) | 264.82 (67.14) | 269.61 (77.01) | 274.52 (87.75) | 279.55 (99.34) | 284.69 (111.81) |
| **Male locals(≥60y)** | 150.75 (16.22) | 146.29 (18.46) | 143.54 (20.58) | 141.84 (23.97) | 140.07 (28.24) | 138.14 (33.39) | 136.65 (39.82) | 136.49 (47.87) | 137.24 (57.47) | 138.26 (68.52) |

**eTable 1.** Predictive means and standard deviations (in brackets) of age-standardized mortality rates of lung cancer per 100,000 population for each gender, age group (less than, greater or equal to 60 years old) and immigrant status from 2022 to 2030. Reported means and standard deviations (in brackets) of age-standardized mortality rates in 2021 are also listed.



For peer review only - http://bmjopen.bmj.com/site/about/guidelines.xhtml

| Predictive mean of age-standardized mortality rates of colon cancer per 100,000 population | | | | | | | | | | |
|---|---|---|---|---|---|---|---|---|---|---|
| Year | 2021 | 2022 | 2023 | 2024 | 2025 | 2026 | 2027 | 2028 | 2029 | 2030 |
| Female immigrants >10 | 20.03 (0.95) | 18.95 (1.13) | 18.77 (1.37) | 18.59 (1.66) | 18.42 (1.98) | 18.27 (2.33) | 18.12 (2.71) | 17.98 (3.11) | 17.85 (3.53) | 17.73 (3.96) |
| Female immigrants ≤ 10 | 8.11 (2.19) | 7.70 (2.51) | 7.25 (2.81) | 6.82 (3.11) | 6.42 (3.37) | 6.03 (3.61) | 5.67 (3.83) | 5.33 (4.01) | 5.01 (4.17) | 4.71 (4.31) |
| Female locals | 13.77 (1.30) | 13.47 (1.61) | 13.24 (1.72) | 13.01 (1.87) | 12.77 (2.04) | 12.53 (2.24) | 12.29 (2.46) | 12.06 (2.68) | 11.82 (2.92) | 11.59 (3.16) |
| Male immigrants >10 | 31.22 (1.28) | 29.82 (1.46) | 29.66 (1.79) | 29.52 (2.19) | 29.41 (2.63) | 29.30 (3.11) | 29.21 (3.64) | 29.14 (4.19) | 29.06 (4.78) | 28.98 (5.39) |
| Male immigrants ≤10 | 15.47 (2.14) | 16.77 (3.77) | 17.02 (4.18) | 17.23 (4.64) | 17.45 (5.14) | 17.67 (5.69) | 17.88 (6.27) | 18.09 (6.91) | 18.31 (7.56) | 18.50 (8.26) |
| Male locals | 21.28 (1.38) | 19.81 (2.07) | 19.39 (2.22) | 18.97 (2.42) | 18.57 (2.61) | 18.18 (2.85) | 17.81 (3.12) | 17.43 (3.40) | 17.06 (3.71) | 16.71 (4.03) |
| Female immigrants >10(<60y) | 7.09 (0.99) | 7.36 (1.12) | 7.46 (1.28) | 7.56 (1.46) | 7.65 (1.68) | 7.74 (1.92) | 7.83 (2.19) | 7.92 (2.48) | 8.01 (2.79) | 8.09 (3.13) |
| Female immigrants ≤ 10(<60y) | 3.11 (0.67) | 2.82 (0.86) | 2.65 (0.91) | 2.51 (0.97) | 2.36 (1.02) | 2.22 (1.07) | 2.08 (1.11) | 1.95 (1.14) | 1.83 (1.18) | 1.72 (1.22) |
| Female locals(<60y) | 4.10 (0.41) | 3.87 (0.50) | 3.73 (0.54) | 3.61 (0.59) | 3.47 (0.65) | 3.34 (0.70) | 3.22 (0.76) | 3.11 (0.82) | 2.99 (0.88) | 2.88 (0.94) |
| Male immigrants >10(<60y) | 8.29 (0.91) | 7.98 (1.17) | 7.85 (1.38) | 7.71 (1.60) | 7.54 (1.83) | 7.36 (2.08) | 7.17(2.32) | 6.97(2.57) | 6.76(2.81) | 6.55(3.05) |
| Male immigrants ≤ 10(<60y) | 5.03 (1.44) | 5.18 (1.58) | 5.22 (1.75) | 5.26 (1.93) | 5.30 (2.14) | 5.34 (2.36) | 5.38(2.59) | 5.43(2.84) | 5.47(3.11) | 5.51(3.38) |
| Male locals(<60y) | 5.14 (0.43) | 4.88 (0.63) | 4.66 (0.79) | 4.46 (0.96) | 4.26 (1.13) | 4.08 (1.31) | 3.91(1.48) | 3.73(1.65) | 3.57(1.82) | 3.42(1.97) |
| Female immigrants >10(≥60y) | 52.16 (2.59) | 49.21 (2.99) | 48.70 (3.56) | 48.26 (4.26) | 47.87 (5.05) | 47.54 (5.94) | 47.26 (6.90) | 47.05 (7.94) | 46.91 (9.06) | 46.81 (10.26) |
| Female immigrants ≤ 10(≥60y) | 24.01 (5.83) | 22.44 (6.56) | 21.69 (6.96) | 20.95 (7.38) | 20.23 (7.80) | 19.52 (8.23) | 18.84 (8.66) | 18.17 (9.08) | 17.51 (9.49) | 16.86 (9.90) |
| Female locals(≥60y) | 37.42 (5.31) | 36.69 (5.74) | 36.29 (6.06) | 35.87 (6.46) | 35.46 (6.95) | 35.04 (7.5) | 34.61 (8.12) | 34.19 (8.79) | 33.77 (9.51) | 33.34 (10.27) |
| Male immigrants >10(≥60y) | 84.17 (3.55) | 82.72 (4.09) | 82.16 (4.95) | 81.64 (5.97) | 81.19 (7.12) | 80.81 (8.39) | 80.47 (9.77) | 80.15 (11.24) | 79.85 (12.81) | 79.56 (14.45) |
| Male immigrants ≤ 10(≥60y) | 43.25 (11.07) | 44.93 (13.09) | 45.62 (14.52) | 46.30 (16.09) | 46.96 (17.80) | 47.61 (19.64) | 48.25 (21.62) | 48.88 (23.73) | 49.51 (25.97) | 50.13 (28.34) |
| Male locals(≥60y) | 55.79 (6.86) | 54.89 (7.65) | 53.75 (8.03) | 52.63 (8.52) | 51.54 (9.12) | 50.47 (9.8) | 49.43 (10.55) | 48.42 (11.37) | 47.42 (12.25) | 46.44 (13.16) |

**eTable 2.** Predictive means and standard deviations (in brackets) of age-standardized mortality rates of colon cancer per 100,000 population for each gender, age group (less than, greater or equal to 60 years old) and immigrant status from 2022 to 2030. Reported means and standard deviations (in brackets) of age-standardized mortality rates in 2021 are also listed.



For peer review only - http://bmjopen.bmj.com/site/about/guidelines.xhtml

| Predictive mean of age-standardized mortality rates of liver cancer per 100,000 population | | | | | | | | | | |
|---|---|---|---|---|---|---|---|---|---|---|
| **Year** | **2021** | **2022** | **2023** | **2024** | **2025** | **2026** | **2027** | **2028** | **2029** | **2030** |
| **Female immigrants >10** | 11.34 (0.66) | 10.68 (0.71) | 10.09 (0.85) | 9.54 (1.01) | 9.01 (1.16) | 8.50 (1.31) | 8.02(1.45) | 7.57(1.59) | 7.14(1.72) | 6.74(1.83) |
| **Female immigrants ≤10** | 9.15 (1.55) | 8.66 (1.82) | 8.38 (1.95) | 8.11 (2.08) | 7.84 (2.22) | 7.58 (2.36) | 7.32(2.49) | 7.07(2.63) | 6.82(2.76) | 6.58(2.88) |
| **Female locals** | 6.72 (0.69) | 6.36 (0.88) | 6.08 (0.90) | 5.81 (0.93) | 5.53 (0.97) | 5.26 (1.01) | 5.01(1.06) | 4.77(1.11) | 4.53(1.15) | 4.3(1.21) |
| **Male immigrants >10** | 52.17 (1.78) | 49.22 (2.36) | 47.76 (2.93) | 46.35 (3.59) | 45.01 (4.31) | 43.67 (5.05) | 42.37 (5.81) | 41.1(6.56) | 39.89 (7.33) | 38.71 (8.08) |
| **Male immigrants ≤10** | 42.33 (5.87) | 39.03 (6.49) | 37.39 (7.47) | 35.81 (8.51) | 34.26 (9.58) | 32.76 (10.63) | 31.31 (11.65) | 29.91 (12.62) | 28.56 (13.54) | 27.25 (14.40) |
| **Male locals** | 24.22 (1.77) | 22.16 (2.09) | 21.02 (2.22) | 19.91 (2.39) | 18.85 (2.58) | 17.83 (2.79) | 16.85 (3.03) | 15.92 (3.21) | 15.03 (3.40) | 14.18 (3.59) |
| **Female immigrants >10(<60y)** | 3.62 (0.45) | 3.39 (0.52) | 3.29 (0.57) | 3.20 (0.63) | 3.12 (0.69) | 3.04 (0.75) | 2.96(0.82) | 2.89(0.89) | 2.82(0.96) | 2.75(1.03) |
| **Female immigrants ≤ 10(<60y)** | 4.10 (0.79) | 3.81 (0.91) | 3.69 (0.96) | 3.57 (1.02) | 3.46 (1.08) | 3.36 (1.15) | 3.25(1.22) | 3.15(1.29) | 3.06(1.36) | 2.97(1.43) |
| **Female locals(<60y)** | 1.50 (0.13) | 1.37 (0.2) | 1.29 (0.21) | 1.22 (0.23) | 1.16 (0.24) | 1.10 (0.26) | 1.04(0.27) | 0.99(0.29) | 0.94(0.30) | 0.89(0.31) |
| **Male immigrants >10(<60y)** | 26.32 (2.11) | 24.04 (2.35) | 23.02 (2.63) | 22.05 (2.94) | 21.13 (3.27) | 20.25 (3.61) | 19.41 (3.95) | 18.62 (4.30) | 17.86 (4.64) | 17.14 (4.98) |
| **Male immigrants ≤ 10(<60y)** | 25.52 (2.99) | 22.56 (3.96) | 21.71 (4.44) | 20.87 (4.94) | 20.04 (5.45) | 19.22 (5.95) | 18.42 (6.45) | 17.63 (6.91) | 16.86 (7.36) | 16.11 (7.78) |
| **Male locals(<60y)** | 8.25 (0.69) | 7.47 (0.74) | 6.97 (0.79) | 6.52 (0.86) | 6.11 (0.93) | 5.73 (1.01) | 5.38(1.08) | 5.04(1.15) | 4.73(1.21) | 4.44(1.27) |
| **Female immigrants >10(≥ 60y)** | 33.67 (1.88) | 29.63 (2.01) | 27.99 (2.36) | 26.42 (2.75) | 24.92 (3.14) | 23.49 (3.52) | 22.13 (3.88) | 20.85 (4.23) | 19.64 (4.55) | 18.50 (4.85) |
| **Female immigrants ≤10(≥ 60y)** | 21.72 (5.11) | 19.08 (5.81) | 18.38 (6.14) | 17.71 (6.48) | 17.03 (6.83) | 16.39 (7.16) | 15.76 (7.49) | 15.16 (7.80) | 14.57 (8.11) | 14.01 (8.39) |
| **Female locals(≥60y)** | 20.63 (3.03) | 18.41 (3.23) | 17.55 (3.26) | 16.72 (3.32) | 15.91 (3.40) | 15.11 (3.49) | 14.34 (3.59) | 13.59 (3.69) | 12.87 (3.81) | 12.17 (3.93) |
| **Male immigrants >10(≥ 60y)** | 115.39 (4.54) | 113.96 (5.95) | 113.43 (7.65) | 113.17 (9.70) | 113.16 (12.04) | 113.37 (14.66) | 113.79 (17.56) | 114.39 (20.73) | 115.19 (24.18) | 116.17 (27.91) |
| **Male immigrants ≤10(≥ 60y)** | 88.61 (15.58) | 85.14 (18.85) | 82.59 (20.6) | 80.02 (22.44) | 77.42 (24.34) | 74.83 (26.24) | 72.23 (28.12) | 69.64 (29.94) | 67.07 (31.70) | 64.52 (33.38) |
| **Male locals(≥60y)** | 62.88 (5.97) | 58.95 (7.91) | 56.51 (8.20) | 54.14 (8.61) | 51.84 (9.12) | 49.61 (9.70) | 47.46 (10.33) | 45.38 (11.01) | 43.38 (11.68) | 41.45 (12.36) |

**eTable 3.** Predictive means and standard deviations (in brackets) of age-standardized mortality rates of liver cancer per 100,000 population for each gender, age group (less than, greater or equal to 60 years old) and immigrant status from 2022 to 2030. Reported means and standard deviations (in brackets) of age-standardized mortality rates in 2021 are also listed.

For peer review only



For peer review only - http://bmjopen.bmj.com/site/about/guidelines.xhtml

| Predictive mean of age-standardized mortality rates of pancreatic cancer per 100,000 population | | | | | | | | | | |
|---|---|---|---|---|---|---|---|---|---|---|
| Year | 2021 | 2022 | 2023 | 2024 | 2025 | 2026 | 2027 | 2028 | 2029 | 2030 |
| Female immigrants >10 | 10.89 (0.62) | 11.11 (0.75) | 11.36 (0.91) | 11.61 (1.09) | 11.87 (1.31) | 12.14 (1.56) | 12.42 (1.84) | 12.71 (2.15) | 13.01 (2.48) | 13.3(2.85) |
| Female immigrants ≤ 10 | 5.51 (1.44) | 5.44 (1.56) | 5.44 (1.69) | 5.43(1.84) | 5.42(1.99) | 5.41(2.15) | 5.39(2.32) | 5.36(2.49) | 5.34(2.66) | 5.31(2.84) |
| Female locals | 8.79 (1.10) | 9.01 (1.22) | 9.15 (1.34) | 9.29(1.48) | 9.43(1.64) | 9.57(1.83) | 9.71(2.05) | 9.85(2.28) | 9.99(2.54) | 10.14 (2.83) |
| Male immigrants >10 | 16.30 (0.98) | 17.87 (1.19) | 18.48 (1.49) | 19.11 (1.87) | 19.78 (2.32) | 20.47 (2.83) | 21.18 (3.42) | 21.92 (4.07) | 22.69 (4.81) | 23.49 (5.61) |
| Male immigrants ≤10 | 8.10 (2.02) | 7.87 (2.37) | 7.76 (2.53) | 7.64(2.70) | 7.51(2.87) | 7.38(3.05) | 7.24(3.23) | 7.09(3.41) | 6.95(3.58) | 6.81(3.75) |
| Male locals | 11.97 (1.26) | 12.29 (1.49) | 12.49 (1.64) | 12.69 (1.83) | 12.91 (2.06) | 13.11 (2.33) | 13.33 (2.63) | 13.55 (2.97) | 13.78 (3.34) | 14.02 (3.74) |
| Female immigrants >10(<60y) | 3.47 (0.33) | 3.62 (0.57) | 3.74 (0.66) | 3.87(0.77) | 4.01(0.89) | 4.14(1.02) | 4.28(1.18) | 4.42(1.34) | 4.57(1.53) | 4.72(1.73) |
| Female immigrants ≤ 10(<60y) | 1.12 (0.33) | 1.21 (0.48) | 1.22 (0.52) | 1.23(0.56) | 1.24(0.61) | 1.25(0.66) | 1.26(0.71) | 1.26(0.77) | 1.27(0.83) | 1.28(0.89) |
| Female locals(<60y) | 2.76 (0.27) | 2.88 (0.36) | 2.91 (0.41) | 2.93(0.48) | 2.96(0.55) | 2.99(0.63) | 3.02(0.71) | 3.04(0.81) | 3.07(0.90) | 3.10(1.01) |
| Male immigrants >10(<60y) | 6.88 (0.98) | 7.05 (1.11) | 7.24 (1.32) | 7.43(1.56) | 7.62(1.84) | 7.82(2.16) | 8.01(2.50) | 8.21(2.88) | 8.40(3.30) | 8.61(3.75) |
| Male immigrants ≤ 10(<60y) | 2.20 (0.71) | 2.01 (0.85) | 1.95 (0.91) | 1.9(0.94) | 1.84(0.99) | 1.79(1.04) | 1.74(1.09) | 1.69(1.14) | 1.64(1.19) | 1.60(1.24) |
| Male locals(<60y) | 4.16 (0.35) | 4.33 (0.48) | 4.41 (0.57) | 4.46(0.68) | 4.53(0.81) | 4.61(0.94) | 4.69(1.09) | 4.77(1.26) | 4.85(1.44) | 4.93(1.63) |
| Female immigrants >10(≥60y) | 28.58 (1.83) | 29.45 (2.11) | 29.91 (2.54) | 30.38 (3.06) | 30.85 (3.66) | 31.33 (4.33) | 31.81 (5.08) | 32.29 (5.91) | 32.78 (6.79) | 33.27 (7.74) |
| Female immigrants ≤ 10(≥60y) | 16.79 (5.29) | 15.65 (6.08) | 15.49 (6.71) | 15.33 (7.36) | 15.16 (8.03) | 14.97 (8.73) | 14.79 (9.43) | 14.59 (10.14) | 14.39 (10.86) | 14.19 (11.58) |
| Female locals(≥60y) | 22.80 (4.23) | 23.85 (4.46) | 24.21 (4.81) | 24.56 (5.23) | 24.91 (5.73) | 25.25 (6.30) | 25.58 (6.95) | 25.90 (7.67) | 26.22 (8.47) | 26.54 (9.34) |
| Male immigrants >10(≥60y) | 42.70 (2.55) | 44.36 (3.02) | 45.85 (3.76) | 47.41 (4.69) | 49.04 (5.78) | 50.73 (7.05) | 52.48 (8.50) | 54.28 (10.13) | 56.16 (11.95) | 58.11 (13.98) |
| Male immigrants ≤ 10(≥60y) | 24.68 (8.21) | 23.96 (9.01) | 23.87 (9.74) | 23.75 (10.52) | 23.61 (11.33) | 23.45 (12.17) | 23.28 (13.04) | 23.09 (13.93) | 22.89 (14.83) | 22.68 (15.75) |
| Male locals(≥60y) | 30.10 (4.68) | 31.17 (5.22) | 31.55 (5.63) | 31.93 (6.14) | 32.30 (6.75) | 32.66 (7.45) | 33.01 (8.23) | 33.35 (9.11) | 33.69 (10.08) | 34.03 (11.12) |

**eTable 4.** Predictive means and standard deviations (in brackets) of age-standardized mortality rates of pancreatic cancer per 100,000 population for each gender, age group (less than, greater or equal to 60 years old) and immigrant status from 2022 to 2030. Reported means and standard deviations (in brackets) of age-standardized mortality rates in 2021 are also listed.



For peer review only - http://bmjopen.bmj.com/site/about/guidelines.xhtml

| Predictive mean of age-standardized mortality rates of stomach cancer per 100,000 population | | | | | | | | | | |
|---|---|---|---|---|---|---|---|---|---|---|
| Year | 2021 | 2022 | 2023 | 2024 | 2025 | 2026 | 2027 | 2028 | 2029 | 2030 |
| Female immigrants >10 | 8.20 (0.55) | 7.95 (0.62) | 7.71 (0.74) | 7.47 (0.87) | 7.25 (1.01) | 7.03 (1.15) | 6.83 (1.29) | 6.62 (1.43) | 6.43 (1.57) | 6.24 (1.71) |
| Female immigrants ≤ 10 | 7.51 (1.44) | 7.36 (1.56) | 7.33 (1.69) | 7.30 (1.85) | 7.28 (2.01) | 7.27 (2.20) | 7.27 (2.40) | 7.28 (2.61) | 7.31 (2.84) | 7.33 (3.09) |
| Female locals | 5.26 (0.40) | 4.91 (0.52) | 4.75 (0.57) | 4.61 (0.63) | 4.47 (0.71) | 4.34 (0.77) | 4.21 (0.84) | 4.08 (0.91) | 3.95 (0.99) | 3.83 (1.06) |
| Male immigrants >10 | 15.22 (0.64) | 13.89 (0.97) | 13.34(1.21) | 12.81 (1.46) | 12.31 (1.73) | 11.83 (1.99) | 11.38 (2.26) | 10.95 (2.51) | 10.54 (2.76) | 10.15 (3.01) |
| Male immigrants ≤10 | 15.83 (3.04) | 15.21 (3.38) | 15.07 (3.67) | 14.93 (3.98) | 14.79 (4.31) | 14.64 (4.65) | 14.51 (5.02) | 14.35 (5.39) | 14.19 (5.78) | 14.03 (6.17) |
| Male locals | 8.14 (0.89) | 8.07 (0.99) | 7.73 (1.03) | 7.41(1.07) | 7.10 (1.13) | 6.81 (1.19) | 6.51 (1.26) | 6.23 (1.33) | 5.97 (1.39) | 5.71 (1.46) |
| Female immigrants >10(<60y) | 4.81 (0.56) | 4.69 (0.79) | 4.62 (0.87) | 4.55 (0.96) | 4.47 (1.07) | 4.39 (1.17) | 4.31 (1.29) | 4.22 (1.41) | 4.13 (1.52) | 4.03 (1.64) |
| Female immigrants ≤ 10(<60y) | 3.89 (0.80) | 4.08 (0.93) | 4.10 (1.03) | 4.13 (1.14) | 4.17 (1.27) | 4.21 (1.41) | 4.24 (1.55) | 4.28 (1.70) | 4.32 (1.87) | 4.36 (2.05) |
| Female locals(<60y) | 2.28 (0.21) | 2.08 (0.27) | 1.98 (0.29) | 1.88 (0.32) | 1.79 (0.35) | 1.71 (0.37) | 1.61 (0.41) | 1.53 (0.43) | 1.44 (0.45) | 1.37 (0.47) |
| Male immigrants >10(<60y) | 4.94 (0.57) | 4.71 (0.79) | 4.55 (0.89) | 4.41 (0.99) | 4.25 (1.10) | 4.12 (1.21) | 3.98 (1.32) | 3.86 (1.43) | 3.74 (1.54) | 3.63 (1.65) |
| Male immigrants ≤ 10(<60y) | 4.81 (1.31) | 4.70 (1.42) | 4.66 (1.55) | 4.63 (1.69) | 4.59 (1.83) | 4.55 (1.99) | 4.52 (2.15) | 4.48 (2.32) | 4.44 (2.50) | 4.41 (2.68) |
| Male locals(<60y) | 2.48 (0.21) | 2.37 (0.29) | 2.28 (0.32) | 2.21 (0.35) | 2.12 (0.38) | 2.04 (0.42) | 1.97 (0.45) | 1.91 (0.49) | 1.83 (0.52) | 1.77(0.55) |
| Female immigrants >10(≥60y) | 17.80 (1.04) | 16.23 (1.26) | 15.65 (1.47) | 15.08 (1.70) | 14.55 (1.94) | 14.03 (2.18) | 13.54 (2.43) | 13.07 (2.68) | 12.62 (2.92) | 12.19 (3.16) |
| Female immigrants ≤ 10(≥60y) | 14.72 (4.29) | 13.01 (4.83) | 12.52 (5.11) | 12.03 (5.37) | 11.55 (5.63) | 11.08 (5.88) | 10.63 (6.12) | 10.19 (6.35) | 9.76(6.56) | 9.34 (6.75) |
| Female locals(≥60y) | 12.20 (1.66) | 11.86 (1.84) | 11.67 (1.98) | 11.49 (2.15) | 11.33 (2.35) | 11.18 (2.58) | 11.04 (2.84) | 10.91 (3.11) | 10.79(3.4) | 10.68 (3.71) |
| Male immigrants >10(≥60y) | 37.23 (2.29) | 36.59 (2.56) | 35.17(3.18) | 33.82 (3.86) | 32.55 (4.57) | 31.34 (5.28) | 30.19 (6.01) | 29.08 (6.70) | 28.02 (7.40) | 27.01 (8.07) |
| Male immigrants ≤ 10(≥60y) | 42.30 (10.88) | 41.43 (11.78) | 41.03 (12.71) | 40.61 (13.70) | 40.17 (14.75) | 39.71 (15.85) | 39.24 (16.99) | 38.75 (18.16) | 38.23 (19.35) | 37.71 (20.57) |
| Male locals(≥60y) | 23.04 (3.29) | 22.69 (3.56) | 22.37(4.07) | 22.16(4.84) | 21.89 (5.86) | 21.61 (7.22) | 21.52 (9.02) | 21.74 (11.29) | 22.17 (14.03) | 22.73 (17.28) |

**eTable 5.** Predictive means and standard deviations (in brackets) of age-standardized mortality rates of stomach cancer per 100,000 population for each gender, age group (less than, greater or equal to 60 years old) and immigrant status from 2022 to 2030. Reported means and standard deviations (in brackets) of age-standardized mortality rates in 2021 are also listed.



For peer review only - http://bmjopen.bmj.com/site/about/guidelines.xhtml

| Predictive mean of age-standardized mortality rates of prostate cancer per 100,000 population | | | | | | | | | | |
|---|---|---|---|---|---|---|---|---|---|---|
| Year | 2021 | 2022 | 2023 | 2024 | 2025 | 2026 | 2027 | 2028 | 2029 | 2030 |
| Male immigrants >10 | 14.81 (0.61) | 14.59 (0.79) | 14.57 (0.96) | 14.56 (1.15) | 14.54 (1.37) | 14.51 (1.61) | 14.48 (1.86) | 14.45 (2.13) | 14.42 (2.42) | 14.38 (2.72) |
| Male immigrants ≤10 | 9.03 (2.95) | 8.78 (3.11) | 8.58 (3.29) | 8.39 (3.49) | 8.19(3.69) | 8.10(3.89) | 7.82(4.11) | 7.63(4.31) | 7.45(4.51) | 7.27(4.72) |
| Male locals | 9.54 (1.40) | 9.66 (1.57) | 9.67 (1.66) | 9.69 (1.77) | 9.72(1.91) | 9.75(2.06) | 9.78(2.23) | 9.82(2.43) | 9.86(2.64) | 9.9(2.88) |
| Male immigrants >10(<60y) | 0.57 (0.12) | 0.52 (0.17) | 0.51 (0.19) | 0.50 (0.21) | 0.49(0.22) | 0.48(0.24) | 0.47(0.25) | 0.46(0.27) | 0.45(0.29) | 0.44(0.31) |
| Male immigrants ≤ 10(<60y) | 0.65 (0.59) | 0.73 (0.77) | 0.81 (0.93) | 0.87 (1.10) | 0.94(1.31) | 1.01(1.51) | 1.09(1.75) | 1.16(2.02) | 1.24(2.32) | 1.33(2.64) |
| Male locals(<60y) | 0.63 (0.12) | 0.66 (0.14) | 0.66 (0.16) | 0.66 (0.19) | 0.67(0.21) | 0.67(0.24) | 0.67(0.27) | 0.68(0.31) | 0.68(0.33) | 0.69(0.37) |
| Male immigrants >10(≥ 60y) | 49.43 (2.59) | 49.61 (2.73) | 49.63 (3.29) | 49.64 (3.94) | 49.64 (4.68) | 49.64 (5.51) | 49.63 (6.38) | 49.62 (7.32) | 49.61 (8.32) | 49.58(9.3 7) |
| Male immigrants ≤ 10(≥60y) | 28.29 (9.15) | 27.66 (9.78) | 26.53 (10.21) | 25.4 (10.63) | 24.28 (11.03) | 23.16 (11.41) | 22.07 (11.76) | 21.01 (12.09) | 19.96 (12.38) | 18.95(12. 63) |
| Male locals(≥60y) | 31.57 (5.17) | 31.48 (5.49) | 31.40 (5.76) | 31.32 (6.09) | 31.24 (6.48) | 31.15 (6.94) | 31.06 (7.44) | 30.96 (8.01) | 30.86 (8.61) | 30.74(9.2 6) |

**eTable 6.** Predictive means and standard deviations (in brackets) of age-standardized mortality rates of prostate cancer per 100,000 population for each age group (less than, greater or equal to 60 years old) and immigrant status from 2022 to 2030. Reported means and standard deviations (in brackets) of age-standardized mortality rates in 2021 are also listed.

For peer review only



For peer review only - http://bmjopen.bmj.com/site/about/guidelines.xhtml

# Reporting checklist for cohort study.

Based on the STROBE cohort guidelines.

## Instructions to authors

Complete this checklist by entering the page numbers from your manuscript where readers will find each of the items listed below.

Your article may not currently address all the items on the checklist. Please modify your text to include the missing information. If you are certain that an item does not apply, please write "n/a" and provide a short explanation.

Upload your completed checklist as an extra file when you submit to a journal.

In your methods section, say that you used the STROBE cohortreporting guidelines, and cite them as:

von Elm E, Altman DG, Egger M, Pocock SJ, Gotzsche PC, Vandenbroucke JP. The Strengthening the Reporting of Observational Studies in Epidemiology (STROBE) Statement: guidelines for reporting observational studies.

| | | Reporting Item | Page Number |
|---|---|---|---|
| **Title and abstract** | | | |
| Title | #1a | Indicate the study’s design with a commonly used term in the title or the abstract | 1 |
| Abstract | #1b | Provide in the abstract an informative and balanced summary of what was done and what was found | 2 |
| **Introduction** | | | |
| Background / rationale | #2 | Explain the scientific background and rationale for the investigation being reported | 4 |
| Objectives | #3 | State specific objectives, including any prespecified hypotheses | 4 |
| **Methods** | | | |
| Study design | #4 | Present key elements of study design early in the paper | 5 |
| Setting | #5 | Describe the setting, locations, and relevant dates, including periods | 5 |

For peer review only

| | | | |
|---|---|---|---|
| | | of recruitment, exposure, follow-up, and data collection | |
| Eligibility criteria | #6a | Give the eligibility criteria, and the sources and methods of selection of participants. Describe methods of follow-up. | 5 |
| Eligibility criteria | #6b | For matched studies, give matching criteria and number of exposed and unexposed | n/a |
| Variables | #7 | Clearly define all outcomes, exposures, predictors, potential confounders, and effect modifiers. Give diagnostic criteria, if applicable | 5 |
| Data sources / measurement | #8 | For each variable of interest give sources of data and details of methods of assessment (measurement). Describe comparability of assessment methods if there is more than one group. Give information separately for for exposed and unexposed groups if applicable. | 5 |
| Bias | #9 | Describe any efforts to address potential sources of bias | 5 |
| Study size | #10 | Explain how the study size was arrived at | 5 |
| Quantitative variables | #11 | Explain how quantitative variables were handled in the analyses. If applicable, describe which groupings were chosen, and why | 5 |
| Statistical methods | #12a | Describe all statistical methods, including those used to control for confounding | |
| 5 | | | |
| Statistical methods | #12b | Describe any methods used to examine subgroups and interactions | 5 |
| Statistical methods | #12c | Explain how missing data were addressed | 5 |
| Statistical methods | #12d | If applicable, explain how loss to follow-up was addressed | n/a |
| Statistical methods | #12e | Describe any sensitivity analyses | |
| n/a | | | |
| **Results** | | | |
| Participants | #13a | Report numbers of individuals at each stage of study—eg numbers potentially eligible, examined for eligibility, confirmed eligible, | n/a |

For peer review only

| | | | |
|---|---|---|---|
| | | included in the study, completing follow-up, and analysed. Give information separately for for exposed and unexposed groups if applicable. | |
| Participants | #13b | Give reasons for non-participation at each stage | 5 |
| Participants | #13c | Consider use of a flow diagram | |
| n/a | | | |
| Descriptive data | #14a | Give characteristics of study participants (eg demographic, clinical, social) and information on exposures and potential confounders. Give information separately for exposed and unexposed groups if applicable. | 5 |
| Descriptive data | #14b | Indicate number of participants with missing data for each variable of interest | |
| n/a | | | |
| Descriptive data | #14c | Summarise follow-up time (eg, average and total amount) | |
| n/a | | | |
| Outcome data | #15 | Report numbers of outcome events or summary measures over time. Give information separately for exposed and unexposed groups if applicable. | |
| n/a | | | |
| Main results | #16a | Give unadjusted estimates and, if applicable, confounder-adjusted estimates and their precision (eg, 95% confidence interval). Make clear which confounders were adjusted for and why they were included | 6 |
| Main results | #16b | Report category boundaries when continuous variables were categorized | n/a |
| Main results | #16c | If relevant, consider translating estimates of relative risk into absolute risk for a meaningful time period | |
| n/a | | | |
| Other analyses | #17 | Report other analyses done—eg analyses of subgroups and interactions, and sensitivity analyses | 7 |

**Discussion**

For peer review only

| | | | |
|---|---|---|---|
| Key results | #18 | Summarise key results with reference to study objectives | 8 |
| Limitations | #19 | Discuss limitations of the study, taking into account sources of potential bias or imprecision. Discuss both direction and magnitude of any potential bias. | 10 |
| Interpretation | #20 | Give a cautious overall interpretation considering objectives, limitations, multiplicity of analyses, results from similar studies, and other relevant evidence. | 8 |
| Generalisability | #21 | Discuss the generalisability (external validity) of the study results | 9 |
| **Other Information** | | | |
| Funding | #22 | Give the source of funding and the role of the funders for the present study and, if applicable, for the original study on which the present article is based | 11 |

The STROBE checklist is distributed under the terms of the Creative Commons Attribution License CC-BY.
This checklist was completed on 11. February 2023 using https://www.goodreports.org/, a tool made by the EQUATOR Network in collaboration with Penelope.ai

For peer review only

BMJ Open

# An age-period-cohort analysis and projection of cancer mortality in Hong Kong, 1998–2030



| | |
|---|---|
| Journal: | *BMJ Open* |
| Manuscript ID | bmjopen-2023-072751.R4 |
| Article Type: | Original research |
| Date Submitted by the Author: | 05-Sep-2023 |
| Complete List of Authors: | Zhao, Yanji; The Hong Kong Polytechnic University, Department of Applied Mathematics<br>Zhuang, Zian; The Hong Kong Polytechnic University, Department of Applied Mathematics; University of California Los Angeles, Department of Biostatistics<br>Yang, Lin; The Hong Kong Polytechnic University, School of Nursing<br>He, Daihai; The Hong Kong Polytechnic University, Department of Applied Mathematics; The Hong Kong Polytechnic University, Research Institute for Future Food |
| <b>Primary Subject Heading</b>: | Research methods |
| Secondary Subject Heading: | Public health, Oncology |
| Keywords: | STATISTICS & RESEARCH METHODS, ONCOLOGY, Risk Factors |
| | |



SCHOLARONE™
Manuscripts

*I, the Submitting Author has the right to grant and does grant on behalf of all authors of the Work (as defined in the below author licence), an exclusive licence and/or a non-exclusive licence for contributions from authors who are: i) UK Crown employees; ii) where BMJ has agreed a CC-BY licence shall apply, and/or iii) in accordance with the terms applicable for US Federal Government officers or employees acting as part of their official duties; on a worldwide, perpetual, irrevocable, royalty-free basis to BMJ Publishing Group Ltd ("BMJ") its licensees and where the relevant Journal is co-owned by BMJ to the co-owners of the Journal, to publish the Work in this journal and any other BMJ products and to exploit all rights, as set out in our licence.*

*The Submitting Author accepts and understands that any supply made under these terms is made by BMJ to the Submitting Author unless you are acting as an employee on behalf of your employer or a postgraduate student of an affiliated institution which is paying any applicable article publishing charge ("APC") for Open Access articles. Where the Submitting Author wishes to make the Work available on an Open Access basis (and intends to pay the relevant APC), the terms of reuse of such Open Access shall be governed by a Creative Commons licence – details of these licences and which Creative Commons licence will apply to this Work are set out in our licence referred to above.*

*Other than as permitted in any relevant BMJ Author's Self Archiving Policies, I confirm this Work has not been accepted for publication elsewhere, is not being considered for publication elsewhere and does not duplicate material already published. I confirm all authors consent to publication of this Work and authorise the granting of this licence.*

For peer review only

# An age-period-cohort analysis and projection of cancer mortality in Hong Kong, 1998–2030

Yanji Zhao[1, #], Zian Zhuang[1,2, #], Lin Yang[3] and Daihai He [1,4*]

1 Department of Applied Mathematics, Hong Kong Polytechnic University, Hong Kong, China

2 Department of Biostatistics, University of California, Los Angeles, CA, United States

3 School of Nursing, Hong Kong Polytechnic University, Hong Kong, China

4 Research Institute for Future Food, Hong Kong Polytechnic University, Hong Kong, China

# These authors contributed equally to this work.

*Correspondence to:

Daihai He

Department of Applied Mathematics, The Hong Kong Polytechnic University, Hong Kong, China

Tel: 852-27667864
Email: daihai.he@polyu.edu.hk

*Word count:3326*



For peer review only - http://bmjopen.bmj.com/site/about/guidelines.xhtml

## Abstract

**Objectives:** To explore the relationship between immigration groups and cancer mortality, this study aimed to explore age, period, birth cohort effects and effects across genders and immigration groups on mortality rates of lung, pancreatic, colon, liver, prostate and stomach cancers and their projections.

**Design, setting, and participants:** Death registry data in Hong Kong between 1998 and 2021, which were stratified by age, sex and immigration status. Immigration status was classified into three groups: locals born in Hong Kong, long-stay immigrants and short-stay immigrants.

**Methods:** Age-period-cohort analysis was used to examine age, period, and birth cohort effects for genders and immigration groups from 1998 to 2021. Bayesian age-period-cohort models were applied to predict the mortality rates from 2022 to 2030.

**Results:** Short-stay immigrants revealed pronounced fluctuations of mortality rates by age and of relative risks by cohort and period effects for six types of cancers than those of long-stay immigrants and locals. Immigrants for each type of cancer and gender will be at a higher mortality risk than locals. After 2021, decreasing trends ($p<0.05$) or plateau ($p>0.05$) of forecasting mortality rates of cancers occur for all immigration groups, except for increasing trends for short-stay male immigrants with colon cancer ($p<0.05$, Avg +0.30 deaths/100,000 per annum from 15.47 to 18.50 deaths/100,000) and long-stay male immigrants with pancreatic cancer ($p<0.05$, Avg +0.72 deaths/100,000 per annum from 16.30 to 23.49 deaths/100,000).,

**Conclusions:** Findings underscore the effect of gender and immigration status in Hong Kong on mortality risks of cancers that immigrants for each type of cancer and gender will be at a higher mortality risk than locals.


***Keywords***: Age-period-cohort analysis, immigration, mortality, lung cancer, pancreatic cancer, colon cancer, liver cancer, prostate cancer, stomach cancer



For peer review only - http://bmjopen.bmj.com/site/about/guidelines.xhtml

## Strengths and limitations of this study

- This study provides new evidence regarding the relationship between immigration status and cancer mortality, given the effects of age, period, birth cohort and their predictions.

- The non-identifiability problem has not been interpreted in age-period-cohort models.

- The future perspective of cancer therapies and techniques have not been considered.

For peer review only



For peer review only - http://bmjopen.bmj.com/site/about/guidelines.xhtml

## Introduction

Several migration waves from mainland China to Hong Kong have occurred over the past century. These migration waves included a large-scale migration inflow from 1945 to 1950 (the Chinese Civil War) and a few small-scale inflows in the 1950s, 1970s, and 1990s [1-3]. In 2016, immigrants from mainland China formed approximately 38% of the population of Hong Kong. These inflows have led to a growing interest in research on the disparity of health conditions between the locals and immigrants.

Cancer has been one of the most common causes of death, as an estimated 19.3 million new cancer cases and 9.9 million new cancer-associated deaths occurred worldwide in 2020 [4]. In Hong Kong, lung cancer is one of the most common causes of cancer deaths [5, 6]. Previous studies suggested that the primary cause of lung cancer is cigarette smoking [7-11]. Genetic factors, asbestos, radon gas, second-hand smoke, and other forms of air pollution have been proven to influence the risk of lung cancer [12-18]. The overall daily smoking rate in mainland China was approximately 23.2% in 2018 [19], whereas the daily smoking rate in Hong Kong was only 10.2% in 2019 [20]. The leading causes of liver cancer include viral infection, drinking of alcohol and polluted water and food supplies which are also culprits for colon, stomach and pancreatic cancer [21]. Alcohol consumption per capita in Hong Kong has reached 2.37 liters in 2021 [22], compared to 7.0 liters of per capita consumption of alcohol in mainland China in 2018 [23]. As approximately 99% of prostate cancer cases occur after age 50, factors of prostate cancer have been regarded as old age, race, family history and the diet of red meat consumption [24]. In addition to these risk factors, studies have suggested that cancer mortality rates vary depending on migrant status [25-28]. According to data from the Census and Statistics Department of Hong Kong, approximately 81% of immigrants in Hong Kong immigrated from mainland China, Macau, and Taiwan. Immigrants from mainland China account for the bulk of this population. Previous studies have shown that child immigrants in Hong Kong tend to suffer from a higher risk of wheezing disorders and cardiovascular diseases, and immigrant women have higher age-specific mortality rates of breast cancer than locally-born women in Hong Kong [29, 30]. However, to date, few studies have investigated the effect of length of stay in Hong Kong and birthplace on the risk of other types of cancer.

In this study, we compared the mortality rates of lung, pancreatic, colon, liver, prostate and stomach cancers between locally born residents in Hong Kong and immigrants from mainland China. Both populations are widely considered as ethnically homogeneous with similar cultures. Nevertheless, due to different early life experiences, immigrants are exposed to more various social economy and lifestyles than locals. Therefore, it's constructive to ascertain whether immigrants from mainland China have a different mortality pattern of cancers from locals to verify the significance of migration status for this health outcome. As age-period-cohort (APC)



For peer review only - http://bmjopen.bmj.com/site/about/guidelines.xhtml

analysis plays a vital role in studying time-specific phenomena in epidemiology, in this study, to evaluate the effect of immigration on cancer mortality in the past and future, we developed APC models specified by sex and migrant status to assess the effects of age, period, birth cohort, and of the length of stay in Hong Kong on the mortality risks of cancers. Additionally, we explore the projection of mortality rates for the locally born population and immigrants in Hong Kong who were younger or older than 60 using a predictive model, taking into account age, period, and birth cohort effects as well.

## Methods

*Data*

We obtained the death registry data in Hong Kong between 1998 and 2021 from the Census and Statistics Department of Hong Kong, as the data in 2022 has not been available up to now. The data was extracted from a routine census held by the Hong Kong government as subjective errors caused by resampling can be neglected. The population data were stratified by age, sex, immigration status, and length of stay in Hong Kong. We retrieved six types of cancer cases from the death registry data using ICD codes, such as ICD-9 code 162 and ICD-10 codes C34.0–C34.3, C348, and C349 for lung cancer. To assure comparability among registries, deaths from the age group of 35–85 years were selected, since cases younger than 35 and older than 85 were relatively trivial for lack of statistical interpretability [31]. Immigration status was classified into three groups: locals born in Hong Kong, immigrants who have lived in Hong Kong for >10 years before death defined as long-stay immigrants, and immigrants who have lived in Hong Kong for ≤10 years before death defined as short-stay immigrants. Notably, much focus was placed on immigrants from mainland China, because approximately 81% of immigrants in Hong Kong came from mainland China, Macau, and Taiwan based on the data from the Census and Statistics Department of Hong Kong. Moreover, few cases recorded from Macau and Taiwan are statistically insignificant in the analysis. Demographics and population projections from 2022 to 2030 were retrieved from the Census and Statistics Department of Hong Kong and estimated with cubic smoothing spline as the prerequisite of the predictive model. Codes for APC and BAPC analysis are available in the GitHub repository (https://github.com/kshz2164313/APC-population-projections-for-immigration-HK).

*Statistical analysis*

We modeled cancer mortality rates in Hong Kong using APC analysis based on log-linear Poisson regression models. The model aimed to disentangle age, period, and cohort effects of time-varying phenomena simultaneously [32, 33], given that

$$\log(E_{ij}) = \alpha_i + \beta_j + \gamma_k + \mu + \log(\theta_{ij}) \quad (1)$$



For peer review only - http://bmjopen.bmj.com/site/about/guidelines.xhtml

where $E_{ij}$ denotes expected mortality; $\alpha_i$, $\beta_j$, and $\gamma_k$ denote age, period, and cohort effect, respectively, for $i=1,...,I$, $j=1,...,J$, $k=1,...,K$ with $k=I-i+j$. $\log(\theta_{ij})$ is the offset. We mainly focused on the contributions of sex and immigration status due to the non-identifiability problem that the effects of these three components are collinear with each other (denoted as period – age = cohort) [34]. Birth cohort effect and period effect were assessed with relative risks to evaluate the effect of three components. The median year of birth among cases was regarded as the reference cohort [35,36]. Since death cases aged at 35–85 years between 1998 and 2021 were selected, the range of birth cohort from 1913 to 1986 covered observations and further projections until 2030. The second and penultimate period effects were constrained to the reference for period. For sex and immigration status, maximum likelihood framework was applied to estimate the relative risks and 95% confidence intervals (CIs) by age groups, calendar period, and birth cohort.

Several projection approaches for future cancer mortality have been developed, but a Bayesian age-period-cohort (BAPC) model built upon integrated nested Laplace approximations (INLA) [37] yields relatively higher coverage and better performance for all evaluated parameter combinations [38]. To prevent some sampling problems caused by Markov chain Monte Carlo (MCMC), this MCMC-free BAPC approach was applied to predict future cancer mortality within a fully Bayesian inference setting and provide outputs of interest simply, such as projected age-standardized and age-specific rates. Convergence checks are not necessary for this technique [37]. The projections of age-standardized cancer mortality rates for each sex, age group (younger or older than 60 years) and migrant status, taking into account age, period, and birth cohort effects, were performed based on the weights of population age groups from the WHO World Standard population [39], with 95% prediction intervals. Mann-Kendall trend test was applied to verify the projection trend. Friedman's Two-Way Analysis of Variance was applied to test interactions between gender and immigration groups for each year.

All analyses were performed via R version 4.2.1 (R Core Team, R Foundation for Statistical Computing, Vienna, Austria, 2013, http://www.R-project.org/). The APC models were established using the Epi package, and the projections based on Bayesian APC models were performed with the BAPC package.

*Patient and public involvement*

None.

## Results

Figure 1 & 2 and eFigure 1(a-e) in **Supplementary Material** illustrate the estimates of age (assessed by cancer mortality), cohort and period effects (assessed by relative risk) based on APC models among three migrant groups for men and women with six types of cancers,



For peer review only - http://bmjopen.bmj.com/site/about/guidelines.xhtml

respectively. All the mortality rates for each gender and immigration status exhibit notable increasing trends with age. Age, cohort and period effects of six types of cancer for immigrants who stayed in Hong Kong for ≤10 years revealed relatively more pronounced fluctuations and deviations from those effects in the other two immigration groups. Significant increasing trends of age effect occurred in all types of cancer, regardless of gender and immigration status. For example, while relatively insignificant differences in lung cancer mortality rates by immigration status among females have performed, male immigrants who remained in Hong Kong for >10 years had higher lung cancer mortality rates at ages above 50 years and those who arrived ≤10 years had lower lung cancer mortality at ages below 62 years compared to local men Figure 1. In addition to compatible dynamics of period effect for locals and long-stay immigrants, similar changes of relative risks by birth cohort for locals and long-stay immigrants in lung, colon, liver and stomach cancers occurred before 1945, whereas significant differences of relative risks by birth cohort between these two immigration groups occurred after 1960 (Figure 1 & eFigure 1(a,b,d)). Locals and long-stay immigrants in pancreatic and prostate cancer perform almost similar changes of relative risks by birth cohort effects all the time (eFigure 1(c,e)). Short-stay immigrants who have stayed in Hong Kong for ≤10 years had more fluctuating relative risks affected by period effects before 2020 than those for locals and long-stay immigrants. Lack of young cases, especially young short-stay immigrants, of prostate cancer leads to significant deviations and variances in age and cohort effects.

Figure 3-5, eFigure 2-6 in **Supplementary Material** illustrate the age-standardized mortality rates of six types of cancer from 1998 to 2021 and their projections by sex, immigrant status and age groups from 2022 to 2030, taking into account age, period, and birth cohort effects. Means and standard deviations of predictive mortality rates are shown in eTable 1-6 in **Supplementary Material**. For all ages projection (Figure 2 & eFigure 2-6), as approximately significant interactions between gender and immigration groups emerge for each type of cancer in each year ($p<0.05$), given the projected trends, immigrants for each gender, especially who have stayed in Hong Kong for > 10 years will suffer from higher mortality rates of cancer in each year than locals. Monotone decreasing trends or plateau of forecasting occur for both genders and all immigration groups in cancers, except for increasing trends for male immigrants who have stayed in Hong Kong for ≤10 years with colon cancer ($p < 0.05$, Avg +0.30 deaths/100,000 per annum) from 15.47 deaths/100,000 (95% CI: 11.28, 19.66) in 2021 to 18.50 deaths/100,000 (95% CI: 2.31, 34.69) in 2030, and male immigrants who have stayed in Hong Kong for > 10 years with pancreatic cancer ($p < 0.05$, Avg +0.72 deaths/100,000 per annum) from 16.30 deaths/100,000 (95% CI: 14.38,17.26) in 2021 to 23.49 deaths/100,000 (95% CI: 12.49, 34.49) in 2030. Most of predictive trends for younger cases (<60 years) and older cases (≥60 years) reach a consensus with those for all ages population, except for two phenomena: 1.) mortality rates of lung cancer for men immigrants ≤10 that insignificant trend for all ages



For peer review only - http://bmjopen.bmj.com/site/about/guidelines.xhtml

For peer review only

($p > 0.05$) vs. decline for younger cases ($p < 0.05$) vs. increase for older cases ($p < 0.05$); 2.) mortality rates of liver cancer for men immigrants >10 that decline for all ages ($p < 0.05$) vs. decline for younger cases ($p < 0.05$) vs. insignificant trend for older cases ($p > 0.05$). Some particular cases occur in the projection of prostate cancer that young long-stay male immigrants (0.44 deaths/100,000, 95% CI: 0, 1.05) aged less than 60 will be at lower mortality rate than locals (0.69 deaths/100,000, 95% CI: 0, 1.42) in 2030 (eTable 6). Compared with other cancers and immigration groups, male immigrants who have stayed in Hong Kong for >10 years with lung cancer would perform the most significant decline in predictive mean from 102.90 (95% CI: 98.14, 107.66) to 79.55 (95% CI: 47.46, 111.64) deaths per 100,000 population (Avg -2.34 deaths/100,000 per annum) (eTable 1), while the same immigration group with pancreatic cancer would indicate the most significant uptrend in each year of 16.30 (95% CI: 14.38,17.26) and 23.49 (95% CI: 12.49, 34.49) deaths per 100,000 population in 2021 and 2030, respectively (Avg +0.72 deaths/100,000 per annum) (eTable 4).

## Discussion

Early detection of cancer is positive and instructive for increasing chances of cure. Nevertheless, the high mortality rate of cancer results from late diagnosis among most patients after progression to more advanced or severe stages. Individuals at high risk of cancer, such as smokers, alcoholics or those who are frequently exposed to susceptible circumstances, should be screened for early detections to increase opportunities for cure [40]. Therefore, the differences in mortality rates among immigration groups are synonymous with detection means, therapies, and social history in disparate periods and areas.

While the changes in mortality rates by age for long-stay immigrants reached approximate harmony with those for locals, the changes in mortality rates by age for short-stay immigrants revealed clear differences with those for the other two populations. The group of long-stay immigrants had a higher risk of death from lung, colon and liver cancers than the other two immigration groups after the age of 60 years. Short-stay male immigrants were less likely to die from lung cancer before the age of 65 years. The contrast in age effects among the immigration groups was partially consistent with studies [25, 41] that highlighted the age effects for locals and immigrants on breast cancer mortality in Hong Kong and lung cancer incidence in Sweden, as they both showed similar trends and magnitudes between locals and immigrants before the age of 60 years. They are also compatible with the results in [42] that diagnosis of liver cancer is the most frequent among populations at 55 to 65 years old. According to these trends, young individuals, especially new young immigrant men, who have benefited from all-rounded development in mainland China and Hong Kong, are more



For peer review only - http://bmjopen.bmj.com/site/about/guidelines.xhtml

likely to seek early detection and be treated for cancers using more advanced treatments [43]. Differences in birth cohort effects among immigrant groups partially comply with the interpretation above.

We observed significant trends of cohort effects among locals and immigrants. These findings are partially consistent but subtly different from previous findings, regarding the effect of immigration status on cancers. Zhao et al. [25] described multiple peaks of cohort effects on breast cancer mortality between locals and immigrants in Hong Kong, as well as a significant decline in cohort effects after 1950. In contrast, Sung et al. [44] investigated the difference in breast cancer incidence between Chinese Americans and non-Hispanic whites in the U.S. and emphasized that Chinese Americans were at lower risk of breast cancer than non-Hispanic whites born in the same year. Here, we interpret the cohort-driven trends resulting from the intricacy of social history and lifestyle. Compared to a relatively stable social development in Hong Kong, representing downward trends of relative risks for locals, wars and social instability in mainland China resulted in several immigration waves from mainland China to Hong Kong before 1950. Additionally, remarkable increasing trends were recorded for new immigrants after 1950, which corresponded to the economic downturn after wars and famine between 1959 and 1961 during their youth [45]. The increasing trends for new immigrants and similar trends for locals and long-stay immigrants were consistent with the finding that nutrient deficiency contributes to a higher risk of severe mortality rates of cancers [46]. Furthermore, we speculate that these trends, especially those for locals and long-stay immigrants, are most likely attributed to social development and personal behaviors, such as daily habits, occupational history, different diagnoses and treatments, and domestic environmental exposures. Notably, short-stay immigrants suffered from a lower risk of death from colon cancer for all ages (eFigure 1a in **Supplementary Material**). As locals and immigrants in Hong Kong transitioned to more westernized lifestyles, higher consumption of meat was associated with a higher risk of these types of cancer, whereas consumption of vegetables had a strong protective effect against pancreatic cancer, and moderate consumption of coffee appeared to be beneficial against lung cancer [47,48]. Further studies on potential risk factors are required.

Short-stay immigrants had more fluctuating and non-stationary but inconspicuous relative risks by period effects before 2021 than locals and long-stay immigrants. Cumulatively, an arch pattern and fluctuating curve depicting period effects externally resulted in an arch pattern of age-standardized mortality rates for short-stay immigrant women and irregular rates for short-stay immigrant men before 2021. The external performance of different period effects on mortality rates could be most likely attributed to the higher effect of different lifestyles and social development on new immigrants than on long-stay immigrants and locals in Hong Kong. For the age-standardized mortality rates and projections, consistent with



For peer review only - http://bmjopen.bmj.com/site/about/guidelines.xhtml

previous findings [49,50], we predict that the mortality rates of cancer in Hong Kong after 2021 will continue to decline or remain relatively stable, consistent with the trends before 2020, except for male immigrants who have stayed in Hong Kong for ≤10 years with colon cancer and male immigrants who have stayed in Hong Kong for >10 years with pancreatic cancer. Men will be at higher risk of mortality rates of cancer than women, regardless of immigration status. They are also compatible with the results in [4] that men suffer from a higher risk of these types of cancer than women, excluding prostate cancer. Furthermore, new immigrant women will be at lower risk than local women, even though long-stay immigrants will suffer from higher mortality rates than locals in the future. Potential interpretations could be consistent with those for birth cohort effects, as age and period effects are considered as confounders of cohort effect.

In the past few decades, spurred by an increasing burden of high incidence and mortality rates of cancer, several studies focused on the inherent identification dilemma of three effects in the APC model. Further, complicated population distribution and immigration status in Hong Kong, one of the areas with the highest population density and migration frequency in the world, have intricate causes and inherent dynamics of cancer and other diseases. To our knowledge, few studies have assessed the relationship between immigration status and cancer mortality. Therefore, this study is original to examine the effect of the length of stay in Hong Kong and origins of previous residence on cancer deaths, which is instructive for further immigration policy-making and targeted strategies of disease detection and intervention. However, this study had several limitations. Given the non-identifiability problem in age-period-cohort models, we could only depict trends and variations among different immigration and sex groups, as illustrated in figures, and insufficiently perform the estimates of the contributions of three effects or subgroups to mortality rates. Furthermore, we adopted a cubic smoothing spline to estimate populations of immigrants and locals due to the large proportion of unspecified immigration status from official demographic projections. A few acceptable cases resulted in a limited type of cancer so that some common cancers, such as the ovary and cervix, were discarded. Since the issue of quantification, the future perspective of cancer therapies and techniques have not been considered in the model of projection.

## Conclusion

We conclude that immigrants, especially short-stay immigrants, had more pronounced fluctuations of mortality rates by age and of relative risks by cohort and period effects for six types of cancers than those of long-stay immigrants and locals. Male immigrants who have stayed in Hong Kong for ≤10 years with colon cancer and male immigrants who have stayed in Hong Kong for >10 years with pancreatic cancer would perform significant uptrend in the



For peer review only - http://bmjopen.bmj.com/site/about/guidelines.xhtml

330 future, while other immigration groups for each type of cancer would continue to decline or
331 remain relatively stable. Immigrants for each gender in Hong Kong would suffer from higher
332 mortality risks of cancers than locals in the future.

For peer review only



For peer review only - http://bmjopen.bmj.com/site/about/guidelines.xhtml

## Declaration

### Ethical approval and consent to participate

Ethical approval and consent to participate are not applicable. This study does not directly involve human participants. Data was obtained from the Census and Statistics Department of Hong Kong.

### Consent for publication

Not applicable.

### Data availability statement

Data are available upon reasonable request.

### Contributors

Yanji Zhao: Methodology, Formal analysis, Data Curation, Writing - Original Draft, Visualization. Zian Zhuang: Methodology, Formal analysis, Data Curation, Writing - Review & Editing. Lin Yang: Validation, Writing - Review & Editing. Daihai He: Conceptualization, Writing - Review & Editing, Supervision

### Funding

The work described in this paper was partially supported by a grant from the Research Grants Council of the Hong Kong Special Administrative Region, China (HKU C7123-20G).

### Competing interests

None declared.

For peer review only



For peer review only - http://bmjopen.bmj.com/site/about/guidelines.xhtml

# References


1.Fan S-C. The population projection of Hong Kong. Southeast Asian Journal of Social Science. 1974;2(1/2):105-17.

2.Department CaS. Hong Kong Statistics 1947-1967 (Report). https://www.statistics.gov.hk/pub/hist/1961_1970/B10100031967AN67E0100.pdf Accessed 4th May 2019.

3. Department CaS. Demographic Trends in Hong Kong 1981-2011 (Report). http://www.statistics.gov.hk/pub/B1120017032012XXXXB0100.pdf, Accessed 4th May 2019.

4. Sung H, Ferlay J, Siegel RL, Laversanne M, Soerjomataram I, Jemal A, et al. Global Cancer Statistics 2020: GLOBOCAN Estimates of Incidence and Mortality Worldwide for 36 Cancers in 185 Countries. CA: A Cancer Journal for Clinicians. 2021 2021/05/01;71(3):209-49. doi: https://doi.org/10.3322/caac.21660.

5. Wang XR, Chiu YL, Qiu H, Au JSK, Yu ITS. The roles of smoking and cooking emissions in lung cancer risk among Chinese women in Hong Kong. Annals of Oncology. 2009 2009/04/01/;20(4):746-51. doi: https://doi.org/10.1093/annonc/mdn699.

6. Chiu Y-L, Wang X-R, Qiu H, Yu IT-S. Risk factors for lung cancer: a case–control study in Hong Kong women. Cancer Causes & Control. 2010 2010/05/01;21(5):777-85. doi: 10.1007/s10552-010-9506-9.

7. Office on S, Health. Publications and Reports of the Surgeon General. Women and Smoking: A Report of the Surgeon General. Atlanta (GA): Centers for Disease Control and Prevention (US); 2001.

8. Escobedo LG, Peddicord JP. Smoking prevalence in US birth cohorts: the influence of gender and education. American Journal of Public Health. 1996 1996/02/01;86(2):231-6. doi: 10.2105/AJPH.86.2.231.

9. Husten CG, Shelton DM, Chrismon JH, Lin YC, Mowery P, Powell FA. Cigarette smoking and smoking cessation among older adults: United States, 1965-94. Tobacco Control. 1997;6(3):175. doi: 10.1136/tc.6.3.175.

10. Bolego C, Poli A, Paoletti R. Smoking and gender. Cardiovascular Research. 2002;53(3):568-76. doi: 10.1016/S0008-6363(01)00520-X.

11. Doll R, Hill AB. The mortality of doctors in relation to their smoking habits; a preliminary report. Br Med J. 1954;1(4877):1451-5. PMID: 13160495. doi: 10.1136/bmj.1.4877.1451.

12. Ramada Rodilla JM, Calvo Cerrada B, Serra Pujadas C, Delclos GL, Benavides FG. Fiber burden and asbestos-related diseases: an umbrella review. Gaceta Sanitaria. 2021 2021/06/11/. doi: https://doi.org/10.1016/j.gaceta.2021.04.001.

13. Collishaw NE, Kirkbride J, Wigle DT. Tobacco smoke in the workplace: an occupational health hazard. Can Med Assoc J. 1984;131(10):1199-204. PMID: 6498670.

14. Dresler CM, Fratelli C, Babb J, Everley L, Evans AA, Clapper ML. Gender differences in genetic susceptibility for lung cancer. Lung Cancer. 2000 2000/12/01/;30(3):153-60. doi: https://doi.org/10.1016/S0169-5002(00)00163-X.

15. Alexandrov K, Cascorbi I, Rojas M, Bouvier G, Kriek E, Bartsch H. CYP1A1 and GSTM1 genotypes affect benzo[ a ]pyrene DNA adducts in smokers' lung: comparison with aromatic/hydrophobic adduct formation. Carcinogenesis. 2002;23(12):1969-77. doi:




For peer review only - http://bmjopen.bmj.com/site/about/guidelines.xhtml

10.1093/carcin/23.12.1969.

16. Samet JM. Radon and Lung Cancer. JNCI: Journal of the National Cancer Institute. 1989;81(10):745-58. doi: 10.1093/jnci/81.10.745.

17. Darby S, Hill D, Auvinen A, Barros-Dios JM, Baysson H, Bochicchio F, et al. Radon in homes and risk of lung cancer: collaborative analysis of individual data from 13 European case-control studies. BMJ. 2005;330(7485):223. doi: 10.1136/bmj.38308.477650.63.

18. Raaschou-Nielsen O, Andersen ZJ, Beelen R, Samoli E, Stafoggia M, Weinmayr G, et al. Air pollution and lung cancer incidence in 17 European cohorts: prospective analyses from the European Study of Cohorts for Air Pollution Effects (ESCAPE). The Lancet Oncology. 2013 2013/08/01/;14(9):813-22. doi: https://doi.org/10.1016/S1470-2045(13)70279-1.

19. *2018 Summary-20190719*. Retrieved August 26, 2022, from https://www.who.int/docs/default-source/wpro---documents/countries/china/2018-gats-china-factsheet-cn-en.pdf?sfvrsn=3f4e2da9_2

20. *Thematic household survey*.Retrieved August 26, 2022, from https://www.censtatd.gov.hk/en/data/stat_report/product/B1130201/att/B11302702020XXXXB0100.pdf

21. Abubakar II, Tillmann T, Banerjee A. Global, regional, and national age-sex specific all-cause and cause-specific mortality for 240 causes of death, 1990-2013: a systematic analysis for the Global Burden of Disease Study 2013. Lancet. 2015 Jan 10;385(9963):117-71.

22. Estimated alcohol consumption per capita in Hong Kong. Change4Health. (n.d.). Retrieved December 1, 2022, from https://www.change4health.gov.hk/en/alcohol_aware/figures/alcohol_consumption/index.html

23. World Health Organization. Global status report on alcohol and health 2018. World Health Organization; 2019 Feb 14.

24. Wild C. World cancer report 2014. Wild CP, Stewart BW, editors. Geneva, Switzerland: World Health Organization; 2014.

25. Zhao S, Dong H, Qin J, Liu H, Li Y, Chen Y, et al. Breast cancer mortality in Chinese women: does migrant status play a role? Annals of Epidemiology. 2019 2019/12/01/;40:28-34.e2. doi: https://doi.org/10.1016/j.annepidem.2019.10.006.

26. Gomez SL, Yang J, Lin S-W, McCusker M, Sandler A, Cheng I, et al. Incidence trends of lung cancer by immigration status among Chinese Americans. Cancer Epidemiol Biomarkers Prev. 2015;24(8):1157-64. PMID: 25990553. doi: 10.1158/1055-9965.EPI-15-0123.

27.Hemminki K, Li X, Czene K. Cancer risks in first-generation immigrants to Sweden. International Journal of Cancer. 2002 2002/05/10;99(2):218-28. doi: https://doi.org/10.1002/ijc.10322.

28. Vanthomme K, Rosskamp M, De Schutter H, Vandenheede H. Lung cancer incidence differences in migrant men in Belgium, 2004–2013: histology-specific analyses. BMC Cancer. 2021 2021/03/30;21(1):328. doi: 10.1186/s12885-021-08038-6.

29. Schooling M, Leung GM, Janus ED, Ho SY, Hedley AJ, Lam TH. Childhood migration and cardiovascular risk. International Journal of Epidemiology. 2004;33(6):1219-26. doi: 10.1093/ije/dyh221.

30. Leung JYY, Li AM, Leung GM, Schooling CM. Mode of delivery and childhood hospitalizations for asthma and other wheezing disorders. Clinical & Experimental Allergy.

For peer review only



For peer review only - http://bmjopen.bmj.com/site/about/guidelines.xhtml

2015 2015/06/01;45(6):1109-17. doi: https://doi.org/10.1111/cea.12548.
31. Baker A, Bray I. Bayesian projections: what are the effects of excluding data from younger age groups?. American Journal of Epidemiology. 2005 Oct 15;162(8):798-805.
32. Rosenberg PS, Anderson WF. Age-Period-Cohort Models in Cancer Surveillance Research: Ready for Prime Time? APC Models. Cancer Epidemiology, Biomarkers & Prevention. 2011 Jul 1;20(7):1263-8.
33. Holford T. Analyzing the effects of age, period and cohort on incidence and mortality rates. Stat Meth Med Res. 1992;1:317-37.
34. Brookmeyer R, Stroup DF, editors. Monitoring the health of populations: statistical principles and methods for public health surveillance. Oxford University Press; 2004.
35. Yang Y, Land KC. Age-period-cohort analysis: New models, methods, and empirical applications. Taylor & Francis; 2013.
36. Robertson C, Gandini S, Boyle P. Age-period-cohort models: a comparative study of available methodologies. Journal of clinical epidemiology. 1999 Jun 1;52(6):569-83.
37. Riebler A, Held L. Projecting the future burden of cancer: Bayesian age–period–cohort analysis with integrated nested Laplace approximations. Biometrical Journal. 2017 May;59(3):531-49.
38. Knoll M, Furkel J, Debus J, Abdollahi A, Karch A, Stock C. An R package for an integrated evaluation of statistical approaches to cancer incidence projection. BMC medical research methodology. 2020 Dec;20(1):1-1.
39. Ahmad OB, Boschi-Pinto C, Lopez AD, Murray CJ, Lozano R, Inoue M. Age standardization of rates: a new WHO standard. Geneva: World Health Organization. 2001 Jan;9(10):1-4.
40. Sung H, Ferlay J, Siegel RL, Laversanne M, Soerjomataram I, Jemal A, Bray F. Global cancer statistics 2020: GLOBOCAN estimates of incidence and mortality worldwide for 36 cancers in 185 countries. CA: a cancer journal for clinicians. 2021 May;71(3):209-49.
41. Mousavi SM, Fallah M, Sundquist K, Hemminki K. Age-and time-dependent changes in cancer incidence among immigrants to Sweden: colorectal, lung, breast and prostate cancers. International journal of cancer. 2012 Jul 15;131(2):E122-8.
42. National Cancer Institute. SEER stat fact sheets: liver and intrahepatic bile duct cancer.
43. Wu X, Chung VC, Hui EP, Ziea ET, Ng BF, Ho RS, Tsoi KK, Wong S, Wu JC. Effectiveness of acupuncture and related therapies for palliative care of cancer: overview of systematic reviews. Scientific reports. 2015 Nov 26;5(1):1-5.
44. Sung H, Rosenberg PS, Chen WQ, Hartman M, Lim WY, Chia KS, Wai-Kong Mang O, Tse L, Anderson WF, Yang XR. The impact of breast cancer-specific birth cohort effects among younger and older C hinese populations. International journal of cancer. 2016 Aug 1;139(3):527-34.
45. The world economy volume 1: a millennial perspective, 2, Historical statistics: Academic Foundation, Gurgaon, India (2007)
46. Elias SG, Peeters PH, Grobbee DE, van Noord PA. The 1944-1945 Dutch famine and subsequent overall cancer incidence. Cancer Epidemiology Biomarkers & Prevention. 2005 Aug;14(8):1981-5.
47. Chiu YL, Wang XR, Qiu H, Yu IT. Risk factors for lung cancer: a case–control study in Hong Kong women. Cancer Causes & Control. 2010 May;21(5):777-85.



For peer review only - http://bmjopen.bmj.com/site/about/guidelines.xhtml

48. Li J, Lam AS, Yau ST, Yiu KK, Tsoi KK. Antihypertensive treatments and risks of lung Cancer: A large population-based cohort study in Hong Kong. BMC cancer. 2021 Dec;21(1):1-9.

49. Du J, Sun H, Sun Y, Du J, Cao W, Sun S. Assessment of age, period, and cohort effects of lung cancer incidence in Hong Kong and projection up to 2030 based on changing demographics. American Journal of Cancer Research. 2021;11(12):5902.

50. *Centre for Health Protection, Department of Health - Lung Cancer*. Centre for Health Protection. Retrieved August 10, 2022, from https://www.chp.gov.hk/en/healthtopics/content/25/49.html

For peer review only



For peer review only - http://bmjopen.bmj.com/site/about/guidelines.xhtml

**FIGURE LEGENDS**

**Figure 1**. Parameter estimates of age (a), cohort (b) and period (c) effects based on an age-period-cohort model of male lung cancer mortality rates by immigrant groups: locals, immigrants stay in Hong Kong for more than 10 years and immigrants stay in Hong Kong for less than or equal to 10 years. Age effect was assessed by mortality (left axis). Cohort and period effects were assessed by relative risk (right axis), 95% confidence intervals are shown as shaded bands.

**Figure 2**. Parameter estimates of age (a), cohort (b) and period (c) effects based on an age-period-cohort model of female lung cancer mortality rates by immigrant groups: locals, immigrants stay in Hong Kong for more than 10 years and immigrants stay in Hong Kong for less than or equal to 10 years. Age effect was assessed by mortality (left axis). Cohort and period effects were assessed by relative risk (right axis), 95% confidence intervals are shown as shaded bands.

**Figure 3.** Projections of lung cancer mortality rates by gender and immigrant status from 2022 to 2030. Observations are shown as dots with the predictive distribution between the 5% and 95% quantile, whereby each lighter shade of red represents an additional 10% predictive CI. The predictive mean is shown as black solid line and the vertical dashed line indicates where prediction started.

**Figure 4.** Projections of lung cancer mortality rates for the population younger than 60 by gender and immigrant status from 2022 to 2030. Observations are shown as dots with the predictive distribution between the 5% and 95% quantile, whereby each lighter shade of red represents an additional 10% predictive CI. The predictive mean is shown as black solid line and the vertical dashed line indicates where prediction started.

**Figure 5.** Projections of lung cancer mortality rates for the population older than or equal to 60 by gender and immigrant status from 2022 to 2030. Observations are shown as dots with the predictive distribution between the 5% and 95% quantile, whereby each lighter shade of red represents an additional 10% predictive CI. The predictive mean is shown as black solid line and the vertical dashed line indicates where prediction started.

For peer review only



For peer review only - http://bmjopen.bmj.com/site/about/guidelines.xhtml

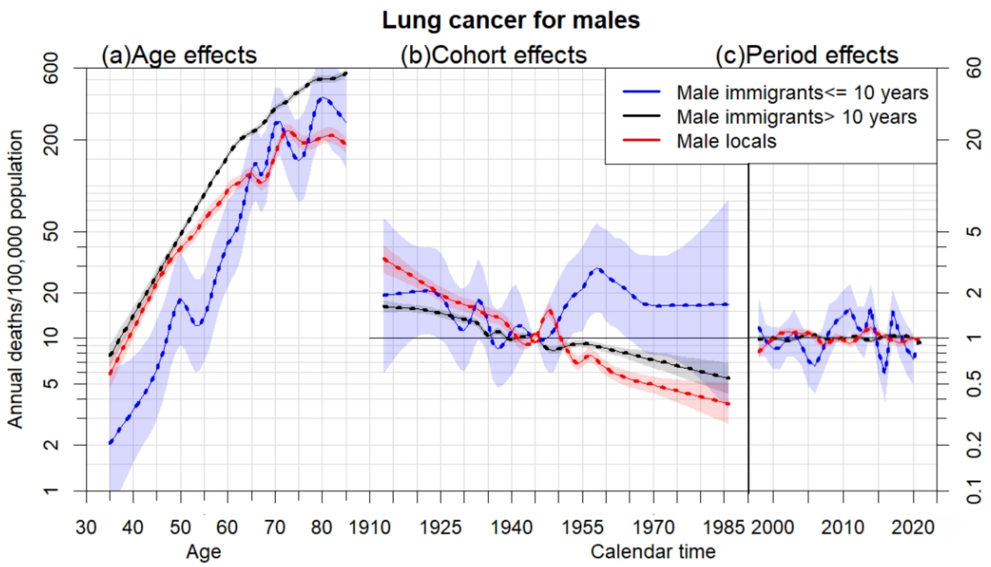


Figure 1. Parameter estimates of age (a), cohort (b) and period (c) effects based on an age-period-cohort model of male lung cancer mortality rates by immigrant groups: locals, immigrants stay in Hong Kong for more than 10 years and immigrants stay in Hong Kong for less than or equal to 10 years. Age effect was assessed by mortality (left axis). Cohort and period effects were assessed by relative risk (right axis), 95% confidence intervals are shown as shaded bands.

533x343mm (57 x 57 DPI)

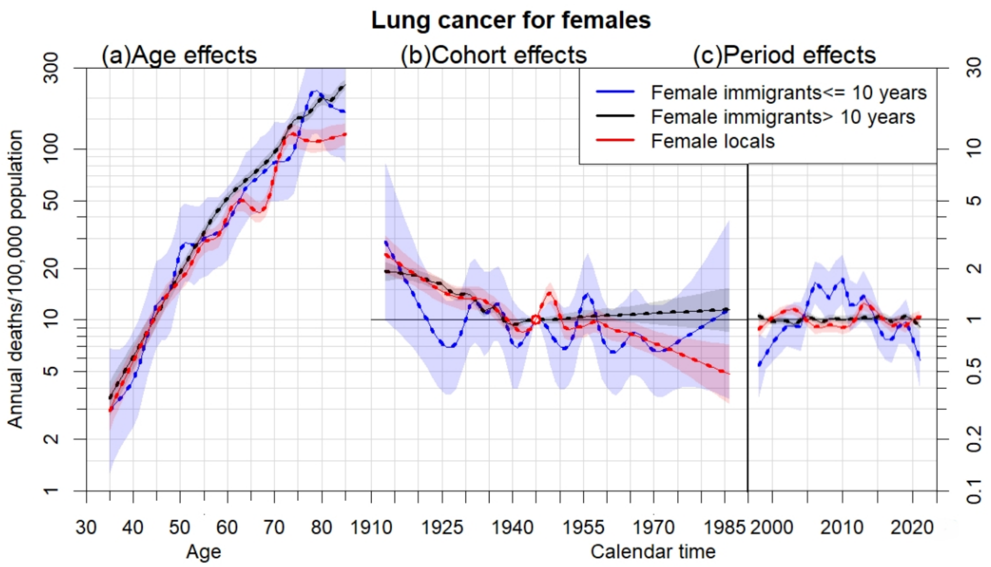


Figure 2. Parameter estimates of age (a), cohort (b) and period (c) effects based on an age-period-cohort model of female lung cancer mortality rates by immigrant groups: locals, immigrants stay in Hong Kong for more than 10 years and immigrants stay in Hong Kong for less than or equal to 10 years. Age effect was assessed by mortality (left axis). Cohort and period effects were assessed by relative risk (right axis), 95% confidence intervals are shown as shaded bands.

533x343mm (57 x 57 DPI)

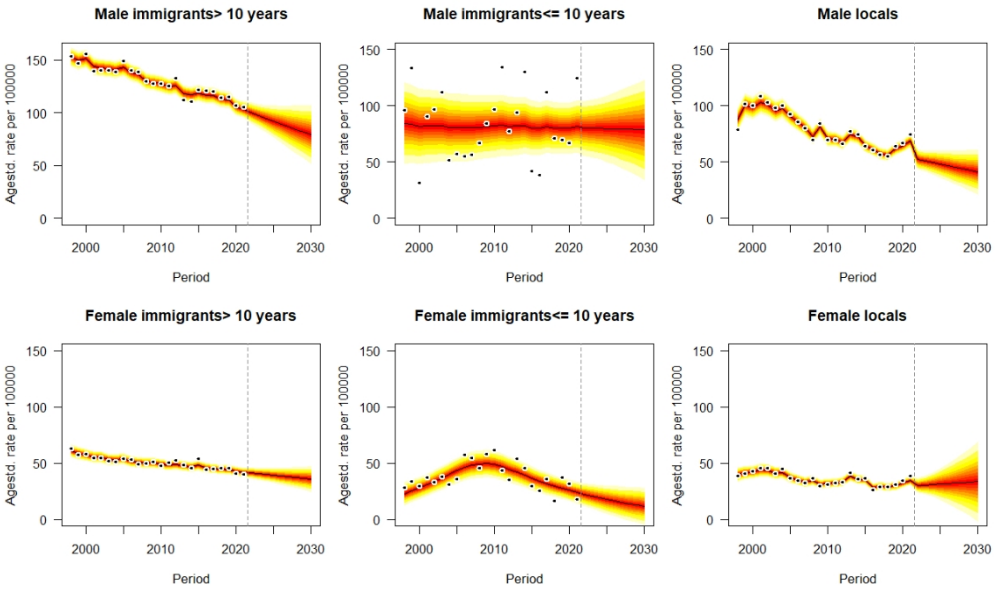


Figure 3. Projections of lung cancer mortality rates by gender and immigrant status from 2022 to 2030. Observations are shown as dots with the predictive distribution between the 5% and 95% quantile, whereby each lighter shade of red represents an additional 10% predictive CI. The predictive mean is shown as black solid line and the vertical dashed line indicates where prediction started.

570x343mm (57 x 57 DPI)

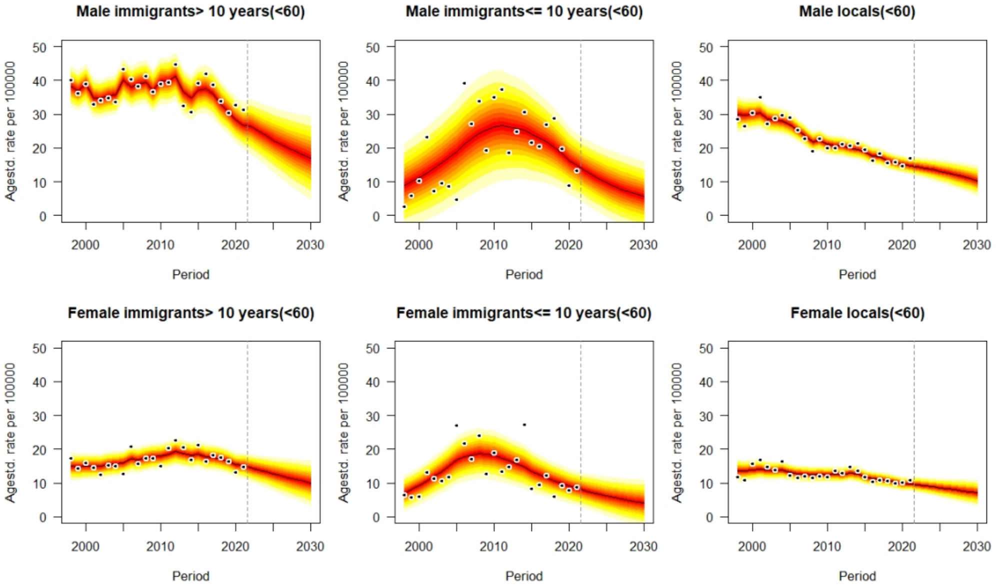


Figure 4. Projections of lung cancer mortality rates for the population younger than 60 by gender and immigrant status from 2022 to 2030. Observations are shown as dots with the predictive distribution between the 5% and 95% quantile, whereby each lighter shade of red represents an additional 10% predictive CI. The predictive mean is shown as black solid line and the vertical dashed line indicates where prediction started.

570x343mm (57 x 57 DPI)

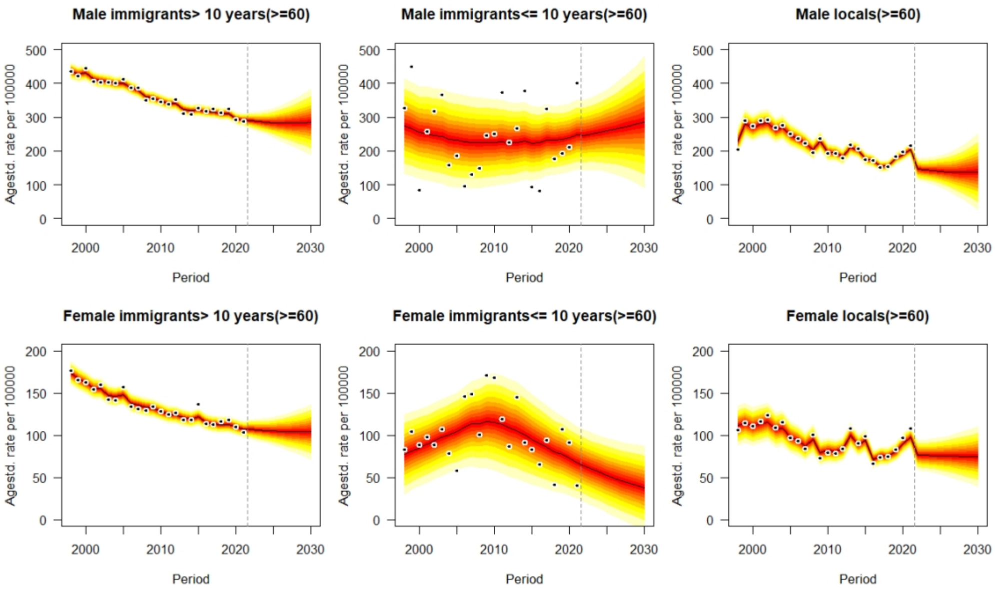


Figure 5. Projections of lung cancer mortality rates for the population older than or equal to 60 by gender and immigrant status from 2022 to 2030. Observations are shown as dots with the predictive distribution between the 5% and 95% quantile, whereby each lighter shade of red represents an additional 10% predictive CI. The predictive mean is shown as black solid line and the vertical dashed line indicates where prediction started.

570x343mm (57 x 57 DPI)

# Supplementary Material for

# "An age-period-cohort analysis and projection of cancer mortality in Hong Kong, 1998–2030"

For peer review only

## APC Figures

eFigure 1(a) Colon cancer ......3

eFigure 1(b) Liver cancer ......4

eFigure 1(c) Pancreatic cancer ......5

eFigure 1(d) Stomach cancer ......6

eFigure 1(e) Prostate cancer ......7

## Projection Figures

Colon cancer

eFigure 2(a)Projection (all ages) ......8

eFigure 2(b)Projection (<60 years) ......9

eFigure 2(c)Projection (⩾60 years) ......10

Liver cancer

eFigure 3(a)Projection (all ages) ......11

eFigure 3(b)Projection (<60 years) ......12

eFigure 3(c)Projection (⩾60 years) ......13

Pancreatic cancer

eFigure 4(a)Projection (all ages) ......14

eFigure 4(b)Projection (<60 years) ......15

eFigure 4(c)Projection (⩾60 years) ......16

Stomach cancer

eFigure 5(a)Projection (all ages) ......17

eFigure 5(b)Projection (<60 years) ......18



For peer review only - http://bmjopen.bmj.com/site/about/guidelines.xhtml

eFigure 5(c)Projection (⩾60 years) ... 19

Prostate cancer

eFigure 6 Projection (all ages, <60 years and ⩾60 years) ... 20

## Tables

eTable 1 Lung cancer ... 21

eTable 2 Colon cancer ... 22

eTable 3 Liver cancer ... 23

eTable 4 Pancreatic cancer ... 24

eTable 5 Stomach cancer ... 25

eTable 6 Prostate cancer ... 26

For peer review only



For peer review only - http://bmjopen.bmj.com/site/about/guidelines.xhtml

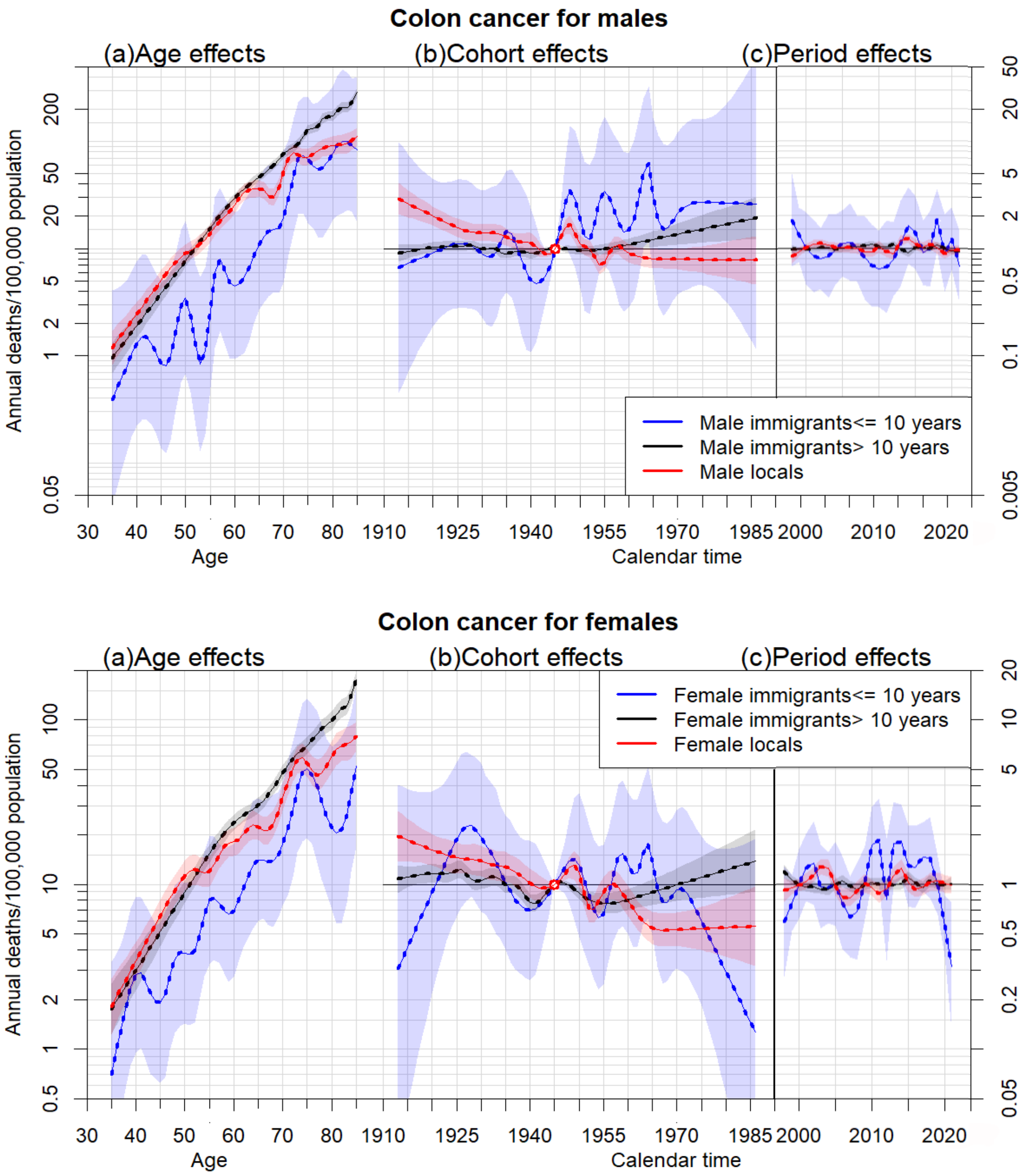


**eFigure 1(a)**. Parameter estimates of age (a), cohort (b) and period (c) effects based on an age-period-cohort model of male and female colon cancer mortality rates by immigrant groups: locals, immigrants stay in Hong Kong for more than 10 years and immigrants stay in Hong Kong for less than or equal to 10 years. Age effect was assessed by mortality (left axis). Cohort and period effects were assessed by relative risk (right axis), 95% confidence intervals are shown as shaded bands.



For peer review only - http://bmjopen.bmj.com/site/about/guidelines.xhtml

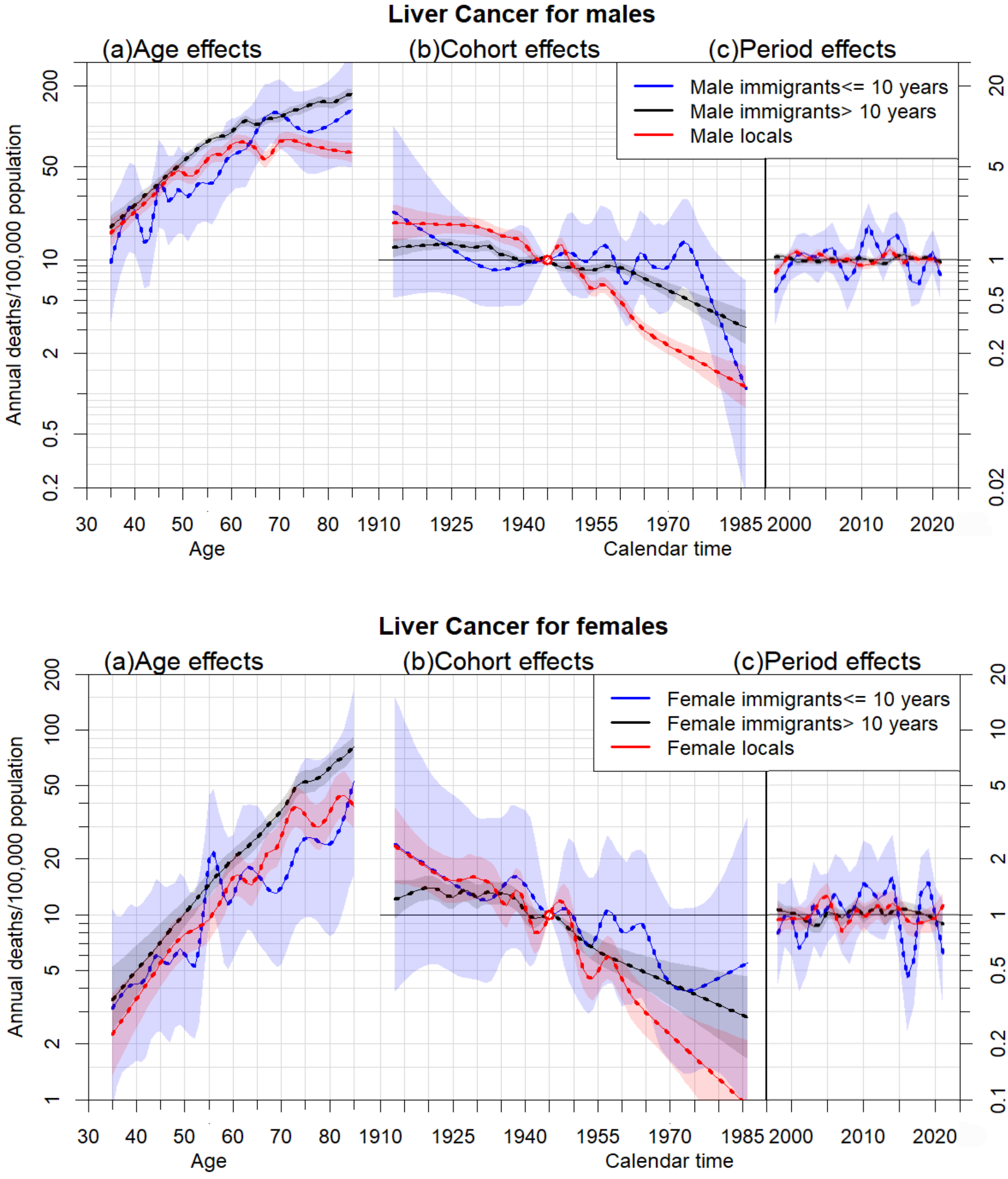


**eFigure 1(b)**. Parameter estimates of age (a), cohort (b) and period (c) effects based on an age-period-cohort model of male and female liver cancer mortality rates by immigrant groups: locals, immigrants stay in Hong Kong for more than 10 years and immigrants stay in Hong Kong for less than or equal to 10 years. Age effect was assessed by mortality (left axis). Cohort and period effects were assessed by relative risk (right axis), 95% confidence intervals are shown as shaded bands.

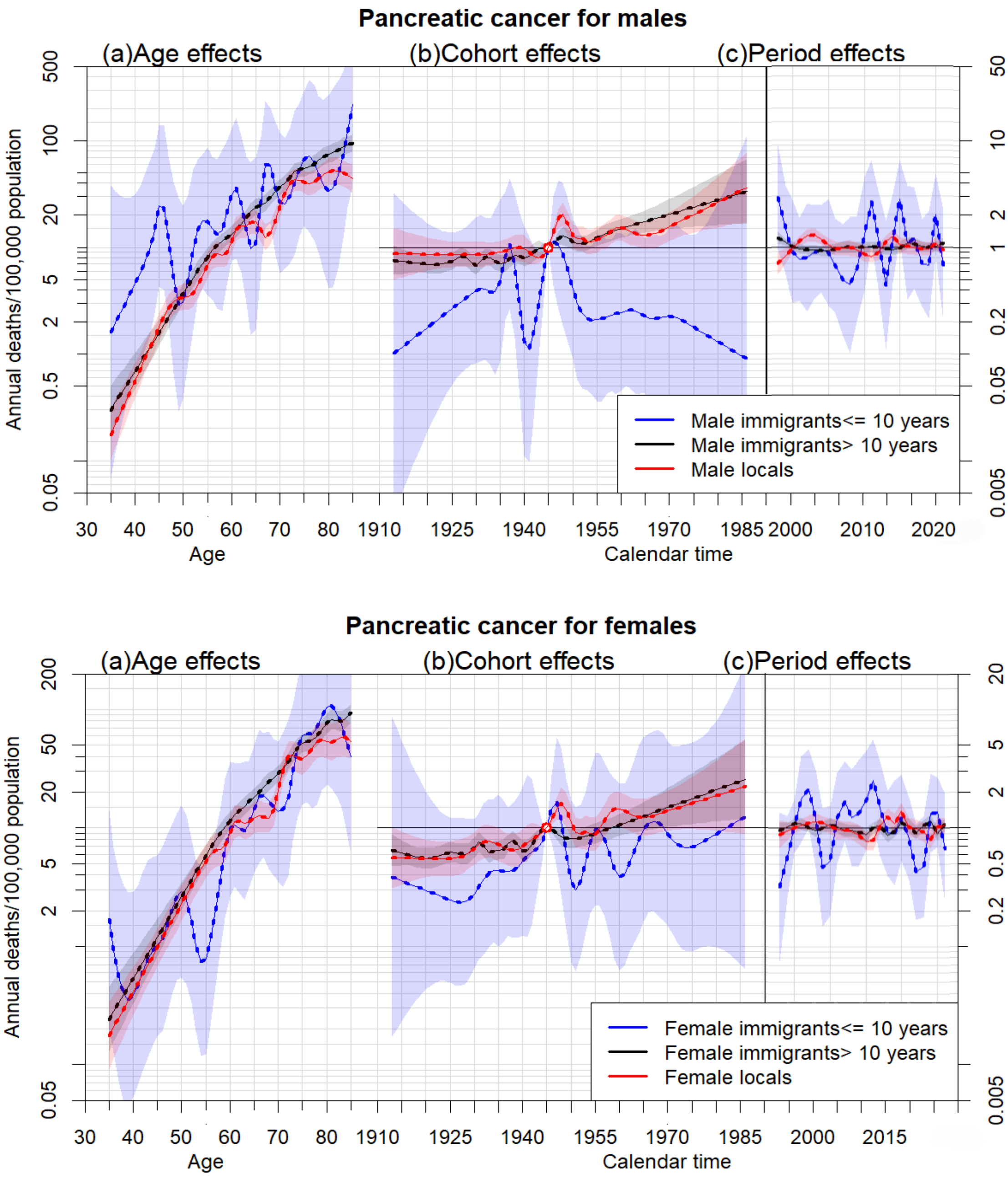


**eFigure 1(c)**. Parameter estimates of age (a), cohort (b) and period (c) effects based on an age-period-cohort model of male and female pancreatic cancer mortality rates by immigrant groups: locals, immigrants stay in Hong Kong for more than 10 years and immigrants stay in Hong Kong for less than or equal to 10 years. Age effect was assessed by mortality (left axis). Cohort and period effects were assessed by relative risk (right axis), 95% confidence intervals are shown as shaded bands.



For peer review only - http://bmjopen.bmj.com/site/about/guidelines.xhtml

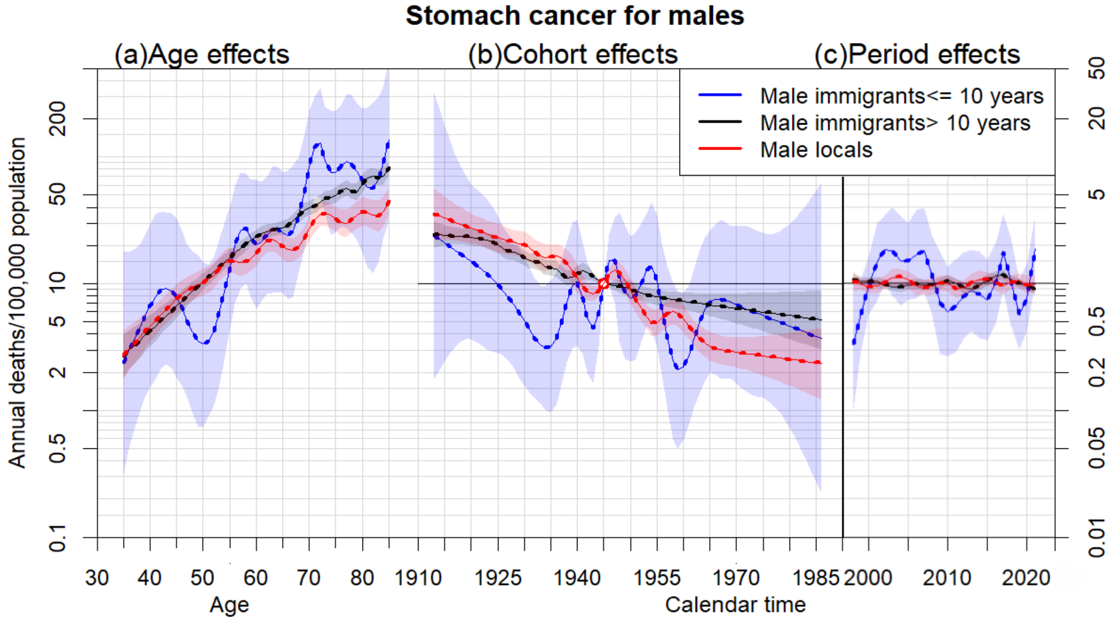


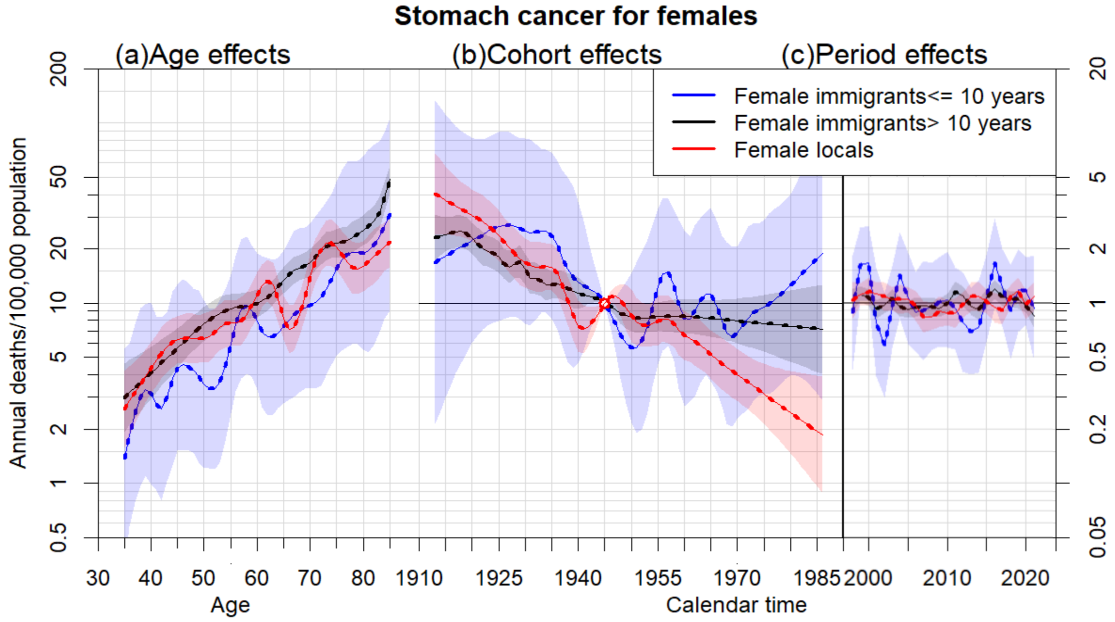


**eFigure 1(d)**. Parameter estimates of age (a), cohort (b) and period (c) effects based on an age-period-cohort model of male and female stomach cancer mortality rates by immigrant groups: locals, immigrants stay in Hong Kong for more than 10 years and immigrants stay in Hong Kong for less than or equal to 10 years. Age effect was assessed by mortality (left axis). Cohort and period effects were assessed by relative risk (right axis), 95% confidence intervals are shown as shaded bands.



For peer review only - http://bmjopen.bmj.com/site/about/guidelines.xhtml

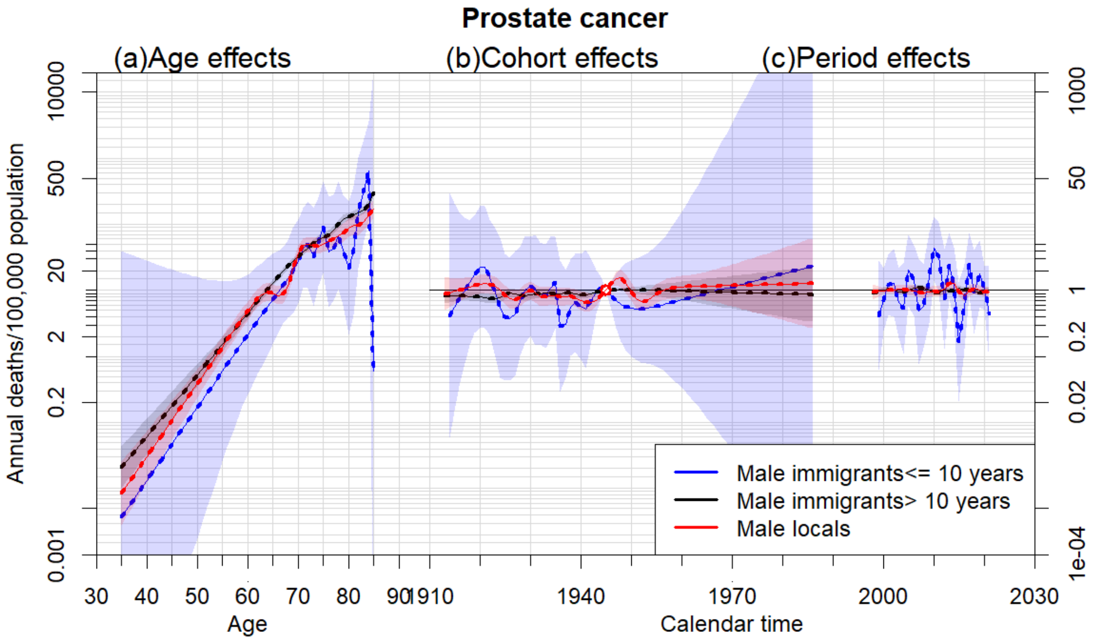


**eFigure 1(e)**. Parameter estimates of age (a), cohort (b) and period (c) effects based on an age-period-cohort model of male prostate cancer mortality rates by immigrant groups: locals, immigrants stay in Hong Kong for more than 10 years and immigrants stay in Hong Kong for less than or equal to 10 years. Age effect was assessed by mortality (left axis). Cohort and period effects were assessed by relative risk (right axis), 95% confidence intervals are shown as shaded bands.



For peer review only - http://bmjopen.bmj.com/site/about/guidelines.xhtml

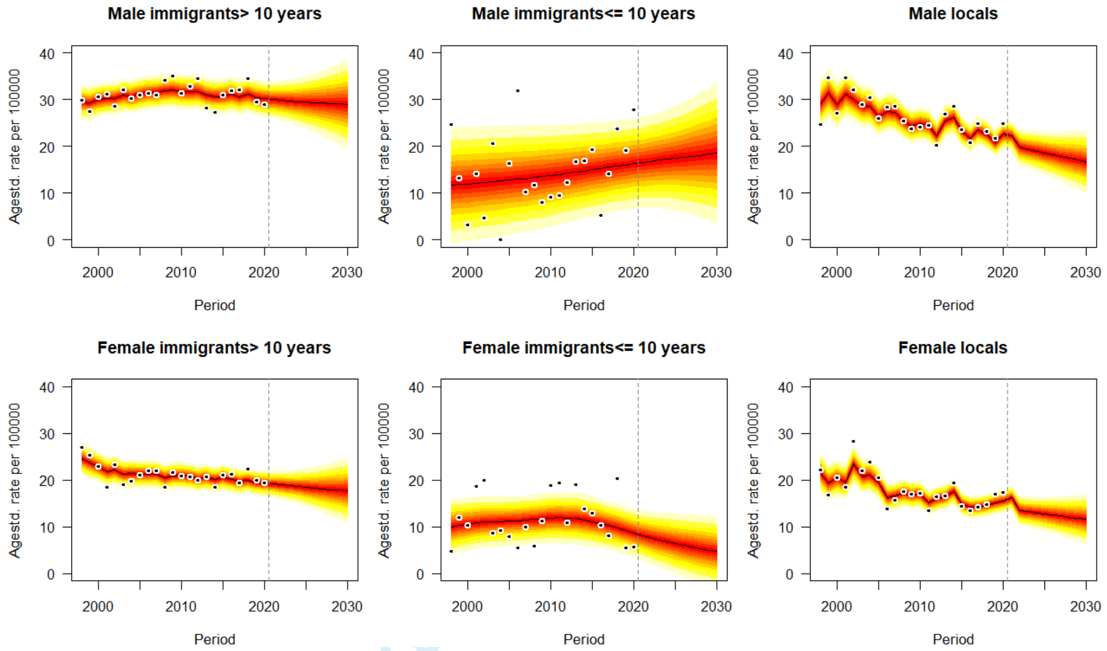


**eFigure 2(a).** Projections of colon cancer mortality rates by gender and immigrant status from 2022 to 2030. Observations are shown as dots with the predictive distribution between the 5% and 95% quantile, whereby each lighter shade of red represents an additional 10% predictive CI. The predictive mean is shown as black solid line and the vertical dashed line indicates where prediction started.



For peer review only - http://bmjopen.bmj.com/site/about/guidelines.xhtml

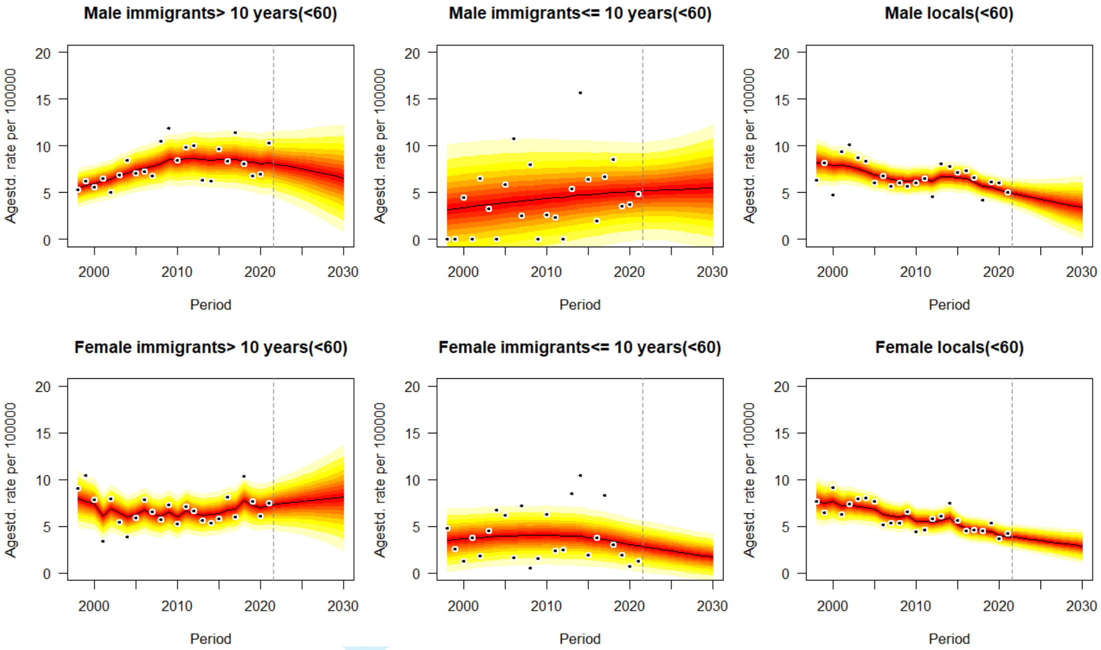


**eFigure 2(b).** Projections of colon cancer mortality rates for the population younger than 60 by gender and immigrant status from 2022 to 2030. Observations are shown as dots with the predictive distribution between the 5% and 95% quantile, whereby each lighter shade of red represents an additional 10% predictive CI. The predictive mean is shown as black solid line and the vertical dashed line indicates where prediction started.



For peer review only - http://bmjopen.bmj.com/site/about/guidelines.xhtml

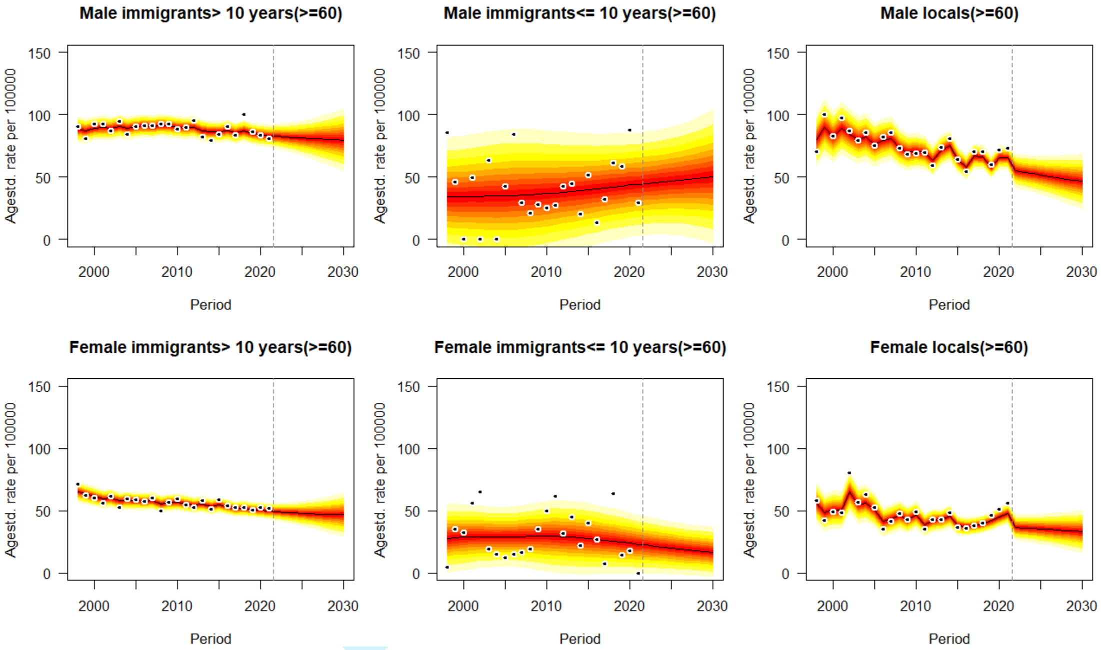


**eFigure 2(c).** Projections of colon cancer mortality rates for the population older than or equal to 60 by gender and immigrant status from 2022 to 2030. Observations are shown as dots with the predictive distribution between the 5% and 95% quantile, whereby each lighter shade of red represents an additional 10% predictive CI. The predictive mean is shown as black solid line and the vertical dashed line indicates where prediction started.



For peer review only - http://bmjopen.bmj.com/site/about/guidelines.xhtml

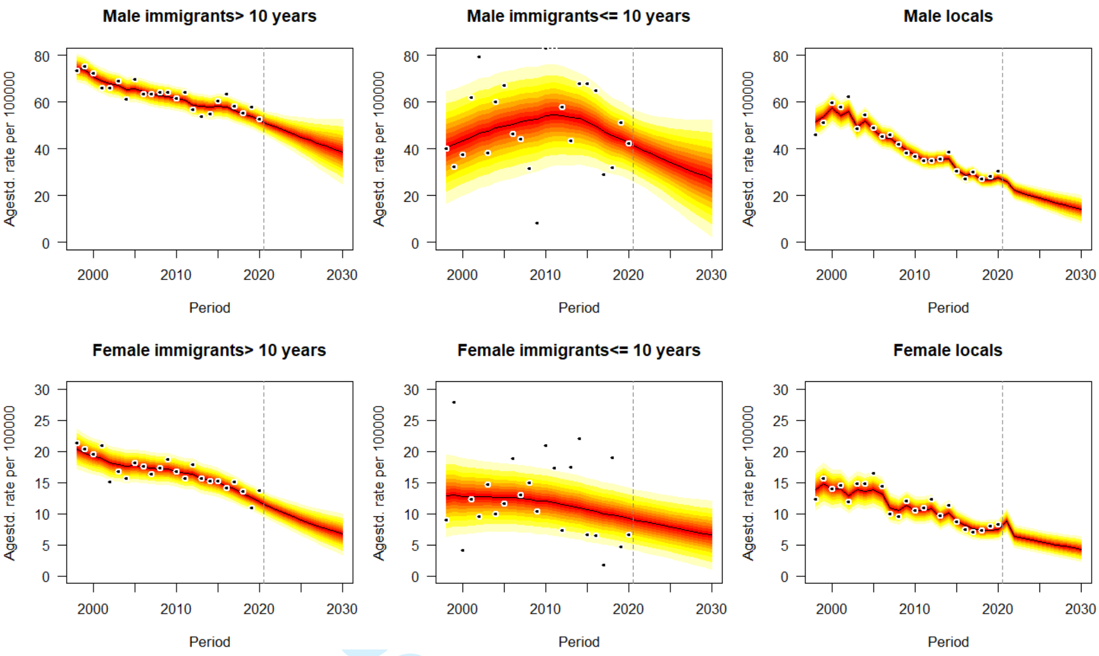


**eFigure 3(a).** Projections of liver cancer mortality rates by gender and immigrant status from 2022 to 2030. Observations are shown as dots with the predictive distribution between the 5% and 95% quantile, whereby each lighter shade of red represents an additional 10% predictive CI. The predictive mean is shown as black solid line and the vertical dashed line indicates where prediction started.

For peer review only



For peer review only - http://bmjopen.bmj.com/site/about/guidelines.xhtml

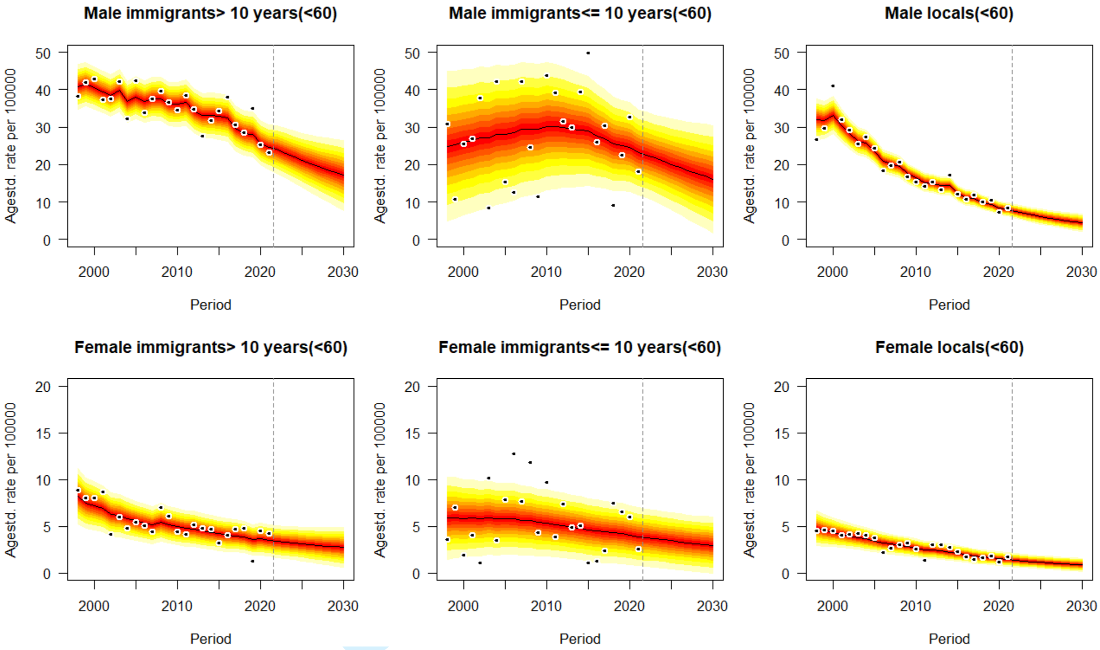


**eFigure 3(b).** Projections of liver cancer mortality rates for the population younger than 60 by gender and immigrant status from 2022 to 2030. Observations are shown as dots with the predictive distribution between the 5% and 95% quantile, whereby each lighter shade of red represents an additional 10% predictive CI. The predictive mean is shown as black solid line and the vertical dashed line indicates where prediction started.



For peer review only - http://bmjopen.bmj.com/site/about/guidelines.xhtml

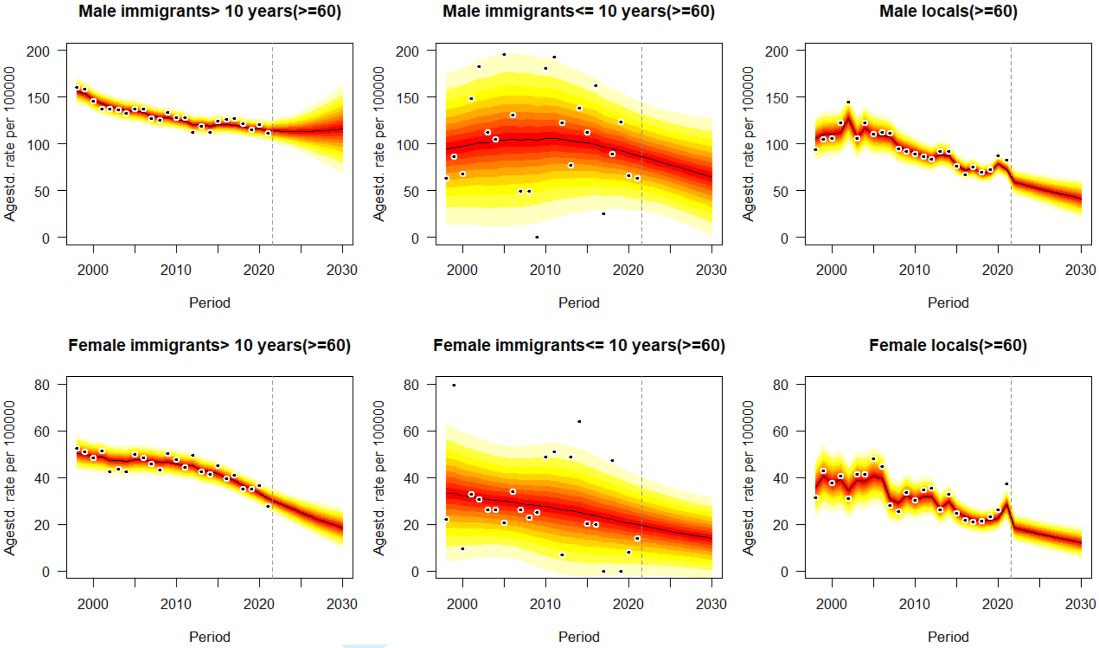


**eFigure 3(c).** Projections of liver cancer mortality rates for the population older than or equal to 60 by gender and immigrant status from 2022 to 2030. Observations are shown as dots with the predictive distribution between the 5% and 95% quantile, whereby each lighter shade of red represents an additional 10% predictive CI. The predictive mean is shown as black solid line and the vertical dashed line indicates where prediction started.



For peer review only - http://bmjopen.bmj.com/site/about/guidelines.xhtml

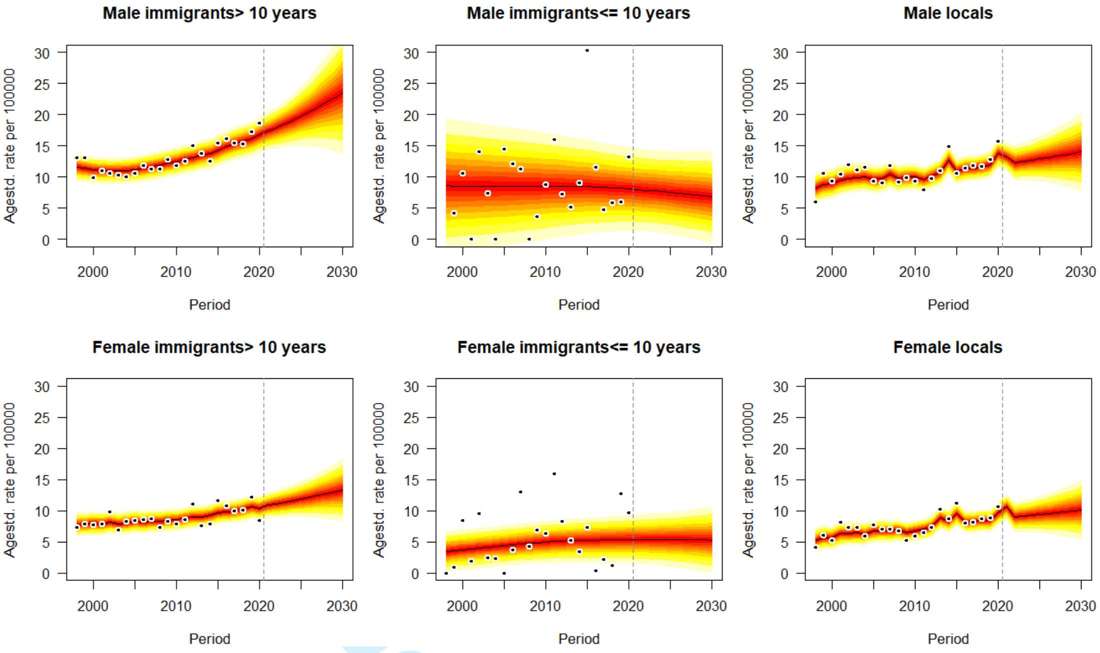


**eFigure 4(a).** Projections of pancreatic cancer mortality rates by gender and immigrant status from 2022 to 2030. Observations are shown as dots with the predictive distribution between the 5% and 95% quantile, whereby each lighter shade of red represents an additional 10% predictive CI. The predictive mean is shown as black solid line and the vertical dashed line indicates where prediction started.



For peer review only - http://bmjopen.bmj.com/site/about/guidelines.xhtml

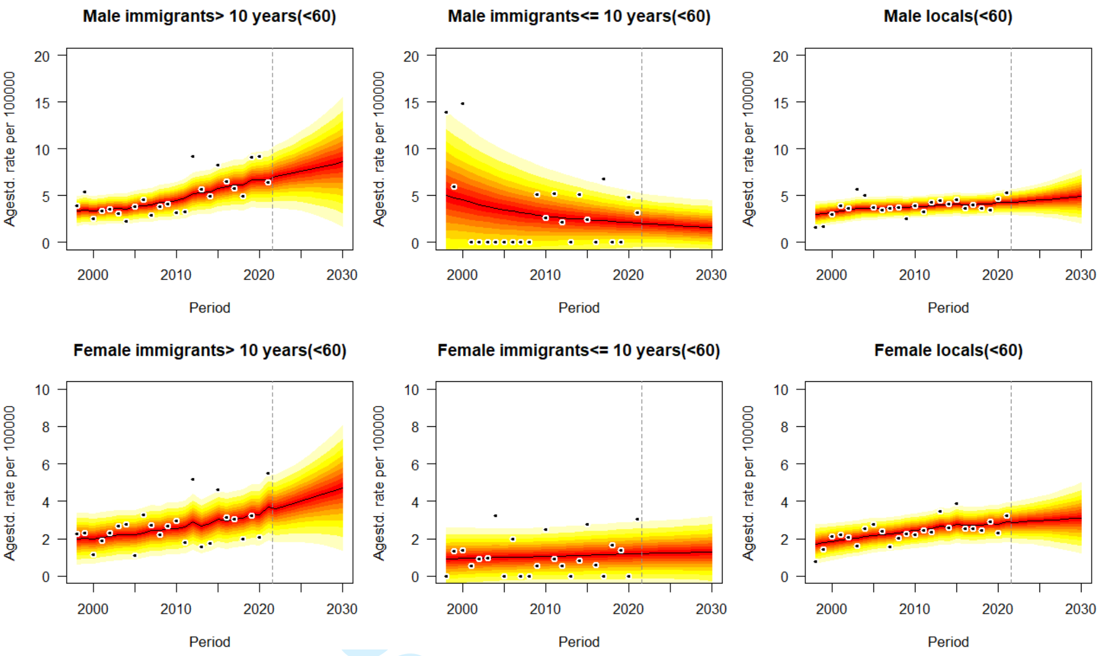


**eFigure 4(b).** Projections of pancreatic cancer mortality rates for the population younger than 60 by gender and immigrant status from 2022 to 2030. Observations are shown as dots with the predictive distribution between the 5% and 95% quantile, whereby each lighter shade of red represents an additional 10% predictive CI. The predictive mean is shown as black solid line and the vertical dashed line indicates where prediction started.

For peer review only



For peer review only - http://bmjopen.bmj.com/site/about/guidelines.xhtml

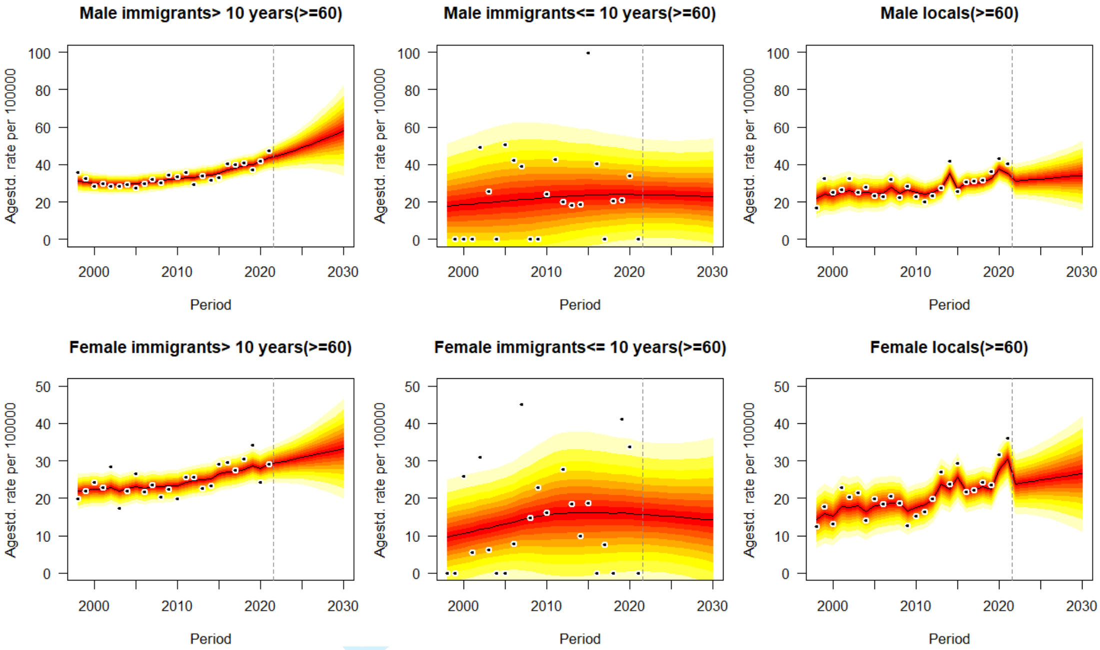


**eFigure 4(c).** Projections of pancreatic cancer mortality rates for the population older than or equal to 60 by gender and immigrant status from 2022 to 2030. Observations are shown as dots with the predictive distribution between the 5% and 95% quantile, whereby each lighter shade of red represents an additional 10% predictive CI. The predictive mean is shown as black solid line and the vertical dashed line indicates where prediction started.



For peer review only - http://bmjopen.bmj.com/site/about/guidelines.xhtml

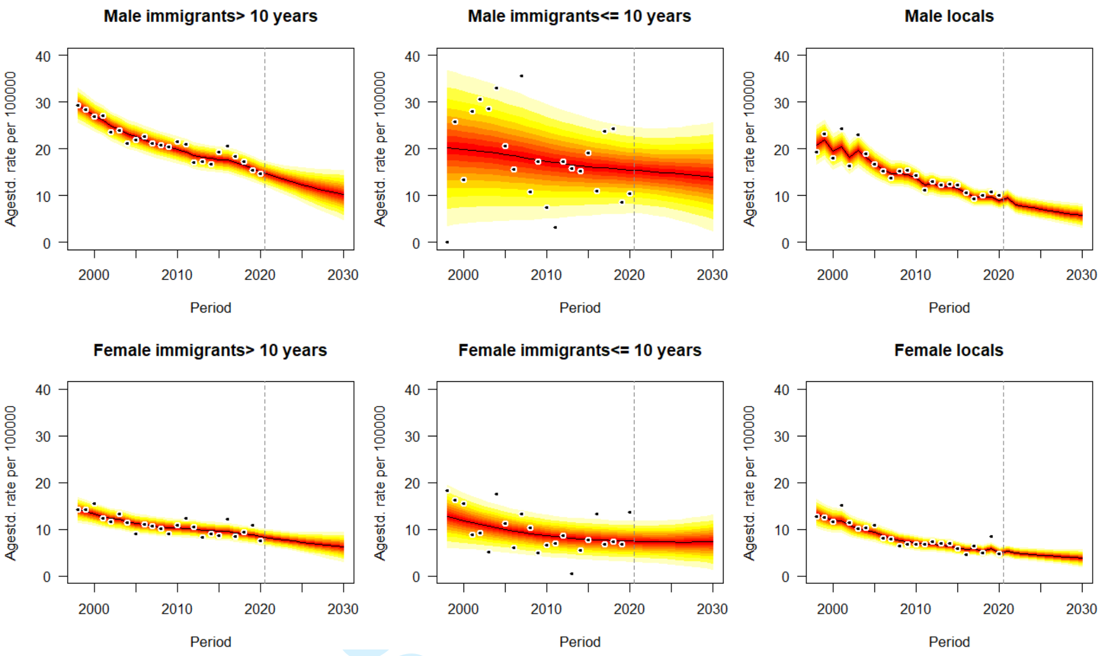


**eFigure 5(a).** Projections of stomach cancer mortality rates by gender and immigrant status from 2022 to 2030. Observations are shown as dots with the predictive distribution between the 5% and 95% quantile, whereby each lighter shade of red represents an additional 10% predictive CI. The predictive mean is shown as black solid line and the vertical dashed line indicates where prediction started.



For peer review only - http://bmjopen.bmj.com/site/about/guidelines.xhtml

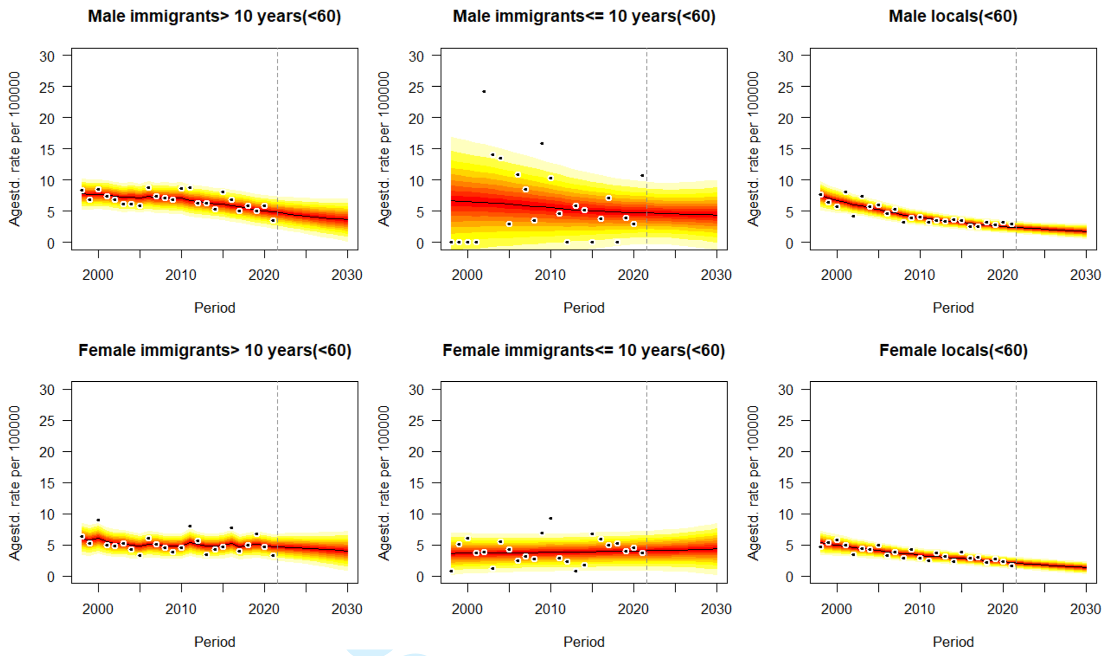


**eFigure 5(b).** Projections of stomach cancer mortality rates for the population younger than 60 by gender and immigrant status from 2022 to 2030. Observations are shown as dots with the predictive distribution between the 5% and 95% quantile, whereby each lighter shade of red represents an additional 10% predictive CI. The predictive mean is shown as black solid line and the vertical dashed line indicates where prediction started.



For peer review only - http://bmjopen.bmj.com/site/about/guidelines.xhtml

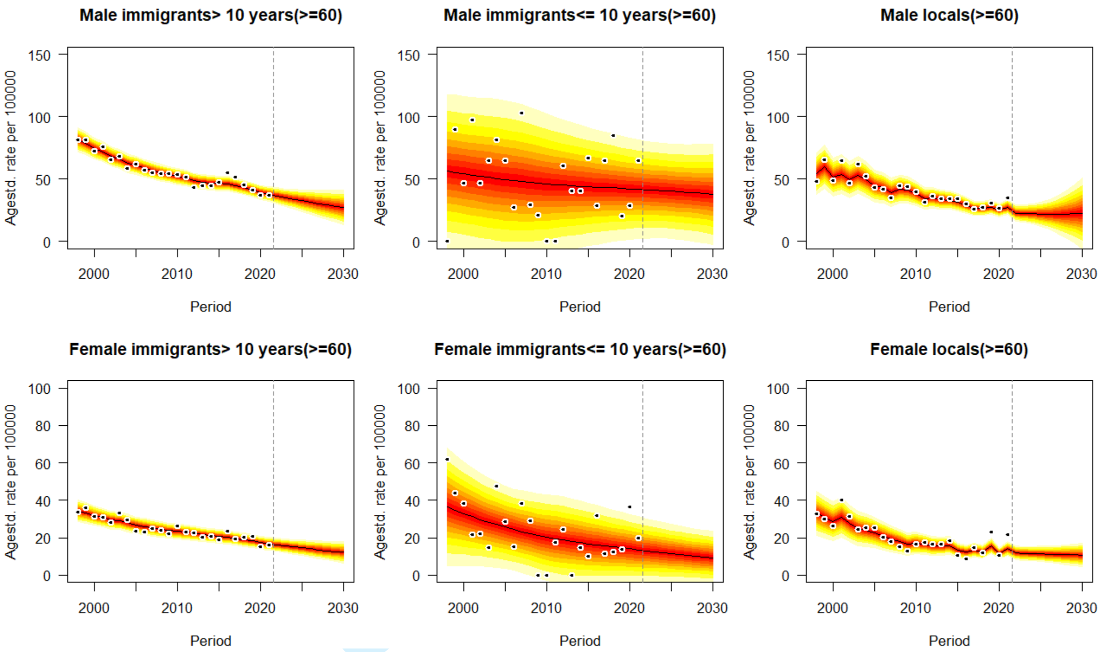


**eFigure 5(c).** Projections of stomach cancer mortality rates for the population older than or equal to 60 by gender and immigrant status from 2022 to 2030. Observations are shown as dots with the predictive distribution between the 5% and 95% quantile, whereby each lighter shade of red represents an additional 10% predictive CI. The predictive mean is shown as black solid line and the vertical dashed line indicates where prediction started.



For peer review only - http://bmjopen.bmj.com/site/about/guidelines.xhtml

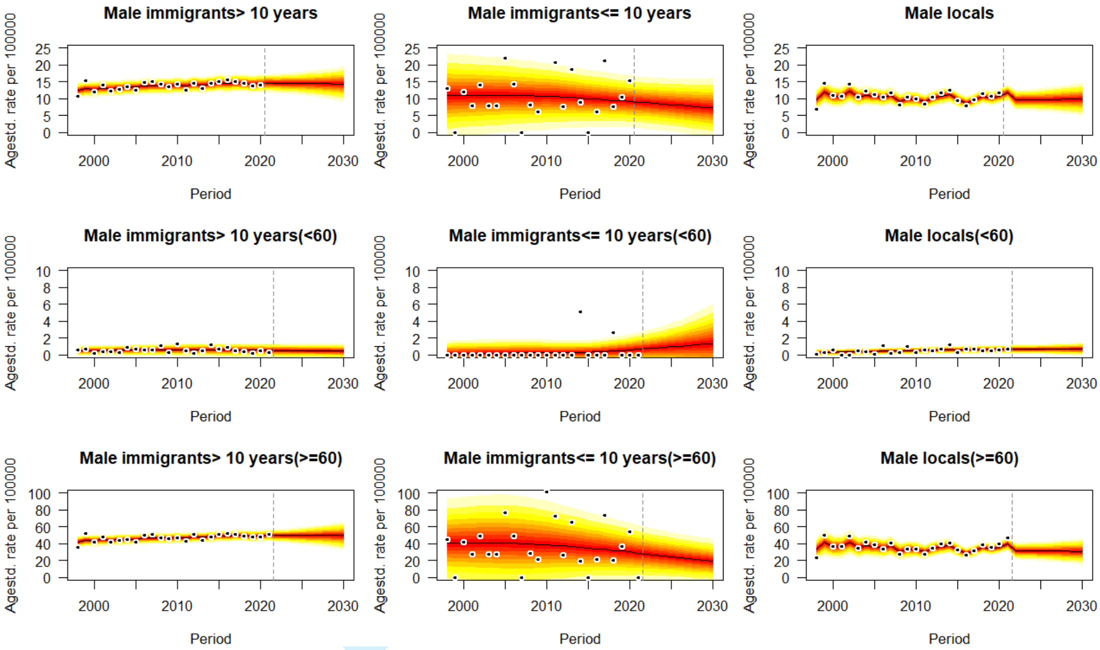


**eFigure 6.** Projections of prostate cancer mortality rates for males by immigrant status and age groups (less than, greater than or equal to 60 years old) from 2022 to 2030. Observations are shown as dots with the predictive distribution between the 5% and 95% quantile, whereby each lighter shade of red represents an additional 10% predictive CI. The predictive mean is shown as black solid line and the vertical dashed line indicates where prediction started.



For peer review only - http://bmjopen.bmj.com/site/about/guidelines.xhtml

| Predictive mean of age-standardized mortality rates of lung cancer per 100,000 population | | | | | | | | | | |
|---|---|---|---|---|---|---|---|---|---|---|
| Year | 2021 | 2022 | 2023 | 2024 | 2025 | 2026 | 2027 | 2028 | 2029 | 2030 |
| **Female immigrants >10** | 41.80 (1.27) | 41.34 (1.86) | 40.58 (2.27) | 39.87 (2.75) | 39.19 (3.28) | 38.53 (3.86) | 37.89 (4.46) | 37.26 (5.09) | 36.65 (5.74) | 36.04 (6.4) |
| **Female immigrants ≤ 10** | 23.92 (4.00) | 22.22 (4.67) | 20.56 (5.38) | 19.01 (6.10) | 17.57 (6.80) | 16.24 (7.45) | 15.00 (8.04) | 13.85 (8.56) | 12.79 (9.01) | 11.81 (9.39) |
| **Female locals** | 34.67 (1.76) | 30.22 (3.54) | 30.63 (4.77) | 31.05 (6.38) | 31.48 (8.29) | 31.9 (10.47) | 32.32 (12.87) | 32.73 (15.48) | 33.15 (18.31) | 33.55 (21.33) |
| **Male immigrants >10** | 102.90 (2.43) | 100.18 (4.18) | 97.18 (5.33) | 94.34 (6.72) | 91.71 (8.24) | 89.15 (9.84) | 86.66 (11.47) | 84.19 (13.11) | 81.81 (14.74) | 79.55 (16.37) |
| **Male immigrants ≤10** | 81.26 (9.21) | 79.90 (10.41) | 79.81 (11.82) | 79.72 (13.42) | 79.62 (15.19) | 79.50 (17.09) | 79.32 (19.09) | 79.08 (21.18) | 78.78 (23.32) | 78.41 (25.53) |
| **Male locals** | 60.96 (2.82) | 52.27 (4.86) | 50.83 (5.39) | 49.56 (6.13) | 48.18 (6.97) | 46.64 (7.84) | 45.13 (8.76) | 43.83 (9.76) | 42.67 (10.8) | 41.43 (11.8) |
| **Female immigrants>10(<60y)** | 15.51 (1.12) | 14.51 (1.50) | 13.90 (1.76) | 13.29 (2.04) | 12.71 (2.33) | 12.13 (2.62) | 11.57 (2.91) | 11.02 (3.18) | 10.49 (3.43) | 9.98 (3.68) |
| **Female immigrants ≤ 10(<60y)** | 8.14 (1.91) | 7.79 (1.95) | 7.18(2.23) | 6.62(2.53) | 6.10(2.81) | 5.63(3.08) | 5.19(3.32) | 4.79 (3.53) | 4.42 (3.72) | 4.09 (3.88) |
| **Female locals(<60y)** | 10.25 (0.77) | 9.48 (0.89) | 9.17(1.02) | 8.87(1.16) | 8.57(1.32) | 8.27(1.49) | 7.97(1.65) | 7.68 (1.82) | 7.38 (1.98) | 7.09 (2.13) |
| **Male immigrants>10(<60y)** | 27.81 (2.10) | 26.36 (3.58) | 24.96 (3.94) | 23.64 (4.35) | 22.38 (4.79) | 21.17 (5.23) | 20.03 (5.67) | 18.96 (6.10) | 17.96 (6.51) | 17.03 (6.90) |
| **Male immigrants ≤ 10(<60y)** | 15.01 (2.98) | 13.38 (3.71) | 12.02 (4.17) | 10.79 (4.59) | 9.68 (4.95) | 8.69 (5.24) | 7.79 (5.46) | 6.98 (5.61) | 6.25 (5.69) | 5.59 (5.72) |
| **Male locals(<60y)** | 15.19 (0.78) | 14.45 (1.15) | 14.03 (1.29) | 13.61 (1.46) | 13.14 (1.64) | 12.65 (1.82) | 12.13 (2.01) | 11.55 (2.17) | 10.93 (2.31) | 10.26 (2.43) |
| **Female immigrants >10(≥ 60y)** | 108.85 (4.80) | 107.21 (5.17) | 106.26 (6.24) | 105.52 (7.54) | 104.94 (9.04) | 104.51 (10.72) | 104.21 (12.57) | 104.07 (14.61) | 104.06 (16.78) | 104.16 (19.14) |
| **Female immigrants ≤ 10(≥60y)** | 66.16 (13.25) | 63.84 (15.72) | 59.88 (17.50) | 56.14 (19.31) | 52.60 (21.03) | 49.27 (22.66) | 46.14 (24.16) | 43.20 (25.52) | 40.44 (26.74) | 37.85 (27.81) |
| **Female locals(≥60y)** | 77.33 (9.40) | 76.53 (10.11) | 76.22 (10.85) | 75.94 (11.79) | 75.69 (12.94) | 75.49 (14.28) | 75.32 (15.80) | 75.19 (17.48) | 75.10 (19.33) | 75.03 (21.32) |
| **Male immigrants>10(≥60y)** | 293.56 (9.13) | 289.8 (11.7) | 286.6 (15.19) | 284.28 (19.51) | 282.78 (24.49) | 281.99 (30.07) | 281.88 (36.31) | 282.31 (43.15) | 283.37 (50.66) | 285.03 (58.86) |
| **Male immigrants ≤ 10(≥60y)** | 244.88 (30.29) | 247.01 (36.85) | 251.24 (42.94) | 255.62 (50.06) | 260.14 (58.14) | 264.82 (67.14) | 269.61 (77.01) | 274.52 (87.75) | 279.55 (99.34) | 284.69 (111.81) |
| **Male locals(≥60y)** | 150.75 (16.22) | 146.29 (18.46) | 143.54 (20.58) | 141.84 (23.97) | 140.07 (28.24) | 138.14 (33.39) | 136.65 (39.82) | 136.49 (47.87) | 137.24 (57.47) | 138.26 (68.52) |

**eTable 1.** Predictive means and standard deviations (in brackets) of age-standardized mortality rates of lung cancer per 100,000 population for each gender, age group (less than, greater or equal to 60 years old) and immigrant status from 2022 to 2030. Reported means and standard deviations (in brackets) of age-standardized mortality rates in 2021 are also listed.



For peer review only - http://bmjopen.bmj.com/site/about/guidelines.xhtml

| Predictive mean of age-standardized mortality rates of colon cancer per 100,000 population | | | | | | | | | | |
|---|---|---|---|---|---|---|---|---|---|---|
| **Year** | **2021** | **2022** | **2023** | **2024** | **2025** | **2026** | **2027** | **2028** | **2029** | **2030** |
| **Female immigrants >10** | 20.03 (0.95) | 18.95 (1.13) | 18.77 (1.37) | 18.59 (1.66) | 18.42 (1.98) | 18.27 (2.33) | 18.12 (2.71) | 17.98 (3.11) | 17.85 (3.53) | 17.73 (3.96) |
| **Female immigrants ≤ 10** | 8.11 (2.19) | 7.70 (2.51) | 7.25 (2.81) | 6.82 (3.11) | 6.42 (3.37) | 6.03 (3.61) | 5.67 (3.83) | 5.33 (4.01) | 5.01 (4.17) | 4.71 (4.31) |
| **Female locals** | 13.77 (1.30) | 13.47 (1.61) | 13.24 (1.72) | 13.01 (1.87) | 12.77 (2.04) | 12.53 (2.24) | 12.29 (2.46) | 12.06 (2.68) | 11.82 (2.92) | 11.59 (3.16) |
| **Male immigrants >10** | 31.22 (1.28) | 29.82 (1.46) | 29.66 (1.79) | 29.52 (2.19) | 29.41 (2.63) | 29.30 (3.11) | 29.21 (3.64) | 29.14 (4.19) | 29.06 (4.78) | 28.98 (5.39) |
| **Male immigrants ≤10** | 15.47 (2.14) | 16.77 (3.77) | 17.02 (4.18) | 17.23 (4.64) | 17.45 (5.14) | 17.67 (5.69) | 17.88 (6.27) | 18.09 (6.91) | 18.31 (7.56) | 18.50 (8.26) |
| **Male locals** | 21.28 (1.38) | 19.81 (2.07) | 19.39 (2.22) | 18.97 (2.42) | 18.57 (2.61) | 18.18 (2.85) | 17.81 (3.12) | 17.43 (3.40) | 17.06 (3.71) | 16.71 (4.03) |
| **Female immigrants >10(<60y)** | 7.09 (0.99) | 7.36 (1.12) | 7.46 (1.28) | 7.56 (1.46) | 7.65 (1.68) | 7.74 (1.92) | 7.83 (2.19) | 7.92 (2.48) | 8.01 (2.79) | 8.09 (3.13) |
| **Female immigrants ≤ 10(<60y)** | 3.11 (0.67) | 2.82 (0.86) | 2.65 (0.91) | 2.51 (0.97) | 2.36 (1.02) | 2.22 (1.07) | 2.08 (1.11) | 1.95 (1.14) | 1.83 (1.18) | 1.72 (1.22) |
| **Female locals(<60y)** | 4.10 (0.41) | 3.87 (0.50) | 3.73 (0.54) | 3.61 (0.59) | 3.47 (0.65) | 3.34 (0.70) | 3.22 (0.76) | 3.11 (0.82) | 2.99 (0.88) | 2.88 (0.94) |
| **Male immigrants >10(<60y)** | 8.29 (0.91) | 7.98 (1.17) | 7.85 (1.38) | 7.71 (1.60) | 7.54 (1.83) | 7.36 (2.08) | 7.17(2.32) | 6.97(2.57) | 6.76(2.81) | 6.55(3.05) |
| **Male immigrants ≤ 10(<60y)** | 5.03 (1.44) | 5.18 (1.58) | 5.22 (1.75) | 5.26 (1.93) | 5.30 (2.14) | 5.34 (2.36) | 5.38(2.59) | 5.43(2.84) | 5.47(3.11) | 5.51(3.38) |
| **Male locals(<60y)** | 5.14 (0.43) | 4.88 (0.63) | 4.66 (0.79) | 4.46 (0.96) | 4.26 (1.13) | 4.08 (1.31) | 3.91(1.48) | 3.73(1.65) | 3.57(1.82) | 3.42(1.97) |
| **Female immigrants >10(≥60y)** | 52.16 (2.59) | 49.21 (2.99) | 48.70 (3.56) | 48.26 (4.26) | 47.87 (5.05) | 47.54 (5.94) | 47.26 (6.90) | 47.05 (7.94) | 46.91 (9.06) | 46.81 (10.26) |
| **Female immigrants ≤ 10(≥60y)** | 24.01 (5.83) | 22.44 (6.56) | 21.69 (6.96) | 20.95 (7.38) | 20.23 (7.80) | 19.52 (8.23) | 18.84 (8.66) | 18.17 (9.08) | 17.51 (9.49) | 16.86 (9.90) |
| **Female locals(≥60y)** | 37.42 (5.31) | 36.69 (5.74) | 36.29 (6.06) | 35.87 (6.46) | 35.46 (6.95) | 35.04 (7.5) | 34.61 (8.12) | 34.19 (8.79) | 33.77 (9.51) | 33.34 (10.27) |
| **Male immigrants >10(≥60y)** | 84.17 (3.55) | 82.72 (4.09) | 82.16 (4.95) | 81.64 (5.97) | 81.19 (7.12) | 80.81 (8.39) | 80.47 (9.77) | 80.15 (11.24) | 79.85 (12.81) | 79.56 (14.45) |
| **Male immigrants ≤ 10(≥60y)** | 43.25 (11.07) | 44.93 (13.09) | 45.62 (14.52) | 46.30 (16.09) | 46.96 (17.80) | 47.61 (19.64) | 48.25 (21.62) | 48.88 (23.73) | 49.51 (25.97) | 50.13 (28.34) |
| **Male locals(≥60y)** | 55.79 (6.86) | 54.89 (7.65) | 53.75 (8.03) | 52.63 (8.52) | 51.54 (9.12) | 50.47 (9.8) | 49.43 (10.55) | 48.42 (11.37) | 47.42 (12.25) | 46.44 (13.16) |

**eTable 2.** Predictive means and standard deviations (in brackets) of age-standardized mortality rates of colon cancer per 100,000 population for each gender, age group (less than, greater or equal to 60 years old) and immigrant status from 2022 to 2030. Reported means and standard deviations (in brackets) of age-standardized mortality rates in 2021 are also listed.



For peer review only - http://bmjopen.bmj.com/site/about/guidelines.xhtml

| Predictive mean of age-standardized mortality rates of liver cancer per 100,000 population | | | | | | | | | | |
|---|---|---|---|---|---|---|---|---|---|---|
| Year | 2021 | 2022 | 2023 | 2024 | 2025 | 2026 | 2027 | 2028 | 2029 | 2030 |
| Female immigrants >10 | 11.34 (0.66) | 10.68 (0.71) | 10.09 (0.85) | 9.54 (1.01) | 9.01 (1.16) | 8.50 (1.31) | 8.02(1.45) | 7.57(1.59) | 7.14(1.72) | 6.74(1.83) |
| Female immigrants ≤10 | 9.15 (1.55) | 8.66 (1.82) | 8.38 (1.95) | 8.11 (2.08) | 7.84 (2.22) | 7.58 (2.36) | 7.32(2.49) | 7.07(2.63) | 6.82(2.76) | 6.58(2.88) |
| Female locals | 6.72 (0.69) | 6.36 (0.88) | 6.08 (0.90) | 5.81 (0.93) | 5.53 (0.97) | 5.26 (1.01) | 5.01(1.06) | 4.77(1.11) | 4.53(1.15) | 4.3(1.21) |
| Male immigrants >10 | 52.17 (1.78) | 49.22 (2.36) | 47.76 (2.93) | 46.35 (3.59) | 45.01 (4.31) | 43.67 (5.05) | 42.37 (5.81) | 41.1(6.56) | 39.89 (7.33) | 38.71 (8.08) |
| Male immigrants ≤10 | 42.33 (5.87) | 39.03 (6.49) | 37.39 (7.47) | 35.81 (8.51) | 34.26 (9.58) | 32.76 (10.63) | 31.31 (11.65) | 29.91 (12.62) | 28.56 (13.54) | 27.25 (14.40) |
| Male locals | 24.22 (1.77) | 22.16 (2.09) | 21.02 (2.22) | 19.91 (2.39) | 18.85 (2.58) | 17.83 (2.79) | 16.85 (3.03) | 15.92 (3.21) | 15.03 (3.40) | 14.18 (3.59) |
| Female immigrants >10(<60y) | 3.62 (0.45) | 3.39 (0.52) | 3.29 (0.57) | 3.20 (0.63) | 3.12 (0.69) | 3.04 (0.75) | 2.96(0.82) | 2.89(0.89) | 2.82(0.96) | 2.75(1.03) |
| Female immigrants ≤ 10(<60y) | 4.10 (0.79) | 3.81 (0.91) | 3.69 (0.96) | 3.57 (1.02) | 3.46 (1.08) | 3.36 (1.15) | 3.25(1.22) | 3.15(1.29) | 3.06(1.36) | 2.97(1.43) |
| Female locals(<60y) | 1.50 (0.13) | 1.37 (0.2) | 1.29 (0.21) | 1.22 (0.23) | 1.16 (0.24) | 1.10 (0.26) | 1.04(0.27) | 0.99(0.29) | 0.94(0.30) | 0.89(0.31) |
| Male immigrants >10(<60y) | 26.32 (2.11) | 24.04 (2.35) | 23.02 (2.63) | 22.05 (2.94) | 21.13 (3.27) | 20.25 (3.61) | 19.41 (3.95) | 18.62 (4.30) | 17.86 (4.64) | 17.14 (4.98) |
| Male immigrants ≤ 10(<60y) | 25.52 (2.99) | 22.56 (3.96) | 21.71 (4.44) | 20.87 (4.94) | 20.04 (5.45) | 19.22 (5.95) | 18.42 (6.45) | 17.63 (6.91) | 16.86 (7.36) | 16.11 (7.78) |
| Male locals(<60y) | 8.25 (0.69) | 7.47 (0.74) | 6.97 (0.79) | 6.52 (0.86) | 6.11 (0.93) | 5.73 (1.01) | 5.38(1.08) | 5.04(1.15) | 4.73(1.21) | 4.44(1.27) |
| Female immigrants >10(≥ 60y) | 33.67 (1.88) | 29.63 (2.01) | 27.99 (2.36) | 26.42 (2.75) | 24.92 (3.14) | 23.49 (3.52) | 22.13 (3.88) | 20.85 (4.23) | 19.64 (4.55) | 18.50 (4.85) |
| Female immigrants ≤10(≥ 60y) | 21.72 (5.11) | 19.08 (5.81) | 18.38 (6.14) | 17.71 (6.48) | 17.03 (6.83) | 16.39 (7.16) | 15.76 (7.49) | 15.16 (7.80) | 14.57 (8.11) | 14.01 (8.39) |
| Female locals(≥60y) | 20.63 (3.03) | 18.41 (3.23) | 17.55 (3.26) | 16.72 (3.32) | 15.91 (3.40) | 15.11 (3.49) | 14.34 (3.59) | 13.59 (3.69) | 12.87 (3.81) | 12.17 (3.93) |
| Male immigrants >10(≥ 60y) | 115.39 (4.54) | 113.96 (5.95) | 113.43 (7.65) | 113.17 (9.70) | 113.16 (12.04) | 113.37 (14.66) | 113.79 (17.56) | 114.39 (20.73) | 115.19 (24.18) | 116.17 (27.91) |
| Male immigrants ≤10(≥ 60y) | 88.61 (15.58) | 85.14 (18.85) | 82.59 (20.6) | 80.02 (22.44) | 77.42 (24.34) | 74.83 (26.24) | 72.23 (28.12) | 69.64 (29.94) | 67.07 (31.70) | 64.52 (33.38) |
| Male locals(≥60y) | 62.88 (5.97) | 58.95 (7.91) | 56.51 (8.20) | 54.14 (8.61) | 51.84 (9.12) | 49.61 (9.70) | 47.46 (10.33) | 45.38 (11.01) | 43.38 (11.68) | 41.45 (12.36) |

**eTable 3.** Predictive means and standard deviations (in brackets) of age-standardized mortality rates of liver cancer per 100,000 population for each gender, age group (less than, greater or equal to 60 years old) and immigrant status from 2022 to 2030. Reported means and standard deviations (in brackets) of age-standardized mortality rates in 2021 are also listed.



For peer review only - http://bmjopen.bmj.com/site/about/guidelines.xhtml

For peer review only

| Predictive mean of age-standardized mortality rates of pancreatic cancer per 100,000 population | | | | | | | | | | |
|---|---|---|---|---|---|---|---|---|---|---|
| **Year** | **2021** | **2022** | **2023** | **2024** | **2025** | **2026** | **2027** | **2028** | **2029** | **2030** |
| **Female immigrants >10** | 10.89 (0.62) | 11.11 (0.75) | 11.36 (0.91) | 11.61 (1.09) | 11.87 (1.31) | 12.14 (1.56) | 12.42 (1.84) | 12.71 (2.15) | 13.01 (2.48) | 13.3(2.85) |
| **Female immigrants ≤ 10** | 5.51 (1.44) | 5.44 (1.56) | 5.44 (1.69) | 5.43(1.84) | 5.42(1.99) | 5.41(2.15) | 5.39(2.32) | 5.36(2.49) | 5.34(2.66) | 5.31(2.84) |
| **Female locals** | 8.79 (1.10) | 9.01 (1.22) | 9.15 (1.34) | 9.29(1.48) | 9.43(1.64) | 9.57(1.83) | 9.71(2.05) | 9.85(2.28) | 9.99(2.54) | 10.14 (2.83) |
| **Male immigrants >10** | 16.30 (0.98) | 17.87 (1.19) | 18.48 (1.49) | 19.11 (1.87) | 19.78 (2.32) | 20.47 (2.83) | 21.18 (3.42) | 21.92 (4.07) | 22.69 (4.81) | 23.49 (5.61) |
| **Male immigrants ≤10** | 8.10 (2.02) | 7.87 (2.37) | 7.76 (2.53) | 7.64(2.70) | 7.51(2.87) | 7.38(3.05) | 7.24(3.23) | 7.09(3.41) | 6.95(3.58) | 6.81(3.75) |
| **Male locals** | 11.97 (1.26) | 12.29 (1.49) | 12.49 (1.64) | 12.69 (1.83) | 12.91 (2.06) | 13.11 (2.33) | 13.33 (2.63) | 13.55 (2.97) | 13.78 (3.34) | 14.02 (3.74) |
| **Female immigrants >10(<60y)** | 3.47 (0.33) | 3.62 (0.57) | 3.74 (0.66) | 3.87(0.77) | 4.01(0.89) | 4.14(1.02) | 4.28(1.18) | 4.42(1.34) | 4.57(1.53) | 4.72(1.73) |
| **Female immigrants ≤ 10(<60y)** | 1.12 (0.33) | 1.21 (0.48) | 1.22 (0.52) | 1.23(0.56) | 1.24(0.61) | 1.25(0.66) | 1.26(0.71) | 1.26(0.77) | 1.27(0.83) | 1.28(0.89) |
| **Female locals(<60y)** | 2.76 (0.27) | 2.88 (0.36) | 2.91 (0.41) | 2.93(0.48) | 2.96(0.55) | 2.99(0.63) | 3.02(0.71) | 3.04(0.81) | 3.07(0.90) | 3.10(1.01) |
| **Male immigrants >10(<60y)** | 6.88 (0.98) | 7.05 (1.11) | 7.24 (1.32) | 7.43(1.56) | 7.62(1.84) | 7.82(2.16) | 8.01(2.50) | 8.21(2.88) | 8.40(3.30) | 8.61(3.75) |
| **Male immigrants ≤ 10(<60y)** | 2.20 (0.71) | 2.01 (0.85) | 1.95 (0.91) | 1.9(0.94) | 1.84(0.99) | 1.79(1.04) | 1.74(1.09) | 1.69(1.14) | 1.64(1.19) | 1.60(1.24) |
| **Male locals(<60y)** | 4.16 (0.35) | 4.33 (0.48) | 4.41 (0.57) | 4.46(0.68) | 4.53(0.81) | 4.61(0.94) | 4.69(1.09) | 4.77(1.26) | 4.85(1.44) | 4.93(1.63) |
| **Female immigrants >10(≥60y)** | 28.58 (1.83) | 29.45 (2.11) | 29.91 (2.54) | 30.38 (3.06) | 30.85 (3.66) | 31.33 (4.33) | 31.81 (5.08) | 32.29 (5.91) | 32.78 (6.79) | 33.27 (7.74) |
| **Female immigrants ≤ 10(≥60y)** | 16.79 (5.29) | 15.65 (6.08) | 15.49 (6.71) | 15.33 (7.36) | 15.16 (8.03) | 14.97 (8.73) | 14.79 (9.43) | 14.59 (10.14) | 14.39 (10.86) | 14.19 (11.58) |
| **Female locals(≥60y)** | 22.80 (4.23) | 23.85 (4.46) | 24.21 (4.81) | 24.56 (5.23) | 24.91 (5.73) | 25.25 (6.30) | 25.58 (6.95) | 25.90 (7.67) | 26.22 (8.47) | 26.54 (9.34) |
| **Male immigrants >10(≥60y)** | 42.70 (2.55) | 44.36 (3.02) | 45.85 (3.76) | 47.41 (4.69) | 49.04 (5.78) | 50.73 (7.05) | 52.48 (8.50) | 54.28 (10.13) | 56.16 (11.95) | 58.11 (13.98) |
| **Male immigrants ≤ 10(≥60y)** | 24.68 (8.21) | 23.96 (9.01) | 23.87 (9.74) | 23.75 (10.52) | 23.61 (11.33) | 23.45 (12.17) | 23.28 (13.04) | 23.09 (13.93) | 22.89 (14.83) | 22.68 (15.75) |
| **Male locals(≥60y)** | 30.10 (4.68) | 31.17 (5.22) | 31.55 (5.63) | 31.93 (6.14) | 32.30 (6.75) | 32.66 (7.45) | 33.01 (8.23) | 33.35 (9.11) | 33.69 (10.08) | 34.03 (11.12) |

**eTable 4.** Predictive means and standard deviations (in brackets) of age-standardized mortality rates of pancreatic cancer per 100,000 population for each gender, age group (less than, greater or equal to 60 years old) and immigrant status from 2022 to 2030. Reported means and standard deviations (in brackets) of age-standardized mortality rates in 2021 are also listed.



For peer review only - http://bmjopen.bmj.com/site/about/guidelines.xhtml

| Predictive mean of age-standardized mortality rates of stomach cancer per 100,000 population | | | | | | | | | | |
|---|---|---|---|---|---|---|---|---|---|---|
| **Year** | **2021** | **2022** | **2023** | **2024** | **2025** | **2026** | **2027** | **2028** | **2029** | **2030** |
| **Female immigrants >10** | 8.20 (0.55) | 7.95 (0.62) | 7.71 (0.74) | 7.47 (0.87) | 7.25 (1.01) | 7.03 (1.15) | 6.83 (1.29) | 6.62 (1.43) | 6.43 (1.57) | 6.24 (1.71) |
| **Female immigrants ≤ 10** | 7.51 (1.44) | 7.36 (1.56) | 7.33 (1.69) | 7.30 (1.85) | 7.28 (2.01) | 7.27 (2.20) | 7.27 (2.40) | 7.28 (2.61) | 7.31 (2.84) | 7.33 (3.09) |
| **Female locals** | 5.26 (0.40) | 4.91 (0.52) | 4.75 (0.57) | 4.61 (0.63) | 4.47 (0.71) | 4.34 (0.77) | 4.21 (0.84) | 4.08 (0.91) | 3.95 (0.99) | 3.83 (1.06) |
| **Male immigrants >10** | 15.22 (0.64) | 13.89 (0.97) | 13.34(1.21) | 12.81 (1.46) | 12.31 (1.73) | 11.83 (1.99) | 11.38 (2.26) | 10.95 (2.51) | 10.54 (2.76) | 10.15 (3.01) |
| **Male immigrants ≤10** | 15.83 (3.04) | 15.21 (3.38) | 15.07 (3.67) | 14.93 (3.98) | 14.79 (4.31) | 14.64 (4.65) | 14.51 (5.02) | 14.35 (5.39) | 14.19 (5.78) | 14.03 (6.17) |
| **Male locals** | 8.14 (0.89) | 8.07 (0.99) | 7.73 (1.03) | 7.41(1.07) | 7.10 (1.13) | 6.81 (1.19) | 6.51 (1.26) | 6.23 (1.33) | 5.97 (1.39) | 5.71 (1.46) |
| **Female immigrants >10(<60y)** | 4.81 (0.56) | 4.69 (0.79) | 4.62 (0.87) | 4.55 (0.96) | 4.47 (1.07) | 4.39 (1.17) | 4.31 (1.29) | 4.22 (1.41) | 4.13 (1.52) | 4.03 (1.64) |
| **Female immigrants ≤ 10(<60y)** | 3.89 (0.80) | 4.08 (0.93) | 4.10 (1.03) | 4.13 (1.14) | 4.17 (1.27) | 4.21 (1.41) | 4.24 (1.55) | 4.28 (1.70) | 4.32 (1.87) | 4.36 (2.05) |
| **Female locals(<60y)** | 2.28 (0.21) | 2.08 (0.27) | 1.98 (0.29) | 1.88 (0.32) | 1.79 (0.35) | 1.71 (0.37) | 1.61 (0.41) | 1.53 (0.43) | 1.44 (0.45) | 1.37 (0.47) |
| **Male immigrants >10(<60y)** | 4.94 (0.57) | 4.71 (0.79) | 4.55 (0.89) | 4.41 (0.99) | 4.25 (1.10) | 4.12 (1.21) | 3.98 (1.32) | 3.86 (1.43) | 3.74 (1.54) | 3.63 (1.65) |
| **Male immigrants ≤ 10(<60y)** | 4.81 (1.31) | 4.70 (1.42) | 4.66 (1.55) | 4.63 (1.69) | 4.59 (1.83) | 4.55 (1.99) | 4.52 (2.15) | 4.48 (2.32) | 4.44 (2.50) | 4.41 (2.68) |
| **Male locals(<60y)** | 2.48 (0.21) | 2.37 (0.29) | 2.28 (0.32) | 2.21 (0.35) | 2.12 (0.38) | 2.04 (0.42) | 1.97 (0.45) | 1.91 (0.49) | 1.83 (0.52) | 1.77(0.55) |
| **Female immigrants >10(≥60y)** | 17.80 (1.04) | 16.23 (1.26) | 15.65 (1.47) | 15.08 (1.70) | 14.55 (1.94) | 14.03 (2.18) | 13.54 (2.43) | 13.07 (2.68) | 12.62 (2.92) | 12.19 (3.16) |
| **Female immigrants ≤ 10(≥60y)** | 14.72 (4.29) | 13.01 (4.83) | 12.52 (5.11) | 12.03 (5.37) | 11.55 (5.63) | 11.08 (5.88) | 10.63 (6.12) | 10.19 (6.35) | 9.76(6.56) | 9.34 (6.75) |
| **Female locals(≥60y)** | 12.20 (1.66) | 11.86 (1.84) | 11.67 (1.98) | 11.49 (2.15) | 11.33 (2.35) | 11.18 (2.58) | 11.04 (2.84) | 10.91 (3.11) | 10.79(3.4) | 10.68 (3.71) |
| **Male immigrants >10(≥ 60y)** | 37.23 (2.29) | 36.59 (2.56) | 35.17(3.18) | 33.82 (3.86) | 32.55 (4.57) | 31.34 (5.28) | 30.19 (6.01) | 29.08 (6.70) | 28.02 (7.40) | 27.01 (8.07) |
| **Male immigrants ≤ 10(≥60y)** | 42.30 (10.88) | 41.43 (11.78) | 41.03 (12.71) | 40.61 (13.70) | 40.17 (14.75) | 39.71 (15.85) | 39.24 (16.99) | 38.75 (18.16) | 38.23 (19.35) | 37.71 (20.57) |
| **Male locals(≥60y)** | 23.04 (3.29) | 22.69 (3.56) | 22.37(4.07) | 22.16(4.84) | 21.89 (5.86) | 21.61 (7.22) | 21.52 (9.02) | 21.74 (11.29) | 22.17 (14.03) | 22.73 (17.28) |

**eTable 5.** Predictive means and standard deviations (in brackets) of age-standardized mortality rates of stomach cancer per 100,000 population for each gender, age group (less than, greater or equal to 60 years old) and immigrant status from 2022 to 2030. Reported means and standard deviations (in brackets) of age-standardized mortality rates in 2021 are also listed.

For peer review only



For peer review only - http://bmjopen.bmj.com/site/about/guidelines.xhtml

| Predictive mean of age-standardized mortality rates of prostate cancer per 100,000 population | | | | | | | | | | |
|---|---|---|---|---|---|---|---|---|---|---|
| Year | 2021 | 2022 | 2023 | 2024 | 2025 | 2026 | 2027 | 2028 | 2029 | 2030 |
| Male immigrants >10 | 14.81 (0.61) | 14.59 (0.79) | 14.57 (0.96) | 14.56 (1.15) | 14.54 (1.37) | 14.51 (1.61) | 14.48 (1.86) | 14.45 (2.13) | 14.42 (2.42) | 14.38 (2.72) |
| Male immigrants ≤10 | 9.03 (2.95) | 8.78 (3.11) | 8.58 (3.29) | 8.39 (3.49) | 8.19(3.69) | 8.10(3.89) | 7.82(4.11) | 7.63(4.31) | 7.45(4.51) | 7.27(4.72) |
| Male locals | 9.54 (1.40) | 9.66 (1.57) | 9.67 (1.66) | 9.69 (1.77) | 9.72(1.91) | 9.75(2.06) | 9.78(2.23) | 9.82(2.43) | 9.86(2.64) | 9.9(2.88) |
| Male immigrants >10(<60y) | 0.57 (0.12) | 0.52 (0.17) | 0.51 (0.19) | 0.50 (0.21) | 0.49(0.22) | 0.48(0.24) | 0.47(0.25) | 0.46(0.27) | 0.45(0.29) | 0.44(0.31) |
| Male immigrants ≤ 10(<60y) | 0.65 (0.59) | 0.73 (0.77) | 0.81 (0.93) | 0.87 (1.10) | 0.94(1.31) | 1.01(1.51) | 1.09(1.75) | 1.16(2.02) | 1.24(2.32) | 1.33(2.64) |
| Male locals(<60y) | 0.63 (0.12) | 0.66 (0.14) | 0.66 (0.16) | 0.66 (0.19) | 0.67(0.21) | 0.67(0.24) | 0.67(0.27) | 0.68(0.31) | 0.68(0.33) | 0.69(0.37) |
| Male immigrants >10(≥ 60y) | 49.43 (2.59) | 49.61 (2.73) | 49.63 (3.29) | 49.64 (3.94) | 49.64 (4.68) | 49.64 (5.51) | 49.63 (6.38) | 49.62 (7.32) | 49.61 (8.32) | 49.58(9.3 7) |
| Male immigrants ≤ 10(≥60y) | 28.29 (9.15) | 27.66 (9.78) | 26.53 (10.21) | 25.4 (10.63) | 24.28 (11.03) | 23.16 (11.41) | 22.07 (11.76) | 21.01 (12.09) | 19.96 (12.38) | 18.95(12. 63) |
| Male locals(≥60y) | 31.57 (5.17) | 31.48 (5.49) | 31.40 (5.76) | 31.32 (6.09) | 31.24 (6.48) | 31.15 (6.94) | 31.06 (7.44) | 30.96 (8.01) | 30.86 (8.61) | 30.74(9.2 6) |

**eTable 6.** Predictive means and standard deviations (in brackets) of age-standardized mortality rates of prostate cancer per 100,000 population for each age group (less than, greater or equal to 60 years old) and immigrant status from 2022 to 2030. Reported means and standard deviations (in brackets) of age-standardized mortality rates in 2021 are also listed.

For peer review only

# Reporting checklist for cohort study.

Based on the STROBE cohort guidelines.

## Instructions to authors

Complete this checklist by entering the page numbers from your manuscript where readers will find each of the items listed below.

Your article may not currently address all the items on the checklist. Please modify your text to include the missing information. If you are certain that an item does not apply, please write "n/a" and provide a short explanation.

Upload your completed checklist as an extra file when you submit to a journal.

In your methods section, say that you used the STROBE cohortreporting guidelines, and cite them as:

von Elm E, Altman DG, Egger M, Pocock SJ, Gotzsche PC, Vandenbroucke JP. The Strengthening the Reporting of Observational Studies in Epidemiology (STROBE) Statement: guidelines for reporting observational studies.

| | | Reporting Item | Page Number |
|---|---|---|---|
| **Title and abstract** | | | |
| Title | #1a | Indicate the study's design with a commonly used term in the title or the abstract | 1 |
| Abstract | #1b | Provide in the abstract an informative and balanced summary of what was done and what was found | 2 |
| **Introduction** | | | |
| Background / rationale | #2 | Explain the scientific background and rationale for the investigation being reported | 4 |
| Objectives | #3 | State specific objectives, including any prespecified hypotheses | 4 |
| **Methods** | | | |
| Study design | #4 | Present key elements of study design early in the paper | 5 |
| Setting | #5 | Describe the setting, locations, and relevant dates, including periods | 5 |

For peer review only

| | | | |
|---|---|---|---|
| | | of recruitment, exposure, follow-up, and data collection | |
| Eligibility criteria | #6a | Give the eligibility criteria, and the sources and methods of selection of participants. Describe methods of follow-up. | 5 |
| Eligibility criteria | #6b | For matched studies, give matching criteria and number of exposed and unexposed | n/a |
| Variables | #7 | Clearly define all outcomes, exposures, predictors, potential confounders, and effect modifiers. Give diagnostic criteria, if applicable | 5 |
| Data sources / measurement | #8 | For each variable of interest give sources of data and details of methods of assessment (measurement). Describe comparability of assessment methods if there is more than one group. Give information separately for for exposed and unexposed groups if applicable. | 5 |
| Bias | #9 | Describe any efforts to address potential sources of bias | 5 |
| Study size | #10 | Explain how the study size was arrived at | 5 |
| Quantitative variables | #11 | Explain how quantitative variables were handled in the analyses. If applicable, describe which groupings were chosen, and why | 5 |
| Statistical methods | #12a | Describe all statistical methods, including those used to control for confounding | |
| 5 | | | |
| Statistical methods | #12b | Describe any methods used to examine subgroups and interactions | 5 |
| Statistical methods | #12c | Explain how missing data were addressed | 5 |
| Statistical methods | #12d | If applicable, explain how loss to follow-up was addressed | n/a |
| Statistical methods | #12e | Describe any sensitivity analyses | |
| n/a | | | |
| **Results** | | | |
| Participants | #13a | Report numbers of individuals at each stage of study—eg numbers potentially eligible, examined for eligibility, confirmed eligible, | n/a |

For peer review only

| | | | |
|---|---|---|---|
| | | included in the study, completing follow-up, and analysed. Give information separately for for exposed and unexposed groups if applicable. | |
| Participants | #13b | Give reasons for non-participation at each stage | 5 |
| Participants | #13c | Consider use of a flow diagram | |
| n/a | | | |
| Descriptive data | #14a | Give characteristics of study participants (eg demographic, clinical, social) and information on exposures and potential confounders. Give information separately for exposed and unexposed groups if applicable. | 5 |
| Descriptive data | #14b | Indicate number of participants with missing data for each variable of interest | |
| n/a | | | |
| Descriptive data | #14c | Summarise follow-up time (eg, average and total amount) | |
| n/a | | | |
| Outcome data | #15 | Report numbers of outcome events or summary measures over time. Give information separately for exposed and unexposed groups if applicable. | |
| n/a | | | |
| Main results | #16a | Give unadjusted estimates and, if applicable, confounder-adjusted estimates and their precision (eg, 95% confidence interval). Make clear which confounders were adjusted for and why they were included | 6 |
| Main results | #16b | Report category boundaries when continuous variables were categorized | n/a |
| Main results | #16c | If relevant, consider translating estimates of relative risk into absolute risk for a meaningful time period | |
| n/a | | | |
| Other analyses | #17 | Report other analyses done—eg analyses of subgroups and interactions, and sensitivity analyses | 7 |

**Discussion**

For peer review only

| | | | |
|---|---|---|---|
| Key results | #18 | Summarise key results with reference to study objectives | 8 |
| Limitations | #19 | Discuss limitations of the study, taking into account sources of potential bias or imprecision. Discuss both direction and magnitude of any potential bias. | 10 |
| Interpretation | #20 | Give a cautious overall interpretation considering objectives, limitations, multiplicity of analyses, results from similar studies, and other relevant evidence. | 8 |
| Generalisability | #21 | Discuss the generalisability (external validity) of the study results | 9 |
| **Other Information** | | | |
| Funding | #22 | Give the source of funding and the role of the funders for the present study and, if applicable, for the original study on which the present article is based | 11 |

The STROBE checklist is distributed under the terms of the Creative Commons Attribution License CC-BY. This checklist was completed on 11. February 2023 using https://www.goodreports.org/, a tool made by the EQUATOR Network in collaboration with Penelope.ai

For peer review only
